# Oral small molecule GLP-1 receptor agonist aleniglipron in people with overweight or obesity: a randomized, double-blind, placebo-controlled phase 2b trial

**DOI:** 10.1038/s41591-026-04476-6

**Published:** 2026-06-05

**Authors:** Julio Rosenstock, Ildiko Lingvay, Donna Ryan, Ania M. Jastreboff, Robert Kushner, Andres Acosta, Thomas C. Blevins, Melissa Choi, Faith L. Holmes, Timothy Smith, Lisa Connery, Yao Li, Mike Ke Liu, Aline Barth, Jamie Butcher, Antonio Civitarese, Huibin Yue, Blai Coll

**Affiliations:** 1 The University of Texas Southwestern Medical Center, Dallas, TX USA; 2 University of Texas Southwestern Medical Center, Internal Medicine, Dallas, TX USA; 3 Pennington Biomedical Research Foundation, Baton Rouge, LA USA; 4 Yale School of Medicine, Internal Medicine, Endocrinology, New Haven, CT USA; 5 Northwestern University Feinberg School of Medicine, Chicago, IL USA; 6 Mayo Clinic, Medicine, Rochester, MN USA; 7 Texas Diabetes and Endocrinology, Austin, TX USA; 8 Trialmed, Minneapolis, MN USA; 9 Elligo Health Research, Austin, TX USA; 10 StudyMetrix Research, St. Peters, MO USA; 11 Alliance for Multispecialty Research, Knoxville, TN USA; 12 Structure Therapeutics, South San Francisco, CA USA

**Keywords:** Drug development, Obesity

## Abstract

Aleniglipron is an oral, small-molecule glucagon-like peptide-1 receptor agonist (GLP1-RA) in development for obesity treatment. The ACCESS phase 2b placebo-controlled, double-blind study randomized 230 adults (mean BMI 39.5 kg m^−2^, 54% female) with obesity or overweight to examine the effects of once-daily aleniglipron escalated every 4 weeks to 45, 90 or 120 mg. At week 36, the trial met its primary endpoint with a placebo-adjusted LS mean (95% confidence interval) body-weight change from baseline of −8.2% (−11.1 to −5.3%), −9.8% (−12.5 to −7.2%) and −11.3% (−13.9 to −8.6%) for the aleniglipron 45-, 90- and 120-mg arms, respectively (*P* < 0.0001, all doses versus placebo), with no apparent weight-loss plateau at the end of the double-blind period. Continued weight loss was observed at the interim analysis (median treatment duration of 20 weeks) of the ongoing open-label extension. Gastrointestinal events were generally mild to moderate and decreased in frequency over time, with little to no recurrence of vomiting after reintroduction following permitted dose interruptions. Treatment-related discontinuations were 10.4% across aleniglipron arms, with no events of drug-induced liver injury. Clinically relevant weight reductions of up to 11.3% with a tolerability profile consistent with the GLP-1RA class support further development of aleniglipron for obesity treatment. ClinicalTrials.gov registration: NCT06693843.

## Abstract (web summary)

As presented at the American Diabetes Association Meeting: this randomized phase 2b trial in 230 adults with overweight or obesity shows that aleniglipron, an oral small-molecule GLP1-RA, led to up to 11.3% body-weight loss compared with placebo after 36 weeks of treatment.

## Main

The incidence of obesity is increasing globally, leading to elevated risk for mortality and comorbidities and increased healthcare costs. GLP-1 RAs that mimic the effects of GLP-1, an incretin hormone that decreases appetite, increases satiety and delays gastric emptying, have been approved for chronic weight management, as well as for type 2 diabetes. In addition, GLP-1RAs have been demonstrated to reduce cardiovascular risk, improve metabolic-dysfunction-associated steatohepatitis (MASH) and moderate-to-severe sleep apnea and consistently show clinically meaningful improvements in markers of systemic inflammation^1–7^. Available peptide-based GLP-1 RAs are increasingly used for obesity treatment but are not widely accessible to broader populations. Most GLP-1 RAs are injectable, which limits patient access because of resistance to this method of administration, inconvenient storage procedures (refrigeration) and scalability limitations due to complex manufacturing processes and cost considerations.

Small-molecule GLP-1 RAs can be orally administered, can be manufactured at scale and could address access and treatment gaps. Several oral GLP-1 RA compounds at different stages of clinical development, including two which are FDA-approved, oral semaglutide and orforglipron, with the latter being a small molecule demonstrating −12.4% body weight loss by 72 weeks (refs. ^1–3,5,8,9^). Oral therapies with this degree of effectiveness could offer more convenient obesity treatments with broader accessibility.

**Extended Data Fig. 1:**
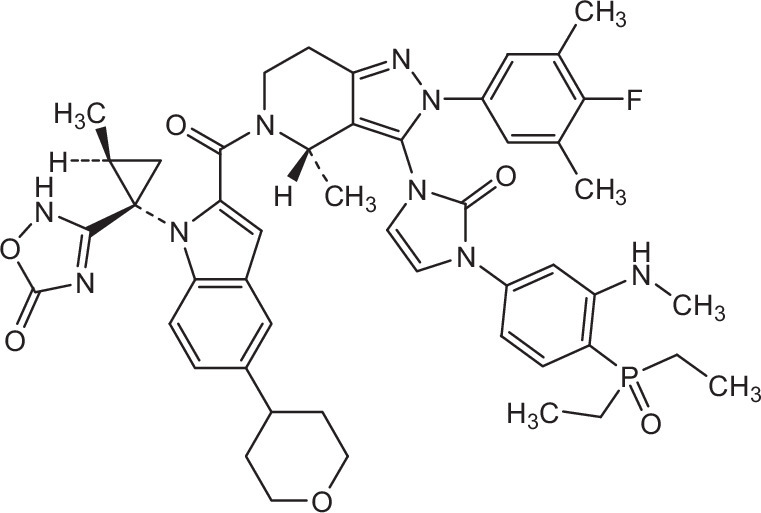
Aleniglipron compound structure. Aleniglipron (GSBR-1290) is a potent, β-arrestin-biased, highly selective small molecule GLP-1 RA. Using structure-based drug discovery, aleniglipron was designed to optimize substituent positioning and provide conformational stability, eliminate chiral centers requiring chiral separation supporting efficient synthesis, oral exposure, and selective and biased receptor engagement.

Aleniglipron (GSBR-1290) is a potent, highly selective small-molecule GLP-1 RA in development for obesity treatment. Aleniglipron was designed using structure-based drug discovery to provide chemical stability, enhance scalability and improve accessibility (Extended Data Fig. 1). The ACCESS phase-2b study evaluated once-daily oral aleniglipron over 36 weeks to assess the efficacy, safety and tolerability of three maintenance doses (45, 90, 120 mg daily) compared with placebo in adults with obesity or overweight and one or more weight-related comorbidity. Additionally, a predefined, open-label extension (OLE) estimated longer-term safety, durability of weight loss beyond 36 weeks and whether starting at a lower dose (2.5 mg) of aleniglipron in the placebo arm improves tolerability.

## Results

### Trial design and participants

**Fig. 1:**
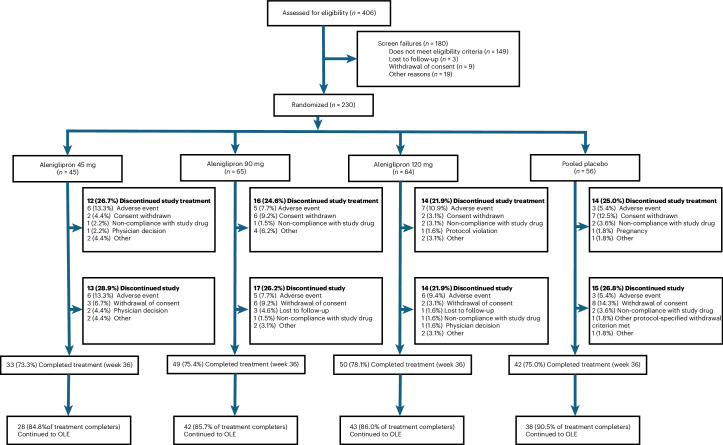
CONSORT diagram. CONSORT diagram illustrating screening and randomization to the aleniglipron treatment arms. Participants who completed treatment were allowed to enroll in the open-label extension phase of the ACCESS trial, or completed the end-of-study visit if they did not continue to open-label extension.

Between 28 October 2024 and 7 February 2025, 406 adults were screened and 230 participants underwent randomization across 38 sites in the United States (Fig. 1 and Supplementary Table 1). The mean (± s.d.) age of the randomized participants was 49.8 ± 13.7 years, body mass index (BMI) was 39.5 ± 6.9 kg m^−2^, body weight was 114.8 ± 22.6 kg and waist circumference was 121.2 ± 14.5 cm; 125 participants (54%) were female (Table 1).

**Table 1:** Participant demographics and baseline clinical characteristics at start of double-blind period

Characteristics Mean (s.d.) or *n* (%)	Aleniglipron 45 mg *n* = 45	Aleniglipron 90 mg *n* = 65	Aleniglipron 120 mg *n* = 64	Placebo *n* = 56	Overall *n* = 230
Age, years, mean (± s.d.)	49.0 (± 12.7)	47.9 (± 12.5)	52.3 (± 13.9)	49.9 (± 15.8)	49.8 (± 13.8)
Sex, female, *n* (%)	25 (55.6)	35 (53.8)	35 (54.7)	30 (53.6)	125 (54.3)
Race or ethnic group, *n* (%)					
White	37 (82.2)	53 (81.5)	56 (87.5)	46 (82.1)	192 (83.5)
Black or African American	5 (11.1)	8 (12.3)	5 (7.8)	6 (10.7)	24 (10.4)
Asian	1 (2.2)	2 (3.1)	0 (0.0)	1 (1.8)	4 (1.7)
Multiple	1 (2.2)	1 (1.5)	1 (1.6)	1 (1.8)	4 (1.7)
Not Hispanic or Latino	38 (84.4)	56 (86.2)	55 (85.9)	45 (80.4)	194 (84.3)
Hispanic or Latino	7 (15.6)	9 (13.8)	9 (14.1)	11 (19.6)	36 (15.7)
Body weight, kg, mean (± s.d.)	115.6 (± 24.7)	117.9 (± 23.6)	113.1 (± 20.5)	112.3 (± 22.0)	114.8 (± 22.6)
Body mass index, kg m^−2^, mean (± s.d.)	39.7 (± 6.9)	39.8 (± 7.5)	39.0 (± 6.4)	39.4 (± 7.0)	39.5 (± 6.9)
Waist circumference, cm, mean (± s.d.)	120.1 (± 15.3)	121.8 (± 15.0)	121.2 (± 12.5)	121.2 (± 15.6)	121.2 (± 14.5)
HbA1c, %, mean (± s.d.)	5.7 (± 0.3)	5.7 (± 0.4)	5.7 (± 0.3)	5.5 (± 0.4)	5.6 (± 0.4)
HbA1c ≥ 5.7%, *n*(%)	23 (± 51.1)	39 (± 60.0)	32 (± 50.0)	19 (± 33.9)	113 (± 49.1)
Fasting glucose level (mg dl^−1^), mean (± s.d.)	96.0 (± 13.53)	96.0 (± 11.10)	94.3 (± 10.04)	94.0 (± 10.27)	95.0 (± 11.1)
Systolic blood pressure, mmHg, mean (± s.d.)	127.0 (± 11.3)	126.4 (± 12.6)	125.3 (± 14.8)	125.3 (± 12.6)	125.9 (± 13.0)
Diastolic blood pressure, mmHg, mean (± s.d.)	83.3 (± 7.9)	81.9 (± 8.0)	80.2 (± 8.8)	80.3 (± 7.9)	81.3 (± 8.2)
Heart rate (beat min^−1^), mean (± s.d.)	72.1 (± 10.4)	72.7 (± 9.6)	69.1 (± 9.4)	71.6 (± 9.7)	71.3 (± 9.8)
eGFR (ml min^−1^ 1.73 m^−2^, mean (± s.d.)	99.0 (± 20.4)	100.2 (± 17.1)	98.8 (± 18.2)	100.5 (± 17.4)	99.6 (± 18.1)
Total cholesterol (mg dl^−1^), mean (± s.d.)	190.3 (± 39.4)	192.4 (± 37.8)	189.4 (± 38.8)	176.8 (± 32.7)	187.3 (± 37.5)
High-density lipoprotein (mg dl^−1^), mean (± s.d.)	48.5 (± 12.0)	48.3 (± 11.54)	51.5 (± 14.42)	48.7 (± 12.00)	49.3 (± 12.59)
Low-density lipoprotein (mg dl^−1^), mean (± s.d.)	116.7 (± 31.73)	117.0 (± 33.5)	111.8 (± 31.4)	105.4 (± 30.4)	112.6 (± 32.0)
Very low-density lipoprotein cholesterol (mg dl^−1^), mean (± s.d.)	25.0 (± 11.8)	27.1 (± 23.2)	26.2 (± 15.1)	22.6 (± 15.3)	25.4 (± 17.3)
Triglycerides (mg dl^−1^), mean (± s.d.)	125.0 (± 58.7)	130.1 (± 81.3)	128.9 (± 69.1)	111.1 (± 65.0)	124.1 (± 70.0)

eGFR, estimated glomerular filtration rate.

Similar percentages of participants completed the study treatment in the 45-mg (33 (73.3%)), 90-mg (49 (75.4%)) and 120-mg (50 (78.1%)) aleniglipron arms and the pooled placebo group (42 (75.0%)) (Fig. 1).

**Fig. 2:**
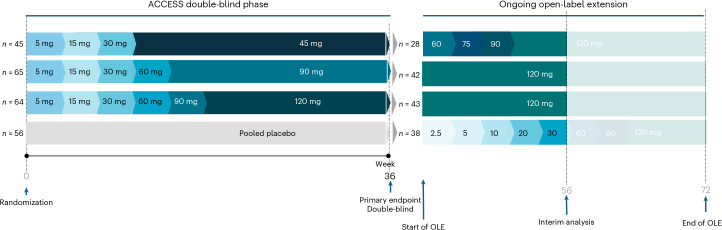
Study design. Study design of ACCESS, showing double-blind phase and number of participants who then continued into the open-label extension phase.

Participants were randomized to three dose-level cohorts (45, 90 or 120 mg) at a ratio of 3:4:4, then to aleniglipron or placebo at a ratio of 3:1 in each cohort (Figs. 1 and 2). Each participant received ascending daily doses of aleniglipron, starting at 5 mg or placebo, with titration every 4 weeks until the maintenance dose was reached over a 36-week period (Fig. 2). Randomization was stratified by baseline BMI (≤35 kg m^−2^; >35 kg m^−2^) and sex. For analyses, participants assigned to the placebo in each cohort were pooled for comparison with each aleniglipron treatment group.

The primary endpoint (efficacy estimand) was the percentage change in body weight from baseline to week 36. The secondary endpoints (efficacy estimand) were the percentages of participants who achieved ≥5%, ≥10% and ≥15% reductions in body weight by week 36, and changes in absolute body weight, waist circumference and BMI from baseline to week 36. Additional secondary safety endpoints were treatment-emergent adverse events (TEAEs), serious adverse events (SAEs) (Extended Data Table 1), adverse events of special interest (AESIs), laboratory parameters, electrocardiograms and vital signs. AESIs included major adverse cardiovascular events, heart-failure syndrome, acute pancreatitis, hypoglycemia, thyroid malignancies, C-cell hyperplasia, supraventricular arrhythmia, cardiac conductive disorders, hepatobiliary disorders, severe gastrointestinal (GI) adverse events (AEs), acute renal events, depressive disorder, suicidal ideation and hypersensitivity (Extended Data Table 1).

### Primary outcomes

**Fig. 3:**
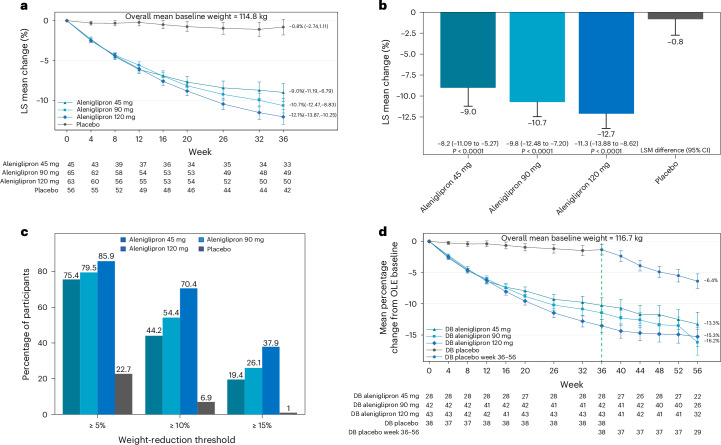
Body-weight reduction. **a**, Body-weight reduction in double-blind period (efficacy estimand). The number of participants at each assessment is denoted below the graph. **b**, Body-weight reduction and placebo-adjusted body-weight reduction at week 36 of the double-blind period (efficacy estimand). Error bars are the standard error of the LS mean change from the MMRM model. The confidence intervals and *P* values are from the MMRM model. **c**, Categorical body-weight reduction in the double-blind period. Model-estimated response rates were calculated using Rubin’s rule by combining the percentages of participants who met the target in imputed data sets analyzed with logistic regression. 5 mg arm, *n* = 33. 90 mg arm, *n* = 49. 120 mg arm, *n* = 50. Pooled placebo, *n* = 42. In **a**–**c**, the baseline is defined as the last measurement before the first dose of the study drug. Under the efficacy estimand, values more than two days after intercurrent events (permanent discontinuation of treatment) are considered invalid and set to missing. Standard error bars are displayed, with the LS mean percentage change from baseline from the MMRM model. The confidence intervals or *P* values were not adjusted for multiplicity. **d**, Body-weight reduction in the open-label extension (summary descriptives). Baseline is defined as the last measurement before the first dose of study drug in the double-blind phase. Results are based on the average of triplicate measurements of weight taken at each visit. The dotted green line indicates the beginning of open-label extension. 95% CIs were not adjusted for multiplicity and were not used for hypothesis testing. Error bars indicate the s.d. The number of participants at each assessment is shown below the graph. Week 56 data, as indicated by the sample size, reflect that not all participants have reached this time point assessment as the study is ongoing. Source data

The primary endpoint, a statistically significant reduction in body weight from baseline to week 36 compared with placebo, was met across all aleniglipron doses in the efficacy estimand. The least squares (LS) mean change in body weight from baseline to week 36 with aleniglipron was −9.0% for 45 mg (95% confidence interval (CI), −11.2 to −6.8%), −10.7% for 90 mg (−12.5 to −8.8%) and −12.1% for 120 mg (−13.9 to −10.3%) compared with placebo (−0.8%, −2.7 to 1.1%) (Fig. 3a). The placebo-adjusted LS mean (95% CI) change in body weight from baseline to week 36 for 45 mg was −8.2% (−11.1 to −5.3%; (−9.2 kg)), −9.8% (−12.5 to −7.2%; (−11.0 kg)) for 90 mg and −11.3% (−13.9 to −8.6%; (−12.4 kg)) for 120 mg aleniglipron (all *P* < 0.0001) (Fig. 3b). The treatment regimen estimand showed similar results, with a LS mean (95% CI) body-weight change from baseline of −8.8% (−11.1 to −6.6%) in the 45-mg aleniglipron arm, −10.2% (−12.1 to −8.4%) in the 90-mg arm and −11.4% (−13.3 to −9.6%) in the 120-mg arm (Extended Data Table 2).

### Secondary outcomes

#### Body-weight loss

Body-weight loss of ≥5%, ≥10% and ≥15% occurred in 86%, 70% and 38% of participants on 120 mg aleniglipron, versus 23%, 7% and 1% in the placebo arm, respectively (Fig. 3c and Extended Data Table 3).

Mean absolute body weight, mean waist circumference and mean BMI in the aleniglipron arms were all statistically significantly reduced compared with their values in the placebo arm (Extended Data Table 4).

#### Clinical parameters

There were no clinically meaningful changes in lipid profiles from baseline to week 36 (Extended Data Table 5). The overall mean baseline hemoglobin A1c (HbA1c) of 5.63% was reduced in participants on aleniglipron by −0.27%, −0.32% and −0.36% in the 45-mg, 90-mg and 120-mg arms, respectively, compared with 0.01% in placebo.

#### Safety and tolerability

**Fig. 4:**
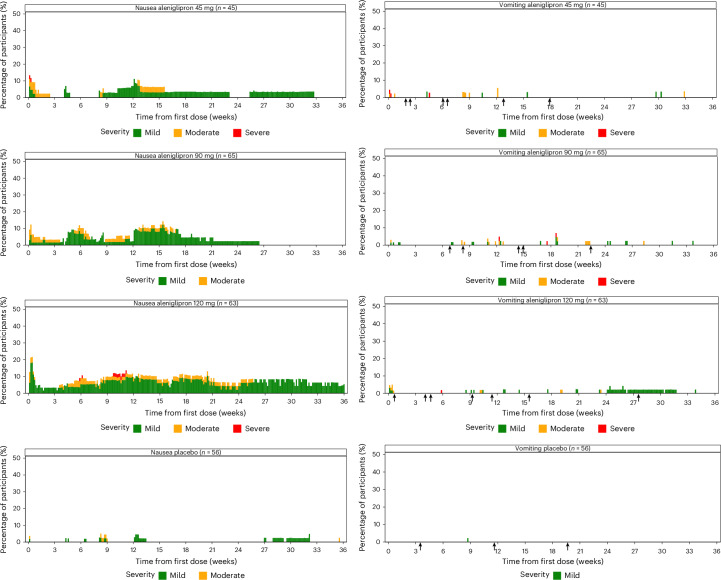
The prevalence of GI TEAEs (nausea and vomiting) and timing of study drug discontinuations due to adverse events. Arrows indicate any AE leading to study drug discontinuations. The *y* axis represents the prevalence rate, calculated as the proportion of participants who experienced nausea (left) or vomiting (right) on a daily basis among participants who took the target dose of the study drug on the day on the *x* axis. TEAEs are defined as AEs that started or worsened in severity after the first dose of study drug, and onset date is within 7 days after the last dose of study drug. Intermittent events with ‘intermittent’ or ‘occasional’ in the verbatim field in electronic data capture were not included. TEAEs are enumerated from onset to resolution or the end of the study (if not resolved), and the maximum severity is selected. Source data

**Extended Data Fig. 2:**
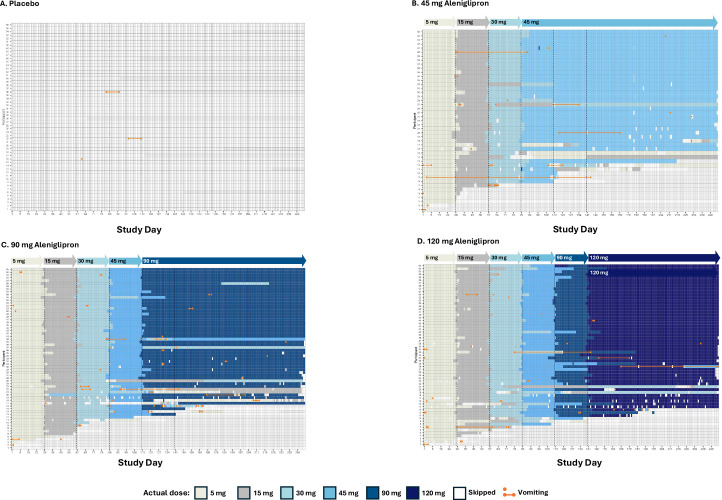
Heat map of vomiting over time and by dose arm. **A**. Placebo arm. **B**. 45 mg aleniglipron arm. **C**. 90 mg aleniglipron arm. **D**. 120 mg aleniglipron arm. Colored arrows at top of each panel indicate targeted dose titration. Colored boxes in panels indicate actual dose level: White box, skipped dose or placebo. Beige, 5- < 15 mg. Medium grey, 15- < 30 mg. Light blue, 30- < 60 mg. Medium blue, 60- < 90 mg. Dark blue, 90- < 120 mg. Navy blue ≥ 120 mg. Light grey indicates early study discontinuation. Orange lines indicate intermittent events of vomiting. Dots represent events of vomiting of 1 day duration. Vertical dashed lines indicate time of titration to next dose, every 4 weeks. Each row indicates an individual participant, represented with mock participant numbers beginning at 1 for each panel.

The most commonly occurring TEAEs with aleniglipron were GI-related, including nausea, diarrhea, vomiting and constipation (Table 2). As shown in Extended Data Figure 2 (heatmap of tolerability), most participants in the three aleniglipron arms were following the titration schedule in the protocol. Of note, participants with dose interruptions (indicated in the panels as white cells) generally did not experience vomiting upon reinitation. This tolerability profile was aligned with the number of AEs leading to treatment discontinuations (Table 2), with an overall rate of 10.4% in the aleniglipron-treated arms. Treatment discontinuations due to TEAEs were mostly related to GI AEs (Table 2), and there was no dose–response relationship across aleniglipron dose arms. The treatment discontinuations due to TEAEs predominantly occurred at earlier titration steps across all doses, and GI AEs were attenuated at later time points (Fig. 4).

**Table 2:** Common TEAEs by preferred term (safety analysis set) during the double-blind study period (baseline to week 36)

	Aleniglipron 45 mg (*n* = 45) *n* (%)	Aleniglipron 90 mg (*n* = 65) *n* (%)	Aleniglipron 120 mg (*n* = 63) *n* (%)	Pooled placebo (*n* = 56) *n* (%)
Any SAEs	1 (2.2)	0 (0.0)	4 (6.3)	3 (5.4)
Any study drug-related SAEs	0 (0.0)	0 (0.0)	1 (1.6)	1 (1.8)
Any AESIs	12 (26.7)	11 (16.9)	17 (27.0)	15 (26.8)
Any TEAEs	42 (93.3)	60 (92.3)	58 (92.1)	44 (78.6)
Any TEAE leading to discontinuation of treatment	6 (13.3)	5 (7.7)	7 (11.1)	3 (5.4)
Any GI-related TEAE leading to dose discontinuation	6 (13.3)	5 (7.7)	3 (4.8)	1 (1.8)
Any GI-related TEAEs	40 (88.9)	55 (84.6)	50 (79.4)	31 (55.4)
Common TEAEs (≥5%; preferred term)				
Nausea	32 (71.1)	44 (67.7)	41 (65.1)	12 (21.4)
Vomiting	18 (40.0)	29 (44.6)	20 (31.7)	3 (5.4)
Diarrhea	19 (42.2)	26 (40.0)	14 (22.2)	13 (23.2)
Constipation	18 (40.0)	20 (30.8)	19 (30.2)	8 (14.3)
Headache	10 (22.2)	11 (16.9)	17 (27.0)	10 (17.9)
Dyspepsia	8 (17.8)	8 (12.3)	6 (9.5)	5 (8.9)
Gastroesophageal reflux disease	7 (15.6)	8 (12.3)	10 (15.9)	0 (0.0)
Abdominal distension	4 (8.9)	11 (16.9)	6 (9.5)	3 (5.4)
Abdominal pain	4 (8.9)	11 (16.9)	7 (11.1)	2 (3.6)
Fatigue	3 (6.7)	7 (10.8)	10 (15.9)	3 (5.4)
Upper respiratory tract infection	5 (11.1)	4 (6.2)	6 (9.5)	4 (7.1)
Eructation	3 (6.7)	6 (9.2)	5 (7.9)	1 (1.8)
Dizziness	2 (4.4)	2 (3.1)	6 (9.5)	3 (5.4)

Treatment-emergent SAEs occurred in one participant (2.2%) in the 45-mg arm, none in the 90-mg arm, four participants (6.3%) in the 120-mg arm and three participants (5.4%) in the pooled placebo group (Table 2). No deaths occurred during the trial.

Participants in the aleniglipron-treated arms showed clinically meaningful reductions in both systolic and diastolic blood pressure, and there was no evidence of QTc prolongation (Extended Data Table 5). At week 36 (pre-dose), heart-rate increases of 0.8 ± 1.3, 2.2 ± 1.1 and 2.1 ± 1.1 beats per minute (b.p.m.) were observed in the 45-, 90- and 120-mg groups, respectively, compared with −1.2 ± 1.1 b.p.m. in the placebo group, representing a placebo-adjusted difference of only 2.0–3.4 b.p.m. (Extended Data Table 5). Additionally, heart-rate measurements were collected 2–4 h after the dose, with a placebo-adjusted difference ranging from 2.0 to 3.8 b.p.m. (Extended Data Table 5).

No drug-induced liver injury or persistent elevation of liver enzyme levels (between baseline and week 36) was observed across all aleniglipron treatment arms. All fluctuations in ALT or AST levels reaching three or more times the upper limit of normal (ULN) (*n* = 8) or five times the ULN (*n* = 1) returned to baseline values without stopping treatment with the study drug. There were no cases of liver enzyme elevations of ten or more times the ULN (Extended Data Table 6).

### Exploratory outcomes

#### C-reactive protein

An exploratory analysis of changes in high-sensitivity C-reactive protein (hsCRP) showed clinically meaningful reductions in the three aleniglipron arms, ranging from −28.8% to −46.4%, compared with a reduction of −6.9% in the placebo group (Extended Data Table 5).

#### Open-label extension efficacy and safety: interim analysis

A prespecified interim analysis of the OLE after a median follow-up of 20 weeks indicated further weight loss beyond 36 weeks (Fig. 3d and Supplementary Table 2), achieving a total mean weight loss from randomization to the interim analysis of 13.3 ± 8.9%, 16.2 ± 10.4% and 15.3 ± 8.7% in the 45-, 90- and 120-mg aleniglipron groups, respectively, at week 56. Percentages of GI-related TEAEs were lower than those seen in the initial phase of the study (weeks 0–36) (Extended Data Table 7).

**Extended Data Fig. 3:**
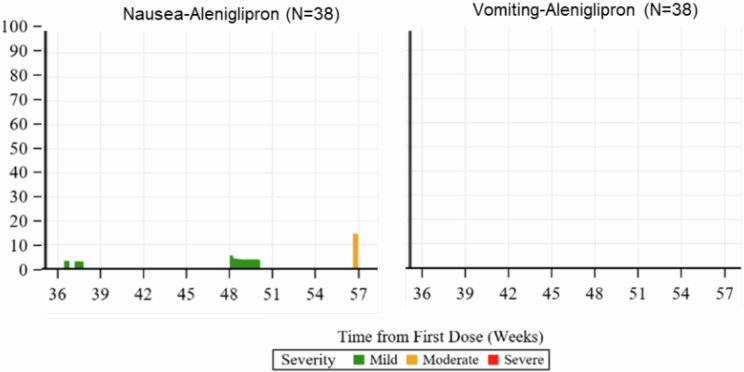
Tolerability to 2.5 mg starting dose of aleniglipron after a median follow up of 20 weeks in the OLE period (previous placebo group initiating aleniglipron at 2.5 mg daily for 4 weeks, followed by uptitration to 5 mg, 10 mg, 20 mg, 30 mg daily at 4 week intervals). Figure represents prevalence of nausea or vomiting over time. Y axis represents the prevalence rate calculated as the proportion of participants who experienced nausea or vomiting on a daily basis among participants who were dosed the study drug at target dose on the respective day. TEAEs are defined as AEs that started or worsened in severity after the first dose of OLE study drug, and onset date is within 7 days after the last dose of study drug. Intermittent events with ‘INTERMITTENT’ or ‘OCCASIONAL’ in the verbatim field in electronic data capture were not included. TEAEs are enumerated from onset to resolution or data cutoff (if ongoing) and the maximum severity is selected.

In addition, participants from the placebo arm who initiated aleniglipron at a lower dose of 2.5 mg had a body-weight reduction of 6.4% at week 56 (Fig. 3d) after a median follow-up of 20 weeks with a titrated dose of around 30 mg by that time. At the time of the interim analysis, there were no reported vomiting events, and 39.9% of participants reported nausea with no AE-related study drug discontinuations (Extended Data Table 7 and Extended Data Fig. 3).

## Discussion

The ACCESS study demonstrated that once-daily aleniglipron at doses of 45 mg, 90 mg or 120 mg led to dose-dependent, clinically meaningful reductions in body weight in adults with obesity or overweight over a 36-week period. Of note, 86% of participants randomized to the 120-mg aleniglipron arm had a body-weight reduction of at least 5%, the recommended cut-off in FDA guidance for an effective obesity medication^10^, with 70% and 38% of participants achieving weight reductions of 10% and 15%, respectively. Other cardiovascular risk factors were also notably improved with aleniglipron treatment, such as systolic blood pressure, hsCRP and HbA1c, which could positively contribute to the known cardiovascular benefits and potential diabetes-prevention effects of approved GLP-1RAs^6,8,11^.

Despite the observed and anticipated attenuation of effect beyond week 36, there were no apparent signs of a weight-loss plateau. Additionally, the ongoing OLE, a unique feature of the program, provided evidence showing additional weight reduction, with an interim analysis revealing up to 16.2% body-weight loss at week 56. This result suggests that further weight-loss benefits could be attainable with a longer treatment duration.

In the absence of head-to-head studies of GLP-1 RAs, any comparisons must be interpreted with caution because of differences in routes of administration, pharmacokinetic characteristics and specific study populations and designs^4,12,13^. However, for other small-molecule GLP-1RAs, an estimated 10.5% body-weight reduction at 36 weeks (phase 3, 72-week study) following a 4-week titration step has been reported^9^. In ACCESS, the 120-mg aleniglipron arm consisted of a participant population with a high number of males, a group in which efficacy has typically been lower^13^; however, in this group, there was still an 11.3% reduction in body weight. This result, along with the lack of apparent plateauing, positions aleniglipron as a solid candidate as an obesity treatment.

The safety and tolerability profiles of aleniglipron in this study reflect the known AEs for the GLP-1 RA class of drugs^14^. The most important variable in assessing safety and tolerability is the percentage of participants experiencing an AE leading to treatment discontinuation (10.4%). Notably, there was no increase in the number of discontinuations as participants reached higher doses of aleniglipron. Most discontinuations due to TEAEs occurred during the initial titration steps to a higher dose and could therefore be considered modifiable with a new titration strategy of starting at a lower dose with smaller increases to the next dose, as the placebo crossover data in the extension phase suggested. In the OLE study, an interim analysis of the placebo group, which began at a lower starting dose of aleniglipron (2.5 mg versus 5 mg) with smaller dose-escalation increments, demonstrated improved tolerability with no TEAE-related discontinuations and few GI events while achieving clinically relevant body-weight reductions over a 20-week median follow-up.

Notably, the proactive collection of GI AEs through an electronic diary (e-diary) provided more rigorous evaluations of AEs but introduced potential ascertainment bias and possibly higher reporting compared with non-solicited AE reporting^15^. The heat maps of dose levels overlaid with vomiting events add clarity to the interpretation of the AE profile and add valuable insights into the participant experience on aleniglipron. Upon examination of each participant’s dosing across the study, it becomes clear that, although some required dose interruptions or reductions, when the dose was reinitiated or titrated up again, vomiting rarely recurred. This suggests that participants on aleniglipron can successfully restart treatment or continue to increase dosing after an interruption. This could increase the likelihood of remaining on treatment for extended periods, which is essential for clinically meaningful and lifelong treatment of obesity.

In the current study, there was no apparent dose–response relationship for the most common GI AEs across all aleniglipron treatment arms, and treatment discontinuations due to any TEAE were limited. One hypothesis for the absence of a dose–response relationship in the tolerability profile is that the three treatment arms used the same titration schemes, with most events occurring early in the titration phase. Consistent with other programs in the GLP-1RA class of medicines, in this study, a low percentage of persistent vomiting events (below 10%) was observed once the maintenance dose was reached^6,9^. Of note, there was no persistent elevation of liver enzyme levels or drug-induced liver injury.

Study limitations included the prospective collection of GI AEs through an e-diary, which could have led to ascertainment bias and higher numbers of recorded GI-related AEs. This also impacted the placebo arm, in which participants reported a 20% incidence of nausea. The percentage of female participants was lower in this study than in other GLP-1RA obesity studies, which might have led to attenuated body-weight-loss results, given that female participants tend to lose more weight than do male participants^9,13,15–17^. The high percentage of white participants, and the fact that all the study sites were in the United States, might limit the generalizability of the results.

The results of the ACCESS trial support the upcoming phase 3 trial design, starting at a 2.5 mg dose rather than 5 mg, which is also expected to further improve tolerability during the early stages of titration.

In summary, aleniglipron, an orally administered small-molecule GLP-1 RA, demonstrated clinically meaningful weight reductions with a favorable safety and tolerability profile that does not worsen after aleniglipron reintroduction following dose interruption. The development of oral small-molecule GLP-1 RAs with lower manufacturing costs, simple storage conditions and greater manufacturing scalability, such as aleniglipron, could help expand access around the world for people with obesity or overweight.

## Methods

### Trial design and participants

Between 28 October 2024 and 7 February 2025, 406 adults were screened and 230 participants underwent randomization across 38 sites in the United States. The research protocol and statistical analysis plan (included as supplementary material) were approved by the central institutional review board Advarra. This study was carried out according to the principles of the Declaration of Helsinki. Each participant provided written informed consent. This was a dose-ranging phase 2b, randomized, placebo-controlled, parallel group, 36-week study. Eligible participants were adults aged ≥18 and <80 years, with obesity (BMI ≥ 30 kg m^−2^) or with overweight (≥27 kg m^−2^) and one or more weight-related comorbidity and stable weight (no more than 5% weight gain or loss) in the previous 3 months. Weight-related comorbidities included hypertension, dyslipidemia, obstructive sleep apnea, cardiovascular disease, heart-failure syndrome and MASH. Other key inclusion criteria included HbA1c < 6.5% at screening. Key exclusion criteria were a previously documented diagnosis of diabetes; body weight ≤80 kg at screening; and prior or planned surgical treatment for obesity. Excluded medications are listed in the protocol. All participants and study personnel were blinded to study drug assignment. Drug–drug interaction exclusion and inclusion criteria are described in the supplementary information (Supplementary Tables 3 and 4) and in the protocol (provided as supplementary information including, summary of any changes to protocol).

#### Full inclusion criteria

Participants were eligible for study inclusion only if all the following criteria applied at screening and baseline (before dosing). If abnormal values were obtained in the first instance, assessment could be repeated once (at the discretion of the investigator and sponsor) to confirm eligibility.

1. Signed informed consent before initiation of any study-related activities, capable of understanding the full nature and purpose of the trial (including possible risks and adverse effects) and willing to comply with all study procedures and adhere to the protocol schedule and restrictions.
2. Men and women, age ≥ 18 years and < 80 years, with:

- BMI ≥30 kg m^−2^ or
- BMI ≥ 27.0 kg m^−2^ and a previous or current diagnosis of one or more comorbidity:
  - Hypertension (as defined by a resting systolic blood pressure (BP) of ≥140 mmHg and a resting diastolic BP of ≥90 mmHg or receiving BP lowering medication for the treatment of hypertension)
  - Dyslipidemia as defined by a fasting triglyceride ≥200 mg dl^−1^ (2.2 mM), fasting low-density lipoprotein (LDL) cholesterol of ≥100 mg dl^−1^ (2.5 mM) or a high-density lipoprotein (HDL) cholesterol <40 mg dl^−1^ (1.0 mM), or receiving treatment with an approved lipid-lowering medication
  - Documented obstructive sleep apnea or receiving treatment for obstructive sleep apnea
  - Cardiovascular disease, including documented coronary artery atherosclerotic disease, peripheral arterial atherosclerotic disease, stroke or transient ischemic attacks
  - Documented diagnosis of MASH (confirmed by clinical, laboratory, imaging or biopsy findings)

3. Screening HbA1c < 6.5%
4. Reproductive status:

- Female participants of non-childbearing potential, defined as either surgically sterilized (having undergone hysterectomy, bilateral salpingectomy, bilateral tubal ligation or bilateral oophorectomy ≥6 weeks before screening) or postmenopausal (defined as no menses for ≥12 months, without an alternative medical cause, and a follicle-stimulating hormone level >40 IU l^−1^ at the screening visit for postmenopausal women ≤ 55 years).
- Female participants of childbearing potential must have had a negative urine pregnancy test (or negative serum pregnancy test) during screening and day 1. Additionally, they must not have been or planned to be breastfeeding. They agreed not to donate ova, to not attempt to become pregnant and, if engaging in sexual intercourse with a male partner, to use a highly effective method of contraception from the time of signing the informed consent form (ICF) until ≥30 days after the last dose of the study drug.
- Male participants agreed to not donate sperm and, if engaging in sexual intercourse with a female partner who could become pregnant, to use a highly effective method of contraception from signing the ICF until ≥90 days after the last dose of study drug.

#### Exclusion criteria

Participants were excluded from the study if any of the following criteria applied at the screening visit or before dosing:

**Diabetes-related medical conditions.**

1. Previous documented diagnosis of diabetes mellitus (except for gestational diabetes and the participant had HbA1c < 6.5%).
2. Use of metformin for any indication.
**Obesity-related medical conditions.**
3. Self-reported change in body weight >5% within 3 months before screening.
4. Body weight ≤80 kg at screening.
5. Had a prior or planned surgical treatment for obesity (excluding liposuction or abdominoplasty, if performed >1 year prior to screening).
6. Had or planned to have endoscopic and/or device-based therapy for obesity including, but not limited to, mucosal ablation, gastric artery embolization, intragastric balloon and duodenal–jejunal endoluminal liner, or had device removal within the 6 months prior to screening.
7. Had obesity induced by other endocrine disorders (such as Cushing’s syndrome, Prader–Willi syndrome or melanocortin-4-receptor deficiency).
**Other medical conditions**
8. History or presence of significant cardiovascular, pulmonary, hepatic, renal, hematological, GI, endocrine, immunologic, dermatologic or neurological disease, including any acute illness or major surgery within the past 3 months, or any clinical laboratory abnormality determined by the investigator or medically certified delegate to be clinically significant.
9. Any chronic disorder or severe disease that, in the opinion of the investigator, might have jeopardized the participant’s safety, compliance with the protocol and/or ability to complete the study.
10. History of treatment for cancer in the past 5 years (excluding surgically resected skin squamous cell or basal cell carcinoma, in situ cervical cancer or in situ prostate cancer).
11. Serum calcitonin level at screening of ≥20 ng l^−1^ if estimated glomerular filtration rate (eGFR) ≥ 60 ml^−1^ min^−1^ 1.73 m^−2^ or ≥35 ng l^−1^ if eGFR <60 ml^−1^ min^−1^ 1.73 m^−2^, or a personal or family history of medullary thyroid carcinoma or multiple endocrine neoplasia syndrome type 2.
12. Had a thyroid-stimulating hormone level outside the range of 0.4 to 6.0 mIU l^−1^ at screening.
13. History of chronic pancreatitis or idiopathic acute pancreatitis, or documented history of biliary stones. A participant with a history of acute pancreatitis caused by gallstones may have been included in the study if the participant had a cholecystectomy to resolve the problem.
14. Chronic malabsorption, regardless of etiology.
15. History of Crohn’s disease, ulcerative colitis or other inflammatory bowel disease.
16. Impaired renal function at screening defined as eGFR <30 ml^−1^ min^−1^ 1.73 m^−2^ per the Chronic Kidney Disease Epidemiology Collaboration.
17. Fasting serum triglyceride level of >500 mg dl^−1^ at screening.
18. Documented diagnosis of heart failure and at Screening New York Heart Association Class III or IV.
19. Within the year before the screening visit, any of the following: episode of acute coronary event, transient ischemic attack, stroke, peripheral arterial vascular disease leading to revascularization or other significant cardiovascular event as determined by the investigator.
20. A seated BP, after resting for 5 minutes, of >160 mm Hg systolic or >100 mm Hg diastolic. The measurement was performed in triplicate, and the average of all three measurements was used to determine eligibility.
21. A pulse rate >100 b.p.m. If elevated, the measurements may have been repeated two more times, and the average of all three measurements was used to determine eligibility.
22. Evidence of abnormality on the screening visit ECG deemed clinically significant by the investigator, history of known arrhythmia or prolonged QT interval corrected using Fridericia’s formula (QTcF) (males >450 ms; females >470 ms), based on the average of triplicate electrocardiograms, or risk factors for torsade de pointes (including a family history of long QT syndrome or sudden cardiac death). A personal or family history of long QT syndrome, family history of sudden death in a first-degree relative (parents, siblings or children) before the age of 40 years, or a personal history of unexplained syncope within the last year. Use of prescription or over-the-counter medications at screening that are known to significantly prolong the QT or QTc interval.
23. Had an ECG considered by the investigator with abnormalities that might have interfered with the interpretation of changes in ECG intervals at screening.
24. Ongoing or history of frequent intermittent or chronic tachyarrhythmia syndromes (such as atrial fibrillation, supraventricular tachycardia, and positional orthostatic tachycardia syndrome). Participants with a history of premature atrial contractions or premature ventricular contractions may have been included.
25. Liver enzyme results elevated more than twice above the ULN for AST or ALT, or more than 1.5 times the ULN for bilirubin (total, conjugated or unconjugated) (except for cases of known Gilbert’s syndrome). Testing could be repeated twice.
26. Documented diagnosis of cirrhosis.
27. Presence or had sequelae of GI, liver, kidney or other conditions known to interfere with the absorption, distribution, metabolism or excretion of drugs.
28. Any history of ileus.
29. Positive test results for an active viral infection: severe acute respiratory syndrome coronavirus 2, HIV-1 or HIV-2 with positive antibodies, hepatitis B virus with a positive hepatitis B surface antigen (HBsAg), or hepatitis C virus (HCV) with positive antibodies and viral load at the screening visit.
30. A history of significant active or unstable major depressive disorder or other severe psychiatric disorder (for example, schizophrenia, bipolar disorder or other serious mood or anxiety disorder) in the past 2 years.
31. A lifetime history of a suicide attempt or psychiatric hospitalization.
32. A Patient Health Questionnaire-9 (PHQ-9) score of 15 or more prior to randomization.
33. On the Columbia Suicide Severity Rating Scale (C-SSRS) during screening (within the last 28 days):
  A ‘yes’ answer to question 4 (active suicidal ideation with some intent to act, without specific plan) on the ‘suicidal ideation’ portion of the C-SSRS or
  A ‘yes’ answer to question 5 (active suicidal ideation with specific plan and intent) on the ‘suicidal ideation’ portion of the C-SSRS or
  A ‘yes’ answer to any of the suicide-related behaviors (actual attempt, interrupted attempt, aborted attempt, preparatory act or behavior) on the ‘suicidal behavior’ portion of the C-SSRS
  and
  the ideation or behavior occurred
**Prior or concomitant therapy.**
34. Use of medications intended to promote weight loss, within 6 months prior to screening, including, but not limited to, the following:
  Saxenda (liraglutide 3.0 mg) or other GLP-1RA
  Wegovy or Ozempic (semaglutide)
  Xenical or Alli (orlistat)
  Meridia (sibutramine)
  Acutrim (phenylpropanolamine)
  Sanorex (mazindol)
  Adipex or Lomaira (phentermine)
  Belviq (lorcaserin)
  Qsymia (phentermine–topiramate combination)
  Contrave (naltrexone when combined with bupropion)
  Zepbound or Mounjaro (tirzepatide)
  Other similar body-weight-loss medication, including over-the-counter medications (for example, Alli)
35. Had current treatment with or history of treatment with (within 3 months prior to screening) medications that may cause significant weight gain, including, but not limited to, tricyclic antidepressants, atypical antipsychotics and mood stabilizers. However, participants at a stable dose (greater than 6 months and with no expectation that the dose will change within the next year) and who are weight stable for the last 6 months on these medications may be included in the study. Examples of medications causing weight gain that must be at stable doses for at least 6 months and with no expectation of changing within the next year include:
  Imipramine
  Amitriptyline
  Mirtazapine
  Paroxetine
  Phenelzine
  Chlorpromazine
  Thioridazine
  Clozapine
  Olanzapine
  Valproic acid and its derivatives
  Lithium
  Selective serotonin reuptake inhibitors other than paroxetine are permitted. Buproprion, when not combined with naltrexone is permitted.
36. Taking daily doses of amphetamine and amphetamine-like stimulants for attention-deficit/hyperactivity disorder, which include Adderall (amphetamine–mixed amphetamine salts), Ritalin (methylphenadate), Vyvance (lisdexamfetamine) and Strattera (atomoxetine) is allowed.
37. Use of strong CYP3A4 inducers or moderate to strong CYP3A4 inhibitors, drugs that are sensitive P-gp or BCRP substrates, or drugs that are P-gp inducers or P-gp inhibitors. See Supplementary Table 3 for details. Concomitant medications that are sensitive CYP3A4 substrates may require monitoring with or without dose reductions, according to the individual respective product labeling, as summarized in Supplementary Table 3. Concomitant use of lovastatin and simvastatin is not allowed (however, use of other statins is not prohibited).
38. History of substance abuse within 12 weeks before screening.
39. Participants recovering from opiate abuse or taking methadone, buprenorphine–naloxone (Suboxone) or pentazocine (Talwin). However, participants prescribed a medically appropriate use for opiates are allowed.
40. Positive drug-abuse screen (excluding marijuana (tetrahydrocannabinol (THC)) before the first dose of study drug, unless the participant is prescribed one of these classes of drugs (narcotics) for a medically appropriate indication.
**Prior or concurrent clinical study experience.**
41. Were currently enrolled in any other clinical study or had participated in another clinical trial of an investigational drug or device within 60 days before the first study drug administration. At least 6 months before the first study drug administration, if the clinical trial included a GLP-1RA, GIP receptor agonist (RA), glucagon RA, amylin RA, peptide YY RA, neuropeptide Y RA or any combination thereof and/or any investigational product currently being used for participants with overweight, obesity or type 2 diabetes.
**Other exclusions.**
42. Pregnant, breastfeeding or intending to become pregnant.
43. Female of childbearing potential and not using an adequate contraceptive method (adequate contraceptive measures as required by local regulation or practice) for the duration of the trial and for ≥30 days following the last dose of study drug.
44. Male not using adequate contraceptive method for the duration of the trial and for ≥90 days following the last dose of study drug.
45. Consumption of grapefruit, tangelo or Seville orange (or products containing grapefruit, tangelo or Seville orange) in the 10 days before the first administration of study drug and throughout the study.
46. Demonstrated clinically significant allergic reactions (for example, food, drug, or atopic reactions, asthmatic episodes) that required intervention, (for example, emergency room visit, epinephrine administration) and which, in the opinion of the investigator, would interfere with the participant’s ability to participate in the trial.
47. Known hypersensitivity to any of the study drug ingredients.

### Procedures

Participants were randomized to three dose level cohorts (45, 90 or 120 mg) at a ratio of 3:4:4, then to aleniglipron or placebo at a ratio of 3:1 in each cohort (Fig. 3 and Extended Data Fig. 1). Each participant received ascending daily doses of aleniglipron starting at 5 mg, with titration occurring every 4 weeks to the assigned dose, or placebo over 36 weeks (Fig. 3). Randomization was stratified by baseline BMI ( ≤ 35 kg m^−2^, >35 kg m^−2^) and sex. For analyses, all participants assigned to placebo in each cohort were pooled to compare with each aleniglipron treatment group. Mitigation strategies for AEs were provided throughout the study (through dietary counselling, prescription of antiemetic or antidiarrheal medication and per investigator judgment).

Participants who experienced AEs that precluded continuation at the prescribed dose were allowed to take a lower tolerated dose and could be rechallenged with the next highest full dose in the morning after 1 or 2 days. If dose re-initiation resulted in intolerable AEs, the dose could be down-titrated, but participants needed to tolerate a daily dose of at least 30 mg to remain on the study drug.

An e-diary specifically designed to inquire about the presence of GI symptomatology (for example, nausea, vomiting, constipation, diarrhea) and their intensity (using a visual analog scale), was proactively used to record and transmit data on AEs and participant-reported outcomes, daily for 7 days, after each dose titration step (every 4 weeks), as well as recording daily medication taken (including date and time of dosing).

The electronic data-capture system MediData Rave was used for data collection.

### Study objectives and endpoints

The primary endpoint (efficacy estimand) was the percentage change in body weight from baseline to week 36. The secondary endpoints were percentages of participants who achieved ≥5%, ≥10% and ≥15% reductions in body weight at week 36, and changes in body weight (absolute), waist circumference and BMI from baseline to week 36. Additional secondary safety endpoints were TEAEs, SAEs, AESIs, laboratory parameters, electrocardiograms and vital signs.

Exploratory objectives were change in lipid profile, hsCRP, resting blood pressure, fasting glucose and HbA1c.

### Open-label extension

ACCESS Participants who completed the double-blind treatment period on study drug (or placebo) and signed informed consent (*n* = 151) were eligible to enroll in the ACCESS OLE to assess the longer-term safety of aleniglipron, durability and maintenance of weight loss for an additional planned 36 weeks (from weeks 36 to 72). Participants in the active study arms continued treatment with aleniglipron titrated every 4 weeks until a dose of 120 mg was reached. Participants in the placebo group were initiated on multiple-ascending daily doses of aleniglipron, starting at a lower dose (2.5 mg versus 5 mg in the ACCESS trial), and the dose was titrated every 4 weeks up to 120 mg (Fig. 3). The results of a pre-specified interim analysis performed after a median follow-up of 20 weeks are reported in this manuscript.

### Statistical methods

We estimated that 230 participants would provide >90% power to detect a treatment difference of −5% body-weight change between the aleniglipron treatment group and placebo from baseline to week 36, assuming an s.d. of 6%, a two-sided alpha level of 0.05, 35 completers in one aleniglipron treatment group and 35 completers in the placebo group. No multiplicity adjustments were made, and CIs were not used for hypothesis testing.

The full analysis set included all randomized participants who received one or more doses of aleniglipron or placebo and had one or more post-dose assessments. The safety-analysis set included all participants who received one or more doses of aleniglipron or placebo. Safety and tolerability data are presented for all participants in the safety analysis set.

The efficacy estimand, predefined in the statistical analysis plan, estimates the treatment effect of aleniglipron relative to placebo at week 36 in participants who met the inclusion criteria and would have completed the treatment period. Data collected before the occurrence of any intercurrent events (ICEs) (+2 days) were used for the analysis. ICEs (hypothetical strategy) were defined as permanent discontinuation of study drug, initiation of another anti-obesity medication or bariatric surgery; data subsequent to the ICE were handled by the hypothetical strategy (regarded as invalid and set to missing). Missing data were imputed implicitly.

The primary endpoint based on the efficacy estimand was analyzed using a mixed model for repeated measures (MMRM) with treatment group, randomization strata (baseline BMI and sex), visit, treatment-by-visit interaction and baseline measurement as covariates. An unstructured covariance model was used.

The treatment regimen estimand, predefined in the statistical analysis plan, was to estimate the treatment effect of aleniglipron relative to placebo at week 36 in participants who met the inclusion criteria, regardless of adherence to treatment. The ICE definition was the same as that in the efficacy estimand, except data following an ICE were handled by the treatment strategy, and all subsequent data were included in the analysis. Missing data were imputed based on observed data, assuming that the benefit accrued up to the ICE is retained but there is no additional residual benefit after the ICE.

The continuous endpoints based on the two estimands were analyzed using an MMRM similar to the primary endpoint analysis above. For the body-weight categorical endpoints, logistic regression was used to analyze the data. Missing values at week 36 were imputed according to the multiple-imputation approach, and values were combined using Rubin’s rule^18^.

All tests of treatment effects were conducted at a two-sided alpha level of 0.05, and two-sided 95% CIs were calculated. Lipid parameters, fasting glucose and hsCRP were log-transformed before fitting the MMRM, as described in the SAP.

Because of the exploratory nature of the OLE study, no estimand or model-based approach was planned. The change from the double-blind baseline in body weight was summarized on the basis of observed data. Summary descriptives (number, mean, median, s.d., minimum and maximum) were presented by visit and treatment group separately.

All data processing, summarization and analyses were performed using the SAS Enterprise Guide Version 8.2 (or higher) statistical software package.

### Reporting summary

Further information on research design is available in the Nature Portfolio Reporting Summary linked to this article.

## Online content

Any methods, additional references, Nature Portfolio reporting summaries, source data, extended data, supplementary information, acknowledgements, peer review information; details of author contributions and competing interests; and statements of data and code availability are available at 10.1038/s41591-026-04476-6.

## Supplementary information

Supplementary Information Drug–drug interaction exclusion and inclusion criteria; Supplementary Tables 1–4; ACCESS trial protocol, amendment, summary of changes; ACCESS trial statistical analysis plan.

Reporting Summary

## Source data

Source Data Figs. 3 and 4.

## Data Availability

Deidentified individual patient data for variables necessary to address a specific research question may be shared with an approved data-sharing request. Researchers will submit a request containing the research objectives, endpoints or outcomes of interest, statistical analysis plan, data requirements, publication plan and their qualifications. Gasherbrum Bio does not grant external requests for individual dedentified patient data for re-evaluating safety and efficacy endpoints already addressed in the product labeling or assessing safety or efficacy for an indication in current development. Requests are reviewed by a committee of internal advisors, and if not approved, might be further arbitrated by a panel of external advisors. Upon approval, information necessary to address the research question will be provided under the terms of a data-sharing agreement. Data-sharing requests will be considered beginning 36 months after the study publication (manuscript accepted for publication) and either (1) the product has been granted marketing authorization in at least two regulatory jurisdictions, or (2) clinical development for the product and/or indication discontinues and the data will not be submitted to regulatory authorities. There is no end date for eligibility to submit a data-sharing request for this study. Data can be requested from info@structuretx.com. Requests will be acknowledged and reviewed within 60 days of receipt of request. Source data are provided with this paper.

## Extended data

**Extended Data Table 1:** SAEs and AESIs in the double-blind period of the ACCESS study

Preferred Term n (%)	Aleniglipron 45 mg (N=45)	Aleniglipron 90 mg (N=65)	Aleniglipron 120 mg (N=63)	Placebo (N=56)
**SAEs in the double-blind period of the ACCESS study**				
Any treatment-emergent SAE	1(2.2)	0	4(6.3)	3(5.4)
Appendicitis	0	0	1(1.6)	0
Cellulitis	0	0	1(1.6)	0
Parotitis	0	0	1(1.6)	0
Sepsis	0	0	1(1.6)	0
Bronchitis	0	0	0	1(1.8)
Metabolic encephalopathy	0	0	1(1.6)	0
Trigeminal neuralgia	1(2.2)	0	0	0
Acute kidney injury	0	0	2(3.2)	0
Cervical vertebral fracture	0	0	1(1.6)	0
Atrial fibrillation	0	0	0	1(1.8)
Cholecystitis	0	0	0	1(1.8)
Cholelithiasis	0	0	0	1(1.8)
**AESIs in the double-blind period of the ACCESS study**				
Any treatment-emergent AESI	12(26.7)	11(16.9)	17(27.0)	15(26.8)
Cough	2(4.4)	0	1(1.6)	4(7.1)
Dyspnoea	1(2.2)	0	0	0
Rhinitis allergic	1(2.2)	0	0	0
Wheezing	0	0	0	2(3.6)
Pruritus	0	1(1.5)	1(1.6)	0
Rash	1(2.2)	1(1.5)	0	0
Dermatitis contact	0	0	1(1.6)	0
Eczema	0	0	1(1.6)	0
Seasonal allergy	0	1(1.5)	0	1(1.8)
Hypotension	0	0	1(1.6)	0
Alanine aminotransferase increased	1(2.2)	2(3.1)	1(1.6)	1(1.8)
Blood bilirubin increased	0	0	1(1.6)	0
Blood bilirubin unconjugated increased	0	0	1(1.6)	0
Blood fibrinogen decreased	0	1(1.5)	0	2(3.6)
Aspartate aminotransferase increased	0	0	0	1(1.8)
International normalized ratio increased	0	0	0	1(1.8)
Prothrombin time prolonged	0	0	0	1(1.8)
Hepatobiliary disorders	0	1(1.5)	1(1.6)	1(1.8)
Hypertransaminasemia	0	1(1.5)	0	0
Metabolic dysfunction-associated liver disease	0	0	1(1.6)	0
Cholecystitis	0	0	0	1(1.8)
Cholelithiasis	0	0	0	1(1.8)
Severe gastrointestinal adverse events	3(6.7)	3(4.6)	1(1.6)	0
Vomiting	3(6.7)	2(3.1)	1(1.6)	0
Nausea	2(4.4)	1(1.5)	1(1.6)	0
Dyspepsia	0	1(1.5)	1(1.6)	0
Palpitations	0	1(1.5)	2(3.2)	1(1.8)
Atrial fibrillation	0	0	0	1(1.8)
Sinus tachycardia	0	0	0	1(1.8)
Syncope	0	0	3(4.8)	0
Electrocardiogram abnormal	0	1(1.5)	0	0
Acute kidney injury	0	0	3(4.8)	0
Renal impairment	1(2.2)	0	0	0
Hypoglycemia	2(4.4)	0	2(3.2)	0
Depression	0	1(1.5)	0	1(1.8)
Depressive symptom	0	0	1(1.6)	0
Suicidal ideation	0	0	1(1.6)	0
Cholecystitis	0	0	0	1(1.8)
Heart Failure Syndrome	0	0	0	1(1.8)
Bronchitis	0	0	0	1(1.8)

SAE = serious adverse event. AESI = adverse event of special interest.

**Extended Data Table 2:** Body weight reduction from baseline at Week 36 (treatment estimand)

	Aleniglipron 45 mg (N=45)	Aleniglipron 90 mg (N=65)	Aleniglipron 120 mg (N=63)	Pooled placebo (N=56)
Percent change in body weight*				
LSM^a^ (SE), (95% CI)	−8.8 (1.13) (−11.08, −6.60)	−10.2 (0.94) (−12.07, −8.37)	−11.4 (0.92) (−13.26, −9.62)	−1.0 (0.99) (−2.93, 0.96)
LSM difference^b^ (SE), (95% CI)	−7.9 (1.50) (−10.81, −4.90)	−9.2 (1.36) (−11.91, −6.55)	−10.5 (1.35) (−13.11, −7.80)	
Category of weight reduction, MBE % of participants (95% CI)**				
≥ 5%	70.1 (55.19, 81.69)	77.6 (65.82, 86.22)	77.0 (64.93, 85.79)	18.3 (10.18, 30.55)
≥ 10%	35.1 (22.64, 50.07)	45.6 (33.80, 57.84)	61.2 (48.59, 72.47)	4.3 (1.24, 13.83)
≥ 15%	13.5 (6.29, 26.75)	21.5 (13.09, 33.33)	30.8 (20.57, 43.40)	0.8 (0.05, 11.72)
≥ 20%	5.6 (1.66, 17.36)	8.6 (3.80, 18.34)	15.0 (8.09, 26.12)	0.9 (0.06, 12.31)

LSM = least squares mean; SE = standard error; CI = confidence interval; MBE = model-based estimate. *LSM, SE, and 95% CI are results from a mixed model for repeated measures (MMRM) with treatment, sex, baseline body mass index stratum, visit week, treatment-by-visit week interaction as fixed factors, and baseline body weight as covariate. An unstructured covariance structure was used. **Model estimated response rate is the average predicted probability from a logistic regression model. Missing values were imputed by categorizing the imputed values of the missing body weight at Week 36 through predicted values obtained from the MMRM analysis of percent change in body weight. 95% CI were not adjusted for multiplicity and were not used for hypothesis testing. ^a^LSM is change from baseline. ^b^LSM difference is change from baseline and placebo-adjusted.

**Extended Data Table 3:** Categorical body weight reduction (efficacy estimand)

	Aleniglipron 45 mg (N=45)	Aleniglipron 90 mg (N=65)	Aleniglipron 120 mg (N=63)	Pooled-Placebo (N=56)
% of participants with ≥5% reduction in body weight				
Observed % (n/N’)	75.8 (25/33)	79.6 (39/49)	86.0 (43/50)	19.0 (8/42)
Model Estimated (95% CI)^a^	75.4 (58.31, 87.02)	79.5 (66.16, 88.49)	85.9 (73.57, 92.98)	22.7 (12.20, 38.26)
% of participants with ≥10% reduction in body weight				
Observed % (n/N’)	42.4 (14/33)	55.1 (27/49)	72.0 (36/50)	4.8 (2/42)
Model Estimated (95% CI)^a^	44.2 (28.45, 61.24)	54.4 (40.41, 67.66)	70.4 (56.21, 81.50)	6.9 (2.09, 20.62)
% of participants with ≥15% reduction in body weight				
Observed % (n/N’)	18.2 (6/33)	26.5 (13/49)	40.0 (20/50)	0 (0/42)
Model Estimated (95% CI)^a^	19.4 (9.28, 36.23)	26.1 (15.74, 40.01)	37.9 (25.38, 52.29)	1.0 (0.07, 14.15)

CI = confidence interval. ^a^Model estimated response rates were calculated with Rubin’s rule by combining the percentages of participants who met the target in imputed data sets analyzed with logistic regression. 95% CI were not adjusted for multiplicity and were not used for hypothesis testing.

**Extended Data Table 4:** Secondary weight-loss endpoints (efficacy estimand)

Change from baseline at Week 36, LSM (SE), (95% CI)	Aleniglipron 45 mg (N=45)	Aleniglipron 90 mg (N=65)	Aleniglipron 120 mg (N=63)	Pooled placebo (N=56)
Waist circumference, cm	-9.7 (1.39) (-12.45, -6.98)	-9.5 (1.15) (-11.78, -7.23)	-12.1 (1.14) (-14.39, -9.88)	-4.1 (1.23) (-6.53, -1.69)
Placebo-adjusted LSM (SE)	-5.6 (1.81)	-5.4 (1.63)	-8.0 (1.63)	n/a
Absolute body weight, kg	-10.4 (1.29) (-12.95, -7.86)	-12.2 (1.07) (-14.35, -10.13)	-13.6 (1.06) (-15.73, -11.54)	-1.2 (1.13) (-3.42, 1.04)
Placebo-adjusted LSM (SE)	-9.2 (1.71)	-11.0 (1.55)	-12.4 (1.54)	n/a
BMI, kg m^−^^2^	-3.6 (0.45) (-4.48, -2.71)	-4.1 (0.37) (-4.86, -3.39)	-4.8 (0.37) (-5.53, -4.07)	-0.4 (0.39) (-1.15, 0.40)
Placebo-adjusted LSM (SE)	-3.2 (0.59)	-3.8 (0.54)	-4.4 (0.54)	n/a

BMI = body mass index. Least squares means (LSM) and their associated standard error (SE), 95% confidence interval (CI) are results from a mixed model for repeated measures. Unstructured covariance structure was used. 95% CI were not adjusted for multiplicity and were not used for hypothesis testing.

**Extended Data Table 5:** Changes in laboratory parameters, ECG, vital signs from baseline at Week 36 (efficacy estimand)

Change from baseline at Week 36, LSM (SE), (95% CI)	Aleniglipron 45 mg (N=45)	Aleniglipron 90 mg (N=65)	Aleniglipron 120 mg (N=63)	Pooled placebo (N=56)
Systolic blood pressure (mmHg)	-7.9 (1.71) (-11.25, -4.48)	-8.9 (1.42) (-11.68, -6.07)	-9.0 (1.40) (-11.74, -6.21)	-1.5 (1.52) (-4.49, 1.49)
Diastolic blood pressure (mmHg)	-3.3 (1.11) (-5.53, -1.14)	-4.1 (0.92) (-5.90, -2.26)	-3.5 (0.91) (-5.33, -1.73)	-0.3 (0.99) (-2.23, 1.66)
QTcF Interval, Aggregate (ms)^a^				
Pre-dose	-7.4 (2.03) (-11.41, -3.41)	-2.5 (1.68) (-5.79, 0.83)	-6.1 (1.68) (-9.44, -2.80)	-0.5 (1.78) (-3.96, 3.04)
2-4 h Post-dose	-6.9 (1.99) (-10.78, -2.96)	-5.8 (1.67) (-9.12, -2.57)	-6.3 (1.67) (-9.56, -3.01)	-5.0 (1.78) (-8.51, -1.51)
ECG Heart Rate (beats/min)^a^				
Pre-dose	0.8 (1.27) (-1.69, 3.34)	2.2 (1.06) (0.13, 4.30)	2.1 (1.06) (-0.03, 4.15)	-1.2 (1.12) (-3.44, 0.99)
2-4 h Post-dose	8.7 (1.44) (5.87, 11.54)	9.4 (1.21) (7.07, 11.82)	10.5 (1.21) (8.13, 12.89)	6.7 (1.30) (4.18, 9.27)
Free Fatty Acid (mEq/L)	-0.07 (0.036) (-0.146, -0.003)	0.00 (0.029) (-0.057, 0.059)	-0.03 (0.029) (-0.089, 0.026)	-0.04 (0.031) (-0.101, 0.021)
Hemoglobin A1C (%)	-0.27 (0.047) (-0.363, -0.178)	-0.32 (0.039) (-0.395, -0.241)	-0.36 (0.039) (-0.434, -0.280)	0.01 (0.042) (-0.070, 0.098)
**Percent change from baseline at Week 36, MBE (SE), (95% CI)**	**Aleniglipron 45 mg (N=45)**	**Aleniglipron 90 mg (N=65)**	**Aleniglipron 120 mg (N=63)**	**Pooled placebo (N=56)**
Cholesterol	-3.9 (2.2) (-8.2, 0.6)	-8.3 (1.8) (-11.8, -4.8)	-7.5 (1.8) (-10.9, -4.0)	-2.5 (2.0) (-6.4, 1.5)
HDL Cholesterol	2.2 (2.5) (-2.6, 7.3)	-1.6 (2.0) (-5.4, 2.5)	-0.2 (2.0) (-4.1, 3.9)	0.9 (2.2) (-3.4, 5.3)
Triglycerides	-13.5 (4.5) (-21.9, -4.1)	-9.3 (3.9) (-16.7, -1.3)	-12.3 (3.7) (-19.4, -4.6)	-5.9 (4.3) (-14.1, 3.1)
LDL Cholesterol	-4.2 (3.1) (-10.1, 2.0)	-8.2 (2.4) (-12.9, -3.2)	-9.0 (2.4) (-13.6, -4.1)	-0.9 (2.8) (-6.3, 4.8)
Fasting Glucose	-6.3 (1.5) (-9.2, -3.2)	-5.6 (1.3) (-8.0, -3.0)	-6.6 (1.2) (-9.0, -4.1)	0.9 (1.4) (-1.9, 3.7)
hsCRP	-33.4 (10.1) (-50.6, -10.0)	-28.8 (8.6) (-43.9, -9.8)	-46.4 (6.6) (-58.0, -31.8)	-6.9 (12.1) (-28.0, 20.4)

QTcF = QT Corrected for Heart Rate using Fridericia’s Formula; ECG = electrocardiogram; MBE = model-based estimate; HDL = high-density lipoprotein; LDL = low-density lipoprotein. Least squares means (LSM) and their associated standard error (SE), 95% confidence interval (CI) are results from a mixed model for repeated measures based on the efficacy estimand. 95% CI were not adjusted for multiplicity and were not used for hypothesis testing.

^a^Change in pre-dose and post-dose parameters at Week 36 are made in comparison to baseline values before any dose of study drug.

**Extended Data Table 6:** Summary of participants with maximum post-baseline ALT or AST ≥ 3×, ≥ 5×, and ≥ 10× upper limit of normal

Liver enzymes	Aleniglipron 45 mg (N=45) n (%)	Aleniglipron 90 mg (N=65) n (%)	Aleniglipron 120 mg (N=63) n (%)	Pooled placebo (N=56) n (%)
≥ 3x ULN (either ALT/AST)	1 (2.3)	3 (4.8)	2 (3.2)	2 (3.6)
ALT	1 (2.3)	3 (4.8)	2 (3.2)	1 (1.8)
AST	0 (0)	0 (0)	0 (0)	1 (1.8)
≥ 5x ULN (either ALT/AST)	0 (0)	1 (1.6)	0 (0)	0 (0)
ALT	0 (0)	1 (1.6)	0 (0)	0 (0)
AST	0 (0)	0 (0)	0 (0)	0 (0)
≥ 10x ULN (either ALT/AST)	0 (0)	0 (0)	0 (0)	0 (0)
ALT	0 (0)	0 (0)	0 (0)	0 (0)
AST	0 (0)	0 (0)	0 (0)	0 (0)

ALT = alanine aminotransferase; AST = aspartate aminotransferase; ULN = upper limit normal. % = n/N’ where N’ is the number of participants with a lab measurement at baseline or post-baseline for the given parameter. Baseline is defined as the last measurement prior to the first dose of study drug.

**Extended Data Table 7:** Common TEAEs by preferred term (safety analysis set) during the OLE study period

Aleniglipron starting dose in OLE period	Aleniglipron 60 mg (N=28) n (%)	Aleniglipron 120 mg (N=42) n (%)	Aleniglipron 120 mg (N=43) n (%)	Aleniglipron 2.5 mg (N=38) n (%)
Arm in the double-blind treatment period	45 mg	90 mg	120 mg	Placebo
Any TEAE	16 (57.1)	27 (64.3)	23 (53.5)	31 (81.6)
Any TEAE leading to discontinuation of treatment	0	1 (2.4)	0	0
Common TEAEs (≥5%; preferred term)				
Nausea	5 (17.9)	5 (11.9)	5 (11.6)	15 (39.9)
Vomiting	2 (7.1)	5 (11.9)	7 (16.3)	0
Diarrhea	2 (7.1)	7 (16.7)	3 (7)	10 (26.3)
Constipation	3 (10.7)	6 (14.3)	1 (2.3)	12 (31.6)

TEAE = treatment-emergent adverse event.
