# Publisher Correction: Oral small molecule GLP-1 receptor agonist aleniglipron in people with overweight or obesity: a randomized, double-blind, placebo-controlled phase 2b trial

**DOI:** 10.1038/s41591-026-04532-1

**Published:** 2026-06-24

**Authors:** Julio Rosenstock, Ildiko Lingvay, Donna Ryan, Ania M. Jastreboff, Robert Kushner, Andres Acosta, Thomas C. Blevins, Melissa Choi, Faith L. Holmes, Timothy Smith, Lisa Connery, Yao Li, Mike Ke Liu, Aline Barth, Jamie Butcher, Antonio Civitarese, Huibin Yue, Blai Coll

**Affiliations:** 1 The University of Texas Southwestern Medical Center, Dallas, TX USA; 2 University of Texas Southwestern Medical Center, Internal Medicine, Dallas, TX USA; 3 Pennington Biomedical Research Foundation, Baton Rouge, LA USA; 4 Yale School of Medicine, Internal Medicine, Endocrinology, New Haven, CT USA; 5 Northwestern University Feinberg School of Medicine, Chicago, IL USA; 6 Mayo Clinic, Medicine, Rochester, MN USA; 7 Texas Diabetes and Endocrinology, Austin, TX USA; 8 Trialmed, Minneapolis, MN USA; 9 Elligo Health Research, Austin, TX USA; 10 StudyMetrix Research, St. Peters, MO USA; 11 Alliance for Multispecialty Research, Knoxville, TN USA; 12 Structure Therapeutics, South San Francisco, CA USA

**Keywords:** Drug development, Obesity

Correction to: *Nature Medicine* 10.1038/s41591-026-04476-6, published online 5 June 2026.

In the version of the article initially published, in Fig. 3b, the Aleniglipron 120 mg column initially listed LS mean change as “–12.1” and has been updated to read “–12.7,” while in Fig. 4, the data was missing from the bottom-right panel, and all arrows were missing from the “Vomiting” panels. Figures 3 and 4 have now been corrected in the HTML and PDF versions of the article.

