# Supplementary material for: Oral small molecule GLP-1 receptor agonist aleniglipron in people with overweight or obesity: a randomized, double-blind, placebo-controlled phase 2b trial
Source: Nat Med. 2026 Jun 5;32(8):2941–7. doi: 10.1038/s41591-026-04476-6 (PMC13472849; doi:10.1038/s41591-026-04476-6)
Supplement: Supplementary file 1 [file 41591_2026_4476_MOESM1_ESM.pdf]

nature medicine

Supplementary information https://doi.org/10.1038/s41591-026-04476-6

Oral small molecule GLP-1 receptor agonist aleniglipron in people with overweight or obesity: a randomized, double-blind, placebo-controlled phase 2b trial

In the format provided by the authors and unedited

Supplementary information

Drug-Drug Interaction Exclusion/Inclusion Criteria

Supplementary Table 1. Principal investigators and study sites

Supplementary Table 2. CYP3A4 inhibition: recommendation from the product label of concomitant medications that are sensitive CYP3A4 substrates

Supplementary Table 3. Excluded medications

Supplementary Table 4. Demographics at Week 36 of ACCESS trial (baseline of open-label extension)

ACCESS Trial Protocol, Amendment, and Summary of Changes

ACCESS Trial Statistical Analysis Plan

Drug-Drug Interaction Exclusion/Inclusion Criteria

Aleniglipron is a P-glycoprotein (P-gp) and cytochrome p-450 3A4 (CYP3A4) substrate based on *in vitro* data. Therefore, strong and moderate CYP3A4 inhibitors, as well as strong CYP3A4 inducers were excluded from the study. In addition, Pg-P inducers and inhibitors were excluded from the study.

Aleniglipron is a breast cancer resistance protein (BCRP), P-gp, and CYP3A4 inhibitor, based on *in vitro* data. Therefore, this study excluded participants who were taking concomitant medications that are sensitive P-gp substrates (e.g., dabigatran etexilate, digoxin, fexofenadine, everolimus, sirolimus, or venetoclax) or sensitive BCRP substrates (e.g., ozanimod, sulfasalazine). Because aleniglipron is only a weak CYP3A4 inhibitor, concomitant medications that are CYP3A4 substrates did not need to be excluded from this study; however, concomitant medications that are sensitive CYP3A4 substrates may require monitoring with or without dose reductions, according to the individual respective product labeling as summarized in Supplementary Table 2. As lovastatin and simvastatin are sensitive CYP3A4 substrates, concomitant use of these medications was not allowed. Participants

taking lovastatin or simvastatin should be switched to rosuvastatin at least 2 days prior to randomization. Participants on other statin drugs, including atorvastatin or rosuvastatin, were allowed to be enrolled. Please refer to Supplementary Table 3 below for a list of excluded medications and herbal products.

Supplementary Table 1. Principal investigators and study sites

Country	Site No.	Address	Investigator Name
USA	1001	6500 North Mopac Expwy, Building 3; Suite 200; Austin, Texas 78731	Thomas Blevins
USA	1002	3121 Signature Court; Medford, Oregon 97504	Sarah Smiley
USA	1003	2500 Ridge Suite 109; Evanston, Illinois 60201	Jeffrey Geohas
USA	1004	Neurosciences Institute 47 New Scotland Avenue MC70; Albany, New York 12208	Robert Busch
USA	1005	160 East 32nd Street., 3rd Floor; New York, New York 10016	Holly Lofton
USA	1006	5758 South Maryland Avenue; Chicago, Illinois 60637	Silvana Pannain
USA	1007	6400 Perkins Road; Baton Rouge, Louisiana 70808	Steven Heymsfield
USA	1008	10225 Austin Dr. #203, Suite 203; Spring Valley, California 91978	Hanid Audish
USA	1009	1202 Medical Center Drive; Wilmington, North Carolina 28401	Kevin Cannon
USA	1011	1016 24th Avenue NW, Suite 100; Norman, Oklahoma 73069	Lisa Connery

USA	1012	4201 Corporate Drive Suite 100; West Des Moines, Iowa 50265	Robert Burr
USA	1013	525 Oak Centre Drive, Suite 340; San Antonio, Texas 78258	Daniel Katselnik
USA	1014	500 Helendale Rd, Suite 200; Rochester, New York 14609	Matthew Davis
USA	1015	33 North Dearborn, Suite 500; Chicago, Illinois 60602	Joseph Newberg
USA	1016	7974 Haven Avenue, Suite 268; Rancho Cucamonga, California 91730	Steven Barag
USA	1017	7777 Forest Ln, Suite C-685; Dallas, Texas 75230	Dan Lender
USA	1018	4350 S. National Ave., Suite C100; Springfield, Missouri 65810	Ralph Duda Jr
USA	1020	7600 North 15th Street, Suite 185; Phoenix, Arizona 85020	Elysa Shaw
USA	1021	11612 Bee Cave Rd, Building 1 Suite 150; Austin, Texas 78738	Faith Holmes
USA	1022	2230 Auburn Avenue, Level B; Cincinnati, Ohio 45219	Matthew Wenker
USA	1023	1585 Kapiolani Blvd, Suite 1500; Honolulu, Hawaii 96814-4526	David Fitz-Patrick
USA	1024	1130 Upper Front Street; Binghamton, New York 13905	Frank Eder
USA	1026	3840 Watt Avenue; Sacramento, California 95821	Douglas Young
USA	1027	621 North Hall Street, Suite 440; Dallas, Texas 75226	Cara East
USA	1028	1621 N. Belt Line Road; Mesquite, Nevada 75149	Sumana Gangi
USA	1029	637 Dunn Runn Road, Suite 135; hazelwood, Missouri	Larry Reed

		63042	
USA	1030	140 Curry Hollow Road, Suite 3; Pittsburgh, Pennsylvania 15236	Bryce Palchick
USA	1031	3590 West 9000 South, Suite 300; West Jordan, Utah 84088	Barbara Rizzardi
USA	1032	6035 Shallowford Road, Suite 109; Chattanooga, Tennessee 37421-3124	Natasha Ballard
USA	1033	2441 W. La Palma Avenue, Suite 140; Anaheim, California 92801	Amina Haggag
USA	1034	104 Selma Drive; Winchester, Virginia 22601-3834	Philip O'Donnell
USA	1036	3862 Mexico Road, St. Peters; Saint Peters, Missouri 63303	Timothy Smith
USA	1037	2061 Highway 52; Moncks Corner, South Carolina 29461	Jeremy Ackermann
USA	1038	1127 Wilshire Blvd., Suite 805; Los Angeles, California 90017	Joseph Lamb
USA	1039	9525 Monte Vista Ave, Suite 200; Montclair, California 91763	Rizwana Mohseni
USA	1040	55 University Drive, Suite 106; Valparaiso, Indiana 46383	Robert Buynak
USA	1041	6601 Lyndale Ave South Richfield, Suite 210; Minneapolis, Minnesota 55423	Melissa Choi
USA	1042	3631 N Morrison Road; Muncie, Indiana 47304	Jonathan Condit

Supplementary Table 2. Demographics at Week 36 of ACCESS trial (baseline of open-label extension)

Characteristic	DB Aleniglipron 45 mg N=28	DB Aleniglipron 90 mg N=42	DB Aleniglipron 120 mg N=43	DB Placebo N=38	Overall N=151
**Age, years, mean (±SD)**	50.8(±12.07)	49.0(±11.57)	53.4(±13.17)	52.1(±14.82)	51.4(±12.98)
**Sex, female, n (%)**	15(53.6)	23(54.8)	26(60.5)	18(47.4)	82(54.3)
**Race or ethnic group, n (%)**					
**White**	23(82.1)	35(83.3)	37(86.0)	31(81.6)	126(83.4)
**Black or African American**	4(14.3)	7(16.7)	4(9.3)	4(10.5)	19(12.6)
**Asian**	0	0	0	0	0
**Multiple**	1(3.6)	0	1(2.3)	1(2.6)	3(2.0)
**Not Hispanic or Latino**	23(82.1)	35(83.3)	37(86.0)	30(78.9)	125(82.8)
**Hispanic or Latino**	5(17.9)	7(16.7)	6(14.0)	8(21.1)	26(17.2)
**Body weight, kg, mean (±SD)**	109.5(±26.60)	107.8(±24.42)	97.0(±19.87)	111.2(±22.32)	105.9(±23.60)
**Body mass index, kg/m^2, mean (±SD)**	36.9(±7.53)	36.1(±6.64)	33.9(±6.29)	39.0(±7.67)	36.3(±7.17)
**Fasting glucose level (mg/dL), mean (±SD)**	88.1(10.74)	88.7(10.64)	88.8(8.94)	96.2(12.37)	90.5(11.09)
**HbA1C level, %, mean (±SD)**	5.36(0.263)	5.32(0.379)	5.34(0.355)	5.54(0.438)	5.39(0.377)
**Systolic blood pressure, mmHg, mean (±SD)**	120.1(±11.76)	116.6(±13.03)	116.7(±12.57)	122.9(±13.24)	118.9(±12.88)
**Diastolic blood pressure, mmHg, mean (±SD)**	80.1(7.13)	77.3(8.44)	77.5(9.68)	80.9(6.70)	78.8(8.27)
**Heart rate (beat/min)**					
**Mean (SD)**	73.5(10.05)	72.5(8.29)	73.1(7.86)	69.1(12.18)	72.0(9.69)
**eGFR (mL/min/1.73 m^2, mean (±SD)**	93.6(20.86)	96.3(16.29)	95.0(19.36)	97.6(14.11)	95.7(17.52)

**Total cholesterol (mg/dL), mean (±SD)**	179.3(±35.10)	172.9(±30.73)	172.6(±26.38)	174.3(±33.38)	174.3(±30.91)
**High-density lipoprotein (mg/dL), mean (±SD)**	49.5(8.56)	46.9(11.34)	49.0(11.14)	48.2(12.29)	48.3(11.01)
**Low-density lipoprotein (mg/dL), mean (±SD)**	109.8(±31.08)	104.2(±24.54)	102.5(±24.76)	104.9(±30.34)	104.9(±27.27)
**Very low-density lipoprotein cholesterol (mg/dL), mean (±SD)**	20.0(7.29)	21.8(10.15)	21.2(8.86)	21.1(7.86)	21.1(8.68)
**Triglycerides (mg/dL)**					
**Mean (SD)**	99.9(36.42)	109.4(50.91)	105.7(44.48)	105.7(39.02)	105.6(43.44)

% = 100 x n/N; BMI = body mass index; DBP = diastolic blood pressure; eGFR = estimated glomerular filtration rate; HbA1c = hemoglobin A1c; HDL = high-density lipoprotein; LDL = low-density lipoprotein; MASH = metabolic dysfunction-associated steatohepatitis; Min = minimum; Max = maximum; SBP = systolic blood pressure; SD = standard deviation; VLDL = very low-density lipoprotein cholesterol; OLE = open label extension. Baseline is defined as the last measurement prior to the first dose of study drug in OLE phase.

Supplementary Table 3. CYP3A4 inhibition: recommendation from the product label of concomitant medications that are sensitive CYP3A4 substrates

Concomitant Medication	Instructions for Use with Strong or Moderate CYP3A4 Inhibitors	Instructions for Use with Weak CYP3A4 Inhibitors
Drugs that have specific recommendations when concomitantly used with a weak CYP3A inhibitor		
Darifenacin	The systemic exposure of darifenacin from Enablex extended-release tablets is increased in the presence of CYP3A4 inhibitors. The daily dose of Enablex should not exceed 7.5 mg when coadministered with potent CYP3A4 inhibitors (for example, ketoconazole, itraconazole, ritonavir, nelfinavir, clarithromycin and nefazadone).	No dosing adjustments are recommended in the presence of mild or moderate CYP3A4 inhibitors.

Lemborexant	Strong or moderate CYP3A inhibitors: Avoid concomitant use.	The maximum recommended dose is 5 mg.
Lomitapide	Strong and moderate CYP3A4 inhibitors are contraindicated with JUXTAPID. Participants must avoid grapefruit juice.	Do not exceed 30 mg daily of JUXTAPID when used concomitantly with weak CYP3A4 inhibitors.
Naloxegol	Concomitant use with strong CYP3A4 inhibitors is contraindicated.<br>Moderate CYP3A4 inhibitors: Increased naloxegol concentrations; avoid concomitant use; if unavoidable, reduce dosage to 12.5 mg once daily and monitor for adverse reactions.	Clinically significant increases in naloxegol concentrations are not expected. No dosage adjustments are necessary.
Drugs that do not distinguish between potent, moderate, or weak CYP3A4 inhibitors and recommend monitoring with or without dose reductions		
Alfentanil	Monitor participants closely at frequent intervals and consider dosage reduction of alfentanil HCI injection.	
Budesonide	If treatment with inhibitors of CYP3A4 activity (such as ketoconazole, itraconazole, ritonavir, indinavir, saquinavir, erythromycin, etc.) is indicated, reduction of the budesonide dose should be considered.	
Buspirone	Subsequent dose adjustment may be necessary and should be based on clinical assessment.	
Darunavir	Administration of darunavir and ritonavir and other drugs that inhibit CYP3A may decrease the clearance of darunavir and ritonavir and may result in increased plasma concentrations of darunavir and ritonavir.	
Felodipine	Caution should be used when CYP3A4 inhibitors are coadministered with felodipine. A conservative approach to dosing felodipine should be taken.	
Indinavir	Coadministration of CRIXIVAN and other drugs that inhibit CYP3A4 may decrease the clearance of indinavir and may result in increased plasma concentrations of indinavir. Dose reductions should be considered.	
Maraviroc	Reduce dose to 150 mg twice daily in participants receiving CYP3A4 inhibitors.	
Midazolam	Caution is advised when midazolam injection is administered concomitantly with drugs that are known to inhibit the P450-3A4 enzyme. These drug interactions may result in prolonged sedation caused by a decrease in plasma clearance of midazolam.	
Nisoldipine	Known inducer or inhibitor of CYP3A4 should be avoided in general.	
Tacrolimus	Since tacrolimus is metabolized mainly by CYP3A enzymes, drugs or substances known to inhibit these enzymes may increase tacrolimus whole blood concentrations. Monitoring of whole blood concentrations and appropriate dosage adjustments of tacrolimus are recommended.	
Drugs that have no recommendations for concomitant use of a weak CYP3A4 inhibitor (exercise caution, monitor and reduce dose as needed)		
Avanafil	Do not use STENDRA in participants taking strong CYP3A4 inhibitors.	None
Conivaptan	The coadministration of VAPRISOL with strong CYP3A inhibitors, such as ketoconazole, itraconazole, clarithromycin, ritonavir, and indinavir, is contraindicated.	None

Dasatinib	The coadministration with strong CYP3A inhibitors may increase dasatinib concentrations. Increased dasatinib concentrations may increase the risk of toxicity. Avoid concomitant use of strong CYP3A4 inhibitors. If concomitant administration of a strong CYP3A4 inhibitor cannot be avoided, consider a SPRYCEL dose reduction.	None
Dronedarone	Concomitant use of a strong CYP3A inhibitor are contraindicated.	None
Eletriptan	Strong CYP3A4 inhibitors significantly increase the exposure of RELPAX. Relpax should not be	None
Eplerenone	Because eplerenone metabolism is predominantly mediated via CYP3A4, do not use INSPRA with drugs that are strong inhibitors of CYP3A4.<br>Reduce the starting dose for hypertension to 25 mg once daily when used with moderate CYP3A4 inhibitors (e.g., verapamil, erythromycin, saquinavir, fluconazole).	None
Ibrutinib	Avoid concomitant use of other strong CYP3A inhibitors.<br>Dose modifications of IMBRUVICA are recommended when used concomitantly with moderate CYP3A inhibitors.	None
Isavuconazonium sulfate (isavuconazole)	Contraindicate coadministration of all potent CYP3A4 inhibitors.	None
Ivabradine	The concomitant use of strong CYP3A4 inhibitors is contraindicated. Avoid concomitant use of moderate CYP3A4 inhibitors when using Corlanor.	None
Lovastatin (lactone)	Concomitant administration with strong CYP3A4 inhibitors are contraindicated.	None
Lurasidone	Strong CYP3A4 inhibitors: Recommended dosage is 10.5 mg once daily.<br>Moderate CYP3A4 inhibitors: Recommended dosage is 21 mg once daily.	None
Mobocertinib	Avoid concomitant use of EXKIVITY with strong or moderate CYP3A inhibitors. If concomitant use of a moderate CYP3A inhibitor is unavoidable, reduce the dose of EXKIVITY.	None
Quetiapine	The dose of SEROQUEL should be reduced to one-sixth of the original dose if coadministered with a strong CYP3A4 inhibitor.	None
Sildenafil	A starting dose of 25 mg of VIAGRA should be considered in participants taking erythromycin or strong CYP3A4 inhibitors.	None
Simvastatin (lactone)	Concomitant use of these drugs and any medication labeled as having a strong inhibitory effect on CYP3A4 should be avoided unless the benefits of combined	None

	therapy outweigh the increased risk.	
Ticagrelor	Strong CYP3A inhibitors substantially increase ticagrelor exposure and thus increase the risk of dyspnea, bleeding, and other adverse events.<br>Avoid use of strong inhibitors of CYP3A.	None
Tolvaptan	Concomitant use of tolvaptan with strong CYP3A inhibitors is contraindicated. Avoid coadministration of SAMSCA with moderate CYP3A inhibitors.	None
Triazolam	Potent inhibitors of CYP 3A that should not be used concomitantly with triazolam.	None
Vardenafil	A single 2.5 mg dose of Levitra should not be exceeded in a 24-hour period when used with potent CYP3A4 inhibitors. A single 5 mg dose should not be exceeded in a 24 hour period when used with moderate CYP3A4 inhibitors	None

Supplementary Table 4. Excluded medications

Class and potential effect	List of excluded medications			
Sensitive P-gp substrates	dabigatran etexilate<br>digoxin	everolimus	fexofenadine<br>sirolimus	venetoclax
Sensitive BCRP substrates	ozanimod	sulfasalazine		
Sensitive CYP3A4 substrates[a]	lovastatin	simvastatin		
P-gp inhibitors (potential to increase exposure to aleniglipron)	amiodarone<br>azithromycin<br>capmatinib<br>carvedilol<br>clarithromycin<br>curcumin<br>cyclosporine<br>digoxin<br>diosmin<br>dronedarone<br>erythromycin	fexofenadine<br>flibanserin<br>fluvoxamine<br>glecaprevir<br>indinavir<br>itraconazole<br>ketoconazole<br>lapatinib lopinavir<br>nirmatrelvir<br>osimertinib	pibrentasvir<br>piperine quercetin<br>quinidine<br>ranolazine<br>rifampin ritonavir<br>saquinavir<br>*Schisandra chinensis*<br>sofosbuvir	telaprevir tepotinib<br>tipranavir valspodar<br>velpatasvir<br>vemurafenib<br>verapamil<br>voxilaprevir
P-gp inducers (potential to decrease exposure to aleniglipron)	apalutamide<br>avasimibe<br>carbamazepine	curcumin<br>dan-shen [*Salvia miltiorrhiza*]	genistein<br>green tea<br>lorlatinib	rifampin<br>St. John's wort<br>tivantinib
Strong CYP3A4 inhibitors (potential to increase exposure to aleniglipron)	adagrasib<br>boceprevir<br>ceritinib<br>clarithromycin<br>cobicistat<br>danoprevir<br>dasabuvir<br>elvitegravir<br>ensartinib	ensitrelvir<br>idelalisib indinavir<br>itraconazole<br>josamycin<br>ketoconazole<br>lonafarnib<br>lopinavir<br>mibefradil	mifepristone<br>nefazodone<br>nelfinavir<br>nirmatrelvir<br>ombitasvir<br>paritaprevir<br>posaconazole<br>relacorilant<br>ribociclib	ritonavir saquinavir<br>telaprevir<br>telithromycin<br>tipranavir<br>troleandomycin<br>tucatinib<br>voriconazole

Moderate CYP3A4 inhibitors at study start[b]<br>(have the potential to increase exposure to aleniglipron)	amprenavir<br>aprepitant<br>atazanavir<br>berotralstat<br>casopitant<br>ciprofloxacin<br>clofazimine<br>conivaptan crizotinib	diltiazem<br>dronedarone<br>duvelisib<br>erythromycin<br>faldaprevir<br>fedratinib<br>fluconazole<br>grapefruit juice<br>imatinib	ipatasertib<br>isavuconazole<br>lefamulin<br>lenacapavir<br>letermovir<br>netupitant<br>nilotinib<br>nivasorexant<br>ravuconazole	ritlecitinib<br>*Schisandra sphenanthera*<br>tofisopam<br>treosulfan<br>verapamil<br>voxelotor<br>ziritaxestat
Strong CYP3A4 inducers<br>(potential to decrease exposure to aleniglipron)	apalutamide<br>avasimibe<br>carbamazepine	enzalutamide<br>ivacaftor<br>ivosidenib	lumacaftor<br>mitotane<br>phenytoin	rifampin rifapentine<br>St. John's wort

[a]As lovastatin and simvastatin are sensitive CYP3A4 substrates, concomitant use of these medications is not allowed. Participants taking lovastatin or simvastatin should be switched to rosuvastatin at least 2 days prior to randomization. Participants on other statin drugs, including atorvastatin or rosuvastatin, are allowed to be enrolled. [b]If acute treatment with a moderate inhibitor is required post-study start and there is no alternative treatment, the Investigator will contact the Sponsor for assessment based on the aleniglipron doses being administered (coadministration at low doses could be feasible). Chronic concomitant treatment with moderate CYP3A4 inhibitors would not be allowed post-study start. BCRP = breast cancer resistance protein; P-gp = P-glycoprotein; CYP3A4 = cytochrome p-450 3A4.

This supplement contains the following items:

1. Original protocol for Study 06, Final protocol for Study 06 + Open Label Extension, Summary of changes

2. Original statistical analysis plan for Study 06, Original statistical analysis plan for Open Label Extension (no changes made to the original SAPs)

TITLE PAGE

Protocol Title:

A Phase 2b, Randomized, Double-blind, Placebo-controlled, Dose-range Finding Study of the Efficacy and Safety of Multiple Doses of GSBR-1290 in Participants Living with Obesity (Body Mass Index ≥ 30 kg/m^2) or Overweight (Body Mass Index ≥ 27 kg/m^2) with at Least One Weight-related Comorbidity

Brief Title: GSBR-1290 Phase 2b Obesity Study

Protocol Number: GSBR-1290-06

Version: Original protocol

Investigational Product: GSBR-1290

Study Phase: 2b

Regulatory Agency Identifier Number: United States IND 166912

Sponsor Name: Gasherbrum Bio, Inc.

Legal Registered Address: 601 Gateway Blvd., Suite 900
South San Francisco, CA 94080

Sponsor Medical Representative: [redacted]

Approval Date: 26 JUL 2024

PRINCIPAL INVESTIGATOR SIGNATURE PAGE

Study Title:

A Phase 2b, Randomized, Double-blind, Placebo-controlled, Dose-range Finding Study of the Efficacy and Safety of Multiple Doses of GSBR-1290 in Participants Living with Obesity (Body Mass Index ≥ 30 kg/m^2) or Overweight (Body Mass Index ≥ 27 kg/m^2) with at Least One Weight-related Comorbidity

Signature of Site Principal Investigator	***Date***
Printed Name of Site Principal Investigator<br>**Institution Name:**	

I, the Principal Investigator, submit this statement of commitment as evidence that I understand my responsibilities pursuant to the Code of Federal Regulations (21 CFR § 312) and International Council for Harmonization of Technical Requirements for Pharmaceuticals for Human Use E6(R2) Good Clinical Practice guidelines, as well as with any and all applicable federal, state, and/or local laws and regulations, and agree to conduct this study in accordance with the protocol referenced herein.

SPONSOR SIGNATURE PAGE

This study will be conducted in compliance with the current approved version of the clinical study protocol, Good Clinical Practice (GCP), and applicable regulatory requirement(s).

Study Title:

A Phase 2b, Randomized, Double-blind, Placebo-controlled, Dose-range Finding Study of the Efficacy and Safety of Multiple Doses of GSBR-1290 in Participants Living with Obesity (Body Mass Index ≥30 kg/m^2) or Overweight (Body Mass Index ≥27 kg/m^2) with at Least One Weight-related Comorbidity

By my signature, I confirm that I have reviewed this protocol and find its content to be acceptable.

[redacted] Date

TABLE OF CONTENTS

TITLE PAGE 1
PRINCIPAL INVESTIGATOR SIGNATURE PAGE 2
SPONSOR SIGNATURE PAGE 3
TABLE OF CONTENTS 4
LIST OF TABLES 9
LIST OF FIGURES 9
LIST OF ABBREVIATIONS 10
Pharmacokinetic Abbreviations and Terms 12
1. PROTOCOL SUMMARY 13
1.1. Synopsis 13
1.2. Schema 17
1.3. Schedule of Assessments (SoA) 19
2. INTRODUCTION 22
2.1. Background 22
2.1.1. People Living with Overweight or Obesity 22
2.1.2. Therapy for People Living with Overweight or Obesity 22
2.1.3. GSBR-1290 Clinical Overview 23
2.1.3.1. Phase 1 Study (GSBR-1290-01) 23
2.1.3.2. Phase 1 Study (GSBR-1290-03) 23
2.1.3.3. Phase 1b/2a Study (GSBR-1290-02) 24
2.1.3.4. Phase 1 Study (GSBR-1290-05) 25
2.1.3.5. Ongoing Phase 1 Study (GSBR-1290-07) 25
2.2. Study Rationale 25
2.3. Benefit/Risk Assessment 26
2.3.1. Risk Assessment 26
2.3.2. Benefit Assessment 27
2.3.3. Overall Benefit-Risk Conclusion 28
3. OBJECTIVES, ENDPOINTS, AND ESTIMANDS 29
4. STUDY DESIGN 30
4.1. Overall Design 30
4.2. Scientific Rationale for Study Design 31

4.3. Justification for Dose 31
4.4. End of Study Definition 31
5. STUDY POPULATION 33
5.1. Inclusion Criteria 33
5.2. Exclusion Criteria 34
5.3. Lifestyle Considerations 39
5.3.1. Nutrition and Good Health Instructions 39
5.3.2. Meals and Nutritional Restrictions 39
5.3.3. Caffeine and Tobacco 39
5.4. Screen Failures 39
6. STUDY INTERVENTION(S) AND CONCOMITANT THERAPY 40
6.1. Study Intervention(s) Administered 40
6.2. Dose Modification/Titration Adjustments 40
6.2.1. Dose Reduction 40
6.2.2. Dose Interruption/Hold 41
6.2.3. Management of Participants with Gastrointestinal Symptoms 41
6.3. Identification and Description of the Investigational Product 42
6.4. Supply and Labeling of Investigational Product 42
6.5. Preparation, Handling, Storage, and Accountability 43
6.6. Assignment to Study Intervention 43
6.7. Blinding and Breaking Code 44
6.8. Study Intervention Compliance 44
6.9. Continued Access to Study Intervention After the End of the Study 45
6.10. Treatment of Overdose 45
6.11. Prior and Concomitant Therapy 46
7. DISCONTINUATION OF STUDY INTERVENTION AND PARTICIPANT DISCONTINUATION/WITHDRAWAL 47
7.1. Discontinuation of Study Intervention (Investigational Product) 47
7.1.1. Liver Chemistry Stopping Criteria 47
7.1.2. QTc Stopping Criteria 48
7.2. Participant Discontinuation/Withdrawal From the Study 48
7.3. Lost to Follow-up 49
8. STUDY ASSESSMENTS AND PROCEDURES 50

8.1. Baseline Procedures 50
8.2. Participant-reported Outcomes 52
8.2.1. Electronic Diary 52
8.2.2. Impact of Weight on Quality of Life-Lite CT, Short Form-36 Acute Version, and Hunger and Satiety Questionnaires 52
8.3. Safety Assessments 52
8.3.1. Physical Examinations 53
8.3.2. Vital Signs 53
8.3.3. Electrocardiograms 53
8.3.4. Clinical Laboratory Tests 54
8.3.4.1. Clinical Laboratory Safety Tests 54
8.3.4.2. Other Clinical Laboratory Tests 54
8.3.5. Pregnancy Testing 55
8.3.6. PHQ-9 and C-SSRS Questionnaires 55
8.4. Adverse Events, Serious Adverse Events, and Other Safety Reporting 55
8.4.1. Adverse Events and Serious Adverse Events 55
8.4.2. Adverse Events of Special Interest 56
8.4.2.1. Major Adverse Cardiovascular Events 56
8.4.2.2. Heart Failure Syndrome 56
8.4.2.3. Acute Pancreatitis 56
8.4.2.4. Hypoglycemia 56
8.4.2.5. Thyroid Malignancies and C-cell Hyperplasia 57
8.4.2.6. Supraventricular Arrythmias and Cardiac Conductive Disorders 57
8.4.2.7. Hepatobiliary Disorders 57
8.4.2.8. Severe Gastrointestinal Adverse Events 57
8.4.2.9. Acute Renal Events 57
8.4.2.10. Depressive Disorder and/or Suicidal Ideation 58
8.4.2.11. Hypersensitivity 58
8.4.3. Investigator-reported Outcomes 58
8.4.3.1. Time Period and Frequency for Collecting Adverse Event and Serious Adverse Event Information 58
8.4.3.2. Method of Detecting Adverse Events and Serious Adverse Events 59
8.4.3.3. Follow-up of Adverse Events and Serious Adverse Events 59

8.4.3.4. Pregnancy 59
8.4.4. Adjudication Committee for Selected Adverse Events 60
8.4.5. Independent Data Monitoring Committee 61
8.5. Pharmacokinetics 61
8.6. Pharmacodynamics 61
8.7. Blood Sample Volumes 62
9. STATISTICAL CONSIDERATIONS 63
9.1. Analysis Sets 63
9.2. Statistical Analyses 63
9.2.1. Participant Disposition 64
9.2.2. Efficacy Data 64
9.2.2.1. Primary Endpoint 64
9.2.2.2. Secondary Endpoints 65
9.2.2.3. Exploratory End Points 65
9.2.3. Demographic and Baseline Characteristics 65
9.2.4. Safety/Tolerability 66
9.2.4.1. Extent of Exposure 66
9.2.4.2. Adverse Event 66
9.2.4.3. Prior and Concomitant Medication 66
9.2.4.4. Vital Signs 66
9.2.4.5. Physical Examination Data 67
9.2.4.6. Clinical Laboratory Data 67
9.2.4.7. Electrocardiogram Data 67
9.2.5. Pharmacokinetic Data 67
9.2.5.1. Plasma Pharmacokinetic Parameters 67
9.2.6. Pharmacodynamic Data 68
9.2.7. Subgroup Analyses 68
9.3. Interim Analysis 68
9.4. Sample Size Determination 69
10. SUPPORTING DOCUMENTATION AND OPERATIONAL CONSIDERATIONS 70
10.1. Appendix 1: Regulatory, Ethical, and Study Oversight Considerations 70
10.1.1. Regulatory and Ethical Considerations 70

10.1.1.1. Investigator Responsibilities 70
10.1.1.2. Ethical Considerations 70
10.1.2. Informed Consent Process 71
10.1.3. Data Protection 71
10.1.4. Study Protocol Guidelines 71
10.1.4.1. Emergency Contact With Investigator 71
10.1.4.2. COVID-19 Preparedness 71
10.1.4.3. Protocol Deviations and Violations 72
10.1.4.4. Protocol Waivers 72
10.1.4.5. Protocol Amendments 72
10.1.5. Quality Control and Assurance 72
10.1.5.1. Study Monitoring 72
10.1.5.2. Quality Assurance and Quality Control 73
10.1.6. Dissemination of Clinical Study Data 74
10.1.7. Source Documents 74
10.1.8. Electronic Case Report Form 75
10.1.9. Recordkeeping and Retention of Records 75
10.1.10. Study and Site Start and Closure 76
10.1.10.1. Notification of the General Practitioner 76
10.1.10.2. Clinical Laboratory Certification and Reference Ranges 76
10.1.10.3. Study/Site Termination 76
10.1.10.4. Study Completion/Site Closure 76
10.1.10.5. EOS and Regulatory Notification 77
10.1.11. Financing and Insurance 77
10.1.12. Intellectual Property, Confidentiality, and Publications 77
10.1.12.1. Ownership 77
10.1.12.2. Confidentiality 77
10.1.12.3. Publication Policy 78
10.2. Appendix 2: Clinical Laboratory Tests 79
10.3. Appendix 3: Adverse Events and Serious Adverse Events: Definitions and Procedures for Recording, Evaluating, Follow-up, and Reporting 79
10.3.1. Definition of Adverse Event 79
10.3.2. Definition of Serious Adverse Event 80

10.3.3. Definition of Suspected Unexpected Serious Adverse Reaction 80
10.3.3.1. Assessment of Intensity 81
10.3.3.2. Assessment of Causality 81
10.3.4. Action Taken and Outcome 82
10.3.5. Recording Adverse Events and/or Serious Adverse Events 83
10.3.5.1. Follow-up of Adverse Events and Serious Adverse Events 84
10.3.6. Reporting of Serious Adverse Events, Pregnancy, and Suspected Unexpected Serious Adverse Reactions 84
10.4. Appendix 4: Contraceptive and Barrier Guidance 85
10.5. Appendix 5: Clinical Inhibitors and Inducers 86
10.6. Appendix 6: Evaluation of Potential Drug-Induced Liver Injury 90
11. REFERENCES 93

LIST OF TABLES

Table 1: ..... Overall Schedule of Assessments 19
Table 2: .....Pharmacokinetic Sampling Schedule 21
Table 3: .....Study Intervention(s) Administered 40
Table 4: .....Approximate Blood Sample Volumes to be Collected per Participant 62
Table 5: .....Full Laboratory Tests 79
Table 6: .....NCI-CTCAE 5-Point Scale for Grading the Intensity of Each AE and SAE 81
Table 7: .....Relationship of the Study Treatment to an AE (Definitions) 82
Table 8: .....Terms and Definitions Used in Assessing the Final Outcome of an AE 83
Table 9: .....CYP3A4 Inhibition: Recommendation from the Product Label of Concomitant Medications that are Sensitive CYP3A4 Substrates 87

LIST OF FIGURES

Figure 1: ....Diagrammatic Representation of Study Design 18
Figure 2: ....Order of Procedures 50

LIST OF ABBREVIATIONS

Abbreviation	Definition or Explanation
AC	Adjudication Committee
AE	adverse event
AESI	adverse event of special interest
ALP	alkaline phosphatase
ALT	alanine transaminase
AST	aspartate transaminase
BCRP	breast cancer resistance protein
BMI	body mass index
BP	blood pressure
CFR	Code of Federal Regulations
COVID-19	coronavirus disease 2019
CRO	contract research organization
CSR	clinical study report
C-SSRS	Columbia Suicide Severity Rating Scale
CYP	cytochrome P450 enzyme
DILI	drug-induced liver injury
ECG	electrocardiogram
eCRF	electronic Case Report Form
eGFR	estimated glomerular filtration rate
EOS	end of study
ETV	early termination visit
FDA	Food and Drug Administration
GCP	Good Clinical Practice
GI	gastrointestinal
GLP-1RA	glucagon-like peptide-1 receptor agonist
HbA1c	hemoglobin A1c
HBsAg	hepatitis B surface antigen
hCG	human chorionic gonadotropin
HCV	hepatitis C virus
HDL	high-density lipoprotein
HIV	human immunodeficiency virus

Abbreviation	Definition or Explanation
HOV	healthy overweight/obese volunteer
HR	heart rate
ICE	intercurrent event
ICF	informed consent form
ICH	International Council for Harmonization of Technical Requirements for Pharmaceuticals for Human Use
IDMC	Independent Data Monitoring Committee
IND	Investigational New Drug
IP	investigational product
IRB / EC	Institutional Review Board/ Ethics Committee
IWQOL-Lite CT	Impact of Weight on Quality of Life-Lite Clinical Trials Version
LDL	low-density lipoprotein
MACE	major adverse cardiovascular event
MASH	metabolic dysfunction-associated steatohepatitis
MedDRA	Medical Dictionary for Regulatory Activities
MM	Medical Monitor
MMRM	mixed models for repeated measures
NCI-CTCAE	National Cancer Institute Common Terminology Criteria for Adverse Events
NHP	non-human primate
NLT	not less than
NOAEL	no-observed-adverse-effect level
PD	pharmacodynamic(s)
P-gp	P-glycoprotein
PHQ-9	Patient Health Questionnaire 9
PK	pharmacokinetic(s)
QD	once daily
QTcF	QT interval corrected using Fridericia's formula
RR	respiratory rate
SAD	single-ascending dose
SAE	serious adverse event
SAP	Statistical Analysis Plan
SD	standard deviation

Abbreviation	Definition or Explanation
SMR	Sponsor Medical Representative
SoA	Schedule of Assessments
SOC	system organ class
STEMI	ST-elevated myocardial infarction
SUSAR	suspected unexpected serious adverse reaction
T2DM	type 2 diabetes mellitus
TBL	total bilirubin
TEAE	treatment-emergent adverse event
THC	tetrahydrocannabinol
ULN	upper limit of normal
US	United States
VAS	visual analogue scale
WHO	World Health Organization
WHO-DD	World Health Organization Drug Dictionary
WOCBP	women of childbearing potential

Pharmacokinetic Abbreviations and Terms

$AUC_{0\text{-}tau}$	area under the concentration-time curve from 0 to tau, where tau is the dosing period of length at steady state
C_{max}	maximum observed plasma concentration
T_{max}	time to C_{max}

1. PROTOCOL SUMMARY

1.1. Synopsis

Protocol Title: A Phase 2b, Randomized, Double-blind, Placebo-controlled, Dose-range Finding Study of the Efficacy and Safety of Multiple Doses of GSBR-1290 in Participants Living with Obesity (Body Mass Index ≥30 kg/m^2) or Overweight (Body Mass Index ≥27 kg/m^2) with at Least One Weight-related Comorbidity

Rationale:

Obesity has reached epidemic proportions in most of the world, with more than 70% of adults in the United States (US) living with overweight or obesity (NIH, 2021). The World Health Organization estimates that in 2022, 1 in 8 people in the world were living with obesity, with 2.5 billion adults (18 years and older) overweight, 890 million of those living with obesity (WHO, 2024). Obesity is associated with various cardio-renal and metabolic diseases. Patients living with obesity are 2 to 4 times more likely to develop cardiovascular disease, including heart failure. Nearly 80% of patients with type 2 diabetes mellitus (T2DM) are living with either overweight or obesity. The number of people with obesity and overweight has been increasing steadily for over 20 years and is paralleled by the rates of people developing T2DM. Glucagon-like peptide receptor agonists (GLP-1RA) are a class of incretin peptides modeled after naturally occurring glucagon-like peptide-1 which functions to reduce appetite and decrease gastric motility. In the modified form, GLP-1RAs have an extended action and show a profound effect on appetite and induction of weight loss. Although the injectable GLP-1RA class of drugs was initially developed for their ability to increase insulin secretion in response to elevated glucose levels for the treatment of hyperglycemia in patients with T2DM, it has since become the staple for the treatment of overweight/obesity. Despite this advancement in weight management, there remains a concern for the cost, cold-storage requirement, and need for subcutaneous injection of this class of drugs. Therefore, there is a need for GLP-1RAs that can maintain effective concentrations throughout the dosing interval, can be stored easily (no refrigeration), and can be administered easily (orally with few, if any, restrictions regarding food/fluid intake and concomitant medications) to address the current unmet medical need.

GSBR-1001290 (GSBR-1290) is a potent, orally bioavailable, non-peptide GLP-1RA being developed as an adjunct to diet and exercise, to improve glycemic control in adults with T2DM under Investigational New Drug (IND) 160966, and for chronic weight management under Pre-IND 166912.

Under IND 160966, studies in non-human primate (NHP) models were performed, which showed that GSBR-1290 increased glucose-dependent insulin secretion and suppressed food intake with efficacy similar to the injectable peptidic GLP-1RA liraglutide. Based on nonclinical data, GSBR-1290 has the potential to achieve body weight loss similar to or greater than the effects observed with other GLP-1RAs, approved or in development, via the more convenient oral route of administration.

GSBR-1290 was well tolerated in a single-ascending dose clinical study at doses that provide exposures associated with glucose-lowering effects in NHPs. A recently completed (proof of principle) Phase 1b/2a study with GSBR-1290 (GSBR-1290-02) demonstrated relatively good tolerability and promising efficacy in both weight loss (in participants living with

obesity/overweight as well as participants living with T2DM) and hemoglobin A1c (HbA1c) reduction (T2DM participants).

This Phase 2b clinical study (the opening study for IND 166912) will evaluate the efficacy (body weight changes), safety, tolerability, pharmacokinetics (PK), and pharmacodynamics (PD) of various GSBR-1290 dose regimens compared with placebo in participants living with obesity or overweight with ≥1 weight-related comorbidity, in addition to diet and exercise, over a 36-week period.

Objectives and Endpoints:

	Objectives	Endpoints
**Primary**	To compare the efficacy of increasing doses of GSBR-1290 against placebo on body weight change	• Percent change in body weight from Baseline to Week 36
**Secondary**	To compare the efficacy of increasing doses of GSBR-1290 against placebo on body weight change	• Percentage of participants who achieve ≥5% reduction in body weight at Week 36<br>• Percentage of participants who achieve ≥10% reduction in body weight at Week 36<br>• Percentage of participants who achieve ≥15% reduction in body weight at Week 36<br>• Change in body weight (absolute) from Baseline to Week 36<br>• Change in waist circumference from Baseline to Week 36<br>• Change in body mass index from Baseline to Week 36
	To assess the safety and tolerability profile of a monthly titration scheme	• TEAEs and SAEs<br>• AESI<br>• Laboratory parameters<br>• Electrocardiogram<br>• Vital signs
**Exploratory**	To assess the plasma pharmacokinetics of GSBR-1290 and other parameters	• Change in triglycerides, FFA, and hsCRP to Week 36<br>• Change in patient-reported outcomes (SF-36 acute version, IWQOL-Lite CT, and hunger/satiety evaluation) from Baseline to Week 36

	Objectives	Endpoints
		• Change in HDL and LDL cholesterol from Baseline to Week 36<br>• Population pharmacokinetic parameters

Abbreviations: AESI=adverse event of special interest; FFA=free fatty acids; HDL=high-density lipoprotein; hsCRP=high-sensitivity C-reactive protein; IWQOL-Lite CT= Impact of Weight on Quality of Life-Lite Clinical Trials Version; LDL=low-density lipoprotein; PD=pharmacodynamic; SAEs=serious adverse events; SF-36=Short Form 36 health survey acute version; TEAEs=treatment-emergent adverse events.

Estimands:

The efficacy estimand is described by the following attributes:

Population: participants with obesity (body mass index [BMI] ≥30 kg/m^2) or overweight (BMI ≥27 kg/m^2) with at least one weight-related comorbidity who meet the inclusion criteria. Refer to Section 5.1 for more details.

Treatment: randomized treatment with titration to 1 of 3 target doses, with allowance of down-titration based on tolerability, or placebo.

Primary endpoint: percent change in body weight from Baseline to Week 36.

Intercurrent events (ICEs): permanent discontinuation of study drug.

Population-level summary: difference in percent change in body weight between GSBR-1290 (45, 90, and 120 mg once daily [QD]) and placebo will be analyzed using a mixed models for repeated measures model with treatment group, randomization strata (baseline BMI and sex), visit, treatment-by-visit interaction, and baseline measurement as covariates. An unstructured covariance model will be used.

The primary clinical question of interest is as follows: What is the treatment difference in percent change in body weight after 36 weeks of treatment in participants who meet the inclusion criteria and would have completed the treatment period?

The primary estimand will handle the ICEs by hypothetical strategy. When an ICE occurs, subsequent data will be regarded as invalid and set to missing.

The secondary estimand will handle the ICEs by treatment policy strategy. All observed values, including those collected after ICEs, will be used in the analysis.

The same estimands for the primary objective will be used for the efficacy endpoints for the secondary objectives.

Unless specified otherwise, safety and tolerability assessments will be guided by an estimand comparing safety of GSBR-1290 doses with placebo irrespective of adherence to study drug, including data collected during the treatment period plus safety follow-up from participants who are randomized and are exposed to at least 1 dose of study drug.

Overall Design Synopsis:

This is a randomized, double-blind, placebo-controlled, dose-range finding study of the efficacy, safety, tolerability, PK, and PD of multiple doses of GSBR-1290 in participants living with overweight or obesity.

The study will enroll male and female participants ≥18 years and <80 years of age at the time of Screening with a BMI ≥30 kg/m^2 (obese) or BMI ≥27 kg/m^2 (overweight) with ≥1 weight-related comorbidity and who have had a stable weight (no more than 5% variation) in the last 3 months prior to Screening. Enrollment of participants with a BMI <30 kg/m^2 will be capped at 10% of the planned sample size, with at least 60% of the participants enrolled with a BMI ≥35 kg/m^2. Enrollment of female participants will also be capped to no more than 65% of the planned sample size. Efforts will be made to ensure that ethnicity and race will be approximately representative of the US population in general (e.g., including approximately 18% Hispanic, 12% Black or African American, etc.) to ensure sufficient diversity based on 2023 US Census Data.

All on-study assessments to be performed are described in the Schedule of Assessments (Section 1.3). Blood samples will be collected at various times to assess biomarkers for safety monitoring as well as for PK assessments.

Number of Participants:

Approximately 220 participants at around 40 sites in the US will be randomized. Participants not meeting the eligibility criteria at Screening and Baseline will be considered a screen failure. Participants who screen fail will be allowed to be rescreened up to 2 times.

Enrollment Criteria

Full inclusion and exclusion criteria are provided in Section 5.1 and Section 5.2, respectively.

Key Inclusion Criteria:

Participants will be eligible if they satisfy ALL of the following pertinent criteria at Screening and Baseline.

- Signed informed consent before any study-related activities are initiated, must be able to understand the full nature and purpose of the trial (including possible risks and adverse effects), and be willing to comply with all study procedures and adhere to the protocol schedule and restrictions
- Men and women, age ≥18 years and <80 years, with:
  - BMI ≥30 kg/m^2

    or
  - BMI ≥27.0 kg/m^2 and previous/current diagnosis of ≥1 comorbidity: hypertension, dyslipidemia, obstructive sleep apnea, cardiovascular disease, heart failure syndrome, or metabolic dysfunction-associated steatohepatitis (MASH).
- Screening HbA1c <6.5%

Study Arms and Duration:

Participants will be randomized to GSBR-1290 or placebo in a ratio of 3:1 within each Cohort as follows:

- Cohort 1: Dose level 1 GSBR-1290 (maintenance dose: 45 mg QD) or placebo N=60
- Cohort 2: Dose level 2 GSBR-1290 (maintenance dose: 90 mg QD) or placebo N=80
- Cohort 3: Dose level 3 GSBR-1290 (maintenance dose: 120 mg QD) or placebo N=80

Participants will receive multiple-ascending, QD doses of GSBR-1290 or placebo in titration steps of 4 weeks duration. Thereafter, in the last titration step dose, participants will receive the target maintenance dose (target dose) of study drug for the remaining duration of treatment (for a total of 36 weeks of treatment).

The maximum study duration for participants is 41 weeks (4 weeks for Screening, 36 weeks for treatment, and 1 week for follow-up).

Titration Adjustments

Titration adjustments will be permitted upon documented discussion with the Sponsor to manage participants experiencing adverse events at the current dose. Adjustments will include down titrating to the next lower dose, or as needed, and then re-titrating up to the target dose, in consultation between the site Investigator and the Sponsor Medical Representative (SMR). Education and coaching on tolerance strategies will be provided by qualified study personnel to enable as many participants as possible to attain their target dose. Recommendations are detailed in the Study Procedural Manual.

1.2. Schema

This is a Phase 2b, multiple-dose, randomized, double-blind, placebo-controlled, dose-range finding study (Figure 1).

Figure 1: Diagrammatic Representation of Study Design

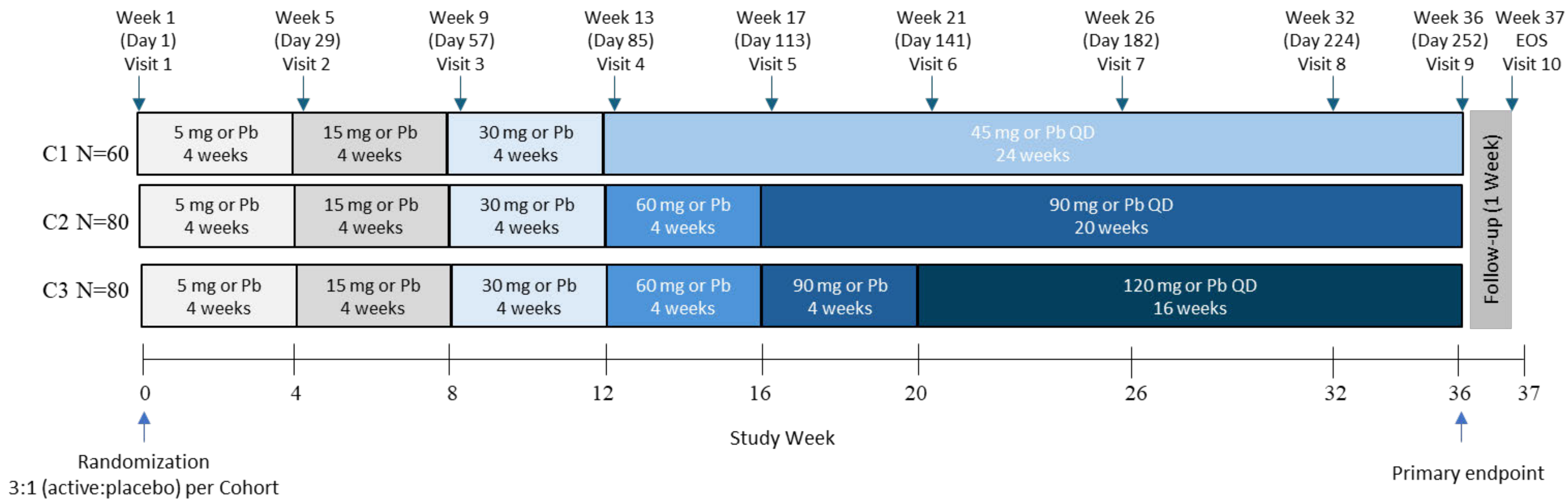

Abbreviations: C = cohort; N = number of participants; Pb = placebo; QD = once daily.

1.3. Schedule of Assessments (SoA)

Table 1: Overall Schedule of Assessments

Study periods	Screen	Treatment period									EOS /ETV[k]
Visit identifier	SCRN	1	2	3	4	5	6	7	8	9	10
Visit day	-28 to -1	1	29	57	85	113	141	182	224	252	259
Visit week	-4 to -1	1	5	9	13	17	21	26	32	36	37
Visit Interval Window (days)		0	±3	±3	±3	±3	±3	±3	±3	±3	±3
**Assessments**											
Informed consent	X										
Eligibility criteria	X										
Demographics	X										
Medical history	X										
Drug screen	X										
Body height in triplicate	X										
Body weight (with certified calibrated scales and in hospital gown) in triplicate	X	X	X	X	X	X	X	X	X	X	X
Waist circumference in triplicate		X					X		X	X	X
Concomitant medication	X	X	X	X	X	X	X	X	X	X	X
Physical examination (full)	X										X
Physical examination (symptom-directed)		X	X	X	X	X	X	X	X	X	
Vital signs (BP, HR, RR, temperature) [a]	X	X	X	X	X	X	X	X	X	X	X
12-Lead ECG [b]	X	X	X	X	X	X	X	X	X	X	X
COVID-19 test [c]	X	X									
FSH (postmenopausal women ≤ 55 years)	X										
Pregnancy test (urine) before dosing [d]		X			X		X			X	X
Pregnancy test (serum) before dosing	X										
Clinical laboratory tests [e]	X	X	X	X	X	X	X	X	X	X	X
Viral serology	X										
Adverse events [f]		X	X	X	X	X	X	X	X	X	X
Randomization		X									
Dose administration [g]		X	X	X	X	X	X	X	X	X	
HbA1c	X	X			X					X	

Study periods	Screen	Treatment period									EOS /ETV[k]
Visit identifier	SCRN	1	2	3	4	5	6	7	8	9	10
Visit day	-28 to -1	1	29	57	85	113	141	182	224	252	259
Visit week	-4 to -1	1	5	9	13	17	21	26	32	36	37
Visit Interval Window (days)		0	±3	±3	±3	±3	±3	±3	±3	±3	±3
**Assessments**											
PK sampling (blood) [h]		X	X	X	X	X	X	X	X	X	
Cardiovascular PD biomarkers (blood)[i]		X			X					X	
Fill out electronic diary daily, including dose administration time		X	X	X	X	X	X	X	X	X	
SF-36 acute version		X					X			X	X
IWQOL-Lite CT		X					X			X	X
Hunger and satiety evaluation[j]		X	X	X	X	X	X	X	X	X	X
PHQ-9 health questionnaires	X	X					X			X	X
C-SSRS screening/baseline	X										
C-SSRS since the last visit		X	X	X	X	X	X	X	X	X	X
Discuss nutrition plan and physical activity	X	X	X	X	X	X	X	X	X	X	

Abbreviations: BP = blood pressure; COVID-19 = coronavirus disease 2019; C-SSRS = Columbia Suicide Severity Rating Scale; ECG = electrocardiogram; EOS = end of study; ETV = early termination visit; FSH = follicle-stimulating hormone; HbA1c = hemoglobin A1c; HR = heart rate; IWQOL-Lite CT = Impact of Weight on Quality of Life-Lite Clinical Trials Version; PD = pharmacodynamic; PHQ-9 = Patient Health Questionnaire-9; PK = pharmacokinetic; RR= respiratory rate; SF-36 = Short Form 36 health survey acute version.

a. Vital signs including blood pressure and heart rate will be collected predose in triplicate. BP will be collected in 5-minute intervals. See Section 8 for order of procedures. Caffeine-containing products (e.g., coffee, tea, chocolate, and caffeine-containing sodas, colas, etc.) may not be consumed within 2 hours before measuring vital signs and ECGs
b. ECGs will be collected and stored centrally. ECGs will be collected in triplicate along with each PK draw and within 15 minutes before the PK draw. See Section 8 for order of procedures.
c. COVID-19 test only if clinically indicated or per site policy.
d. Urine pregnancy test is performed first, and if positive or inconclusive, confirmed with a serum pregnancy test.
e. Includes fasting glucose after Screening visit (i.e., starting on Day 1). Clinical safety laboratory tests will include hematology, serum chemistry, coagulation, and urinalysis.
f. Adverse events and serious adverse events collected from time of signing the informed consent form through 30 days after date of the last dose.
g. On-study visit days, participants should take all of their study drug at the clinic within 15 minutes due to the PK assessments being performed on those days.
h. For PK timepoints, refer to Table 2. See Section 8 for order of procedures.
i. PD markers, including, but not limited to, free fatty acid, high-sensitivity C-reactive protein, and succinate, will be measured at Day 1, Day 85, and Day 252. In addition, at Day 85, triglycerides, cholesterol, lipid panel, and other inflammatory markers will be measured.
j. The hunger and satiety evaluation will be performed for the first 3 days following each clinic visit.
k. ETV should be scheduled 7(±3) days from the last dose.

Note: Participants who discontinue study drug should return to the clinic at the next scheduled visit for the ETV assessments.

Table 2: Pharmacokinetic Sampling Schedule

Study Day	Study Week	Planned Dose (Irrespective of Down-Titration)	PK Timepoints (Exact Time of Blood Collection Must be Recorded)
Day 1	Week 1	All cohorts: start of 5 mg	Within 15 minutes before dosing<br>Between 4 and 6 hours postdose
Day 29	Week 5	All cohorts: start of 15 mg	Within 15 minutes before dosing<br>Between 4 and 6 hours postdose
Day 57	Week 9	All cohorts: start of 30 mg	Within 15 minutes before dosing<br>Between 4 and 6 hours postdose
Day 85	Week 13	Cohort 1 – start of 45 mg<br>Cohort 2 and 3 – start of 60 mg	Within 15 minutes before dosing<br>Between 4 and 6 hours postdose
Day 113	Week 17	Cohort 1 – after 4 weeks at 45 mg<br>Cohort 2 and 3 – start of 90 mg	Within 15 minutes before dosing<br>Between 4 and 6 hours postdose
Day 141	Week 21	Cohort 1 – after 8 weeks at 45 mg<br>Cohort 2 – after 4 weeks at 90 mg<br>Cohort 3 – start of 120 mg	Within 15 minutes before dosing<br>Between 4 and 6 hours postdose
Day 182	Week 26	At steady state for all cohorts	Within 15 minutes before dosing
Day 224	Week 32	At steady state for all cohorts	Within 15 minutes before dosing
Day 252	Week 36	Last day of dosing	Within 15 minutes before dosing<br>Between 4 and 6 hours postdose

Abbreviations: PK=pharmacokinetic.
Note: On-study days with PK assessments, all study tablets should be consumed at the clinic within 15 minutes.
Note: ECGs will be collected in triplicate along with each PK draw and within 15 minutes before the PK draw. See Laboratory Manual for details.
Note: To verify compliance with the study dosing regimen and adherence to the protocol requirements, random PK blood draws may be conducted on enrolled participants to monitor the concentration of the study drug in the bloodstream.

2. INTRODUCTION

2.1. Background

GSBR-1290 is an orally available glucagon-like peptide-1 receptor agonist (GLP-1RA) being developed as an adjunct to diet and exercise, to improve glycemic control in adult participants with type 2 diabetes mellitus (T2DM) (Investigational New Drug [IND] 160966), and for chronic weight management (Pre-IND 166912).

2.1.1. People Living with Overweight or Obesity

Obesity is a multifactorial disease with a complex pathogenesis in which biological, social, and environmental factors play a critical role (Powell-Wiley, et al., 2021). In addition, people living with overweight, or obesity can present with multiple other adverse health outcomes associated with a heterogeneity in the pathophysiologic pathways and biological mechanisms (Jastreboff, et al., 2019). Patients with a high body mass index (BMI) present with an increased risk of cardiovascular and all-cause mortality and higher incidence of major comorbid conditions, such as type 2 diabetes, hypertension, dyslipidemia, and cardiovascular diseases (Ghoorah, et al., 2016). The World Health Organization (WHO) defines overweight as a BMI ≥25 and <30 kg/m^2 and obesity as a BMI ≥30 kg/m^2 (WHO, 2024).

People living with overweight and obesity are highly prevalent in the general population. In the United States (US) and according to 2017 to 2018 data from the National Health and Nutrition Examination Survey, nearly 1 in 3 adults (30.7%) are overweight and more than 2 in 5 adults (42.4%) are obese (NIH, 2021). In addition, about 1 in 11 adults (9.2%) have severe obesity (BMI >35 kg/m^2) (NIH, 2021). The WHO estimates that in 2022, 1 in 8 people in the world were living with obesity (WHO, 2024).

2.1.2. Therapy for People Living with Overweight or Obesity

Multiple medical societies have issued treatment recommendations for the treatment of patients living with overweight or obesity, depending on the obesity severity (as approximated by BMI) and associated risk factors (T2DM, previous atherosclerotic cardiovascular disease, and others) (Apovian, et al., 2015; Garvey, et al., 2016). The Endocrine Society recommends that diet, exercise, and behavioral modification be included in all obesity management approaches for patients with BMI ≥25 kg/m^2 and that other tools such as pharmacotherapy be reserved for patients with BMI ≥27 kg/m^2 with comorbidity or BMI >30 kg/m^2. Behavioral modification to reduce food intake, increase physical activity, and reduce sedentariness should be implemented in addition to pharmacotherapies and/or bariatric surgery. As with other chronic disease states, the persistence and maintenance of therapeutic strategy is key to improve long-term outcomes. To achieve this goal, it is recommended to use approved weight loss medications that may also enhance adherence to behavioral changes.

While greater weight loss may be associated with greater health benefits, even a modest 5% to 10% body weight loss, such as that produced by lifestyle modifications and medications, has been shown to produce significant improvements in the control of T2DM (Apovian, et al., 2015; Davies, et al., 2022).

It is recommended that the patient's response to a pharmacologic or other intervention be monitored (both in terms of efficacy and tolerability/safety) on a regular basis (approximately every 3 months). Interventions that yield a weight loss reduction <5% may be ineffective, and on a case-by-case basis, the patient in consultation with the physician should evaluate potential alternatives (Apovian, et al., 2015).

The majority of approved therapies for obesity target central nervous system appetite centers. They work primarily in the arcuate nucleus to promote satiety. There are several classes of drugs in that category (serotoninergic, dopaminergic, or norepinephrine-releasing agents/reuptake inhibitors). Other classes of drugs include lipase inhibitor (orlistat), and most recently, GLP-1RAs (liraglutide and semaglutide), and the GLP-1RA + GIP tirzepatide.

2.1.3. GSBR-1290 Clinical Overview

2.1.3.1. Phase 1 Study (GSBR-1290-01)

GSBR-1290 has been evaluated in 1 completed single-ascending dose (SAD) clinical study in healthy individuals (Study GSBR-1290-01). This was a Phase 1, randomized, double-blind, placebo-controlled, SAD, multi-cohort study conducted in Australia. The primary endpoint was safety and tolerability (adverse events [AEs], vital signs, electrocardiogram [ECG] findings, and laboratory values) of single doses of GSBR-1290. The key secondary endpoint was pharmacokinetics (PK) of GSBR-1290.

Each cohort enrolled 8 participants, with 6 participants randomized to GSBR-1290 and 2 participants randomized to placebo. Participants in Cohorts 1, 2, 3, 5, and 6 received a single oral dose of GSBR-1290 at 1, 10, 30, 60, or 90 mg, respectively, under fasted conditions. Participants in Cohort 4 (food effect) received 1 dose of oral GSBR-1290 (15 mg) under fasted conditions and 1 dose of oral GSBR-1290 (15 mg) under fed conditions (i.e., total of 2 doses). The study enrolled 48 participants, 36 who received GSBR-1290 at 1 of 6 different dose levels and 12 participants who received placebo.

There were no deaths or other serious adverse events (SAEs) in this study. GSBR-1290 was safe when administered as a single dose of up to 90 mg and generally well tolerated at lower doses (1, 10, and 15 mg). As expected for the class of GLP-1RA drugs, there were apparent dose-related trends in the incidence, severity, and causality of treatment-emergent adverse events (TEAEs), particularly gastrointestinal (GI)-related TEAEs. The most commonly occurring TEAEs following GSBR-1290 treatment were nausea, headache, vomiting, dehydration, decreased appetite, dizziness, and diarrhea.

A detailed summary of results from Study GSBR-1290-01 is provided in the Investigator's Brochure.

2.1.3.2. Phase 1 Study (GSBR-1290-03)

GSBR-1290-03 was a Phase 1, randomized, double-blind, placebo-controlled, ethno-bridging study of the safety, PK, and pharmacodynamics (PD) of multiple-ascending doses of GSBR-1290 in healthy Japanese and non-Japanese participants.

The primary endpoint was safety and tolerability (AEs, vital signs, ECG findings, and laboratory values) of multiple doses of GSBR-1290. The key secondary endpoint was PK.

The study enrolled a total of 18 male and female healthy participants, 18 to 55 years of age (inclusive). Japanese and non-Japanese participants were enrolled to receive multiple-ascending doses of study drug (GSBR-1290 or placebo) from Day 1 to Day 28. Dose levels were titrated at weekly intervals and were evaluated in 2 cohorts.

Cohort 1 (double-blind) randomized 12 healthy adult Japanese participants to receive GSBR-1290 (n=9) or placebo (n=3) once daily (QD) for 7 consecutive days at each dose (5, 15, 30, and 60 mg), with a total of 4 doses titrated up over a 4-week period (i.e., 1 dose level per week).

Cohort 2 (open-label) enrolled 6 non-Japanese participants who received GSBR-1290 for 7 consecutive days at each dose (5, 15, 30, and 60 mg), with a total of 4 doses titrated up over a 4-week period to match the titration schedule in Cohort 1.

The majority of TEAEs were mild and were GI-related or appetite/satiety related.

GSBR-1290 demonstrated a weight reduction in Japanese participants (3.91% on GSBR-1290 versus 1.67% placebo) and in non-Japanese participants (5.13% not placebo-adjusted), with no discontinuations or dose reductions, and no SAEs.

2.1.3.3. Phase 1b/2a Study (GSBR-1290-02)

GSBR-1290-02 was a Phase 1b/2a, randomized, double-blind, Sponsor-open, placebo-controlled, multiple-ascending dose study in which dose levels with different titration steps were evaluated.

The primary endpoint was safety and tolerability (AEs, vital signs, ECG findings, and laboratory values) of multiple doses of GSBR-1290. The key secondary endpoints included decrease in hemoglobin A1c (HbA1c) for Cohort 5 and decrease in weight (absolute and percent) for Cohorts 4 and 5 of GSBR-1290 versus placebo over 4 to 12 weeks.

The study enrolled 142 male and female participants, ≥18 and ≤75 years of age, with a BMI ≥27.0 and ≤40.0 kg/m^2. Participants who had T2DM must have been on metformin not less than (NLT) 500 mg/day with a duration NLT 6 months and at stable doses for NLT 2 months.

This was a 2-part study:

Part 1: 24 Healthy overweight/obese volunteer (HOV) participants were enrolled in 3 cohorts (Cohorts 1 to 3) with each cohort having 8 participants; 6 participants were randomized to receive GSBR-1290 and 2 participants were randomized to receive placebo, and were dosed for 4 weeks, following a weekly or biweekly titration of doses ranging from 5 to 90 mg, administered once a day. Safety data showed a tolerability profile consistent with previous GLP-1RAs, with GI-related events being the most common, followed by headache. Most events were categorized as mild, spontaneously resolved, or sporadically needing symptomatic treatment. Those participants with AEs experienced a progressive decline in the incidence of events as the study was advancing to the prespecified up-titration, except for vomiting in Cohort 2 that experienced an increase coinciding with the 90 mg titration. The study evidenced a dose response for the most common AEs (nausea, vomiting, diarrhea, abdominal pain, and headache), with higher incidences in the groups targeting 60 or 90 mg of GSBR-1290 compared to the groups of 30 mg or placebo. No significant changes in laboratory parameters were found, including chemistry, hematology, and coagulation. Participants in Cohorts 1 and 2 showed an increasing trend in heart rate (HR) measured by ECG, as expected by the class of GLP-1RA

drugs. There were also a fluctuating number of participants with an increase of at least 20 beats per minute from baseline, up to Week 4. None of those observations were reported as AEs.

Part 2: HOV participants without T2DM (N=64) have been enrolled in Cohort 4. Participants with T2DM (N=54) were enrolled in Cohort 5. GSBR-1290 demonstrated clinically meaningful efficacy in both participants with T2DM (statistically significant HbA1c reduction) and participants living with overweight/obesity without T2DM (statistically significant reduction in weight). In the latter group (Cohort 4), GSBR-1290 demonstrated a clinically meaningful and statistically significant placebo-adjusted mean decrease in weight of 6.2% ($p<0.0001$) at 12 weeks. At Week 12, 67% of GSBR-1290-treated participants achieved ≥6% weight loss and 33% achieved ≥10% weight loss, compared to 0% for placebo. In Part 2, the incidence of TEAEs was similar across cohorts, including HOV participants (Cohorts 1 through 4) and participants with T2DM (Cohort 5), and higher for participants treated with GSBR-1290 compared with placebo. The majority of the TEAEs were mild to moderate in severity. The majority of TEAEs leading to dose reduction of study drug were gastrointestinal-related TEAEs.

2.1.3.4. Phase 1 Study (GSBR-1290-05)

Study GSBR-1290-05 was a 2-part, Phase 1, randomized study to evaluate the safety, tolerability, PK, and comparative bioavailability of multiple-ascending doses of GSBR-1290 in healthy overweight/obese participants.

The primary objectives of Study GSBR-1290-05 were to compare the PK of the GSBR-1290 capsule formulation (which has been used in the initial clinical studies: GSBR-1290-01, GSBR-1290-02, GSBR-1290-03) and a tablet formulation (planned to be used in future clinical studies, including the Phase 2b GSBR-1290-06 study) (Part 1), and to assess the safety, tolerability, and PK of multiple-ascending oral doses of GSBR-1290 (tablet) in HOVs in 3 parallel cohorts using 3 different titration regimens (Part 2).

In this 12-week study, GBSR-1290 was generally safe and well tolerated with the majority of TEAEs mild or moderate. In Part 1, reduction in mean body weight was observed in both GSBR-1290 10 mg tablet and capsule groups with changes similar between tablet and capsule groups. In Part 2 reductions in mean body weight from baseline were observed in all 3 GSBR-1290 cohorts. The results of Study GSBR-1290-05 are consistent with the previous studies (GSBR-1290-02 and GSBR-1290-03) in terms of the magnitude of the effect and also the dynamic of weight loss observed over 12 weeks.

2.1.3.5. Ongoing Phase 1 Study (GSBR-1290-07)

Study GSBR-1290-07 is a Phase 1 study to assess the mass balance, PK, metabolism, and excretion of GSBR-1290, containing a microtracer dose of [^{14}C]-GSBR-1290 in healthy adult participants. The study is being conducted in the Netherlands.

2.2. Study Rationale

Obesity has reached epidemic proportions in most of the world, with more than 70% of adults in the US living with overweight or obesity (NIH, 2021). The World Health Organization estimates that in 2022, 1 in 8 people in the world were living with obesity, with 2.5 billion adults (18 years and older) overweight, 890 million of those living with obesity (WHO, 2024). Obesity is associated with various cardio-renal and metabolic diseases. Patients living with obesity are 2 to

4 times more likely to develop cardiovascular disease, including heart failure. Nearly 80% of patients with T2DM are living with either overweight or obesity. The number of people with obesity and overweight has been increasing steadily for over 20 years and is paralleled by the rates of people developing T2DM. GLP-1RAs are a class of incretin peptides modeled after naturally occurring glucagon-like peptide-1 which functions to reduce appetite and decrease gastric motility. In the modified form, GLP-1RAs have an extended action and show a profound effect on appetite and induction of weight loss. Although the injectable GLP-1RA class of drugs was initially developed for their ability to increase insulin secretion in response to elevated glucose levels for the treatment of hyperglycemia in patients with T2DM, it has since become the staple for the treatment of overweight/obesity. Despite this advancement in weight management, there remains a concern for the cost, cold-storage requirement, and need for subcutaneous injection of this class of drugs. Therefore, there is a need for GLP-1RAs that can maintain effective concentrations throughout the dosing interval, can be stored easily (no refrigeration), and can be administered easily (orally with few, if any, restrictions regarding food/fluid intake and concomitant medications) to address the current unmet medical need.

GSBR-1001290 (GSBR-1290) is a potent, orally bioavailable, non-peptide GLP-1RA being developed as an adjunct to diet and exercise, to improve glycemic control in adults with T2DM under IND 160966, and for chronic weight management under Pre-IND 166912.

Under IND 160966, studies in non-human primate (NHP) models were performed, which showed that GSBR-1290 increased glucose-dependent insulin secretion and suppressed food intake with efficacy similar to the injectable peptidic GLP-1RA liraglutide. Based on nonclinical data, GSBR-1290 has the potential to achieve body weight loss similar to or greater than the effects observed with other GLP-1RAs, approved or in development, via the more convenient oral route of administration.

GSBR-1290 was well tolerated in a SAD clinical study at doses that provide exposures associated with glucose-lowering effects in NHPs. A recently completed (proof of principle) Phase 1b/2a study with GSBR-1290 (GSBR-1290-02) demonstrated relatively good tolerability and promising efficacy in both weight loss (in participants living with obesity/overweight as well as participants living with T2DM) and hemoglobin A1c (HbA1c) reduction (T2DM participants).

This Phase 2b clinical study (the opening study for IND 166912) will evaluate the efficacy (body weight changes), safety, tolerability, PK, and PD of various GSBR-1290 dose regimens compared with placebo in participants living with obesity or overweight with ≥1 weight-related comorbidity, in addition to diet and exercise, over a 36-week period.

2.3. Benefit/Risk Assessment

More detailed information about the known and expected benefits and risks and reasonably expected AEs of GSBR-1290 may be found in the Investigator’s Brochure.

2.3.1. Risk Assessment

GSBR-1290 is an investigational drug with safety data derived from 203 participants exposed to GSBR-1290 treatment in clinical studies (GSBR-1290-01, GSBR-1290-02, GSBR-1290-03, and GSBR-1290-05), up to 6 months (rodent) and 9 months (non-human primate) in vivo nonclinical, and extensive in vitro studies.

As with any investigational drug, unexpected clinically significant AEs or SAEs may occur with the use of GSBR-1290. All participants treated with GSBR-1290 should be closely monitored by means of AE monitoring, vital signs, physical examinations, ECGs, and clinical laboratory safety tests. The inclusion and exclusion criteria, screening, and safety monitoring practices used in this protocol are seen as adequate to protect the participants' safety and should detect all TEAEs.

The nonclinical safety of GSBR-1290 was also evaluated in 6- and 9-month repeat-dose toxicology studies in rats and NHPs, respectively. The safety margins are >200-fold at the no-observed-adverse-effect level (NOAEL) relative to the predicted exposure in humans receiving GSBR-1290 120 mg daily.

Rats were given oral gavage doses of 10, 100, or 1000 mg/kg/day QD for 26 weeks which was well tolerated and did not result in any mortality. The NOAEL was 1000 mg/kg/day.

Non-human primates were given oral gavage doses of 3, 10, or 30 mg/kg/day QD for 39 weeks which was tolerated and did not result in mortality or adverse findings. Test article-related changes included inappetence observed visually in animals administered ≥ 10 mg/kg/day, and transient vomiting and body weight loss in animals administered ≥ 3 mg/kg/day. The NOAEL was 30 mg/kg/day. Exposure in NHPs were limited by pharmacologically related GI effects, which can be closely monitored in humans and exceeded based on human tolerability, so rats are considered to be the most sensitive species to cap the projected safety margins.

Based on in vitro data, GSBR-1290 is a substrate for human P-glycoprotein (P-gp) and is metabolized by cytochrome P450 enzyme (CYP)3A4. Therefore, potent inducers or moderate to potent inhibitors of CYP3A4, as well as inducers or inhibitors of P-gp, will be excluded from this study due to their potential to increase/decrease GSBR-1290 exposure (see Section 10.5 for details).

GSBR-1290 is an in vitro inhibitor of P-gp and breast cancer resistance protein (BCRP). Based on these in vitro studies, this Phase 2b study will exclude participants who are taking concomitant medications that are P-gp or BCRP substrates. Because GSBR-1290 is only a weak CYP3A4 inhibitor, concomitant medications that are CYP3A4 substrates do not need to be excluded from this Phase 2b study; however, concomitant medications that are sensitive CYP3A4 substrates may require monitoring with or without dose reductions, according to the individual respective product labeling (see Section 10.5 for details).

GSBR-1290 has been assessed in 4 completed clinical studies (GSBR-1290-01, GSBR-1290-02, GSBR-1290-03, and GSBR-1290-05) with single-ascending doses up to 90 mg and multiple-ascending doses of up to 120 mg QD. To date, no important potential risks with GSBR-1290 have been identified.

The AE profile of GSBR-1290 in previous clinical studies was consistent with the side-effect profile of the GLP-1RA class of medicines, with AEs being mainly GI-related. Gastrointestinal symptoms will be carefully monitored in this study.

2.3.2. Benefit Assessment

The information gained in this trial could be of benefit to the public health and to individuals living with overweight or obesity in need of weight management intervention. Based on the preliminary results from Study GSBR-1290-02, GSBR-1290 demonstrated clinically meaningful activity in both participants with T2DM (statistically significant HbA1c reduction) and

participants living with overweight/obesity (statistically significant reduction in weight). This study is designed to evaluate a range of GSBR-1290 doses to assess optimal efficacy at 36 weeks, with a titration scheme to minimize known GI-related events. An oral GLP-1RA offers a user-friendly option for individuals struggling with overweight or obesity.

2.3.3. Overall Benefit-Risk Conclusion

This is a Phase 2b study; a complete understanding of the risks involved with administration of GSBR-1290 is not known. Frequent monitoring of safety data will be performed, with timely review and safety monitoring to be adjusted as required if any risks become apparent. An Independent Data Monitoring Committee (IDMC) and Adjudication Committee (AC) will review safety data on an ongoing basis. Measures to minimize the risk to participants will be implemented, including selection of a low starting dose, selection of study facilities incorporating constant medical supervision with access to emergency care, and appropriately trained study personnel.

3. OBJECTIVES, ENDPOINTS, AND ESTIMANDS

Objectives and Endpoints:

	Objectives	Endpoints
**Primary**	To compare the efficacy of increasing doses of GSBR-1290 against placebo on body weight change	• Percent change in body weight from Baseline to Week 36
**Secondary**	To compare the efficacy of increasing doses of GSBR-1290 against placebo on body weight change	• Percentage of participants who achieve ≥5% reduction in body weight at Week 36<br>• Percentage of participants who achieve ≥10% reduction in body weight at Week 36<br>• Percentage of participants who achieve ≥15% reduction in body weight at Week 36<br>• Change in body weight (absolute) from Baseline to Week 36<br>• Change in waist circumference from Baseline to Week 36<br>• Change in body mass index from Baseline to Week 36
	To assess the safety and tolerability profile of a monthly titration scheme	• TEAEs and SAEs<br>• AESI<br>• Laboratory parameters<br>• Electrocardiogram<br>• Vital signs
**Exploratory**	To assess the plasma pharmacokinetics of GSBR-1290	• Change in triglycerides, FFA, and hsCRP at Week 36<br>• Change in patient-reported outcomes (SF-36 acute version, IWQOL-Lite CT, and hunger/satiety evaluation) from Baseline to Week 36<br>• Change in HDL and LDL cholesterol from Baseline to Week 36<br>• Population pharmacokinetic parameters

Abbreviations: AESI=adverse event of special interest; FFA=free fatty acids; HDL=high-density lipoprotein; hsCRP=high-sensitivity C-reactive protein; IWQOL-Lite CT= Impact of Weight on Quality of Life-Lite Clinical Trials Version; LDL=low-density lipoprotein; PD=pharmacodynamic; SAEs=serious adverse events; SF-36=Short Form 36 health survey acute version; TEAEs=treatment-emergent adverse events.

4. STUDY DESIGN

4.1. Overall Design

This is a randomized, double-blind, placebo-controlled, dose-range finding study of the efficacy, safety, tolerability, PK, and PD of multiple doses of GSBR-1290 in participants living with obesity or overweight with at least 1 weight-related comorbidity. The starting doses and titration schema are based on results from the SAD study (GSBR-1290-01), the multiple-ascending dose study (GSBR-1290-02), the ethno-bridging PK study (GSBR-1290-03), as well as the formulation PK bridging and titration optimization study (GSBR-1290-05).

The study will enroll male and female participants ≥18 years and <80 years of age at the time of Screening with a BMI ≥30 kg/m^2 (obese) or BMI ≥27 kg/m^2 (overweight) with ≥1 weight-related comorbidity and who have had a stable weight (no more than 5% variation) in the last 3 months prior to Screening. Enrollment of participants with a BMI <30 kg/m^2 will be capped at 10% of the planned sample size, with at least 60% of the participants enrolled with a BMI ≥35 kg/m^2. Enrollment of female participants will also be capped to no more than 65% of the planned sample size. Efforts will be made to ensure that ethnicity and race will be approximately representative of the US population in general (e.g., including approximately 18% Hispanic, 12% Black or African American, etc.) to ensure sufficient diversity based on 2023 US Census Data.

The total number of participants is anticipated to be approximately 220 at around 40 sites in the US. Participants not meeting the eligibility criteria at Screening and Baseline will be considered a screen failure, with the exception of protocol specific and qualifying laboratory results not available on Day -1. Participants who screen fail will be allowed to be rescreened up to 2 times. If abnormal values are obtained for vital signs, ECG, clinical laboratory tests, viral serology, or drugs of abuse assessments in the first instance, that assessment may be repeated once (at the discretion of the Investigator and Sponsor to confirm eligibility).

All on-study assessments to be performed are described in the Schedule of Assessments (SoA) (Section 1.3).

Participants will be randomized to GSBR-1290 or placebo in a ratio of 3:1 within each cohort as follows:

- Cohort 1: Dose level 1 GSBR-1290 (45 mg QD) or placebo N=60
- Cohort 2: Dose level 2 GSBR-1290 (90 mg QD) or placebo N=80
- Cohort 3: Dose level 3 GSBR-1290 (120 mg QD) or placebo N=80

Participants will receive multiple-ascending doses of GSBR-1290 or placebo for 36 weeks in titration steps of 4 weeks per step. Thereafter, in the last titration step dose, participants will

receive the target dose of GSBR-1290 or placebo of their assigned cohort for the remaining duration of treatment (Figure 1).

Participants receiving placebo treatment will be included in each of the 3 cohorts in order to reduce the pill burden, improve logistics and instructions for participants, and to potentially facilitate higher compliance.

4.2. Scientific Rationale for Study Design

Study GSBR-1290-06 is a Phase 2 study designed to compare the ability of GSBR-1290 to reduce body weight (dose ranging from 45 to 120 mg) compared to placebo during a 36-week treatment period, in participants living with obesity, or overweight with at least 1 weight-related comorbidity and without diabetes mellitus. The primary endpoint is percent change in body weight from Baseline to Week 36. Evaluation of the primary endpoint at 36 weeks is considered adequate for evaluation of weight loss efficacy in a Phase 2 trial and will allow evaluation of exposure-response parameters for selection of dose levels and dose escalation schemes for future Phase 3 studies.

In addition, safety, and tolerability over a range of doses will enhance the benefit-risk characterization of GSBR-1290.

A placebo group in each cohort will be included to allow evaluation of the efficacy and safety attributable to GSBR-1290 in this population. A 4-week titration scheme was selected to enhance tolerability before escalation to the next higher dose level.

All participants will receive nutrition plan and physical activity counseling throughout the study.

4.3. Justification for Dose

This study is planned to evaluate a range of maintenance GSBR-1290 doses (45, 90, and 120 mg daily) compared to placebo to optimize efficacy (% change in body weight) at 36 weeks balanced with the need for a low starting dose and titration schemes to minimize known GI-related events associated with GLP-1RAs.

A starting GSBR-1290 dose of 5 mg was selected to minimize the GI-related tolerability effects (e.g., nausea and emesis), which are more likely to be observed at starting doses ≥10 mg. Based on the results from the completed and ongoing clinical studies with GSBR-1290, the starting dose of 5 mg is expected to be safe and tolerable.

This Phase 2b study plans for slow, 4-week titration steps to potentially further improve tolerability.

Maintenance doses in the range of 45 to 120 mg were selected aiming to characterize the exposure response. These doses are expected to be safe and well tolerated based on the available clinical data (up to 120 mg) and based on nonclinical safety studies (6-month Good Laboratory Practice toxicity in rats).

4.4. End of Study Definition

The end of study (EOS) is defined as the date of the last contact of the last participant involved in the study.

A participant is considered to have completed the study if the participant has completed all periods of the study, including the EOS visit.

If a participant meets the criteria of discontinuation of study (see Section 7.1), the study intervention should be permanently discontinued. The participant should return to the clinic within 7 (±3) days for the early termination visit assessments. That visit will become the EOS (discontinuation) visit.

5. STUDY POPULATION

Prospective approval of protocol deviations to recruitment and enrollment criteria, also known as protocol waivers or exemptions, is not permitted.

This study will enroll male and female participants ≥18 years and <80 years of age at the time of screening. All participants will have a BMI ≥27.0 kg/m^2. Enrollment of participants with a BMI <30 kg/m^2 will be capped at 10% of the planned sample size, with at least 60% of the participants enrolled with a BMI ≥35 kg/m^2. Enrollment of female participants will also be capped to no more than 65% of the planned sample size. Efforts will be made to ensure that ethnicity and race will be approximately representative of the US population in general (e.g., including approximately 18% Hispanic, 12% Black or African American, etc.) to ensure sufficient diversity based on 2023 US Census Data.

5.1. Inclusion Criteria

Participants are eligible to be included in the study only if all the following criteria apply at Screening and Baseline. If abnormal values are obtained for vital signs, ECG, clinical laboratory tests, viral serology, or drugs of abuse assessments in the first instance, assessment may be repeated once (at the discretion of the Investigator and Sponsor) to confirm eligibility.

1. Signed informed consent before any study-related activities are initiated, must be able to understand the full nature and purpose of the trial (including possible risks and adverse effects), and be willing to comply with all study procedures and adhere to the protocol schedule and restrictions.
2. Men and women, age ≥18 years and <80 years, with:
   - BMI ≥30 kg/m^2 or
   - BMI ≥27.0 kg/m^2 and previous/current diagnosis of ≥1 comorbidity:
     - Hypertension (as defined by a resting systolic blood pressure [BP] of ≥140 mmHg and a resting diastolic BP of ≥90 mmHg or receiving BP lowering medication for the treatment of hypertension)
     - Dyslipidemia as defined by a fasting plasma triglyceride ≥200 mg/dL (2.2 mM), fasting low-density lipoprotein (LDL) cholesterol of ≥100 mg/dL (2.5 mM) or a high-density lipoprotein (HDL) cholesterol <40 mg/dL (1.0 mM), or receiving treatment with an approved lipid lowering medication
     - Documented obstructive sleep apnea or receiving treatment for obstructive sleep apnea
     - Cardiovascular disease, including documented coronary artery atherosclerotic disease, peripheral arterial atherosclerotic disease, stroke, or transient ischemic attacks
     - Documented diagnosis of heart failure syndrome, New York Heart Association Class 1 or 2 only
     - Documented diagnosis of metabolic dysfunction-associated steatohepatitis (MASH) (confirmed by clinical, laboratory, imaging, or biopsy findings)

3. Screening HbA1c <6.5%
4. Reproductive status:
    a. Female participants of nonchildbearing potential, defined as either surgically sterilized (hysterectomy, bilateral salpingectomy, bilateral tubal ligation, or bilateral oophorectomy ≥6 weeks before screening) or postmenopausal (defined as no menses for ≥12 months without an alternative medical cause and a follicle -stimulating hormone level >40 IU/L at the Screening visit for postmenopausal women ≤55 years).
    b. Female participants of childbearing potential must have a negative serum pregnancy test at the Screening visit and a negative urine pregnancy test (or confirmatory negative serum pregnancy test) at check-in on Day -1. Additionally, they must not be or plan to be breastfeeding, must agree not to donate ova, not attempt to become pregnant and, if engaging in sexual intercourse with a male partner, must agree to use a highly effective method of contraception (refer to Section 10.4, Appendix 4) from the time of signing the informed consent form (ICF) until ≥30 days after the last dose of the study drug.
    c. Male participants must agree not to donate sperm and, if engaging in sexual intercourse with a female partner who could become pregnant, must agree to use a highly effective method of contraception (refer to Section 10.4, Appendix 4) from signing the ICF until ≥90 days after the last dose of study drug.

5.2. Exclusion Criteria

Participants are excluded from the study if any of the following criteria apply at the Screening visit or before dosing at the timepoints in the SoA (Section 1.3):

Diabetes-related Medical Conditions

1. Previous documented diagnosis of diabetes mellitus (except for gestational diabetes and the participant has an HbA1c <6.5%)

Obesity-related Medical Conditions

2. Self-reported change in body weight >5% within 3 months before Screening
3. Body weight ≤80 kg at Screening
4. Have a prior or planned surgical treatment for obesity (excluding liposuction or abdominoplasty, if performed >1 year prior to screening)
5. Have or plan to have endoscopic and/or device-based therapy for obesity or have had device removal within the last 6 months prior to Screening, including, but not limited to, mucosal ablation, gastric artery embolization, intragastric balloon, and duodenal-jejunal endoluminal liner
6. Have obesity induced by other endocrine disorders (such as Cushing's syndrome, Prader-Willi syndrome, or melanocortin 4 receptor deficiency)

Other Medical Conditions

7. History or presence of significant cardiovascular, pulmonary, hepatic, renal, hematological, GI, endocrine, immunologic, dermatologic, or neurological disease, including any acute illness or major surgery within the past 3 months, or any clinical laboratory abnormality determined by the Investigator or medically certified delegate to be clinically significant
8. Any chronic disorder or severe disease that, in the opinion of the Investigator, might jeopardize the participant's safety, compliance with the protocol, and/or ability to complete the study
9. History of treatment for cancer in the last 5 years (excludes surgically resected skin squamous cell or basal cell carcinoma, in situ cervical cancer, or in situ prostate cancer)
10. Serum calcitonin level at Screening of ≥20 ng/L if estimated glomerular filtration rate (eGFR) ≥60 mL/min/1.73 m^2 or ≥35 ng/L if eGFR <60 mL/min/1.73 m^2, or a personal or family history of medullary thyroid carcinoma or multiple endocrine neoplasia syndrome type 2. Values at Day -1 will not be considered for eligibility criteria
11. Have a thyroid stimulating hormone level outside the range of 0.4 to 6.0 mIU/L at Screening
12. History of chronic pancreatitis or idiopathic acute pancreatitis, or documented history of biliary stones. A participant with a history of acute pancreatitis caused by gallstones may be included in the study if the participant has a cholecystectomy to resolve the problem
13. Chronic malabsorption, regardless of etiology
14. History of Crohn's disease, ulcerative colitis, or other inflammatory bowel disease
15. Impaired renal function at Screening defined as eGFR <30 mL/min/1.73m^2 per Chronic Kidney Disease Epidemiology Collaboration
16. Fasting serum triglyceride level of > 500 mg/dL at Screening
17. Documented diagnosis of heart failure and at Screening New York Heart Association Class III or IV
18. Within the past year before the Screening visit, any of the following: episode of acute coronary event, transient ischemic attack, stroke, peripheral arterial vascular disease leading to revascularization, or other significant cardiovascular event as determined by the Investigator
19. A supine BP after resting for 5 minutes >160 mm Hg systolic or >100 mm Hg diastolic. The measurement will be performed in triplicate and the average of all 3 measurements used to determine eligibility
20. An apical (brachial is acceptable) pulse rate >100 bpm. If elevated, the measurements may be repeated 2 more times and the average of all 3 measurements used to determine eligibility

21. Evidence of abnormality on the screening visit ECG deemed clinically significant by the Investigator, history of known arrhythmia or prolonged QT interval corrected using Fridericia's formula (QTcF) (males >450 ms; females >470 ms), or risk factors for torsade de pointes (including a family history of long QT syndrome or sudden cardiac death)
22. Liver enzyme results elevated >3.0× above the upper limit of normal (ULN) for aspartate transaminase (AST) or alanine transaminase (ALT), or >1.5× ULN for bilirubin (total, conjugated, or unconjugated) (except for cases of known Gilbert's Syndrome)
23. Documented diagnosis of cirrhosis
24. Presence or having sequelae of GI, liver, kidney, or other conditions known to interfere with the absorption, distribution, metabolism, or excretion of drugs
25. Any history of ileus
26. Positive test results for an active viral infection: severe acute respiratory syndrome coronavirus 2, human immunodeficiency virus (HIV)-1 or HIV-2 with positive antibodies, hepatitis B virus with a positive hepatitis B surface antigen (HBsAg), or hepatitis C virus (HCV) with positive antibodies and viral load at the Screening visit
27. Have a history of significant active or unstable major depressive disorder or other severe psychiatric disorder (e.g., schizophrenia, bipolar disorder, or other serious mood or anxiety disorder) within the last 2 years
28. Have a lifetime history of suicide attempt
29. Have a Patient Health Questionnaire-9 (PHQ-9) score of 15 or more prior to randomization
30. On the Columbia Suicide Severity Rating Scale (C-SSRS) during Screening (within the last 28 days):

    - A "yes" answer to Question 4 (Active Suicidal Ideation with Some Intent to Act, Without Specific Plan) on the "Suicidal Ideation" portion of the C-SSRS

    or

    - A "yes" answer to Question 5 (Active Suicidal Ideation with Specific Plan and Intent) on the "Suicidal Ideation" portion of the C-SSRS

    or

    - A "yes" answer to any of the suicide-related behaviors (actual attempt, interrupted attempt, aborted attempt, preparatory act, or behavior) on the "Suicidal Behavior" portion of the C-SSRS

    and

    - the ideation or behavior occurred

Prior/Concomitant Therapy

31. Use of medications intended to promote weight loss, within 6 months prior to Screening, including, but not limited to, the following:
    - Saxenda® (liraglutide 3.0 mg) or other GLP-1RA
    - Wegovy® or Ozempic® (semaglutide)
    - Xenical®/Alli® (orlistat)
    - Meridia® (sibutramine)
    - Acutrim® (phenylpropanolamine)
    - Sanorex® (mazindol)
    - Adipex® or Lomaira™ (phentermine)
    - Belviq® (lorcaserin)
    - Qsymia® (phentermine/topiramate combination)
    - Contrave® (naltrexone when combined with bupropion)
    - Zepbound™ or Mounjaro® (tirzepatide)
    - Other similar body weight loss medication, including over-the-counter medications (e.g., Alli)
32. Have current treatment with or history of treatment with (within 3 months prior to Screening) medications that may cause significant weight gain including, but not limited to, tricyclic antidepressants, atypical antipsychotics, and mood stabilizers. However, participants at a stable dose (greater than 6 months and with no expectation that the dose will change within the next year) and who are weight stable for the last 6 months on these medications may be included in the study.

    Examples of medications causing weight gain that must be at stable doses for at least 6 months and with no expectation of changing within the next year include:
    - imipramine
    - amitriptyline
    - mirtazapine
    - paroxetine
    - phenelzine
    - chlorpromazine
    - thioridazine
    - clozapine
    - olanzapine
    - valproic acid and its derivatives

- lithium

Note: Selective serotonin reuptake inhibitors other than paroxetine are permitted. Buproprion, when not combined with naltrexone is permitted.

33. Use of potent CYP3A4 inducers or moderate to potent CYP3A4 inhibitors, drugs that are P-gp or BCRP substrates, or drugs that are P-gp inducers or P-gp inhibitors. See Appendix 5 for details. CYP3A4 substrates are not excluded but concomitant medications that are sensitive CYP3A4 substrates may require monitoring with or without dose reductions, according to the individual respective product labeling, as summarized in Section 10.5 Appendix 5.
34. History of substance abuse within 12 weeks before Screening
35. Participants recovering from opiate abuse, taking methadone or buprenorphine/naloxone (Suboxone®)
36. Positive drugs of abuse screen (excluding marijuana [tetrahydrocannabinol (THC)]) before the first dose of study drug

Prior/Concurrent Clinical Study Experience

37. Are currently enrolled in any other clinical study or have participated in another clinical trial of an investigational drug or device targeting a weight reduction therapy within 60 days before the first study drug administration. At least 6 months before the first study drug administration if the clinical trial included a GLP-1RA

Other Exclusions

38. Pregnant, breastfeeding, or intends to become pregnant
39. Female of childbearing potential and not using an adequate contraceptive method (adequate contraceptive measures as required by local regulation or practice) for the duration of the trial and for ≥30 days following the last dose of study drug
40. Male not using adequate contraceptive method for the duration of the trial and for ≥90 days following the last dose of study drug
41. Consumption of grapefruit, tangelo, or Seville orange (or products containing grapefruit, tangelo, or Seville orange) within 10 days before the first administration of study drug and throughout the study
42. Demonstrated clinically significant allergic reactions (e.g., food, drug, or atopic reactions, asthmatic episodes) that required intervention, (e.g., emergency room visit, epinephrine administration) and which, in the opinion of the Investigator, would interfere with the participant’s ability to participate in the trial
43. Known hypersensitivity to any of the study drug ingredients

5.3. Lifestyle Considerations

5.3.1. Nutrition and Good Health Instructions

Participants will be instructed by a nutritionist/dietician or a trained site coordinator on how to manage nutritional needs and how to increase their activity. In general, the prescribed nutritional plan will encourage a low fat, high protein plan that will help reduce loss of muscle mass while decreasing the intake of high calorie foods with low nutritional benefit. Exercise regimens will also be recommended to increase energy utilization and maintain muscle mass. Detailed guidance for nutrition and exercise interventions will be provided in detail in the Study Procedural Manual.

5.3.2. Meals and Nutritional Restrictions

Consumption of grapefruit, tangelo, or Seville orange (or products containing grapefruit, tangelo, or Seville orange) is not permitted within 10 days before the first administration of study drug and throughout the study.

5.3.3. Caffeine and Tobacco

Caffeine-containing products (e.g., coffee, tea, chocolate, and caffeine-containing sodas, colas, etc.) may not be consumed within 2 hours before measuring vital signs and ECGs. Participants will be allowed to maintain their regular caffeine consumption throughout the study period (excluding the 2 hours before measuring vital signs and ECGs).

Participants must consume no more than 10 cigarettes or equivalent per day.

Participants must have a negative result in a serum or urine drugs of abuse test (excluding marijuana [THC]) at Screening and must abstain from recreational drugs throughout the study. Participants must not be in a drug recovery program that prescribes either methadone or buprenorphine/naloxone (Suboxone®).

5.4. Screen Failures

A screen failure occurs when a participant who has consented to participate in the clinical study is not subsequently assigned to study intervention/entered into the study. A minimal set of screen failure information is required to ensure transparent reporting of screen failure participants to meet the CONSORT publishing requirements and to respond to queries from regulatory authorities. Minimal information includes demography, screen failure details, eligibility criteria, and any SAE.

6. STUDY INTERVENTION(S) AND CONCOMITANT THERAPY

6.1. Study Intervention(s) Administered

Study interventions are all prespecified, investigational and non-investigational medicinal products intended to be administered to the study participants during the study conduct. Delegated study site personnel will supervise and instruct participants on the administration of the study intervention (i.e., investigational product [IP]). Participants will self-administer the GSBR-1290 or placebo tablets (Table 3) orally in the morning with noncarbonated room temperature water (approximately 240 mL), followed by a light breakfast (details of nutritional recommendations will be provided in the Study Procedural Manual). Orally administered study drugs must be swallowed whole and not chewed, divided, dissolved, or crushed. The exact dates and times of study drug administration (when administered in the clinic) will be recorded in the electronic Case Report Form (eCRF). The exact date and time of study drug administration at home will be recorded in the e-diary.

Table 3: Study Intervention(s) Administered

Intervention Name	GSBR-1290	Placebo
Type	Drug	Control
Dose formulation	Tablet	Tablet
Unit dose strength(s)	5 and 30 mg	Not applicable
Route of administration	Oral	Oral
Sourcing	GSBR-1290 tablets supplied by designated manufacturer/ distributor of Sponsor	Placebo tablets supplied by designated manufacturer/ distributor of Sponsor
Packaging and labeling	Study intervention will be provided in 75 mL oral solid pharmaceutical HDPE bottle containing 1 g desiccant enclosed with a 33 mm PP CRC (fitted with an aluminum foil induction seal). Each bottle will be labeled to conform with country requirements.	

Abbreviations: CRC=child-resistant caps; HDPE=high-density polyethylene; PP=polypropylene; QD=once daily.

Participants will receive QD doses of study drug for 252 consecutive days.

6.2. Dose Modification/Titration Adjustments

6.2.1. Dose Reduction

In the event that tolerability cannot be mitigated by any other means of nutritional adjustment, holistic or prescribed anti-emetic treatments, titration adjustments will be permitted upon documented discussion with the Sponsor, as a last resort to manage AEs at the current dose. Every effort should be made to avoid down-titration. Adjustments will include down titrating to a lower dose, and then titrating back up to the target dose. Education and coaching on tolerance strategies will be provided to enable as many participants as possible to attain their target dose. Recommendations will be detailed in a Study Procedural Manual.

In the event that a participant experiences AEs that preclude continuing at a prescribed dose, the participant may take a lower tolerated dose and should be rechallenged to take the next highest full dose in the morning after 1 or 2 days of introducing further nutritional counseling or anti-emetic/symptomatic therapy. Both the Investigator and SMR must be consulted/informed and communication documented prior to the down-titration and providing the up-titration plan. The participant should receive reinforcement of counseling on steps to improve tolerability. This should be delivered at every visit by the qualified study staff.

Following down-titration, participants should be up-titrated to the next dose level, with the goal of having participants achieve their target dose level, even if delayed, per Investigator discretion.

6.2.2. Dose Interruption/Hold

If the medication needs to be temporarily stopped, it is not desirable to hold dosing beyond 1 or 2 days in order to avoid losing tolerability gained from maintaining daily dosing. Both the Investigator and SMR must be consulted/informed and communication documented providing the up-titration plan.

If unacceptable tolerability symptoms re-occur or persist, the participant should continue in the study at the highest tolerable dose unless the Investigator and the SMR agree on an alternative appropriate approach. All participants must be able to tolerate a minimum dose of 30 mg/day to remain in the study, otherwise early termination will be initiated. Doses of 5 or 15 mg/QD are not considered maintenance doses, and participants who cannot tolerate these doses will be permanently discontinued from the study.

If the participant has been on a dose hold for >7 days, a new titration schema should be devised upon consultation with the SMR.

Date and reasons for study drug dose interruptions, reductions, and discontinuations will be captured in the Case Report Form.

6.2.3. Management of Participants with Gastrointestinal Symptoms

In the clinical studies conducted with GSBR-1290 to date, the most commonly reported TEAEs for participants receiving GSBR-1290 were GI-related events, including nausea, vomiting, constipation, and diarrhea. To mitigate GI symptoms and manage participants with GI AEs, the Investigator should do the following:

- Advise participants to eat smaller meals, e.g., having 4 or more smaller meals throughout the day instead of 3 large daily meals, and to stop eating when they are full.
- Advise participants that lower-fat meals and a bland meal plan might be better tolerated.
- Advise participants to eat slowly to allow time to sense fullness and to stay hydrated.
- Prescribe anti-emetic or antidiarrheal medication according to the Investigator judgment. This should be captured as concomitant medication in the eCRF.
- Advise participants that over time, their body can adapt to the study intervention, and the symptoms may abate.

- Other measures as appropriate which, in the opinion of the Investigator, may help with tolerability.
- A reduction in study drug for unrelenting tolerability issues should rarely be warranted and the process to be followed is described in Section 6.2 and will be outlined in the Study Procedural Manual.

6.3. Identification and Description of the Investigational Product

GSBR-1290 oral tablets at the dose strength of 5 and 30 mg will be used as the IP in this study. Tablets of matching composition and appearance to the IP will be administered as placebo.

6.4. Supply and Labeling of Investigational Product

GSBR-1290 tablets and placebo tablets will be supplied by a designated manufacturer/distributor of the Sponsor to the clinical sites. All IP material will be packaged in 75 mL oral solid pharmaceutical high-density polyethylene bottle containing 1 g desiccant enclosed with a 33 mm polypropylene child-resistant caps (fitted with an aluminum foil induction seal) with an appropriate label.

Based on available stability data, the GSBR-1290 tablets and placebo tablets are to be stored at controlled room temperature, 15°C to 25°C (59°F to 77°F).

Additional information on GSBR-1290 is provided in the Investigator's Brochure.

GSBR-1290 tablet and placebo tablet doses to be administered to a participant will be labeled according to current Good Manufacturing Practices for medicinal products. The detail on the labels will include, but not be limited to, the following:

- The notation- 'Caution: New Drug—Limited by Federal (or US) Law to Investigational Use.'
- Protocol number
- Name of Sponsor
- Route of administration
- Storage conditions

The final labels will be coded as appropriate to the study design and in compliance with this protocol.

Standard operating procedures will be followed for the receipt, handling, and accountability of the study drugs (GSBR-1290 tablets and placebo tablets). Study drug supplies will be stored securely under the appropriate conditions at the site pharmacy.

Detailed instructions for the labeling and supply of GSBR-1290 tablets and placebo tablets are described in the study Pharmacy Manual.

6.5. Preparation, Handling, Storage, and Accountability

The study drugs (GSBR-1290 tablets and placebo tablets) must be stored in a secure area with access limited to the Investigator and authorized staff at the specified storage temperature. Refer to the directions for use in the study Pharmacy Manual for additional storage requirements.

The Investigator (or delegate) must ensure that deliveries of study drug from the Sponsor (or designated supplier) are correctly received by a responsible person, that all receipts of shipments are recorded on the appropriate drug accountability forms prepared by the site pharmacy, and that the study drugs are stored in a secure area under the recommended storage conditions. It is also the responsibility of the Investigator (or delegate) to ensure that the integrity of packaging not be jeopardized before dispensing.

Only participants enrolled in the study may receive the study drugs in accordance with all applicable regulatory requirements. Only authorized and trained site staff may dispense and administer the study drugs.

Upon receiving the study drug from a trained staff member and being instructed how to take their daily dose, the participant will then take the first dose as instructed and record the dose in the appropriate accountability records. The site staff member will be responsible for dispensing study drug in accordance with the randomization schedule. The dispensing procedures are described in the Pharmacy Manual.

The site pharmacy or a study coordinator will dispense the study drug, and the clinical site staff will give the study drug only to participants included in this study following the procedures stated in this study protocol. Dispensation of study drugs will be recorded in the appropriate drug accountability records and the eCRF. Dispensation of study drug will be verified by a second staff member. Study participants will be instructed on dosing at each dispensation visit.

6.6. Assignment to Study Intervention

All study participants who sign an ICF at Screening will receive a unique sequential number (i.e., a Screening Number).

Participants who meet the study eligibility criteria will be assigned a randomization number on Day 1 that corresponds to a study treatment. The allocation to GSBR-1290 tablet or placebo tablet will be performed using a randomization algorithm and in accordance with the randomization schedule. To obtain balance across treatment groups, the randomization will be stratified by baseline BMI and sex. The stratification factors and strata within each factor are listed below:

1. Baseline BMI
    a. ≤35 kg/m^2
    b. >35 kg/m^2
2. Sex
    a. Male
    b. Female

No more than 10% of the participants with BMI <30 kg/m^2 and no more than 65% female participants will be randomized.

All eligible participants will be centrally assigned to a randomized study drug after all eligibility criteria have been verified and confirmed by the Investigator or designee.

6.7. Blinding and Breaking Code

This is a double-blind, placebo-controlled study. Blinding of study drug/placebo assignment is critical to the integrity of this clinical trial. Those blinded to study drug assignment include individuals from the Sponsor (and delegates) involved in day-to-day, study-related activities, the Investigator, clinical study personnel participating in participants' care or clinical evaluations, and the study participants. Blinded personnel must not make any effort to determine which study drug therapy is being administered. Some individuals from the Sponsor who are not involved in the daily operations of the study may be unblinded in order to evaluate clinical, tolerability data for discussions of PK parameters, and SAEs that may need to be reported expeditiously.

Blind-breaking instructions, and the unblinding process will be provided to investigative sites. It is expected that in the majority of cases, AEs can be properly managed without the need for unblinding. However, in the event of a medical emergency in which knowledge of an individual participant's intervention assignment is considered critical to the participant's well-being and management, the Investigator may unblind the intervention assignment for the participant in question. Unless the clinical situation warrants emergency unblinding, the Investigator is strongly advised to discuss options with the SMR or contract research organization (CRO) Medical Monitor (MM) prior to any unblinding. The Investigator should contact the SMR or CRO MM to discuss the participant and circumstances requiring the unblinding. The blind will be broken only for the specific participant under consideration. If the blind is broken for any reason and the Investigator was unable to contact the Sponsor or CRO MM before unblinding, the Investigator must notify the Sponsor and CRO MM as soon as possible, without revealing the participant's intervention assignment (unless important to the safety of participants remaining in the study). The Sponsor (and any Sponsor nominee, e.g., blinded Study Monitor[s]) will remain blinded to the participant's intervention assignment. Every effort will be made to restrict knowledge of the participant assignment to GSBR-1290 or placebo to the Investigator only. The Study Monitor must be informed promptly that the Investigator has been unblinded to a participant's intervention assignment. The participant may remain in the study under a single blind. The date and reason that the blind was broken must be recorded in the source documentation.

6.8. Study Intervention Compliance

All study drugs are for use only in this clinical study and should not be used for any other purpose.

The Investigator is responsible for study drug accountability, reconciliation, and record maintenance at the clinical site. In accordance with all applicable regulatory requirements, the Investigator or designated trained site staff must maintain study drug accountability records throughout the course of the study. This person will document the quantity of GSBR-1290 tablets or placebo tablets received by the site pharmacy and the amount dispensed to, administered to, and returned by participants, as applicable. The shipment/receipt records as well as records of dispensing, administration, and return will be verified and documented by a second member of the site staff. The Investigator (or designated trained site staff) is responsible for

assuring the retrieval of all remaining study drug supplies following administration to participants.

The study drug inventory and accountability/dispensing records must be available for inspection by the Study Monitor during the study. Used containers of the study drug will be retained and sequestered per participant and made available to the Study Monitor during reconciliation of study medication. Please see the Pharmacy Manual for further details.

When requested in writing by the Sponsor and following drug accountability and reconciliation, unused study drug supplies may be returned to the Sponsor or destroyed by the Investigator (or delegate) according to investigational site standard operating procedures, provided destruction does not expose humans to risks from the study drugs. Records shall be maintained of the return or destruction of the study drug materials. These records must show the identification and quantity of each unit returned or disposed of the method of destruction (taking into account the requirements of local law), and the person who returned or disposed of the study drug materials. Such records must be submitted to the Sponsor.

Pharmacokinetic Monitoring to Verify Compliance with Dosing Regimen:

To verify compliance with the study dosing regimen and adherence to the protocol requirements, random PK blood draws may be conducted on enrolled participants, to monitor the concentration of the study drug in the bloodstream. Based on model projections and existing data, further adherence assessments and follow-up may be conducted.

6.9. Continued Access to Study Intervention After the End of the Study

GSBR-1290 will not be made available to participants after conclusion of the study.

6.10. Treatment of Overdose

The maximum tolerated dose for GSBR-1290 has not yet been established. For this study, any dose of study intervention greater than the planned highest dose in this study within a 24-hour time period will be considered an overdose.

In the event of an overdose, the Investigator/treating physician should do the following:

- Evaluate the participant to determine, in consultation with the MM, if possible, whether study intervention should be interrupted or whether the dose should be reduced.
- Closely monitor the participant for any AE/SAE and laboratory abnormalities, as medically appropriate.
- Document the quantity of the excess dose as well as the duration of the overdose on eCRF.

There is no specific antidote for overdose with GSBR-1290. In the event of a suspected overdose, it is recommended that appropriate supportive clinical care should be instituted, as dictated by the participant’s clinical status.

6.11. Prior and Concomitant Therapy

Prior medication use as of the Screening visit will be recorded at Screening and Day 1. Concomitant medication (all medication/treatment/therapy taken between the date of first study drug administration and the end of the follow-up period inclusive) use will be recorded at each clinic visit (Section 1.3).

Use of any prescription or over-the-counter medications (including herbal products, diet aids, and hormone supplements, with the exception of contraception) are prohibited within 10 days or 5-times apparent terminal elimination half-life values of the medication (whichever is longer) before the first study drug administration and throughout the study, with the exception of medications used to manage AEs at the discretion of the Investigator or medically certified delegate, including, but not limited to, the examples described below.

- For common ailments, occasional use of paracetamol/acetaminophen (doses of 500 mg up to every 6 hours or 2 g per day maximum for ≤3 consecutive days), topical hydrocortisone for skin irritation, and contraceptives for women of childbearing potential (WOCBP).
- Treatment of GI-related symptoms or symptoms due to respiratory viral infections may be allowed (including, but not limited to, antiemetics, analgesics, therapies to treat cough, etc.). The Investigator should consult with the Sponsor on a case-by-case basis.
- While participating in the study, participants will not be allowed the concomitant use moderate to potent inducers or inhibitors of CYP3A4 (Section 10.5) or P-gp inducers or inhibitors due to their potential to increase/decrease GSBR-1290 exposure (Section 10.5). If an acute treatment with a moderate CYP3A4 inhibitor is required post study start and there is no alternative treatment, the Investigator should contact the Sponsor for assessment based on the GSBR-1290 doses being administered (co-administration at low doses could be feasible). Chronic concomitant treatment with moderate CYP3A4 inducers or inhibitors is not allowed. Co-administration of GSBR-1290 with P-gp or BCRP substrates is also not allowed.

7. DISCONTINUATION OF STUDY INTERVENTION AND PARTICIPANT DISCONTINUATION/WITHDRAWAL

Discontinuation of specific sites or of the study as a whole are detailed in Section 10.1.10.3, Appendix 1.

7.1. Discontinuation of Study Intervention (Investigational Product)

In rare instances, it may be necessary for a participant to permanently discontinue study intervention. If study intervention is permanently discontinued, the participant should return to the clinic within 7(±3) days from the last dose for the early termination visit assessments. The date and reason for discontinuation of study intervention will be captured in the eCRF. See the SoA for details on information to be collected at the time of discontinuation of study intervention and follow-up.

Possible reasons leading to permanent discontinuation of study drug include the following:

- The participant requested to discontinue the study drug.
- The participant has discontinued the study drug on their own and refuses to restart the study drug.
- Any nonstudy medication for weight loss is given for more than 1 week.
- Any TEAE, SAE, or clinically significant laboratory value assessed as possibly or probably related to study drug that the Investigator deems to be medically relevant and may potentially endanger the welfare of the participant.
- Pregnancy.
- Significant noncompliance with the protocol.
- Inability to tolerate 5 mg or 15 mg QD dosing.
- If the participant develops any exclusion criteria during the course of the study, the Investigator should consult with the Sponsor to determine whether discontinuation of study drug is necessary.
- The Investigator decides that the participant should be discontinued from the study drug.

7.1.1. Liver Chemistry Stopping Criteria

The study drug should be interrupted or discontinued if one of more of the following conditions occur:

- ALT or AST >8× ULN
- ALT or AST >5× ULN for more than 2 weeks
- ALT or AST >3× ULN and serum total bilirubin (TBL) >2× ULN or international normalized ratio (INR) >1.5 × ULN without elevated serum alkaline phosphatase (ALP), and no other disease or condition can be found to explain the abnormalities

- ALT or AST >3× ULN with the appearance of fatigue, nausea, vomiting, right upper quadrant pain or tenderness, fever, rash, and/or eosinophilia (>5%)

See Section 10.6, Appendix 6 for details on suggested actions and follow-up regarding liver safety.

7.1.2. QTc Stopping Criteria

If the mean QTcF of an ECG is >500 ms or there is a >60 ms change from the Baseline predose ECG triplicate, then further dosing with study drug should be withheld until the participant can be assessed. Further assessment will include repeat triplicate ECGs, physical examination, and assessment of symptoms, after which the Investigator or qualified designee in consultation with the SMR will determine if the participant can continue in the study.

7.2. Participant Discontinuation/Withdrawal From the Study

Participants will be advised that they are free to withdraw from the study at any time for any reason or, if necessary, the Investigator (or medically certified delegate) may discontinue a participant from the study to protect the participant's health.

A participant may voluntarily withdraw or be withdrawn from the study for reasons, including, but not limited to, the following:

- The need to take medication that may interfere with study measurements
- Intolerable/unacceptable AEs
- Noncompliance of the participant with the protocol
- Discontinuing study drug and unwilling to be rechallenged
- Withdrawal of consent
- If, in the Investigator's judgment, it is in the participant's best interest

The Sponsor will be notified as soon as possible of any participant withdrawals. The date and reasons for withdrawal will be recorded in the eCRF and included in the final clinical study report (CSR), along with any AEs and necessary medical treatment.

Any participant who prematurely discontinues study drug should have all scheduled safety assessments performed at the next study visit, which will be the EOS visit, after which they will be withdrawn from the study. If a participant withdraws from the study, an attempt should be made to collect all remaining safety and PK samples scheduled for that day.

In the event that a participant is discontinued from the study due to an SAE, the Investigator or medically certified delegate will evaluate the urgency of the event. If the situation warrants, the Investigator, or medically certified delegate, will take appropriate diagnostic and therapeutic measures and attempt to notify the SMR. If the situation is not an immediate emergency, the Investigator, or medically certified delegate, at the clinical study site will attempt to contact the SMR for consultation. No medical help, diagnosis, or advice will be withheld from the participant due to an inability to contact the SMR. The participant will be encouraged to remain available for follow-up medical monitoring.

7.3. Lost to Follow-up

A participant will be considered lost to follow-up if the participant repeatedly fails to return for scheduled visits and is unable to be contacted by the study site.

The following actions must be taken if a participant fails to return to the clinic for a required study visit:

- The site must attempt to contact the participant and reschedule the missed visit as soon as possible, counsel the participant on the importance of maintaining the assigned visit schedule and ascertain whether the participant wishes to and/or should continue in the study.
- Before a participant is deemed lost to follow-up, the Investigator or medically certified delegate must make every effort to regain contact with the participant (when possible, telephone calls, emails, and if necessary, a certified letter to the participant's last known mailing address or local equivalent methods). These contact attempts should be documented in the participant's medical record.
- Should the participant continue to be unreachable, the participant will be considered to have withdrawn from the study and lost to follow-up.
- Site personnel, or an independent third party, will attempt to collect the vital status of the participant within legal and ethical boundaries for all participants randomized, including those who did not get study intervention. Public sources may be searched for vital status information. If vital status is determined as deceased, this will be documented, and the participant will not be considered lost to follow-up. Sponsor personnel will not be involved in any attempts to collect vital status information.

8. STUDY ASSESSMENTS AND PROCEDURES

All study assessments to be performed and the timing of each assessment are presented in the SoAs (Section 1.3). Clinical staff are required to perform assessments at the prespecified timepoints within the time windows indicated in this protocol. Actual times of procedures for each participant may vary depending on scheduling and will be recorded in the eCRF. In the event of multiple procedures scheduled at the same time, the order, where possible, will be as described in Figure 2.

Figure 2: Order of Procedures

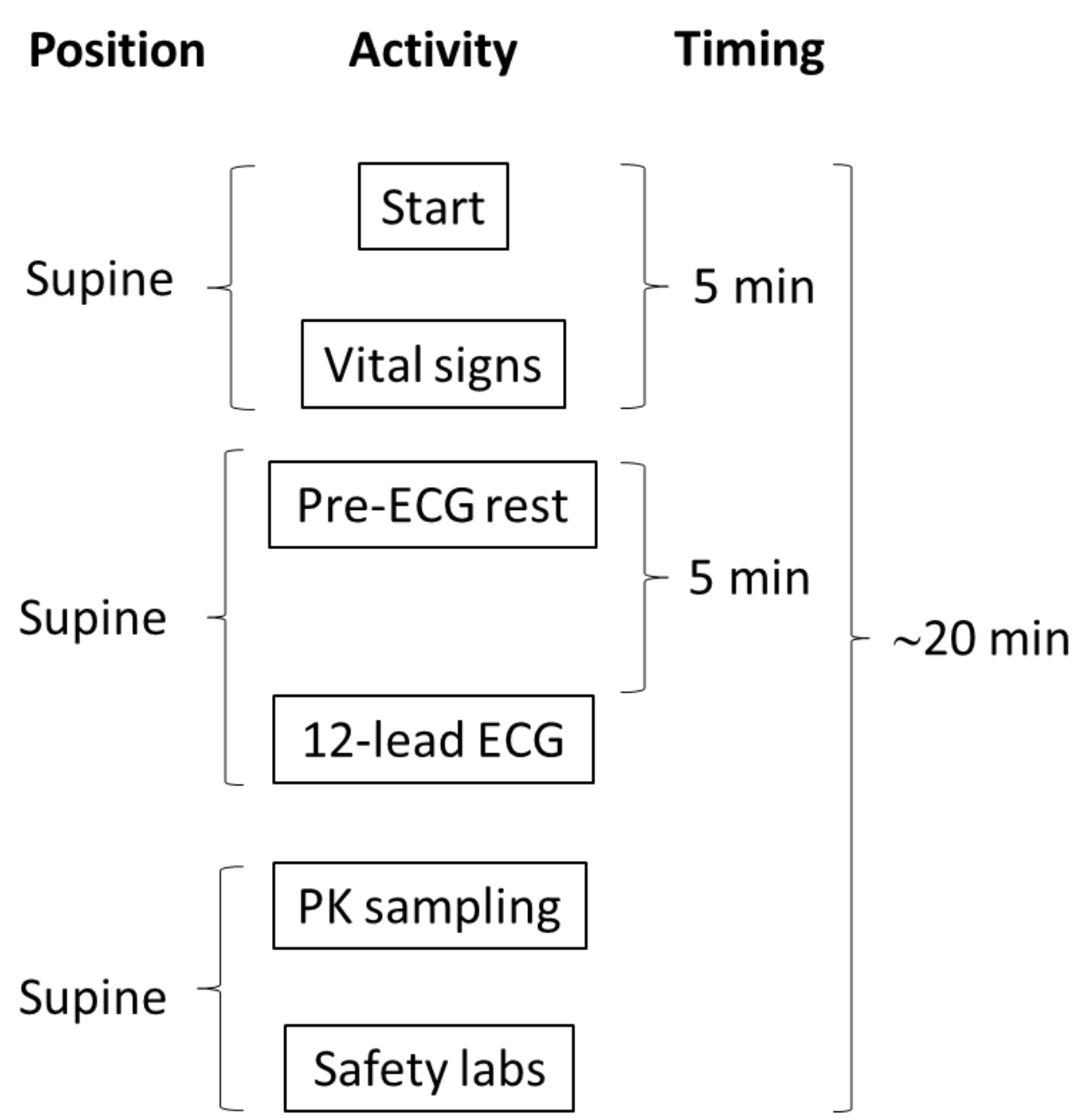

Abbreviations: ECG = electrocardiogram; PK = pharmacokinetic

8.1. Baseline Procedures

Before performing any study-related procedures, the Investigator (or delegate) will obtain written informed consent from participants.

Clinical staff is required to perform all assessments at the prespecified timepoints within the time windows indicated in the SoAs (Section 1.3). Baseline for body weight, vital signs, blood and urine clinical laboratory parameters, and ECG parameter data will be defined as the last assessment done before the first administration of study drug for each participant.

Demographic data, including sex, age, race, and ethnicity, will be recorded.

Body height (centimeters) using a stadiometer and weight (kilograms) using certified calibrated weight scales in hospital gowns without shoes will be measured at baseline and postbaseline visits as detailed below.

Measuring Height (to be done in triplicate)

- Step 1: Ask the participant to remove their footwear and any headgear (light headgear worn for religious reasons can remain, but this should be worn by the participant at every clinic visit when height is measured).
- Step 2: Ask the participant to stand on the height measuring board (stadiometer) or against a wall with their feet together and their knees straight with their heels against the backboard, the stadiometer, or the wall.
- Step 3: Ask the participant to look straight ahead without tilting their head up.
- Step 4: Ask the participant to breathe in and stand tall. Measure and record the participant's height in centimeters to 1 decimal place.

Measuring Weight (to be measured in triplicate)

- Body weight measurements should be done in a consistent manner using a certified calibrated electronic scale capable of measuring weight in kilograms to 1 decimal place.
- All weights for a given participant should be measured using the same scale, whenever possible, at approximately the same time in the morning after evacuation of bladder contents.
- Body weight must be measured in fasting state. If the participant is not fasting, the participant should be called in for a new visit within the visit window to have the fasting body weight measured.
- Step 1: Ask the participant to empty their pockets, remove their footwear, outerwear (coat, jacket, etc.), and any headgear (light headgear worn for religious reasons can remain, but this should be worn by the participant at every clinic visit when weight is measured). Participant should be wearing light clothing (no sweaters, coats, jackets, etc.) or a hospital gown.
- Step 2: Make sure the scale is placed on a firm, flat, even surface (not on carpet, on a sloping surface, or a rough, uneven surface).
- Step 3: Ask the participant to step onto the scale with 1 foot on each side of the scale.
- Step 4: Ask the participant to stand still with arms by sides and then record weight in kilograms to the nearest one-tenth kilogram.

Waist circumference will also be measured at the timepoints as indicated in the SoAs (Section 1.3). Proper measurement of waist circumference requires identifying the iliac crests and placing a measuring tape along the top of the iliac crests, parallel to the ground and around the central abdomen. Three measurements should be collected, placing the tape measure each time.

Blood pressure measurements should be performed in triplicate after the participant has been seated for at least 5 minutes. A proper sized cuff should be placed around the left upper arm. Three measurements should be taken, 5 minutes apart and recorded.

Medical history, including pre-existing conditions, allergies, prior significant illnesses, and surgical history, will be recorded. Medical history will also include smoking history.

Any nonstudy concomitant medication or therapy that is taken by or administered to a participant during the study will be recorded. The medication/therapy name as well as dose, route of administration, indication, and dates of use will be noted, as applicable.

8.2. Participant-reported Outcomes

8.2.1. Electronic Diary

An electronic diary (e-diary) will be used for the purpose of recording and transmitting data on participant-reported outcomes. Symptoms will be reported one week after each dose titration step and information regarding medication taking (including date and time of dosing) will be recorded daily. Upon completion of the e-diary, data will be available for periodic review by the sites, CRO, and Sponsor through the web-based electronic data and control system and summary reports.

A study-specific and secure website will be used by study staff to access and review electronic participant diary data and data summaries at each visit but could be more frequent at Investigator discretion, if needed. The permissions for enabling or preventing such review are set so that only authorized site personnel and e-diary vendor staff can see individually identifying health information, as defined in regulations concerning the protection of privacy for trial participants as well as the individuals who serve as study staff. All users of the website will have undergone identity certification.

Detailed directions for the administration of the e-diary are provided in the electronic patient reported outcome ePRO Manual. Symptoms in the diary should be reviewed by the Investigator for clinical significance and possible AE reporting, but not every symptom in the diary needs to be recorded as an AE. More severe symptoms have greater likelihood of being clinically significant and therefore reported as an AE, but less severe symptoms should still be reviewed for AE reporting at the discretion of the Investigator.

8.2.2. Impact of Weight on Quality of Life-Lite CT, Short Form-36 Acute Version, and Hunger and Satiety Questionnaires

Participants will complete an Impact of Weight on Quality of Life-Lite Clinical Trials Version (IWQOL-Lite CT) and Short Form 36 health survey (SF-36) acute version health survey questionnaires assessing weight-related physical and psychosocial function as indicated in the SoA (Section 1.3). Participants will also complete a hunger and satiety evaluation at each clinic visit as indicated in the SoA (Section 1.3). For additional information on the IWQOL-Lite CT assessment, please refer to the following reference (Kolotkin, et al., 2019). For more information on the SF-36 assessment, please refer to the following reference: (Maruish, 2011).

8.3. Safety Assessments

Safety assessments will include clinical assessments, such as medical history, concomitant medication use, evaluation of AEs and TEAEs, weight, physical examination, vital signs (systolic and diastolic BP, HR, respiratory rate [RR], and temperature), 12-lead ECGs in triplicate, mental health questionnaires (C-SSRS and PHQ-9), pregnancy testing if indicated, and

laboratory test results. Planned timepoints for all safety assessments are provided in the SoAs (Section 1.3).

8.3.1. Physical Examinations

Full physical examinations will include, at a minimum, assessment of the following: general appearance, head, ears, eyes, nose and throat, neck (including thyroid and lymph nodes), respiratory system, cardiovascular system, GI system, neurological condition, musculoskeletal system, dermatological system, and any other focused assessments suggested by the presence of specific symptoms.

Symptom-directed physical examinations will be performed if deemed required by a medically certified delegate member and will include focused assessments suggested by the presence of specific symptoms.

All physical examinations will be conducted by a licensed Investigator or medically certified delegate or licensed nurse practitioner within the time windows specified in the SoAs (Section 1.3). Physical examinations may be performed at various unscheduled timepoints if deemed necessary by the Investigator or medically certified delegate. All clinically significant findings post dose will be recorded as AEs.

8.3.2. Vital Signs

Vital signs assessments will include systolic and diastolic BP, HR, RR, and temperature. Participants should be resting in a supine position for ≥ 5 minutes before and during vital signs measurements. Predose vital signs assessments should be performed within 1 hour before dosing.

Abnormal vital signs assessments that are NOT clinically significant may be repeated at the Investigator’s discretion. Abnormal vital signs assessments that ARE clinically significant will be repeated. All clinically significant findings will be recorded as AEs.

8.3.3. Electrocardiograms

Twelve-lead ECGs in triplicate (including, but not limited to, the measurements of ventricular HR, PR interval, QRS duration, QT interval, and QTcF) will be performed at the timepoints and windows indicated in the SoAs (Section 1.3). Note that during PK sample collections, ECGs will need to be collected in triplicate with each indicated PK sample and should be within 15 minutes before the blood draw. At each protocol scheduled timepoint, ECGs will be performed for safety evaluation. Each ECG will be conducted after the participant has been supine for ≥5 minutes in a quiet setting without distractions (e.g., television, cell phones). ECGs will be interpreted, signed, and dated by the Investigator or medically certified delegate. The ECGs will be classified as normal, abnormal not clinically significant, or abnormal clinically significant. All clinically significant findings will be recorded as AEs and may also be centrally evaluated by the vendor if necessary.

Abnormal ECGs that are NOT clinically significant may be repeated at the Investigator’s discretion. Abnormal ECGs that ARE clinically significant will be repeated. In the case of evident bad quality (e.g., muscle tremor) of the tracing, an ECG will be repeated. If the QTcF values from the ECG shows a result above the threshold (i.e., ≥500 msec), then a single 12-lead ECG in triplicate must be repeated at least hourly until QTcF values from 2 successive ECGs fall

below the threshold. If QTcF values remain elevated after 2 successive ECGs (QTcF intervals do not return to < 500 msec or to < 60 msec above baseline), a cardiologist must be consulted. Participants under those circumstances should not receive drugs known to prolong QTc interval (e.g., erythromycin, fluconazole, nelfinavir, chloroquine). If the participant has been taking ondansetron (Zofran®), this should also be noted.

All ECG tracings will be kept as part of the participant's permanent trial file at the site. Digital recordings will be analyzed and stored at a central ECG laboratory.

8.3.4. Clinical Laboratory Tests

A full list of clinical laboratory tests to be performed for this study is described in Section 10.2, Appendix 2. The actual date and times of sample collection must be recorded in source documents. Instructions for collection, handling, and shipment are found in the Laboratory Manual.

In the event of an unexplained, clinically significant abnormal laboratory test result, the test should be repeated and followed up until it has returned to the normal range and/or an adequate explanation of the abnormality is found. Abnormal laboratory test results that are not clinically significant will be repeated at the Investigator's discretion. All clinically significant findings will be recorded as AEs (Section 8.4 and Section 10.3.1).

8.3.4.1. Clinical Laboratory Safety Tests

Blood samples for hematology, serum chemistry, and coagulation as well as urine samples for urinalysis will be collected at selected timepoints throughout the study as defined in the SoAs (Section 1.3). All samples should be collected within the timepoints indicated in the SoAs.

All clinical laboratory safety assessments will be assessed by a central laboratory using that laboratory's normal ranges. The Investigator must review the laboratory report and document this review. All out of range (abnormal) laboratory values must be evaluated and commented on by the Investigator for clinical significance. Laboratory safety test results will be classified as normal, abnormal not clinically significant, or abnormal clinically significant. All clinically significant findings will be recorded as AEs (Section 8.4 and Section 10.3.1).

8.3.4.2. Other Clinical Laboratory Tests

Other clinical laboratory tests will be assessed by a central laboratory using that laboratory's normal ranges. The Investigator must review the laboratory report and document this review.

Viral serology: HIV-1, HIV-2, HbsAg, and HCV antibody and viral load testing will be performed at the Screening visit. Participants will be counseled if any tests return a positive result. Participants who return a positive test result for HbsAg, HCV, or HIV at the screening visit will not be eligible to participate in the study.

Coronavirus disease 2019 (COVID-19) test: A COVID-19 test is performed during Screening and at check-in on Day 1 only if clinically indicated or per site policy; an antigen test is performed first and then polymerase chain reaction test for confirmation if clinically indicated. Participants with a positive COVID-19 result at Screening or on Day 1 will not be allowed to continue in the study. Participants who test positive for COVID-19 during participation in the

study will be considered on a case-by-case basis for the best course of action per Investigator medical judgment.

Drugs of abuse: Serum or urine drugs of abuse screen will be performed during Screening, on Day 1, and as indicated in the SoAs (Section 1.3).

8.3.5. Pregnancy Testing

Women of child-bearing potential will have a serum human chorionic gonadotropin (hCG) pregnancy test at Screening to determine their eligibility for participation in this study. A urine hCG pregnancy test also will be conducted on Day 1, at intervals throughout the study, and at the EOS/Early Termination visit to confirm no exposure in utero. If a urine test is positive or inconclusive, pregnancy will be confirmed by a serum test.

During their participation in this study, participants must be instructed to notify the clinical site if they or their partner becomes pregnant.

Serum follicle-stimulating hormone levels will be measured at screening in postmenopausal women ≤55 years of age to confirm postmenopausal status.

8.3.6. PHQ-9 and C-SSRS Questionnaires

Participants will complete a PHQ-9 and C-SSRS health questionnaire as indicated in the SoAs (Section 1.3). The Investigator will refer a participant to a Mental Health Professional or a Qualified Primary Care Professional if a participant has any of the following:

- A PHQ-9 score of ≥ 10
- Any suicidal behavior
- Any suicidal ideation of type 4 or 5 on the C-SSRS

In addition, the IDMC will review the aggregate data throughout the study. If a participant’s psychiatric disorder can be adequately treated with psychotherapy and/or pharmacotherapy, then the participant, at the discretion of the Mental Health Professional or Qualified Primary Care Provider, can be continued in the trial on randomized therapy.

For additional information on the PHQ-9 assessment, please refer to the following reference: (Kroenke, et al., 2001). For additional information on the C-SSRS assessment, please refer to the following reference: (Project, 2016).

8.4. Adverse Events, Serious Adverse Events, and Other Safety Reporting

8.4.1. Adverse Events and Serious Adverse Events

An AE is any untoward medical occurrence in a clinical study participant that is administered a pharmaceutical product and does not necessarily have to have a causal relationship with this treatment. SAE is any AE that results in death, is life-threatening, requires inpatient hospitalization, results in persistent or significant disability/incapacity, and/or other important medical event. SAEs must be notified to Sponsor within 24 hours. Please refer to Section 10.3 for definition and reporting requirements.

8.4.2. Adverse Events of Special Interest

The following AEs are defined as adverse events of special interest (AESIs): AESIs of major adverse cardiovascular event (MACE), heart failure syndrome, acute pancreatitis, hypoglycemia, thyroid malignancies, supraventricular arrhythmia and cardiac conductive disorders, hepatobiliary disorders, severe GI AEs, acute renal events, depressive disorder and/or suicide ideation, hypersensitivity, and C-cell hyperplasia. All AESIs should be captured on the AE Case Report Form and followed until resolution, stabilization, or the participant is lost to follow-up (also see Section 7.3).

8.4.2.1. Major Adverse Cardiovascular Events

The MACE of special interest include the following:

- Cardiac death
- Acute coronary syndrome
- Nonfatal stroke

Any event of MACE will be sent for adjudication (Section 8.4.4).

8.4.2.2. Heart Failure Syndrome

Criteria for heart failure syndrome include decreased functionality as defined by New York Heart Association, lung congestion and peripheral edema with elevated N-terminal pro-brain natriuretic peptide >400, and echocardiogram demonstrating wall motion findings consistent with heart failure, regardless of ejection fraction.

Any event of (suspected) heart failure syndrome will be sent for adjudication (Section 8.4.4).

8.4.2.3. Acute Pancreatitis

The diagnosis of acute pancreatitis requires 2 of the following 3 criteria:

- Abdominal pain consistent with acute pancreatitis (acute onset, severe, epigastric pain may radiate to the back)
- Serum amylase and/or lipase ≥ 3× ULN
- Characteristic findings of acute pancreatitis on computed tomography and/or magnetic resonance imaging

For any events of suspected acute pancreatitis, appropriate laboratory tests and/or imaging study should be performed. GSBR-1290 should be discontinued for any confirmed acute pancreatitis. Any suspected or confirmed pancreatitis should be managed at the Investigator’s discretion.

Any event of (suspected) acute pancreatitis will be sent for adjudication (Section 8.4.4).

8.4.2.4. Hypoglycemia

Blood glucose is monitored by laboratory tests. Hypoglycemia is defined as blood glucose level ≤ 70 mg/dL (3.9 mmol/L).

Occurrence and risk for hypoglycemia should be reviewed at every visit and investigated as indicated. Any episode of hypoglycemia should be recorded in the eCRF. Hypoglycemia events are classified as 3 levels:

- Level 1: Glucose <70 mg/dL (3.9 mmol/L) and ≥54 mg/dL (3.0 mmol/L)
- Level 2: Glucose <54 mg/dL (3.0 mmol/L)
- Level 3: A severe event characterized by altered mental and/or physical status requiring assistance for treatment of hypoglycemia

Any events of level 3 hypoglycemia should be reported as an SAE. The hypoglycemia events should be managed at the Investigator's discretion.

8.4.2.5. Thyroid Malignancies and C-cell Hyperplasia

The C-cell function will be monitored by calcitonin. Participants with serum calcitonin level ≥ 20 ng/L at screening will be excluded, and GSBR-1290 will be discontinued for any participant who develops serum calcitonin level ≥ 20 ng/L (Section 7.1).

Any event of (suspected) thyroid malignancies and C-cell hyperplasia will be sent for adjudication (Section 8.4.4).

8.4.2.6. Supraventricular Arrythmias and Cardiac Conductive Disorders

Defined as ECG findings of supraventricular arrythmias and/or cardiac conductive disorders. For any suspected supraventricular arrythmias and cardiac conductive disorders, 12-lead ECG should be performed and/or additional ECG or diagnostic tests should be performed at the Investigator's discretion.

8.4.2.7. Hepatobiliary Disorders

Liver function (Section 7.1.1) and potential drug-induced liver injury (DILI) (Section 10.6) should be closely monitored and managed.

Any events or suspected events related to hepatobiliary system, including gallbladder diseases, should be evaluated and additional diagnostic tests should be performed at the Investigator's discretion.

Any event of (suspected) cholecystitis demonstrated on ultrasound with or without cholelithiasis will be sent for adjudication (Section 8.4.4).

8.4.2.8. Severe Gastrointestinal Adverse Events

Severe GI AEs are defined as any AEs of GI system with severity of National Cancer Institute Common Terminology Criteria for Adverse Events (NCI-CTCAE) Grade 3 and above.

8.4.2.9. Acute Renal Events

Renal events will be assessed based on laboratory tests and any AEs/SAEs suggestive of new and/or progression of existing renal diseases. Participants and Investigators are advised to report and manage severe nausea, vomiting, and/or dehydration.

8.4.2.10. Depressive Disorder and/or Suicidal Ideation

Any AE of depressive disorder or suicidal ideation are considered as AESI.

8.4.2.11. Hypersensitivity

All allergic or hypersensitivity reactions should be reported as AE or SAE if serious criterion is met. GSBR-1290 should be temporarily held in the event of suspected severe or serious allergic reactions. The study drug can be restarted if agreed by both Investigator and SMR.

8.4.3. Investigator-reported Outcomes

The definitions of AEs and SAEs can be found in Section 10.3, Appendix 3.

The Investigator and any medically certified delegates are responsible for detecting, documenting, recording, and reporting (as appropriate) events that meet the definition of an AE or SAE and remain responsible for following up of AEs that are serious, considered related to the study intervention, or that caused the participant to discontinue study drug or withdraw from the study (Section 7). This includes events reported by the participant (or, when appropriate, by a caregiver, surrogate, or the participant's legally authorized representative).

The method of recording, evaluating, and assessing causality of AEs and SAEs and the procedures for completing and transmitting SAE reports are provided in Section 10.3, Appendix 3.

8.4.3.1. Time Period and Frequency for Collecting Adverse Event and Serious Adverse Event Information

Detection and recording of study-related AEs and SAEs starts from the signing of the consent form until completion of the last study-related procedure (including follow-up for safety assessments). Detection and recording of TEAEs will start at the time of the first dose of study drug and continue until the last study procedure (Section 1.3).

Any pre-existing conditions or signs and/or symptoms present in a participant before any involvement in the study (i.e., before first dose of study drug) should be recorded as medical history.

In addition, any change in health status, which is reported after informed consent but started before informed consent by the participant, will be documented as medical history. Any worsening of a pre-existing condition that occurs following informed consent will be recorded as an AE.

Any AE reported or observed after the start of dosing with any study treatment until completion of the last study-related procedure (includes follow-up for safety assessments) will be recorded as a TEAE.

A poststudy AE/SAE is defined as any event that occurs outside of the nominal AE/SAE study detection period. Investigators are not obligated to actively seek AEs/SAEs in former study participants. However, if the Investigator learns of any SAE, including a death, at any time after a participant has completed the study and he/she considers the event reasonably related to the study treatment, the Investigator will promptly notify the Sponsor.

8.4.3.2. Method of Detecting Adverse Events and Serious Adverse Events

The Investigator (or medically certified delegate) is responsible for detection, recording, and reporting of events that meet the criteria and definition of AEs. As a consistent method of soliciting AEs, the participant shall be asked a nonleading question such as: "How do you feel?".

8.4.3.3. Follow-up of Adverse Events and Serious Adverse Events

Following initial observation of an AE/SAE, the Investigator is required to proactively follow the progress of the relevant participant. Any information obtained in relation to the status of the AE/SAE and the condition of the participant must be appropriately documented in the eCRF and in a follow-up SAE Form where required.

All AEs and SAEs documented at a previous visit/contact that are designated as ongoing will be reviewed at subsequent visits/contacts. AEs/SAEs that have not resolved by EOS, or that have not resolved upon discontinuation of the participant's involvement in the study, must be followed until any of the following occurs:

- The event resolves
- The event stabilizes
- The event returns to baseline, if a baseline value is available
- The event can be attributed to agents other than the study drug or to factors unrelated to study conduct
- It becomes unlikely that any additional information can be obtained (participant or health care practitioner refusal to provide additional information, lost to follow-up after demonstration of due diligence with follow-up efforts)

8.4.3.4. Pregnancy

In instances of pregnancies or suspected pregnancies identified or reported for any female participant (or male participant's female partner), including a positive pregnancy test regardless of age, following administration of study treatment, the pregnant female participant (or the male participant's female partner) will be advised to notify her healthcare provider and the study Investigator. Study drug administration will be discontinued immediately in the event of a reported (or suspected) pregnancy in a female participant.

Pregnancies should be documented on the Pregnancy Form and reported in the same manner as an SAE. The pregnancy will be followed up by the Sponsor (or its representative) through to the expected outcome. At the conclusion of the pregnancy, the outcome should be documented on the exposure in utero report if it is a normal birth (i.e., no SAE for mother or offspring). In the event of an SAE for either mother or offspring, the outcome must be documented on the SAE Form and transmitted within 24 hours according to the Safety Monitoring Plan in place for this study.

Informed consent will be sought from the pregnant female participant (or male participant's female partner) in order to allow the Investigator to access and review relevant medical records to conduct follow-up throughout the gestational period and on the infant following delivery. The Investigator shall follow-up newborn infants that have been exposed to IP in utero for a

minimum of 12 months. Upon discovery of any congenital anomalies (or neonatal deaths) the Investigator shall submit a follow-up report to the Sponsor or designee Officer using the Pregnancy Form, including information regarding the status of the newborn. A miscarriage or abortion shall also be reported by the Investigator to the Sponsor or designee using the Pregnancy Form.

8.4.4. Adjudication Committee for Selected Adverse Events

An independent external AC will be established to perform ongoing adjudication of selected types of events in randomized participants in a blinded manner. The AC is composed of permanent members covering all required medical specialties. The AC will be totally independent and will have no authorizations to impact trial conduct, trial protocol, or amendments.

The AC will evaluate events sent for adjudication using predefined definitions and guidelines detailed in the AC Charter.

Events for adjudication

MACEs (3-point MACE, including acute coronary syndrome, nonfatal stroke, and cardiovascular death), acute pancreatitis, thyroid cancer, and cholecystitis will be adjudicated, defined as following:

- Death due to cardiovascular cause
- Acute coronary syndrome to include unstable angina, defined as chest pain at rest, MI, which includes ST-elevated myocardial infarction (STEMI) and nonSTEMI, and cardiovascular death, which includes sudden death witnessed and unwitnessed
- Stroke, hemorrhagic and ischemic, transient ischemic attacks, which include central neurologic deficits lasting up to 24 hours without sequela, and ministrokes with minor neurologic deficit lasting more than 24 hours with some permanent damage
- Heart failure syndrome, with decreased functionality as defined by New York Heart Association, lung congestion and peripheral edema with elevated N-terminal pro-brain natriuretic peptide >400, echocardiogram demonstrating wall motion findings consistent with heart failure, regardless of ejection fraction.
- Pancreatitis defined by mid abdominal and mid back pain with elevations in lipase and amylase and elevated white blood cell and platelet counts, with or without cholelithiasis
- Thyroid cancer based on biopsy pathology
- Cholecystitis demonstrated on ultrasound with or without cholelithiasis

Sources of events for adjudication include the following:

- Investigator-reported events for adjudication: When reporting AEs, the Investigator must send the appropriate AE category based on predefined criteria.
- AC-identified events: When reviewing documents of any event for adjudication, the AC can identify additional events in scope for adjudication that were not initially reported by the Investigator. In these instances, the Investigator will be notified of the

newly identified event and has the option to report the AC-identified event. Regardless of whether the Investigator decides to report the event, it will undergo adjudication. The AC may require the Investigator to provide additional information and/or the Sponsor to search perform database search to identify potential events for adjudication.

If new information becomes available for an event sent for adjudication, it is the responsibility of the Investigator to ensure the new information is uploaded to the system. The adjudication outcome will be reported back to the Investigator.

Further information about this AC is provided in a charter, including a description of the scope of the committee's responsibilities, the process and definitions to be utilized by the committee for adjudication, and the communication plan, including timelines.

8.4.5. Independent Data Monitoring Committee

In addition to routine trial monitoring, an external IDMC will monitor the safety data from this trial. The IDMC will have access to unblinded data as needed.

Further information about the IDMC is provided in the IDMC charter, including a description of the scope of the committee's composition, responsibilities and governance, the process to be utilized by the committee, and the communication plan, including timelines.

8.5. Pharmacokinetics

Blood samples will be collected for assessment of plasma concentrations of GSBR-1290 and derived PK parameters. Clinical staff are required to collect each blood PK sample at the scheduled timepoints within the windows indicated (Section 1.3). The actual date and time (as hh:mm) of each blood sample collection will be recorded in the eCRF.

All samples will be assayed by a validated bioanalytical method that is specific for the measurement of the analytes relevant to this study. The method validation conducted by the appointed bioanalytical laboratory will address (at a minimum) method accuracy, precision, reproducibility, specificity, recovery and sample stability, and other parameters as appropriate.

Samples of all participants who receive a dose of the active study drug treatments will be analyzed. Repeat analysis may be carried out according to predefined criteria as described in standard operating procedures of the bioanalytical laboratory conducting PK sample analysis for this study. Blood samples (3 mL per timepoint) will be processed with the resultant plasma divided into aliquots and placed into labeled plasma storage tubes. The samples will then be used for the analysis of GSBR-1290 plasma concentrations and determination of PK parameters.

Identification and quantification of GSBR-1290 plasma metabolites may be performed.

Instructions for collection, processing, storage, and transfer of blood samples are provided in the study Laboratory Manual.

8.6. Pharmacodynamics

Cardiovascular PD markers, including but not limited to, free fatty acid, high-sensitivity C-reactive protein, and succinate, will be measured at Day 1, Day 85, and Day 252. In addition, at

Day 85 and Day 252, triglycerides, cholesterol, lipid panel, and other inflammatory markers will be measured.

8.7. Blood Sample Volumes

Blood samples will be collected from each participant throughout the study resulting in the approximate total blood volumes per participant as shown in Table 4. Additional blood may be collected from each participant at any time if required for safety reasons.

Table 4: Approximate Blood Sample Volumes to be Collected per Participant

Sample Type	Blood Volume per Participant
Hematology	30 mL (10 × 3 mL)
Serum chemistry[a]	80 mL (10 × 8 mL)
Coagulation	30 mL (10 × 3 mL)
Viral serology	5 mL (1 × 5 mL)
PK samples	48 mL (16 × 3 mL)
Cardiovascular PD samples	30 mL (10 × 3 mL)
**Total blood volume collected per participant**	**223 mL**

Abbreviations: FSH = follicle-stimulating hormone; PD = pharmacodynamic; PK = pharmacokinetic

a. Also includes serum pregnancy and FSH testing, where required.

9. STATISTICAL CONSIDERATIONS

Detailed methodology for summaries and statistical analyses of the data collected will be documented in a separate Statistical Analysis Plan (SAP) that will be finalized before database lock and unblinding. Any changes to the methods described in the final SAP will be described and justified as needed in the CSR.

This section describes the general framework to be used for the analysis and presentation of data in this study. The information described in this section may be revised during the study to accommodate protocol amendments and to make changes to adapt to unexpected issues in study execution and data that could affect planned analyses. All available data will be included in data listings. Data tabulations will be performed for specific analysis populations.

The study hypothesis for the primary objective is that at least 1 dose level of GSBR-1290 is superior in percent change from baseline in body weight to placebo at Week 36 in participants without diabetes who have obesity or are overweight with at least 1 weight-related comorbidity.

Treatment comparisons will be tested for the primary objective at 2-sided significance level of 0.05. No multiplicity adjustments will be made for the analysis of secondary and exploratory objectives.

9.1. Analysis Sets

For the purposes of analysis, the following analysis sets are defined:

Participant Analysis Set	Description
All Randomized Set	All participants who are randomly assigned to a treatment group.<br>Participant disposition and background data, including demographics, baseline characteristics, participation, and compliance data, will be presented for all participants in the All Randomized Set.
Full Analysis Set	All randomized participants who receive ≥1 dose of study drug (GSBR-1290 or placebo).<br>Efficacy data will be presented for all participants in the Full Analysis Set.
Safety Analysis Set	All participants who receive ≥1 dose of study drug (GSBR-1290 or placebo).<br>Safety and tolerability data will be presented for all participants in the Safety Analysis Set.
Pharmacokinetic Analysis Set	All participants in the Safety Analysis Set who have at least 1 quantifiable plasma concentration.<br>Concentration data will be presented for all participants in the PK Analysis Set.
Pharmacodynamic Analysis Set	All participants in the Safety Analysis Set who have sufficient data to derive ≥1 PD parameter.

Abbreviations: PD = pharmacodynamic; PK = pharmacokinetic.

9.2. Statistical Analyses

Unless stated otherwise, all statistical summaries and analyses will be conducted based on planned randomized treatment group, regardless of the actual treatment received by the participant due to any dose modification. For safety summaries based on the Safety Analysis Set, if a participant randomized to the placebo treatment group accidentally received any GSBR-1290, this participant will be included in the GSBR-1290 group in that cohort.

Participants assigned to placebo in Cohorts 1 to 3 will be pulled together as the treatment group of placebo.

All tests of treatment effects will be conducted at a 2-sided alpha level of 0.05, and confidence intervals (CIs) will be calculated at 95%, 2-sided. All tests of interactions between treatment groups and other factors will be conducted at a 2-sided alpha level of 0.10.

9.2.1. Participant Disposition

Summaries of study disposition and study drug disposition for all randomized participants will be provided by treatment group. Corresponding listing will be provided. Counts and percentages of participants who were randomized, discontinued early from the study, study treatment and completed the study, as well as those who experienced dose interruption or dose reduction, will be summarized by treatment group and in total based on the All Randomized Set. Primary reasons for premature study drug or study discontinuation will also be summarized.

9.2.2. Efficacy Data

A restricted maximum likelihood-based mixed models for repeated measures (MMRM) analysis will be used to analyze continuous longitudinal variables. The models will incorporate treatment group, randomization strata, visit, treatment-by-visit interaction, and baseline weight values as covariates. All scheduled timepoints will be included in the MMRM models. Participants will be considered as repeated measure units over visits. Linear contrast from the model will be used to test the treatment difference between each dose group and placebo at each time point. Test p-values, point estimates, and corresponding 95% CIs of treatment difference will be provided at each timepoint using the MMRM analysis based on least squares means and Type III tests. In the MMRM model, missing data will not be imputed. This analysis is unbiased under the missing at random assumption. An unstructured covariance will be used to model the within-subject variance-covariance structure for the model errors. If convergence fails, a Toeplitz covariance matrix will be used. If Toeplitz covariance matrix does not converge, a first order autoregressive, AR(1), covariance matrix will be used. If convergence is still not met, then compound symmetry will be used.

9.2.2.1. Primary Endpoint

The primary efficacy assessment on percent change from Baseline in body weight at Week 36 will be guided by the primary and secondary estimands and conducted using the Full Analysis Set.

For the primary estimand, the hypothetical strategy is used to handle the ICEs, so only data collected before the occurrence of the ICEs will be used in the MMRM analysis (Section 1.1).

For the secondary estimand, the treatment policy strategy is used to handle the ICEs; that is, all observed values including those collected after ICEs will be used in the analysis regardless of the occurrence of the ICEs.

Primary efficacy comparison will be based on the contrast between each treatment group of GSBR-1290 (45, 90, or 120 mg QD) and placebo at Week 36 from the MMRM analysis of percent change from baseline in body weight. More detail will be provided in the SAP.

9.2.2.2. Secondary Endpoints

The following secondary endpoints will be analyzed on Full Analysis Set:

- Percentage of participants who achieve ≥5% reduction in body weight at Week 36
- Percentage of participants who achieve ≥10% reduction in body weight at Week 36
- Percentage of participants who achieve ≥15% reduction in body weight at Week 36
- Change in body weight (absolute) from Baseline to Week 36
- Change in BMI from Baseline to Week 36
- Change in waist circumference from Baseline to Week 36

Change from Baseline in continuous variables will be analyzed by the MMRM model for primary and secondary estimands as described Section 9.2.2.1.

Treatment comparisons for the percentage of participants with ≥5%, ≥10%, and ≥15% reduction in body weight will be analyzed using a logistic regression model with treatment group, randomization strata, and baseline weight value as covariates. Missing values for such binary endpoints will be imputed by categorizing the imputed values of the missing body weight at Week 36.

9.2.2.3. Exploratory End Points

A descriptive characterization will be done on the following exploratory end points including, but not limited to:

- Change in triglycerides, free fatty acids (FFA), and high-sensitivity C-reactive protein (hsCRP) at Week 36
- Change in patient-reported outcomes (SF-36 acute version, IWQOL-Lite CT, and hunger/satiety evaluation) from Baseline to Week 36
- Change in HDL and LDL cholesterol from Baseline to Week 36

Exploratory endpoints also include evaluation of drug exposure via population pharmacokinetics. Results may be reported in a separate document.

9.2.3. Demographic and Baseline Characteristics

Demographic data (including sex, age, race, and ethnicity) will be summarized using descriptive statistics (n, mean, standard deviation [SD], median, minimum, and maximum) and tabulated by dose group, and overall on the All Randomized Set.

Height, weight, and BMI will be summarized using descriptive statistics (n, mean, standard SD, median, minimum, and maximum) and tabulated by dose group and overall.

Medical history will be coded using the latest version of Medical Dictionary for Regulatory Activities (MedDRA). The number of participants reported with conditions/procedures and the number of conditions/procedures reported will be summarized by treatment and overall, grouped according to system organ class (SOC) and preferred term, using descriptive statistics.

9.2.4. Safety/Tolerability

Unless specified otherwise, safety and tolerability assessments will be guided by an estimand comparing safety of GSBR-1290 doses with placebo irrespective of adherence to study drug including data collected during the treatment period plus safety follow-up from participants who are randomized and are exposed to at least 1 dose of study drug. Thus, safety analyses will be conducted using the Safety Analysis Set.

Safety evaluations will be based on the incidence, severity, and relationship of AEs, vital signs, physical examination findings, clinical laboratory results, and ECGs.

Abnormalities in clinical laboratory parameters and the ECG parameter QTcF will be based on predefined normal ranges and will be tabulated by dose group showing participant counts and percentages.

9.2.4.1. Extent of Exposure

Study drug administration will be listed by participant. Duration of treatment and drug compliance (% of dosage and % of tablet) will be presented by treatment group and overall with descriptive statistics, as well as counts and percentage of participants with exposure and compliance in certain categories.

Days of exposure to study drug will be calculated as date of last dose of study drug – date of first dose of study drug + 1. Percent of compliance to the study drug regimen will be calculated as 100 × number of actual tablets taken / number of expected tablets taken.

9.2.4.2. Adverse Event

All AEs will be coded using the latest version of MedDRA by SOC and preferred term, classified from verbatim terms. The count and percentage of participants with TEAEs will be summarized by SOC and preferred term and tabulated for each dose group. Summaries of TEAEs by severity and relationship will also be presented. Summaries will also be presented for AESI (Section 8.4.2), AEs leading to death, SAEs, AEs leading to dose interruption, AEs leading to dose reduction, and for AEs leading to study withdrawal. The duration, treatment, and outcome of AEs will be determined and included in the listings.

9.2.4.3. Prior and Concomitant Medication

Prior and concomitant medications will be coded using the latest version of the World Health Organization Drug Dictionary (WHO-DD). The number and percentage of participants taking prior medication and concomitant medication will be summarized using frequency tables according to WHO Drug Anatomical Therapeutic Chemical Level 1 Code Description and the WHO-DD Preferred Name. If a participant has >1 prior or concomitant medication coded to the same category, the participant will be counted only once.

9.2.4.4. Vital Signs

Observed values and changes from baseline for vital signs (systolic and diastolic BP, HR, body temperature, and RR) will be summarized at each scheduled timepoint using descriptive statistics (n, mean, SD, median, minimum, and maximum) and tabulated for each dose group.

9.2.4.5. Physical Examination Data

Physical examination data will be listed only. It is noted that any postdose clinically significant findings will be reported as AEs. Body weight and BMI changes from baseline compared with placebo will be assessed, details of which will be addressed in the SAP.

9.2.4.6. Clinical Laboratory Data

Clinical laboratory safety data will be summarized by the laboratory parameter. Normal reference ranges and abnormal results will be used in the summary of laboratory data. Observed values and changes from baseline for continuous clinical laboratory parameters will be summarized at each scheduled timepoint using descriptive statistics (n, mean, SD, median, minimum, and maximum) and tabulated for each dose group. Categorical clinical laboratory data will be summarized at each scheduled timepoint using participant counts and percentages and tabulated by dose group. Shift tables presenting the number and percentage of participants with a normal value at baseline and a postbaseline abnormal value will also be summarized by treatment group. Individual participant profiles will be presented for any laboratory parameters with ≥1 postdose value outside the laboratory's reference ranges and deemed clinically significant.

9.2.4.7. Electrocardiogram Data

The following ECG parameters will be analyzed: PR interval, QT interval, QRS duration, QTcF, and ventricular HR. Observed values and changes from baseline for ECG parameters will be summarized at each scheduled timepoint using descriptive statistics (n, mean, SD, median, minimum, and maximum) and tabulated for each dose group.

For QTcF, the number of participants with values >450, >480, and >500 msec or an increase from baseline >30 and >60 msec will also be presented, in accordance with International Council for Harmonization of Technical Requirements for Pharmaceuticals for Human Use (ICH) E14 (note for guidance on the clinical evaluation of QT/corrected QT interval prolongation and proarrhythmic potential for non-antiarrhythmic drugs, Committee for Medicinal Products for Human Use/ICH/2/04).

9.2.5. Pharmacokinetic Data

9.2.5.1. Plasma Pharmacokinetic Parameters

Plasma concentrations of GSBR-1290 at specified timepoints predose and postdose will be used to calculate PK parameters, via population PK analysis.

Individual participant GSBR-1290 plasma concentrations at each timepoint will be listed and summarized by treatment using descriptive statistics (n, mean, SD, coefficient of variation, minimum, maximum, median, geometric mean and coefficient of variation of the geometric mean) for each dose group. Individual and mean (by treatment) plasma concentration-time data may be presented graphically.

Plasma concentration-time data of GSBR-1290 may be used for the calculation of the following PK parameters using population PK:

- C_{max}

- T_{max}
- AUC_{0-tau}
- Accumulation Ratio

PK parameters and analysis might be described in a separate report.

Plasma samples may be used for identification of GSBR-1290 metabolites. Metabolite identification data will be used for internal exploratory purposes and will not be included in the CSR.

9.2.6. Pharmacodynamic Data

The PD effect of GSBR-1290 will be assessed based on several variables, including, but not limited to, triglycerides, FFA, cholesterol, hsCRP, and body weight.

9.2.7. Subgroup Analyses

Subgroup analyses of the primary endpoint will be made to assess consistency of the treatment effect across the following subgroups using the primary estimand:

- Age group: <65 versus ≥65 years
- Sex: female versus male
- Baseline BMI: ≤30, >30 to <35, ≥35 kg/m^2
- Pre-diabetes status (HbA1c <5.7% versus ≥5.7%)
- Race
- Ethnicity
- Study centers
- Participants with and without known (previously diagnosed) metabolic disorders at Baseline (ACVD, MASH, eGFR<60, triglycerides >200 mg/dL (2.2 mmol), or heart failure)

If the number of participants is too small (<10%) within a subgroup, then the subgroup categories may be redefined prior to unblinding the study. Further details on the statistical analysis will be provided in the SAP.

For the subgroup analysis, the same fixed effects given for the primary analysis model plus factors of subgroup, 2-way interaction of subgroup and treatment, 2-way interaction of subgroup and visit, and 3-way interaction of treatment, visit, and subgroup. The interaction of subgroup and treatment at the primary efficacy endpoint will be evaluated to assess the treatment by subgroup interaction. Additional subgroup analyses may also be performed.

9.3. Interim Analysis

An interim (efficacy and safety) analysis will be conducted to inform dose escalation schemes to accelerate the development of future studies after all participants complete the Week 26 visit (when participants have completed titration and safety and tolerability has been observed for at

least 4 weeks at each dose level). There will be no action taken with respect to the study conduct and analysis, including stopping the study based on the superiority of GSBR-1290 versus placebo of the interim analysis. There will be no inflation of the Type 1 error rate and no need to employ an alpha spending function or multiplicity adjustment. An independent data center will carry out the analysis and the Sponsor will review the interim analysis for safety and efficacy. Details of the interim analyses, operational support, and unblinding will be specified in the SAP.

9.4. Sample Size Determination

Approximately 220 participants will be randomly assigned to active treatment and placebo in a randomization ratio of 3:1 in all 3 cohorts. All participants assigned to placebo in each cohort will be pulled together as the placebo group, which will be compared to each GSBR-1290 treatment group in Cohort 1 to 3 to establish efficacy. The sample size calculation is based on the primary estimand and its endpoint (percent body weight change from Baseline to Week 36). Assuming an SD of 6%, a 2-sided alpha level of 0.05, 35 completers for one GSBR-1290 treatment group and 35 completers for the placebo group can provide approximately >90% power to detect a treatment difference of -5% between the GSBR-1290 treatment group and placebo in percent body weight change from Baseline to Week 36. Assuming a 20% dropout rate in GSBR-1290 45 mg group, 60 participants will be randomized for Cohort 1, with 45 participants on GSBR-1290 45 mg and 15 participants on placebo. Assuming a 35% dropout rate in all other treatment groups, 80 participants will be randomized for each of Cohort 2 and 3, with 60 participants on GSBR-1290 and 20 participants on placebo.

10. SUPPORTING DOCUMENTATION AND OPERATIONAL CONSIDERATIONS

10.1. Appendix 1: Regulatory, Ethical, and Study Oversight Considerations

10.1.1. Regulatory and Ethical Considerations

10.1.1.1. Investigator Responsibilities

The Investigator must conduct the clinical study in accordance with the Institutional Review Board / Ethics Committee (IRB / EC)-approved study protocol and the GCP regulations, applicable in the jurisdiction(s) in which the study is conducted and, in which, the Sponsor intends to submit the study for regulatory consideration and/or marketing authorization.

10.1.1.2. Ethical Considerations

The Investigator(s) and all parties involved in this study will conduct the study in the spirit of the ethical principles set forth in the Declaration of Helsinki, and the current ICH GCP guidelines.

Conduct of the study must be approved by an appropriately constituted IRB / EC before participants are enrolled. Prior to initiation of the study, written IRB / EC approval of the protocol and ICFs, based on the principles of current ICH GCP will be received. This approval must be issued on the IRB / EC letterhead and refer to the ICFs and to the study protocol by title and protocol number and include date of each document.

A copy of the signed and dated letter of approval will be emailed to the Sponsor or designee before commencement of the study. Any written information and/or advertisements to be used for participant recruitment must be approved by the IRB / EC before use. A list of the IRB / EC voting members, their titles or occupations, and their institutional affiliations will also be provided to the Sponsor's representative before study initiation. If approval is suspended or terminated by the IRB / EC, the Investigator will notify the Sponsor immediately.

Protocol amendments that may impact participant safety or the validity of the study will be approved by the IRB / EC, following written agreement for the amendment from the Sponsor. The amended protocol will not be implemented at sites until such approvals are received other than in the case of an urgent safety measure.

The Investigator must maintain an accurate and complete record of all submissions made to the IRB / EC. The records should be filed in the Investigator Site File and copies must be sent to the Sponsor and maintained in the study Trial Master File.

The trial will be submitted to required clinical trial registries (www.clinicaltrials.gov).

The Sponsor will ensure that participants will be informed, through the ICF, of the availability of medical care or compensation for injury (if any) for more than minimal risk research.

10.1.2. Informed Consent Process

The Investigator is responsible for obtaining informed consent from each participant participating in the trial in compliance with US Title 21 Code of Federal Regulations (21 CFR) Part 50, and ICH and IRB / EC guidelines.

Prior to initiation of the trial at the site, the ICF must be reviewed and accepted by the Sponsor and approved by the governing IRB / EC. The Investigator or medically certified delegate will discuss the purpose and pertinent details of the trial with each potential participant, provide sufficient time to consider the study, and if the potential participant understands and agrees to participate, sign, and date the appropriate IRB / EC-approved ICF before undergoing any trial-specific procedures. The ICF must be personally signed and dated by the participant and by the person who conducted the informed consent discussion. Additional signature requirements may exist. The original signed and dated ICF will be retained with the participant's trial records, and a copy of the signed ICF will be given to the participant. The Investigator or medically certified delegate will maintain a log of all participants who sign the ICF. At a minimum, the log will include a participant identifier, the dates of informed consent and screening procedures, the outcome of the screening, and the reason the participant did not enroll in the trial, if applicable.

Should a protocol amendment become necessary, the ICF may need to be revised to reflect the changes to the protocol. It is the responsibility of the Sponsor or designee to ensure that an amended ICF is reviewed and has received favorable opinion from the IRB / EC, and the Investigator has to ensure that the amended ICF is signed by all participants subsequently entered in the trial and those currently in the trial, if affected by the amendment.

10.1.3. Data Protection

The Investigator is responsible for maintaining the privacy and confidentiality of the participant's medical or health information collected during the trial. The Investigator is also responsible for ensuring that all use, review, and disclosure of participant's medical or health information is in accordance with Health Insurance Portability and Accountability Act regulations and the ICF approved by the IRB / EC and signed by the participant. Specifically, all data collected about a participant during the trial will be identified only by a number and the participant's initials.

10.1.4. Study Protocol Guidelines

10.1.4.1. Emergency Contact With Investigator

Suitable arrangements must be made for participants to contact the Investigator or medically certified delegate in the event of an emergency.

10.1.4.2. COVID-19 Preparedness

The clinical site should have in place procedures and strategies to accommodate the current COVID-19 preparedness. Such procedures should include requirements in relation to criteria, such as the following:

- Attendance (e.g., who is permitted to be on-site, limitations, records of attendance, plans for suppliers and deliveries)

- Physical layout (e.g., physical distancing requirements and signage)
- Flexibility (e.g., procedures for scaling, ability to respond to outbreak)
- Support for remote interactions with Sponsors and study teams (e.g., communication, including infrastructure)
- The local environment (e.g., contact with local health authorities, ability to access up-to-date information)
- Any other relevant criteria

10.1.4.3. Protocol Deviations and Violations

Investigators and study site personnel must not deviate from the protocol unless it is absolutely necessary to avoid an immediate hazard to the study participants. The Investigator is responsible for complying with all protocol requirements and applicable regulations regarding protocol deviations. The Sponsor must be notified of all deviations to the protocol (e.g., inclusion/exclusion criteria, dosing, missed study visits) to determine the impact of the deviation on the participant and/or the study.

The Investigator should notify the IRB / EC of significant deviations from the protocol in accordance with local procedures.

10.1.4.4. Protocol Waivers

Protocol waivers will not be granted by the Sponsor in this study.

10.1.4.5. Protocol Amendments

Administrative amendments to the protocol will be classed as amendments of typographical errors, clarifications of confusing wording, and other minor modifications, including, but not limited to, name, address, and contact information changes that have no impact on the safety of the participant or the science of the study. Administrative amendments will be submitted to the IRB / EC for information only. The Sponsor (or designee) will ensure that acknowledgement is received and filed. Otherwise, an amendment will be classed as a substantial amendment and will be submitted to the IRB / EC for approval and the appropriate regulatory authorities, as applicable.

10.1.5. Quality Control and Assurance

10.1.5.1. Study Monitoring

The Sponsor is responsible for assuring the proper conduct of the study with regard to protocol adherence and validity of the data recorded in the eCRF.

During the course of the trial, the Sponsor and/or designee will visit the trial sites and/or perform remote monitoring at regular intervals. The Investigators must make themselves available to the Study Monitor during an on-site (or remote) monitoring visit to enable discussion on any arising issues relating to the study. The purpose of the monitoring (on-site or remote) is to ensure that the eCRF is completed correctly and the protocol is being adhered to, to perform source data verification, and to monitor drug accountability.

The Investigator agrees to allow the Study Monitor direct access to relevant source documents (original documents, data, and records). Direct access includes permission to examine, analyze, verify, and reproduce any record(s) and report(s) that are important to evaluation of the clinical trial. The Investigator also agrees to allocate his/her time and the time of his/her staff to the Study Monitor to discuss findings and any relevant issues.

Participant confidentiality must be maintained during any monitoring activities conducted by the Sponsor or designee.

In accordance with applicable regulations and ICH GCP, a Study Monitor will contact the site to organize an on-site (or remote) visit before participant enrolment to review the protocol and data collection procedures with site staff. In addition, the assigned Study Monitor will periodically contact the site, including conducting on-site (or remote) visits. The extent, nature, and frequency of on-site visits will be based on such considerations as the study objectives and/or endpoints, the purpose of the study, study design complexity, and enrolment rate.

During these site visits, the Study Monitor will do the following:

- Check the progress of the study.
- Review study data collected.
- Conduct source document verification.
- Identify any issues and address their resolution.
- Check study treatment accountability records.
- Review biological samples collected for the study and ensure that they are labeled and stored correctly.
- Verify the following:
  - The data are authentic, accurate and complete
  - Safety and rights of participants are being protected
  - Study is conducted in accordance with the currently approved protocol (and any amendments), ICH GCP, and all applicable regulatory requirements

At study closure, a Study Monitor will conduct the following activities in conjunction with the Investigator or site staff as appropriate:

- Ensure that all data queries have been resolved
- Conduct final accountability and reconciliation for study treatment supplies, including inventory and final disposition (e.g., destruction, shipping to repository, etc.)
- Review of site study records for completeness

10.1.5.2. Quality Assurance and Quality Control

To ensure compliance with all applicable GCP regulations, the Sponsor (or a designated third party), may conduct a quality assurance audit of the study site. Regulatory agencies may also conduct a regulatory inspection of this study. Such audits/inspections can occur at any time during or after completion of the study. If an audit or inspection occurs, the Investigator and

Institution agree to notify the Sponsor as soon as possible following awareness of an impending regulatory inspection. The Investigator and Institution agree to allow the auditor/inspector direct access to all relevant documents and allocate his/her time and the time of his/her staff to the auditor/inspector to discuss findings and any relevant issues.

The Sponsor will perform or oversee the performance of the quality assurance and quality control activities for this study according to GCP and standard operating procedures; however, responsibility for the accuracy, completeness, and reliability of the study data presented to the Sponsor lies with the Investigator (and delegate[s]) generating the data.

Prior to the study initiation, the Sponsor or Sponsor's representative will explain the protocol, Investigator's Brochure, and eCRF to the Investigator and the site staff involved in this study. In addition, during and after the trial, periodic site visits will be made to monitor for compliance, including verification of the accuracy and completeness of data recorded on the eCRFs, source documents, and trial treatments accountability records.

10.1.6. Dissemination of Clinical Study Data

After the EOS, a CSR will be written and submitted to relevant IRB / EC and regulatory authorities in accordance with local requirements.

10.1.7. Source Documents

Source documentation must be prepared and available for Sponsor review prior to commencement of enrollment activities and must capture, at a minimum, the following aspects and parameters:

- Adherence to protocol procedures
- Participant identification, eligibility, and participation
- Informed consent procedures
- Protocol specified assessments
- Dates of visits
- Safety parameters, including reporting and follow-up of AEs and use of concomitant medication
- PK and PD parameters
- Study drug receipt/dispensing/return records
- Study drug administration information
- Date of participant completion
- Discontinuation from treatment or withdrawal from the study and the reason, if appropriate

The author of an entry in the source documents must be identifiable. Worksheets may be used for the capture of data to facilitate completion of the eCRF. Any such worksheets will become part of the participants' source documentation.

Following the ICH GCP guidelines, direct access to source documentation and medical records must be allowed for the purpose of verifying that the data recorded in the eCRF are consistent with the original source data.

10.1.8. Electronic Case Report Form

The Sponsor will designate a CRO to perform data management. eCRFs will be used at the clinical trial site to collect trial data for enrolled participants. When data are available, authorized clinical trial site personnel will carefully and accurately record the data on the eCRF. Sites must ensure that all source documents are maintained according to ICH/GCP guidance and support the data that are entered onto the eCRFs. The eCRF data will be captured in a system validated according to procedures that comply with Food and Drug Administration 21 CFR Part 11 and the ICH E6(R2) Guidelines for GCP. The eCRFs will be reviewed and signed by the Investigator or someone clinically qualified and identified on the delegation log as someone who can sign-off on the eCRF.

The CRO will write a data management plan outlining the data management systems, procedures, and agreements between the CRO and Sponsor. The plan will be reviewed and signed by a representative of the Sponsor's data management department. Outside the electronic data capture system, when appropriate, the CRO will receive FDA 21 CFR Part 11 compliant external laboratory data transfers from a validated laboratory information management system. After database lock, the Investigator will receive a copy of the participant data for archiving at the trial site.

10.1.9. Recordkeeping and Retention of Records

In compliance with the ICH GCP guidelines, the Investigator/institution will maintain all source documents that support the data collected from each participant, as well as all study documents as specified in ICH E6(R2) GCP, Essential Documents for the Conduct of a Clinical Trial. The Investigator/institution will take measures to prevent accidental or premature destruction of these documents.

The Investigator must retain all records relating to the conduct of this trial for ≥2 years after a marketing application for the drug is approved; or if an application is not filed or not approved for the drug, for ≥2 years after clinical development for the drug has been formally discontinued, and the appropriate regulatory or health authorities have been notified. However, in certain instances, documents may need to be retained for a longer period if required by regulatory requirements or by an agreement with the Sponsor. The Investigator must allow representatives of the FDA, the governing IRB / EC, or other regulatory agencies to inspect all trial records. If informed of such an inspection, the Investigator will notify the Sponsor immediately. The Investigator must obtain written approval from the Sponsor prior to the destruction of any records relating to the conduct of this trial. If the responsible Investigator retires, relocates, or for other reasons withdraws from the responsibility of keeping the study records, custody must be transferred to a person who will accept the responsibility. The Sponsor must be notified in writing of the name and address of the new custodian.

Under no circumstance shall the Investigator relocate or dispose of any study documents before having obtained written approval from the Sponsor.

10.1.10. Study and Site Start and Closure

10.1.10.1. Notification of the General Practitioner

It is the responsibility of the Investigator, or medically certified delegate, to notify, where applicable, the participant's general practitioner of the participant's involvement in the trial. This notification should include the nature of the trial, treatments, expected benefits, AEs, and concomitant drugs to be avoided. The consent of the participant should be sought before contacting their general practitioner.

10.1.10.2. Clinical Laboratory Certification and Reference Ranges

Before the initiation of this study, the Investigator, or medically certified delegate, will obtain a copy of the certification form, with certification number and expiration date for all clinical laboratories used in the study. Reference ranges for each clinical laboratory test used in this study will be obtained from the laboratory that will perform the test during the study.

10.1.10.3. Study/Site Termination

The Sponsor reserves the right to do the following:

- Suspend or terminate execution of this clinical study at any time
- Suspend or terminate participation of an investigational site of this clinical study at any time

Reasons will be provided in the event of any of the above occurrences taking place. Reasons for termination may include but are not limited to the following:

- Unacceptable safety and tolerability
- Serious or persistent noncompliance by the Investigator with the protocol, clinical research agreement, principles of the ICH GCP, or applicable regulatory guidelines in conducting the study
- IRB / EC decision to terminate or suspend approval for the Investigator
- Regulatory authority request for study suspension or termination
- Enrollment is unsatisfactory

The Investigator reserves the right to withdraw from the study for safety reasons at any time following consultation with the Sponsor.

10.1.10.4. Study Completion/Site Closure

The study is considered completed at the last contact of the last participant involved in the study. The final data from the investigational site will be sent to the Sponsor (or designee) in the time frame specified in the Clinical Trial Agreement. Investigational sites will be closed upon study completion. An investigational site is considered closed when all required documents and study supplies have been collected and a site closure visit has been performed by the Sponsor (or designee).

10.1.10.5. EOS and Regulatory Notification

At EOS, the IRB / EC and relevant regulatory authorities will be notified by the Sponsor (or designee) according to applicable IRB / EC and regulatory requirements.

10.1.11. Financing and Insurance

Financial aspects of the study are addressed in a separate clinical study agreement.

The Investigator is required to have adequate current insurance to cover claims for negligence and/or malpractice.

The Sponsor will provide insurance coverage for the clinical study as required by national regulations. The Sponsor will be subscribed to an insurance policy covering, in its terms and provisions, its legal liability for injuries caused to participating persons and arising out of this research performed strictly in accordance with the study protocol as well as with applicable law and professional standards prior to commencement of the study.

10.1.12. Intellectual Property, Confidentiality, and Publications

10.1.12.1. Ownership

All information provided by the Sponsor and all data and information generated by the clinical site staff as part of the study (other than a participant's medical records) are the sole property of the Sponsor.

All rights, title, and interests in any inventions, know-how, or other intellectual or industrial property rights that are conceived or reduced to practice by clinical site staff during the course of or as a result of the study are the sole property of the Sponsor and are hereby assigned to Gasherbrum Bio, Inc.

If a written contract, which includes ownership provisions inconsistent with this statement for the conduct of the study, is executed between the Sponsor and the clinical site, that contract's ownership provisions shall apply rather than this statement.

10.1.12.2. Confidentiality

All information provided by the Sponsor and all data and information generated by the clinical site as part of the study will be kept confidential by the Investigator and other site staff. The Investigator or other site personnel will not use this information and data for any purpose other than conducting the study. These restrictions do not apply to the following:

- Information that becomes publicly available through no fault of the Investigator or site staff
- Information that is necessary to disclose in confidence to an IRB / EC solely for the evaluation of the study
- Information that is necessary to disclose in order to provide appropriate medical care to a study participant
- Study results that may be published as described in the Publication Policy (Section 10.1.12.3)

If a written contract, which includes confidentiality provisions inconsistent with this statement for the conduct of the study, is executed, that contract's confidentiality provisions shall apply rather than this statement.

10.1.12.3. Publication Policy

The Sponsor may publish the results of this study at an appropriate time. No publication of the results shall take place without the express consent of the Sponsor. Prior to submitting for any publication, presentation, use for instructional purposes, or otherwise disclosing the study results generated by the sites (collectively, a "Publication"), the Investigator shall provide the Sponsor with a copy of the proposed publication and allow the Sponsor a period of ≥30 calendar days (or for abstracts, ≥15 working days) to review the proposed publication. Proposed publications shall not include Sponsor confidential information.

At the request of the Sponsor the submission or other disclosure of a proposed publication will be delayed for a sufficient length of time to allow the Sponsor to seek patent or similar protection of any inventions, know-how or, other intellectual or industrial property rights disclosed in the proposed publication.

If a written contract, which includes publication provisions inconsistent with the statement for the conduct of the study, is executed, that contract's publication provisions shall apply rather than this statement.

10.2. Appendix 2: Clinical Laboratory Tests

Table 5: Full Laboratory Tests

**Hematology**	**Serum Chemistry, Including Liver Function and Urinalysis**		
Hemoglobin	Sodium	Creatine kinase	Total cholesterol
Hematocrit	Potassium	Creatinine	High-density lipoprotein
Red blood cell indices	Chloride	eGFR calculated using CKD-EPI formula	Low-density lipoprotein
Thrombocyte count (platelets)	Bicarbonate	Blood urea nitrogen	Triglycerides
WBC count (absolute and %) with differential	Phosphate	Alanine transaminase	Calcitonin
**Coagulation**	Calcium	Alkaline phosphatase	Albumin
Prothrombin time	Glucose	Aspartate transaminase	Globulins
INR	Amylase	Gamma-glutamyl transferase	Total protein
aPTT	Lipase	Total bilirubin	Lactate dehydrogenase
Fibrinogen	Uric acid	Conjugated bilirubin	Thyroid stimulating hormone
	Magnesium	Unconjugated bilirubin	hemoglobin A1c
			high-sensitivity C-reactive protein
**Virology**	**Drug Screen (Serum or Urine)** [b]		**Confirmation of Postmenopausal Status**
HIV-1/-2 antibody	Methamphetamines	Barbiturates	FSH for postmenopausal women
HbsAg	Opiates	Methadone	Pregnancy test
Hepatitis C virus antibody and viral load	Cocaine	Tricyclic antidepressants	Serum hCG [c]
COVID-19, SARS-COV-2 antibody[a]	Benzodiazepines	Amphetamines	Urine hCG [c]

Abbreviations: aPTT = activated partial prothrombin time; CKD-EPI = Chronic Kidney Disease Epidemiology Collaboration; COVID-19 = coronavirus disease 2019; eGFR = estimated glomerular filtration rate; FSH = follicle-stimulating hormone; HbsAg = hepatitis B surface antigen; hCG = human chorionic gonadotropin; HIV = human immunodeficiency virus; INR = international normalized ratio; PCR = polymerase chain reaction; SARS-COV-2 = severe acute respiratory syndrome coronavirus 2; WBC = white blood cell.

a. COVID-19 test performed only if clinically indicated as determined by the Investigator; COVID-19 antigen test is performed first, and then PCR test for confirmation if clinically indicated.
b. Either serum or urine drug screening is acceptable according to site routine procedure but should be kept consistent throughout the study.
c. If the urine hCG is positive or inconclusive, pregnancy will be confirmed by a serum hCG test.

10.3. Appendix 3: Adverse Events and Serious Adverse Events: Definitions and Procedures for Recording, Evaluating, Follow-up, and Reporting

10.3.1. Definition of Adverse Event

An AE is any untoward medical occurrence in a clinical study participant that is administered a pharmaceutical product and does not necessarily have to have a causal relationship with this treatment. An AE can therefore be any unfavorable and unintended sign (including an abnormal laboratory finding, for example), symptom, or disease temporally associated with the use of a medicinal (investigational) product, whether or not considered related to the medicinal (investigational) product (definition per ICH).

Any AE for which a causal relationship with the IP is at least a reasonable possibility (possibly or probably related) is referred to as an adverse drug reaction.

The following are considered normal for the class of GLP-1RA drugs and thus should not be considered adverse events:

- Loss of appetite
- Early satiety
- Weight loss

10.3.2. Definition of Serious Adverse Event

An SAE is any AE that, at any dose, meets at least 1 of the following:

- Results in death
- Is life-threatening

  Note: The term 'life-threatening' in the definition of 'serious' refers to an event in which the participant was at risk of death at the time of the event; it does not refer to an event that hypothetically might have caused death, if it were more severe.

- Requires inpatient hospitalization or prolongation of an existing hospitalization,
- Note: Only hospitalizations that are longer than expected based on Investigator judgment will be considered prolonged hospitalizations.
- Results in persistent or significant disability/incapacity
- Note: Results in a substantial disruption of a person's ability to conduct normal life functions.
- Is a congenital anomaly/birth defect (that occurs in the offspring of a participant exposed to the IP)
- Other important medical event

  Medical and scientific judgment should be exercised in deciding whether other situations should be considered serious, such as important medical events that may not be immediately life--threatening or result in death or hospitalization but may jeopardize the participant or may require medical or surgical intervention to prevent one of the other outcomes listed in the above definition. These should also be considered serious.

  Examples of such events are intensive treatment in an emergency room or at home for allergic bronchospasm, blood dyscrasias or convulsions that do not result in hospitalization, or development of drug dependency or drug abuse.

10.3.3. Definition of Suspected Unexpected Serious Adverse Reaction

A suspected unexpected serious adverse reaction (SUSAR) is an AE that meets all the following criteria:

- Is serious

- There is at least a reasonable possibility of a causal relationship between the event and the IP
- Is considered unexpected (i.e., the event is not listed in the Reference Safety Information section of the Investigator's Brochure or is not listed at the specificity or severity that has been observed)
- Note: "Unexpected" as used in this definition also refers to AEs that are mentioned in the Investigator's Brochure as occurring with a class of drugs or are anticipated from the pharmacological properties of the drug but are not specifically mentioned as occurring with the particular drug under investigation.

10.3.3.1. Assessment of Intensity

The Investigator will assess the severity of each AE and SAE reported during the study. The assessment will be based on the Investigator's clinical judgment. The severity of each AE and SAE will be graded using the most current version of the NCI-CTCAE (Version 5) 5-point scale (Table 6).

Table 6: NCI-CTCAE 5-Point Scale for Grading the Intensity of Each AE and SAE

Grade		Definition
I	Mild	Asymptomatic or mild symptoms: Clinical or diagnostic observations only; intervention not indicated
II	Moderate	Minimal, local or noninvasive intervention indicated; limiting age-appropriate- instrumental activities of daily living
III	Severe	Severe or medically significant but not immediately life-threatening: Hospitalization or prolongation of hospitalization indicated; disabling; limiting self-care activities of daily living
IV	Life-threatening[a]	Life-threatening consequences, urgent intervention indicated
V	Death[a]	Death related to AE

Abbreviations: AE = adverse event; NCI-CTCAE = National Cancer Institute Common Terminology Criteria for Adverse Events; SAE = serious adverse event.

a. Applies only to SAEs.

If an AE has multiple aspects (symptoms), the aspect with the highest severity will be graded.

The term severe is a measure of severity. Thus, a severe AE is not necessarily serious. For example, itching for several days may be rated as severe but may not be clinically serious.

10.3.3.2. Assessment of Causality

The Investigator will make an assessment as to the relationship between the study drug and the occurrence of each AE/SAE. The Investigator will use clinical judgment to determine whether or not the AE/SAE is causally related to the study treatment(s). Alternative causes, such as natural history of an underlying medical condition, concomitant therapy, other risk factors, and the temporal relationship of the event to the study drug, will be considered and investigated. The Investigator will also consult the Investigator's Brochure (or the product information sheet in instances where the study drug is an approved agent) in the determination of his/her assessment.

The causal relationship of the study drug to an AE will be rated according to the scale in Table 7.

Table 7: Relationship of the Study Treatment to an AE (Definitions)

Causal Relationship	Definition
Not related	Another cause of the event is most plausible; or clinically plausible temporal sequence is inconsistent with the onset of the event and the trial treatment administration; or a causal relationship is considered biologically implausible.
Unlikely related	An event or laboratory test abnormality, with a time to drug intake that makes a relationship improbable (but not impossible); disease or other drugs provide plausible explanations.
Possibly	An event that follows a reasonable temporal sequence from administration of the trial treatment or a known or expected response pattern to the suspected drug, but that could readily have been produced by a number of other factors.
Probably	An event that follows a reasonable temporal sequence from administration of the trial treatment, and there is a biologically plausible mechanism for trial treatment causing or contributing to the AE, and the event could not be reasonably explained by the known characteristics of the participant's clinical state. In addition, the relationship may be confirmed by improvement on stopping the trial treatment and reappearance of the event on repeated exposure.
Related	An event cannot be reasonably explained by an alternative explanation, e.g., concomitant drug(s), concomitant disease(s). The relationship in time to study drug administration is very suggestive.

Abbreviations: AE = adverse event.

The Investigator will follow all related AEs observed during the trial until the AEs are considered resolved, until 30 days after the last dose of GSBR-1290, or until completion of the follow-up visit, whichever is later. The Investigator will follow all SAEs, whether assessed as possibly related, probably related, or not related to study treatment, until the SAEs are considered stable, resolved, or until EOS (whichever is earlier). The Sponsor may request additional follow-up on specific unresolved AEs.

10.3.4. Action Taken and Outcome

For all AEs reported, the actions taken and outcomes must be specified and documented in the eCRF. Actions taken with the study drug may include any of the following:

- Dose not changed
- Dose reduced
- Drug withdrawn
- Not applicable
- Unknown
- Drug interrupted

Other action(s) taken:

- No action
- Medication required

- Termination of a concomitant medication
- Change of the dose of a concomitant medication
- Hospitalization or prolongation of hospitalization (please complete an SAE Form)
- Initiation/termination of a nondrug therapy
- Other (please specify)

The following terms and definitions are used in assessing the final outcome of an AE (Table 8).

Table 8: Terms and Definitions Used in Assessing the Final Outcome of an AE

Term	Definition
Recovered	The participant has fully recovered, or by medical or surgical treatment, the condition has returned to the level observed at the first trial-related activity after the participant signed the informed consent.
Recovering	The condition is improving and the participant is expected to recover from the event.
Recovered with sequelae	The participant has recovered from the condition but with lasting effect due to a disease, injury, treatment, or procedure. If a sequela meets an SAE criterion, the AE must be reported as an SAE.
Not recovered	The condition of the participant has not improved, and the symptoms are unchanged, or the outcome is not known at the time of reporting.
Fatal	This term is only applicable if the participant died from a condition related to the reported AE. Outcomes of other reported AEs in a participant before he/she died should be assessed as “recovered,” “recovering,” “recovered with sequelae,” or “not recovered.” An AE with a fatal outcome must be reported as an SAE.
Unknown	This term is only applicable if the participant is lost to follow-up.

Abbreviations: AE = adverse event; SAE = serious adverse event.

10.3.5. Recording Adverse Events and/or Serious Adverse Events

All AEs, regardless of seriousness, severity, or presumed relationship to study treatment, must be collected from time of signing the ICF and through 30 days after date of the last dose and recorded using medical terminology in the eCRF and/or other sources. Whenever possible, diagnoses should be given when signs and symptoms are due to a common etiology (e.g., cough, runny nose, sneezing, sore throat, and head congestion may be reported as “upper respiratory infection”). Investigators must record in the eCRF the date of onset of the event, their opinion concerning the relationship of the AE to the study drug, severity of the event, whether the event is serious or nonserious, actions taken to manage the event, the outcome of the event, and date of resolution where applicable.

This study will utilize electronic patient-reported outcome/e-diary technology. Any specific symptoms should be captured in the e-diary by the participant and assessed by the site staff for reporting as AEs.

Any increase to the severity of an AE must be recorded as a separate AE, ensuring that the end date and time of the preceding AE matches the start date and time of the subsequent AE so that the overall duration of the AE is continuous.

10.3.5.1. Follow-up of Adverse Events and Serious Adverse Events

The Investigator will ensure that AE/SAE follow-up includes any supplemental investigations as may be indicated to elucidate the nature and/or causality of the event. This may include additional laboratory tests or investigations, histopathological examinations, or consultation with other health care professionals. The Sponsor may also request that the Investigator perform or arrange for the conduct of supplemental measurements and/or evaluations.

If a participant dies during participation in the study or during a recognized follow-up period, the Sponsor will be provided with a copy of any postmortem findings, including histopathology.

New or updated information will be reported as required by Section 10.3.6. Due to COVID-19, Investigators must also ensure compliance with local governing legislation and reporting requirements associated with COVID-19 infections.

10.3.6. Reporting of Serious Adverse Events, Pregnancy, and Suspected Unexpected Serious Adverse Reactions

The Investigator must notify the study Sponsor and/or its designee within 24 hours of becoming aware of the occurrence of an SAE or pregnancy. If any new clinical information relating to an existing SAE or pregnancy becomes available, this information must also be reported within 24 hours.

The notification must be directed to the following:<br>E-mail: [redacted]

Information on SAE or pregnancy will be recorded on the SAE or Pregnancy Form, respectively. All SAE/pregnancy information should be entered into the eCRF. If the eCRF is not available, a paper SAE or Pregnancy Form should be completed and sent to Parexel Safety Services via email. Blank copies are included in the study Investigator's file. It is not acceptable for the Investigator to send photocopies of the participant's medical records to the Sponsor company or its representative in lieu of completion of the appropriate AE eCRF page or SAE Form. However, there may be instances when copies of medical records for certain cases are requested by the Sponsor or its representative. In this instance, all participant identifiers will be blinded on the copies of the medical records before submission to the Sponsor or its representative.

The completed SAE/Pregnancy Form and cover sheet should be sent via email immediately upon completion to structuretx_safety@parexel.com. If the Investigator does not have all information regarding an SAE/pregnancy, they will not wait to receive additional information before completing and sending the form.

Additional relevant information or clinical follow-up should be sent via email to structuretx_safety@parexel.com as soon as it becomes available. The Investigator should follow the participant with the event until resolution or stabilization of the condition. Follow-up reports (as many as required) should be completed and emailed following the same procedure above.

A final report is required once the condition is resolved or stabilized and no more information about the event is expected. The final report should be completed and emailed following the same procedure above.

The Investigator must keep a copy of all documentation related to the event in the clinical site files.

If the Investigator learns of any SAE, including death, at any other time after a participant completes the study, and he/she considers the event reasonably related to the study drug, the Investigator will promptly notify the Sponsor/MM.

It is the responsibility of the Sponsor to assess the expectedness of an SAE in order to determine whether a reported SAE fits the classification of a SUSAR. The Investigator's opinion regarding causality will be considered in the Sponsor's determination of the SAE as a SUSAR. The causality assessment given by the Investigator cannot be downgraded by the Sponsor. Both the Sponsor's and Investigator's causality assessment will be considered to determine expedited reportability of an SAE as a SUSAR.

The Investigator must also report SAEs to the IRB / EC that approved the protocol in accordance with the IRB / EC's safety reporting procedures.

The Sponsor assumes responsibility for appropriate reporting of SAEs (including SUSARs) to the regulatory authorities, in accordance with applicable regulatory requirements.

10.4. Appendix 4: Contraceptive and Barrier Guidance

WOCBP must agree not to donate ova, not to attempt to become pregnant, and, if engaging in sexual intercourse with a male partner, must agree to use a highly effective method of contraception from signing the consent form until ≥ 30 days after the last dose of study drug.

Male participants must agree not to donate sperm and, if engaging in sexual intercourse with a female partner who could become pregnant, must agree to use a highly effective method of contraception from signing the consent form until ≥ 90 days after the last dose of study drug.

A highly effective method of contraception for WOCBP is 1 of the following:

- Total abstinence from heterosexual intercourse (acceptable only if it is the participant's usual form of birth control/lifestyle choice)
- Have a biologically sterile or surgically sterile partner (i.e., a female participant's male partner has undergone effective surgical sterilization such as vasectomy) before the female participant entered the clinical trial and has obtained documentation of absence of sperm in his ejaculate, and he is the sole sexual partner of the female participant during the clinical trial
- Use of a combination of a condom by the female participant's partner and one of the following effective methods of contraception:
  - Oral or intravaginal combined (estrogen- and progestogen-containing) hormonal contraceptives
  - Oral, injectable, or implantable progestogen-only hormonal contraceptives associated with inhibition of ovulation
  - Intrauterine device or intrauterine hormone-releasing system

A highly effective method of contraception for **male participants** is 1 of the following:

a. Total abstinence from heterosexual intercourse (acceptable only if it is the participant's usual form of birth control/lifestyle choice)

b. Biologically sterile or surgically sterile (i.e., the male participant has undergone effective surgical sterilization such as vasectomy) before entering the clinical trial and has obtained documentation of absence of sperm in his ejaculate

c. Use of a combination of a condom in addition to having a female partner of childbearing potential and use 1 the following effective methods of contraception:

   a. Oral or intravaginal combined (estrogen- and progestogen-containing) hormonal contraceptives

   b. Oral, injectable, or implantable progestogen-only hormonal contraceptives associated with inhibition of ovulation

   c. Intrauterine device or intrauterine hormone-releasing system

   d. Bilateral tubal ligation/occlusion

Note: Total abstinence is defined as the strict avoidance of all forms of heterosexual activity. Periodic abstinence (e.g., calendar, ovulation, symptothermal post-ovulation methods) and withdrawal are not acceptable methods.

When it is their preferred and usual lifestyle, WOCBP or males who have a same sex partner (i.e., abstain from heterosexual intercourse) are not required to use a highly effective method of contraception.

10.5. Appendix 5: Clinical Inhibitors and Inducers

GSBR-1290 is a P-gp and CYP3A4 substrate. Because GSBR-1290 is a Biopharmaceutics Classification System (BCS) Class 4 compound (poor solubility, low permeability) and a Biopharmaceutical Drug Disposition Classification System (BDDCS) Class 4 compound (where absorptive and efflux transporters could be important), changes in transporters (i.e., P-gp) will have the largest effect on variability in GSBR-1290 exposure. Therefore, it is much more likely that P-gp inhibition could increase the exposure to GSBR-1290 and that P-gp inducers could decrease the exposure to GSBR-1290. It is also likely that GSBR-1290 is not a sensitive CYP3A4 substrate. Therefore, the following exclusion criteria will be applied to the Phase 2b study:

- P-gp inhibitors (e.g., amiodarone, azithromycin, capmatinib, carvedilol, clarithromycin, curcumin, cyclosporine, digoxin, diosmin, dronedarone, erythromycin, fexofenadine, flibanserin, fluvoxamine, glecaprevir, pibrentasvir, indinavir, itraconazole, ketoconazole, lapatinib, lopinavir, nirmatrelvir, osimertinib, piperine, quercetin, quinidine, ranolazine, rifampin, ritonavir, saquinavir, Schisandra chinensis, sofosbuvir, velpatasvir, voxilaprevir, telaprevir, tepotinib, tipranavir, valspodar, vemurafenib, verapamil) that have the potential to increase exposure to GSBR-1290.

- P-gp inducers (e.g., apalutamide, avasimibe, carbamazepine, curcumin, dan-shen [Salvia miltiorrhiza], genistein, green tea, lorlatinib, rifampin, St. John's wort, tivantinib) that have the potential to decrease exposure to GSBR-1290.

- Potent CYP3A4 inhibitors (e.g., adagrasib, boceprevir, ceritinib, clarithromycin, cobicistat, danoprevir, dasabuvir, elvitegravir, ensartinib, ensitrelvir, idelalisib, indinavir, itraconazole, josamycin, ketoconazole, lonafarnib, lopinavir, mibefradil, mifepristone, nefazodone, nelfinavir, nirmatrelvir, ombitasvir, paritaprevir, posaconazole, relacorilant, ribociclib, ritonavir, saquinavir, telaprevir, telithromycin, tipranavir, troleandomycin, tucatinib, voriconazole) that have the potential to increase exposure to GSBR-1290.
- Moderate CYP3A4 inhibitors at study start (e.g., amprenavir, aprepitant, atazanavir, berotralstat, casopitant, ciprofloxacin, clofazimine, conivaptan, crizotinib, diltiazem, dronedarone, duvelisib, erythromycin, faldaprevir, fedratinib, fluconazole, ravuconazole, grapefruit juice, imatinib, ipatasertib, isavuconazole, lefamulin, lenacapavir, letermovir, netupitant, nilotinib, nivasorexant, ritlecitinib, Schisandra sphenanthera, tofisopam, treosulfan, verapamil, voxelotor, ziritaxestat) that have the potential to increase exposure to GSBR-1290. If an acute treatment with a moderate inhibitor is required post study start and there is no alternative treatment, the Investigator will contact the Sponsor for assessment based on the GSBR-1290 doses being administered (coadministration at low doses could be feasible). Chronic concomitant treatment with moderate CYP3A4 inhibitors would not be allowed post study start.
- Potent CYP3A4 inducers (e.g., apalutamide, avasimibe, carbamazepine, enzalutamide, ivacaftor, lumacaftor, ivosidenib, lumacaftor, mitotane, phenytoin, rifampin, rifapentine, St. John's wort) that have the potential to decrease exposure to GSBR-1290.

Concomitant medications that are sensitive CYP3A4 substrates may require monitoring with or without dose reductions, according to the individual respective product labeling, as summarized below.

Table 9: CYP3A4 Inhibition: Recommendation from the Product Label of Concomitant Medications that are Sensitive CYP3A4 Substrates

Concomitant Medication	Instructions for Use with Potent or Moderate CYP3A4 Inhibitors	Instructions for Use with Weak CYP3A4 Inhibitors
Drugs that have specific recommendations when concomitantly used with a weak CYP3A inhibitor		
Darifenacin	The systemic exposure of darifenacin from Enablex extended-release tablets is increased in the presence of CYP3A4 inhibitors. The daily dose of Enablex should not exceed 7.5 mg when coadministered with potent CYP3A4 inhibitors (for example, ketoconazole, itraconazole, ritonavir, nelfinavir, clarithromycin and nefazadone).	No dosing adjustments are recommended in the presence of mild or moderate CYP3A4 inhibitors.
Lemborexant	Strong or moderate CYP3A inhibitors: Avoid concomitant use.	The maximum recommended dose is 5 mg.
Lomitapide	Strong and moderate CYP3A4 inhibitors are contraindicated with JUXTAPID. Participants must avoid grapefruit juice.	Do not exceed 30 mg daily of JUXTAPID when used concomitantly with weak CYP3A4 inhibitors.

Concomitant Medication	Instructions for Use with Potent or Moderate CYP3A4 Inhibitors	Instructions for Use with Weak CYP3A4 Inhibitors
Naloxegol	Concomitant use with strong CYP3A4 inhibitors is contraindicated.<br>Moderate CYP3A4 inhibitors: Increased naloxegol concentrations; avoid concomitant use; if unavoidable, reduce dosage to 12.5 mg once daily and monitor for adverse reactions.	Clinically significant increases in naloxegol concentrations are not expected. No dosage adjustments are necessary.
Drugs that do not distinguish between potent, moderate, or weak CYP3A4 inhibitors and recommend monitoring with or without dose reductions		
Alfentanil	Monitor participants closely at frequent intervals and consider dosage reduction of alfentanil HCl injection.	
Budesonide	If treatment with inhibitors of CYP3A4 activity (such as ketoconazole, itraconazole, ritonavir, indinavir, saquinavir, erythromycin, etc.) is indicated, reduction of the budesonide dose should be considered.	
Buspirone	Subsequent dose adjustment may be necessary and should be based on clinical assessment.	
Darunavir	Administration of darunavir and ritonavir and other drugs that inhibit CYP3A may decrease the clearance of darunavir and ritonavir and may result in increased plasma concentrations of darunavir and ritonavir.	
Felodipine	Caution should be used when CYP3A4 inhibitors are coadministered with felodipine. A conservative approach to dosing felodipine should be taken.	
Indinavir	Coadministration of CRIXIVAN and other drugs that inhibit CYP3A4 may decrease the clearance of indinavir and may result in increased plasma concentrations of indinavir. Dose reductions should be considered.	
Maraviroc	Reduce dose to 150 mg twice daily in participants receiving CYP3A4 inhibitors.	
Midazolam	Caution is advised when midazolam injection is administered concomitantly with drugs that are known to inhibit the P450-3A4 enzyme. These drug interactions may result in prolonged sedation caused by a decrease in plasma clearance of midazolam.	
Nisoldipine	Known inducer or inhibitor of CYP3A4 should be avoided in general.	
Tacrolimus	Since tacrolimus is metabolized mainly by CYP3A enzymes, drugs or substances known to inhibit these enzymes may increase tacrolimus whole blood concentrations. Monitoring of whole blood concentrations and appropriate dosage adjustments of tacrolimus are recommended.	
Drugs that have no recommendations for concomitant use of a weak CYP3A4 inhibitor (exercise caution, monitor and reduce dose as needed)		
Avanafil	Do not use STENDRA in participants taking strong CYP3A4 inhibitors.	None
Conivaptan	The coadministration of VAPRISOL with potent CYP3A inhibitors, such as ketoconazole, itraconazole, clarithromycin, ritonavir, and indinavir, is contraindicated.	None
Dasatinib	The coadministration with strong CYP3A inhibitors may increase dasatinib concentrations. Increased dasatinib concentrations may increase the risk of toxicity. Avoid concomitant use of strong CYP3A4 inhibitors. If concomitant	None

Concomitant Medication	Instructions for Use with Potent or Moderate CYP3A4 Inhibitors	Instructions for Use with Weak CYP3A4 Inhibitors
	administration of a strong CYP3A4 inhibitor cannot be avoided, consider a SPRYCEL dose reduction.	
Dronedarone	Concomitant use of a strong CYP3A inhibitor are contraindicated.	None
Eletriptan	Potent CYP3A4 inhibitors significantly increase the exposure of RELPAX. Relpax should not be used within at least 72 hours of treatment with potent CYP3A4 inhibitors.	None
Eplerenone	Because eplerenone metabolism is predominantly mediated via CYP3A4, do not use INSPRA with drugs that are strong inhibitors of CYP3A4. Reduce the starting dose for hypertension to 25 mg once daily when used with moderate CYP3A4 inhibitors (e.g., verapamil, erythromycin, saquinavir, fluconazole).	None
Ibrutinib	Avoid concomitant use of other strong CYP3A inhibitors.<br>Dose modifications of IMBRUVICA are recommended when used concomitantly with moderate CYP3A inhibitors.	None
Isavuconazonium sulfate (isavuconazole)	Contraindicate coadministration of all potent CYP3A4 inhibitors.	None
Ivabradine	The concomitant use of strong CYP3A4 inhibitors is contraindicated. Avoid concomitant use of moderate CYP3A4 inhibitors when using Corlanor.	None
Lovastatin (lactone)	Concomitant administration with strong CYP3A4 inhibitors are contraindicated.	None
Lurasidone	Strong CYP3A4 inhibitors: Recommended dosage is 10.5 mg once daily.<br>Moderate CYP3A4 inhibitors: Recommended dosage is 21 mg once daily.	None
Mobocertinib	Avoid concomitant use of EXKIVITY with strong or moderate CYP3A inhibitors. If concomitant use of a moderate CYP3A inhibitor is unavoidable, reduce the dose of EXKIVITY.	None
Quetiapine	The dose of SEROQUEL should be reduced to one-sixth of the original dose if coadministered with a strong CYP3A4 inhibitor.	None
Sildenafil	A starting dose of 25 mg of VIAGRA should be considered in participants taking erythromycin or strong CYP3A4 inhibitors.	None

Concomitant Medication	Instructions for Use with Potent or Moderate CYP3A4 Inhibitors	Instructions for Use with Weak CYP3A4 Inhibitors
Simvastatin (lactone)	Concomitant use of these drugs and any medication labeled as having a strong inhibitory effect on CYP3A4 should be avoided unless the benefits of combined therapy outweigh the increased risk.	None
Ticagrelor	Strong CYP3A inhibitors substantially increase ticagrelor exposure and thus increase the risk of dyspnea, bleeding, and other adverse events. Avoid use of strong inhibitors of CYP3A.	None
Tolvaptan	Concomitant use of tolvaptan with strong CYP3A inhibitors is contraindicated. Avoid coadministration of SAMSCA with moderate CYP3A inhibitors.	None
Triazolam	Potent inhibitors of CYP 3A that should not be used concomitantly with triazolam.	None
Vardenafil	A single 2.5 mg dose of Levitra should not be exceeded in a 24-hour period when used with potent CYP3A4 inhibitors. A single 5 mg dose should not be exceeded in a 24 hour period when used with moderate CYP3A4 inhibitors	None

Abbreviations: CYP = cytochrome P450 enzyme; HCl = hydrochloride.

10.6. Appendix 6: Evaluation of Potential Drug-Induced Liver Injury

Definition

- Hepatocellular injury (usually detected by serum ALT/AST elevations) can be caused by drugs that rarely, if ever, cause severe DILI.
- Hy's Law is an ominous indicator of severe DILI, which is defined as follows:
  - ALT/ AST > 3× ULN
  - TBL >2× ULN
  - Without initial findings of cholestasis (elevated serum ALP)
  - No other reason can be found to explain the combination of increased ALT/AST and TBL

Detection

Any laboratory testing (specific in protocol or ad hoc/additional testing). If any ad hoc testing is carried out, the information is to be added to the case report forms and database. If any other information (clinical signs/symptoms and/or laboratory data) indicates potential liver injury, liver enzyme (ALT/AST) and TBL should be obtained.

Confirmation

Any ALT/AST elevation to >3× ULN should be followed by repeat testing as soon as possible but not later than 72 hours for all usual serum measures (ALT, AST, ALP, and TBL) to confirm the abnormalities and to monitor the changes.

Close observation

All potential DILI events should be closely observed, which include the following:

- Repeating liver enzyme and serum bilirubin tests 2 or 3 times weekly. The frequency of retesting can decrease to once a week or less if abnormalities stabilize or the trial drug has been discontinued and the participant is asymptomatic.
- Obtaining a more detailed history of symptoms and prior or concurrent diseases.
- Obtaining a history of concomitant drug use (including nonprescription medications and herbal and dietary supplement preparations), recreational drug use, and special diets.
- Ruling out acute viral hepatitis types A, B, C, D, and E; autoimmune or alcoholic hepatitis; NASH; hypoxic/ischemic hepatopathy; and biliary tract disease.
- Obtaining a history of exposure to environmental chemical agents.
- Obtaining additional tests to evaluate liver function, as appropriate (e.g., INR, direct bilirubin).
- Considering gastroenterology or hepatology consultations.

Actions

The study drug should be interrupted or discontinued if one of more of these conditions occur:

- ALT or AST >8× ULN
- ALT or AST >5× ULN for more than 2 weeks
- ALT or AST >3× ULN and serum TBL > 2× ULN or INR > 1.5 × ULN without elevated serum ALP, and no other disease or condition can be found to explain the abnormalities
- ALT or AST >3× ULN with the appearance of fatigue, nausea, vomiting, right upper quadrant pain or tenderness, fever, rash, and/or eosinophilia (>5%)

Evaluation for alternative causes

The SMR and Investigator should work closely together to evaluate potential alternative causes, including, but not limited to, the following:

- Acute viral hepatitis
- Alcoholic and autoimmune hepatitis
- Hepatobiliary disorders
- MASH

- Cardiovascular causes
- Concomitant treatments

Follow-up to resolution

All participants with potential DILI should be followed until all abnormalities return to normal or to the baseline state.

Rechallenge

For any study drug interruption or discontinuation due to potential DILI, rechallenge decision should be made jointly by Investigator and Sponsor MM and based on the following:

- For confirmed Hy's Law case, rechallenge should not be attempted.
- For potential Hy's Law case with manageable alternative causes and moderate liver enzyme elevations (ALT/AST = 5× ULN), rechallenge should not be attempted unless the alternative causes are well controlled, liver function tests are back to normal or baseline, and agreed upon by both SMR and Investigator.
- For moderate ALT/AST (between 3× and 5× ULN) elevation without TBL elevation, the study drug might be restarted if liver function tests are back to normal or baseline and agreed upon by both SMR and Investigator.

Reference

Guidance for Industry DILI: Premarketing Clinical Evaluation by FDA

11. REFERENCES

APA, 2013. *Diagnostic and Statistical Manual fof Mental Disorders (DSM-5).* Washington DC: American Psychiatric Asssociation.

Apovian, C. et al., 2015. Pharmacological management of obesity: an endocrine society clinical practice guideline. *J Clin Endocrinol Metab,* pp. 100(2):342-362.

Benet, L., 2013. The role of BCS (Biopharmaceutics Classification System) and BDDCS (Biopharmaceutics Drug Disposition Classification System) in drug development. *J Pharm Sci,* pp. 102(1):34-42.

Davies, M. et al., 2022. Managment of hyperglycemia in type 2 diabetes, 2022. A consensus report by the American Diabetes Association (ADA) and the European Association for the Study of Diabetes (EASD). *Diabetes Care,* pp. 45(11):2753-2786.

Garvey, W. et al., 2016. American association of clinical endocrinologists and American college of endocrinology comprehensive clinical practice guidelines for medical care of patients with obesity. *Endoc Pract,* pp. 22 Suppl 3:1-203.

Ghoorah, K. et al., 2016. Obesity and cardiovascular outcomes: a review. *Eur Heart J Acute Cardiovasc Care,* pp. 5(1):77-85.

Gorgojo-Martinez, J. et al., 2023. Clinical recommendations to manage gastrointestinal adverse events in patients treated with Glp-1 receptor agonists: a multidisciplinary expert consensus. *Journal of Clinical Medicine,* pp. 12, 145.

Jastreboff, A. et al., 2019. Obesity as a disease: the obesity society 2018 position statement. *Obesity,* pp. 27:7-9.

Kolotkin, R., Williams, V., Ervin, C. & al, e., 2019. Validation of a new measure of quality of life in obesity trials: impact of weight on quality of life-lite clinical trials version. *Clin Obes,* 9(3), p. e12310.

Kroenke, K., Spitzer, R. & Williams, J., 2001. The PHQ-9: validity of a brief depression severity measure. *Journal of GEneral INternal Medicine,* 16(9), pp. 606-613.

Lin, P. et al., 2003. DASH Collaborative Research Group. Estimation of energy requirements in a controlled feeding trial.. *Am J Clin Nutr,* pp. 77(3):639-45.

Maruish, M., 2011. *User's manual for the SF-36v2 Health Survey.* 3rd ed. Lincoln, RI: Quality Metric Incorporated.

NIH, N. I. o. A. A. a. A., 2024. *Rethinking drinking. Alcohol and your health..* [Online]
Available at: https://www.rethinkingdrinking.niaaa.nih.gov/

NIH, N. I. o. D. a. D. a. K. D., 2021. *Overweight & obesity statistics.* [Online]
Available at: https://www.niddk.nih.gov/health-information/health-statistics/overweight-obesity

Powell-Wiley, T., Poirier, P., Burke, L. & al, e., 2021. Obesity and cardiovascular disease a scientific statement from the American Heart Association. *Circulation,* pp. 143:e984-e1010.

Project, T. C. L., 2016. *The Columbia Protocol for Research.* [Online]
Available at: https://cssrs.columbia.edu/the-columbia-scale-c-ssrs/cssrs-for-research/
[Accessed June 2024].

WHO, W. H. O., 2024. *Obesity and overweight.* [Online]
Available at: https://www.who.int/news-room/fact-sheets/detail/obesity-and-overweight

TITLE PAGE

ACCESS

Protocol Title:

A Phase 2b, Randomized, Double-blind, Placebo-controlled, Dose-range Finding Study of the Efficacy and Safety of Multiple Doses of Aleniglipron (GSBR-1290) in Participants Living with Obesity (Body Mass Index ≥ 30 kg/m^2) or Overweight (Body Mass Index ≥ 27 kg/m^2) with at Least One Weight-related Comorbidity

**Brief Title:**	GSBR-1290 Phase 2b Obesity Study
**Protocol Number:**	GSBR-1290-06
**Version:**	Amendment 6 (Version 7)
**Investigational Product:**	Aleniglipron (GSBR-1290)
**Study Phase:**	2b
**Regulatory Agency Identifier Number:**	United States IND 166912
**Sponsor Name:**	Gasherbrum Bio, Inc.
**Legal Registered Address:**	601 Gateway Blvd., Suite 900<br>South San Francisco, CA 94080
**Sponsor Medical Representative:**	[redacted]
**Approval Date:**	27 Feb 2026

Confidentiality Statement

This document is confidential and may not be used, divulged, published, photographed, or otherwise disclosed other than recipient study staff and members of the IRB/EC committee. This information cannot be used for any other purposes other than the evaluation or conduct of the clinical study without the prior consent of Gasherbrum Bio, Inc.

PRINCIPAL INVESTIGATOR SIGNATURE PAGE

Study Title:

A Phase 2b, Randomized, Double-blind, Placebo-controlled, Dose-range Finding Study of the Efficacy and Safety of Multiple Doses of Aleniglipron (GSBR-1290) in Participants Living with Obesity (Body Mass Index ≥ 30 kg/m^2) or Overweight (Body Mass Index ≥ 27 kg/m^2) with at Least One Weight-related Comorbidity

Signature of Site Principal Investigator	***Date***
Printed Name of Site Principal Investigator<br>**Institution Name:**<br><br>I, the Principal Investigator, submit this statement of commitment as evidence that I understand my responsibilities pursuant to the Code of Federal Regulations (21 CFR § 312) and International Council for Harmonization of Technical Requirements for Pharmaceuticals for Human Use E6(R2) Good Clinical Practice guidelines, as well as with any and all applicable federal, state, and/or local laws and regulations, and agree to conduct this study in accordance with the protocol referenced herein.	

SPONSOR SIGNATURE PAGE

This study will be conducted in compliance with the current approved version of the clinical study protocol, Good Clinical Practice (GCP), and applicable regulatory requirement(s).

Study Title:

A Phase 2b, Randomized, Double-blind, Placebo-controlled, Dose-range Finding Study of the Efficacy and Safety of Multiple Doses of Aleniglipron (GSBR-1290) in Participants Living with Obesity (Body Mass Index ≥30 kg/m^2) or Overweight (Body Mass Index ≥27 kg/m^2) with at Least One Weight-related Comorbidity

By my signature, I confirm that I have reviewed this protocol and find its content to be acceptable.

PLEASE REFER TO THE FINAL PAGE FOR THE ELECTRONIC SIGNATURES

[redacted] Date

TABLE OF CONTENTS

TITLE PAGE 1
PRINCIPAL INVESTIGATOR SIGNATURE PAGE 2
SPONSOR SIGNATURE PAGE 3
List of Tables 9
List of Figures 9
List of Abbreviations 10
Pharmacokinetic Abbreviations and Terms 12
1. PROTOCOL SUMMARY 13
1.1. Synopsis 13
1.2. Schema 19
1.3. Schedule of Assessments (SoA) 21
2. INTRODUCTION 28
2.1. Background 28
2.1.1. People Living with Overweight or Obesity 28
2.1.2. Therapy for People Living with Overweight or Obesity 28
2.1.3. Aleniglipron Clinical Overview 29
2.2. Study Rationale 30
2.3. Benefit/Risk Assessment 31
3. OBJECTIVES AND ENDPOINTS 32
4. STUDY DESIGN 34
4.1. Overall Design 34
4.2. Scientific Rationale for Study Design 35
4.3. Justification for Dose 36
4.4. End of Study Definition 36
5. STUDY POPULATION 37
5.1. Inclusion Criteria 37
5.2. Exclusion Criteria 38
5.3. Lifestyle Considerations 43
5.3.1. Nutrition and Good Health Instructions 43
5.3.2. Meals and Nutritional Restrictions 44
5.3.3. Caffeine and Tobacco 45

5.4. Screen Failures 45
6. STUDY INTERVENTION(S) AND CONCOMITANT THERAPY 46
6.1. Study Intervention(s) Administered 46
6.2. Dose Modification/Titration Adjustments 46
6.2.1. Dose Reduction 46
6.2.2. Dose Interruption/Hold 47
6.2.3. Management of Participants with Gastrointestinal Symptoms 47
6.3. Identification and Description of the Investigational Product 48
6.4. Supply and Labeling of Investigational Product 48
6.5. Preparation, Handling, Storage, and Accountability 49
6.6. Assignment to Study Intervention 49
6.7. Blinding and Breaking Code 50
6.8. Study Intervention Compliance 50
6.9. Continued Access to Study Intervention After the End of the Study 51
6.10. Treatment of Overdose 51
6.11. Prior and Concomitant Therapy 52
7. DISCONTINUATION OF STUDY INTERVENTION AND PARTICIPANT DISCONTINUATION/WITHDRAWAL 53
7.1. Discontinuation of Study Intervention (Investigational Product) 53
7.1.1. Liver Chemistry Stopping Criteria 54
7.1.2. QTc Stopping Criteria 54
7.2. Participant Discontinuation/Withdrawal From the Study 54
7.3. Lost to Follow-up 55
8. STUDY ASSESSMENTS AND PROCEDURES 56
8.1. Baseline Procedures 56
8.2. Participant-reported Outcomes 58
8.2.1. Electronic Diary 58
8.2.2. Impact of Weight on Quality of Life-Lite CT, Short Form-36 Acute Version, and Hunger and Satiety Questionnaires 58
8.3. Safety Assessments 58
8.3.1. Physical Examinations 59
8.3.2. Vital Signs 59
8.3.3. Electrocardiograms 59

8.3.4. Clinical Laboratory Tests 60
8.3.4.1. Clinical Laboratory Safety Tests 60
8.3.4.2. Other Clinical Laboratory Tests 60
8.3.5. Pregnancy Testing 61
8.3.6. PHQ-9 and C-SSRS Questionnaires 61
8.4. Adverse Events, Serious Adverse Events, and Other Safety Reporting 62
8.4.1. Adverse Events and Serious Adverse Events 62
8.4.2. Adverse Events of Special Interest 62
8.4.2.1. Major Adverse Cardiovascular Events 62
8.4.2.2. Heart Failure Syndrome 62
8.4.2.3. Acute Pancreatitis 62
8.4.2.4. Hypoglycemia 63
8.4.2.5. Thyroid Malignancies and C-cell Hyperplasia 63
8.4.2.6. Supraventricular Arrythmias and Cardiac Conductive Disorders 63
8.4.2.7. Hepatobiliary Disorders 63
8.4.2.8. Severe Gastrointestinal Adverse Events 64
8.4.2.9. Acute Renal Events 64
8.4.2.10. Depressive Disorder and/or Suicidal Ideation 64
8.4.2.11. Hypersensitivity 64
8.4.3. Investigator-reported Outcomes 64
8.4.3.1. Time Period and Frequency for Collecting Adverse Event and Serious Adverse Event Information 64
8.4.3.2. Method of Detecting Adverse Events and Serious Adverse Events 65
8.4.3.3. Follow-up of Adverse Events and Serious Adverse Events 65
8.4.3.4. Pregnancy 66
8.4.4. Adjudication Committee for Selected Adverse Events 66
8.4.5. Independent Data Monitoring Committee 67
8.5. Pharmacokinetics 67
8.6. Pharmacodynamics 68
8.7. Blood Sample Volumes 68
9. STATISTICAL CONSIDERATIONS 69
9.1. Analysis Sets 69
9.1.1. Open-Label Extension Analysis Set 70

9.2. Statistical Analyses 70
9.2.1. Participant Disposition 70
9.2.2. Efficacy Data 71
9.2.2.1. Primary Endpoint 71
9.2.2.2. Secondary Endpoints 71
9.2.2.3. Exploratory End Points 72
9.2.2.4. Open-Label Extension Endpoints 72
9.2.3. Demographic and Baseline Characteristics 73
9.2.4. Safety/Tolerability 73
9.2.4.1. Extent of Exposure 73
9.2.4.2. Adverse Event 73
9.2.4.3. Prior and Concomitant Medication 74
9.2.4.4. Vital Signs 74
9.2.4.5. Physical Examination Data 74
9.2.4.6. Clinical Laboratory Data 74
9.2.4.7. Electrocardiogram Data 74
9.2.5. Pharmacokinetic Data 75
9.2.5.1. Plasma Pharmacokinetic Parameters 75
9.2.6. Pharmacodynamic Data 75
9.2.7. Subgroup Analyses 75
9.3. Interim Analysis 76
9.4. Sample Size Determination 76
10. SUPPORTING DOCUMENTATION AND OPERATIONAL CONSIDERATIONS 77
10.1. Appendix 1: Regulatory, Ethical, and Study Oversight Considerations 77
10.1.1. Regulatory and Ethical Considerations 77
10.1.1.1. Investigator Responsibilities 77
10.1.1.2. Ethical Considerations 77
10.1.2. Informed Consent Process 78
10.1.3. Data Protection 78
10.1.4. Study Protocol Guidelines 78
10.1.4.1. Emergency Contact With Investigator 78
10.1.4.2. COVID-19 Preparedness 78

10.1.4.3. Protocol Deviations and Violations 79
10.1.4.4. Protocol Waivers 79
10.1.4.5. Protocol Amendments 79
10.1.5. Quality Control and Assurance 79
10.1.5.1. Study Monitoring 79
10.1.5.2. Quality Assurance 80
10.1.6. Dissemination of Clinical Study Data 81
10.1.7. Source Documents 81
10.1.8. Electronic Case Report Form 82
10.1.9. Recordkeeping and Retention of Records 82
10.1.10. Study and Site Start and Closure 83
10.1.10.1. Notification of the General Practitioner 83
10.1.10.2. Clinical Laboratory Certification and Reference Ranges 83
10.1.10.3. Study/Site Termination 83
10.1.10.4. Study Completion/Site Closure 83
10.1.10.5. EOS and Regulatory Notification 84
10.1.11. Financing and Insurance 84
10.1.12. Intellectual Property, Confidentiality, and Publications 84
10.1.12.1. Ownership 84
10.1.12.2. Confidentiality 84
10.1.12.3. Publication Policy 85
10.2. Appendix 2: Clinical Laboratory Tests 86
10.3. Appendix 3: Adverse Events and Serious Adverse Events: Definitions and Procedures for Recording, Evaluating, Follow-up, and Reporting 86
10.3.1. Definition of Adverse Event 86
10.3.2. Definition of Serious Adverse Event 87
10.3.3. Definition of Suspected Unexpected Serious Adverse Reaction 87
10.3.3.1. Assessment of Intensity 88
10.3.3.2. Assessment of Causality 88
10.3.4. Action Taken and Outcome 89
10.3.5. Recording Adverse Events and/or Serious Adverse Events 90
10.3.5.1. Follow-up of Adverse Events and Serious Adverse Events 91

10.3.6. Reporting of Serious Adverse Events, Pregnancy, and Suspected Unexpected Serious Adverse Reactions 91

10.4. Appendix 4: Contraceptive and Barrier Guidance 92

10.5. Appendix 5: Clinical Inhibitors and Inducers 93

10.6. Appendix 6: Evaluation of Potential Drug-Induced Liver Injury 98

11. REFERENCES 101

List of Tables

Table 1: ..... Overall Schedule of Assessments 21

Table 2: .....Overall Schedule of Assessments for Open-Label Extension 24

Table 3: .....Pharmacokinetic Sampling Schedule 26

Table 4: .....Pharmacokinetic Sampling Schedule for Participants Who Were on Placebo Moving into Open-Label Extension 27

Table 5: .....Study Intervention(s) Administered 46

Table 6: .....Approximate Blood Sample Volumes to be Collected per Participant 68

Table 7: .....Full Laboratory Tests 86

Table 8: .....NCI-CTCAE 5-Point Scale for Grading the Intensity of Each AE and SAE 88

Table 9: .....Relationship of the Study Treatment to an AE (Definitions) 89

Table 10: ...Terms and Definitions Used in Assessing the Final Outcome of an AE 90

Table 11: ...Excluded Medications Based on Concomitant Use of P-gp and CYP3A4 Substrates, Inhibitors, and Inducers 94

Table 12: ...CYP3A4 Inhibition: Recommendation from the Product Label of Concomitant Medications that are Sensitive CYP3A4 Substrates 95

List of Figures

Figure 1: ....Diagrammatic Representation of Double-Blind Study Design 20

Figure 2: ....Diagrammatic Representation of Open-Label Extension Study Design 20

Figure 3: ....Order of Procedures 56

List of Abbreviations

Abbreviation	Definition or Explanation
AC	Adjudication Committee
AE	adverse event
AESI	adverse event of special interest
ALP	alkaline phosphatase
ALT	alanine transaminase
AST	aspartate transaminase
BCRP	breast cancer resistance protein
BMI	body mass index
BP	blood pressure
CFR	Code of Federal Regulations
COVID-19	coronavirus disease 2019
CRO	contract research organization
CSR	clinical study report
C-SSRS	Columbia Suicide Severity Rating Scale
CYP	cytochrome P450 enzyme
DILI	drug-induced liver injury
ECG	electrocardiogram
eCRF	electronic Case Report Form
eGFR	estimated glomerular filtration rate
EOS	end of study
ETV	early termination visit
FDA	Food and Drug Administration
GCP	Good Clinical Practice
GI	gastrointestinal
GIP	glucose-dependent insulinotropic peptide
GLP-1RA	glucagon-like peptide-1 receptor agonist
HbA1c	hemoglobin A1c
HBsAg	hepatitis B surface antigen
hCG	human chorionic gonadotropin
HCV	hepatitis C virus
HDL	high-density lipoprotein

Abbreviation	Definition or Explanation
HIV	human immunodeficiency virus
HOV	healthy overweight/obese volunteer
HR	heart rate
ICE	intercurrent event
ICF	informed consent form
ICH	International Council for Harmonization of Technical Requirements for Pharmaceuticals for Human Use
IDMC	Independent Data Monitoring Committee
IND	Investigational New Drug
IP	investigational product
IRB / EC	Institutional Review Board/ Ethics Committee
IWQOL-Lite CT	Impact of Weight on Quality of Life-Lite Clinical Trials Version
LDL	low-density lipoprotein
MACE	major adverse cardiovascular event
MASH	metabolic dysfunction-associated steatohepatitis
MedDRA	Medical Dictionary for Regulatory Activities
MM	Medical Monitor
MMRM	mixed models for repeated measures
NCI-CTCAE	National Cancer Institute Common Terminology Criteria for Adverse Events
NHP	non-human primate
NLT	not less than
NOAEL	no-observed-adverse-effect level
OLE	open-label extension
PD	pharmacodynamic(s)
P-gp	P-glycoprotein
PHQ-9	Patient Health Questionnaire 9
PK	pharmacokinetic(s)
QD	once daily
QTcF	QT interval corrected using Fridericia’s formula
RA	receptor agonist
RR	respiratory rate
SAD	single-ascending dose

Abbreviation	Definition or Explanation
SAE	serious adverse event
SAP	Statistical Analysis Plan
SD	standard deviation
SMR	Sponsor Medical Representative
SoA	Schedule of Assessments
SOC	system organ class
STEMI	ST-elevated myocardial infarction
SUSAR	suspected unexpected serious adverse reaction
T2DM	type 2 diabetes mellitus
TBL	total bilirubin
TEAE	treatment-emergent adverse event
THC	tetrahydrocannabinol
ULN	upper limit of normal
US	United States
WHO	World Health Organization
WHO-DD	World Health Organization Drug Dictionary
WOCBP	women of childbearing potential

Pharmacokinetic Abbreviations and Terms

Abbreviation	Definition or Explanation
$AUC_{0\text{-}tau}$	area under the concentration-time curve from 0 to tau, where tau is the dosing period of length at steady state
C_{max}	maximum observed plasma concentration
T_{max}	time to C_{max}

1. PROTOCOL SUMMARY

1.1. Synopsis

Protocol Title: A Phase 2b, Randomized, Double-blind, Placebo-controlled, Dose-range Finding Study of the Efficacy and Safety of Multiple Doses of Aleniglipron (GSBR-1290) in Participants Living with Obesity (Body Mass Index ≥30 kg/m^2) or Overweight (Body Mass Index ≥27 kg/m^2) with at Least One Weight-related Comorbidity

Rationale:

Obesity has reached epidemic proportions in most of the world, with more than 70% of adults in the United States (US) living with overweight or obesity (NIH, 2021). The World Health Organization estimates that in 2022, 1 in 8 people in the world were living with obesity, with 2.5 billion adults (18 years and older) overweight, 890 million of those living with obesity (WHO, 2024). Obesity is associated with various cardio-renal and metabolic diseases. Patients living with obesity are 2 to 4 times more likely to develop cardiovascular disease, including heart failure. Nearly 80% of patients with type 2 diabetes mellitus (T2DM) are living with either overweight or obesity. The number of people with obesity and overweight has been increasing steadily for over 20 years and is paralleled by the rates of people developing T2DM. Glucagon-like peptide receptor agonists (GLP-1RA) are a class of incretin peptides modeled after naturally occurring glucagon-like peptide-1 which functions to reduce appetite and decrease gastric motility. In the modified form, GLP-1RAs have an extended action and show a profound effect on appetite and induction of weight loss. Although the injectable GLP-1RA class of drugs was initially developed for their ability to increase insulin secretion in response to elevated glucose levels for the treatment of hyperglycemia in patients with T2DM, it has since become the staple for the treatment of overweight/obesity. Despite this advancement in weight management, there remains a concern for the cost, cold-storage requirement, and need for subcutaneous injection of this class of drugs. Therefore, there is a need for GLP-1RAs that can maintain effective concentrations throughout the dosing interval, can be stored easily (no refrigeration), and can be administered easily (orally with few, if any, restrictions regarding food/fluid intake and concomitant medications) to address the current unmet medical need.

Aleniglipron (GSBR-1290) is a potent, orally bioavailable, non-peptide GLP-1RA being developed as an adjunct to diet and exercise, to improve glycemic control in adults with T2DM under Investigational New Drug (IND) 160966, and for chronic weight management under IND 166912.

Under IND 160966, studies in non-human primate (NHP) models were performed, which showed that aleniglipron increased glucose-dependent insulin secretion and suppressed food intake with efficacy similar to the injectable peptidic GLP-1RA liraglutide. Based on nonclinical data, aleniglipron has the potential to achieve body weight loss similar to or greater than the effects observed with other GLP-1RAs, approved or in development, via the more convenient oral route of administration.

Aleniglipron was assessed in a single-ascending dose clinical study at doses that provide exposures associated with glucose-lowering effects in NHPs. A recently completed (proof of principle) Phase 1b/2a study with aleniglipron (GSBR-1290-02) demonstrated relatively good tolerability and promising efficacy in both weight loss (in participants living with

obesity/overweight as well as participants living with T2DM) and hemoglobin A1c (HbA1c) reduction (T2DM participants).

This Phase 2b clinical study will evaluate the efficacy (body weight changes), safety, tolerability, pharmacokinetics (PK), and pharmacodynamics (PD) of various aleniglipron dose regimens compared with placebo in participants living with obesity or overweight with ≥1 weight-related comorbidity, in addition to diet and exercise, over a 36-week period. At the end of the double-blind period (after completing 36 weeks of treatment), participants will be offered to continue with an open-label extension (OLE) period where they will receive aleniglipron for an additional 36 weeks according to the schematic in Section 1.2.

Objectives and Endpoints for Double Blind Period:

	Objectives	Endpoints
**Primary**	To compare the efficacy of increasing doses of aleniglipron against placebo on body weight change	• Percent change in body weight from Baseline to Week 36
**Secondary**	To compare the efficacy of increasing doses of aleniglipron against placebo on body weight change	• Percentage of participants who achieve ≥5% reduction in body weight at Week 36<br>• Percentage of participants who achieve ≥10% reduction in body weight at Week 36<br>• Percentage of participants who achieve ≥15% reduction in body weight at Week 36<br>• Change in body weight (absolute) from Baseline to Week 36<br>• Change in waist circumference from Baseline to Week 36<br>• Change in body mass index from Baseline to Week 36
	To assess the safety and tolerability profile of a monthly titration scheme	• TEAEs and SAEs<br>• AESIs<br>• Laboratory parameters<br>• Electrocardiogram parameters<br>• Vital signs
**Exploratory**	To assess the plasma pharmacokinetics of aleniglipron and other parameters	• Change in triglycerides, FFA, and hsCRP to Week 36<br>• Change in patient-reported outcomes (SF-36 acute version, IWQOL-Lite CT, and hunger/satiety evaluation) from Baseline to Week 36

	Objectives	Endpoints
		• Change in HDL and LDL cholesterol from Baseline to Week 36<br>• Population pharmacokinetic parameters<br>• Change in resting blood pressure from Baseline to Week 36<br>• Change in fasting glucose from Baseline to Week 36

Abbreviations: AESI=adverse event of special interest; FFA=free fatty acids; HDL=high-density lipoprotein; hsCRP=high-sensitivity C-reactive protein; IWQOL-Lite CT=Impact of Weight on Quality of Life-Lite Clinical Trials Version; LDL=low-density lipoprotein; PD=pharmacodynamic; SAE=serious adverse event; SF-36=Short Form 36 health survey acute version; TEAE=treatment-emergent adverse event.

Objectives and Endpoints Open-Label Extension:

	Objectives	Endpoints
**Primary**	To evaluate long-term safety and tolerability of aleniglipron in adults with obesity or overweight with a comorbidity at Week 72	• TEAEs and SAEs<br>• AESIs<br>• Laboratory parameters<br>• Electrocardiogram parameters<br>• Vital signs
**Secondary**	Durability and maintenance of weight loss from Week 0 – 72, and Week 36 - 72	• Percent change in body weight<br>• Percentage of participants who achieve ≥5% reduction in body weight<br>• Percentage of participants who achieve ≥10% reduction in body weight<br>• Percentage of participants who achieve ≥15% reduction in body weight<br>• Change in body weight (absolute)<br>• Change in waist circumference<br>• Change in body mass index
**Exploratory**	To assess the long term effect of aleniglipron on other parameters	• Change in triglycerides, FFA, and hsCRP<br>• Change in patient-reported outcomes (SF-36 acute version, IWQOL-Lite CT, and hunger/satiety evaluation)

	Objectives	Endpoints
		• Change in HDL and LDL cholesterol<br>• Change in resting blood pressure<br>• Change in fasting glucose

Abbreviations: AESI=adverse event of special interest; FFA=free fatty acids; HDL=high-density lipoprotein; hsCRP=high-sensitivity C-reactive protein; IWQOL-Lite CT=Impact of Weight on Quality of Life-Lite Clinical Trials Version; LDL=low-density lipoprotein; PD=pharmacodynamic; SAE=serious adverse event; SF-36=Short Form 36 health survey acute version; TEAE=treatment-emergent adverse event.

Estimands:

The efficacy estimand is described by the following attributes:

Population: participants with obesity (body mass index [BMI] ≥30 kg/m^2) or overweight (BMI ≥27 kg/m^2) with at least one weight-related comorbidity who meet the inclusion criteria. Refer to Section 5.1 for more details.

Treatment: randomized treatment with titration to 1 of 3 target doses, with allowance of down-titration based on tolerability, or placebo.

Primary endpoint: percent change in body weight from Baseline to Week 36.

Intercurrent events (ICEs): permanent discontinuation of study drug.

Population-level summary: difference in percent change in body weight between aleniglipron (45, 90, 120 and 180 mg once daily [QD]) and placebo will be analyzed using a mixed model for repeated measures model with treatment group, randomization strata (baseline BMI and sex), visit, treatment-by-visit interaction, and baseline measurement as covariates. An unstructured covariance model will be used.

The primary clinical question of interest is as follows: What is the treatment difference in percent change in body weight after 36 weeks of treatment in participants who meet the inclusion criteria and would have completed the treatment period?

The primary estimand will handle the ICEs by hypothetical strategy. When an ICE occurs, subsequent data will be regarded as invalid and set to missing.

The secondary estimand will handle the ICEs by treatment policy strategy. All observed values, including those collected after ICEs, will be used in the analysis.

The same estimands for the primary objective will be used for the efficacy endpoints for the secondary objectives.

Unless specified otherwise, safety and tolerability assessments will be guided by an estimand comparing safety of aleniglipron doses with placebo irrespective of adherence to study drug, including data collected during the treatment period plus safety follow-up from participants who are randomized and are exposed to at least 1 dose of study drug.

Overall Design Synopsis:

This is a randomized, double-blind, placebo-controlled, dose-range finding study of the efficacy, safety, tolerability, PK, and PD of multiple doses of aleniglipron followed by a 36-week open

label extension in participants living with overweight or obesity with at least one weight-related comorbidity.

The study will enroll male and female participants ≥18 years and <80 years of age at the time of Screening with a BMI ≥30 kg/m^2 (obese) or BMI ≥27 kg/m^2 (overweight) with ≥1 weight-related comorbidity and who have had a stable weight (no more than 5% variation) in the last 3 months prior to Screening. Enrollment of participants with a BMI <30 kg/m^2 will be capped at 10% of the planned sample size, with at least 60% of the participants enrolled with a BMI ≥35 kg/m^2. Enrollment of female participants will also be capped to no more than 65% of the planned sample size. Efforts will be made to ensure that ethnicity and race will be approximately representative of the US population in general to ensure sufficient diversity based on 2023 US Census Data.

During the double-blind period, participants will start at a 5 mg dose of study drug (or placebo) QD for 4 weeks and will receive multiple ascending doses at 4-week intervals until they reach the maintenance dose, for a total of 36 weeks of treatment. Participants will be up-titrated at the end of Week 4 (start of Week 5), end of Week 8 (start of Week 9), etc., as shown in the Study Schema (Section 1.2) and the Schedule of Assessments (Section 1.3).

All on-study assessments to be performed are described in the Schedule of Assessments (Section 1.3). Blood samples will be collected at various times to assess biomarkers for safety monitoring as well as for PK assessments.

Participants completing the 36 weeks of treatment during the double-blind period will be offered to participate in an OLE to receive up to 180 mg aleniglipron (or the maximal tolerated dose) once daily for an additional 36 weeks (72 weeks total treatment).

Number of Participants:

Approximately 220 participants at around 40 sites in the US will be randomized. Participants not meeting the eligibility criteria at Screening will be considered a screen failure. Participants who screen fail will be allowed to be rescreened up to 2 times.

Enrollment Criteria

Full inclusion and exclusion criteria are provided in Section 5.1 and Section 5.2, respectively.

Key Inclusion Criteria:

Participants will be eligible if they satisfy ALL of the following pertinent criteria at Screening and Baseline (prior to dosing).

- Signed informed consent before any study-related activities are initiated, must be able to understand the full nature and purpose of the trial (including possible risks and adverse effects), and be willing to comply with all study procedures and adhere to the protocol schedule and restrictions
- Men and women, age ≥18 years and <80 years, with:
  - BMI ≥30 kg/m^2

    or

  - BMI ≥27.0 kg/m^2 and previous/current diagnosis of ≥1 comorbidity: hypertension, dyslipidemia, obstructive sleep apnea, cardiovascular disease, or metabolic dysfunction-associated steatohepatitis (MASH).
- Screening HbA1c <6.5%

Study Arms and Duration:

Participants will be randomized to alenigliptron or placebo in a ratio of 3:1 within each Cohort as follows:

- Cohort 1: Dose level 1 alenigliptron (maintenance dose: 45 mg QD) or placebo N=60
- Cohort 2: Dose level 2 alenigliptron (maintenance dose: 90 mg QD) or placebo N=80
- Cohort 3: Dose level 3 alenigliptron (maintenance dose: 120 mg QD) or placebo N=80

During the double-blind period, participants will receive multiple-ascending, QD doses of alenigliptron or placebo in titration steps of 4 weeks duration. Thereafter, in the last titration step dose, participants will receive the target maintenance dose (target dose) of study drug for the remaining duration of treatment (for a total of 36 weeks of treatment).

The maximum study double-blind duration for participants is 41 weeks (4 weeks for Screening, 36 weeks for treatment, and 1 week for follow-up). Participants agreeing to continue in OLE (with an amended signed consent) will either continue (participants who were on active study drug in Cohorts 1, 2, and 3) or be switched to the study drug (participants who were on placebo in Cohorts 1, 2, and 3) for an additional 36 weeks, for a maximum study duration of 77 weeks (4 weeks for Screening, 36 weeks for blinded study treatment, 36 weeks for open-label treatment, and 1 week for follow-up).

OLE: Participants who were taking 45 mg alenigliptron will receive multiple-ascending QD doses of alenigliptron in titration steps of 4 weeks duration up to 180 mg alenigliptron (the target maintenance dose) and will continue on this dose for the remaining duration of the 36 week OLE treatment period. Participants who were taking 90 mg or 120 mg of alenigliptron during the double-blind period will titrate up to reach the maintenance dose of 180 mg alenigliptron QD for the 36 additional weeks of treatment. Note that some participants may not reach the 180 mg maintenance dose if they have already completed the OLE. Participants who were taking placebo will start at a 2.5 mg QD dose (in the event of delay in availability of the 2.5 mg tablet, participants can start at 5 mg QD until the 2.5 mg tablet is available) and will receive multiple-ascending QD doses of alenigliptron in titration steps of 4 weeks duration up to 180 mg alenigliptron, and will then receive the target maintenance dose (180 mg) for the remaining duration of treatment (for a total of 36 additional weeks of treatment, including the up-titration steps). Participants in the OLE will titrate from 90 mg to 180 mg directly unless they are already at the 120 mg dose, in which case they will up titrate to 180 mg on their next visit. Participants who remain in the double-blind part of the study for 36-weeks on a lower maximum tolerated dose than the maintenance dose may enter the open-label extension at that maximum tolerated dose.

Up Titrations

At 4-week intervals, participants will be up-titrated to the next higher dose during Visit 2 (Week 5), Visit 3 (Week 9), Visit 4 (Week 13), for those in Cohorts 2 and 3 Visit 5 (Week 17),

and for those in Cohort 3 Visit 6 (Week 21). The up-titrations will continue in the OLE as described above.

Down Titration Adjustments for Adverse Events

Titration adjustments (i.e., down titrations) will be permitted upon documented discussion with the Sponsor to manage participants experiencing adverse events at the current dose during the primary treatment and OLE periods. Adjustments will include down titrating to the next lower dose, or as needed, and then re-titrating up to the target dose, in consultation between the site Investigator and the Sponsor Medical Representative (SMR). Education and coaching on tolerance strategies will be provided by qualified study personnel to enable as many participants as possible to attain their target dose. Recommendations are detailed in the Site Procedural Manual.

1.2. Schema

This is a Phase 2b, multiple-dose, randomized, double-blind, placebo-controlled, dose-range finding study with OLE. (Double-Blind Period Figure 1).

The OLE is shown in Figure 2.

Figure 1: Diagrammatic Representation of Double-Blind Study Design

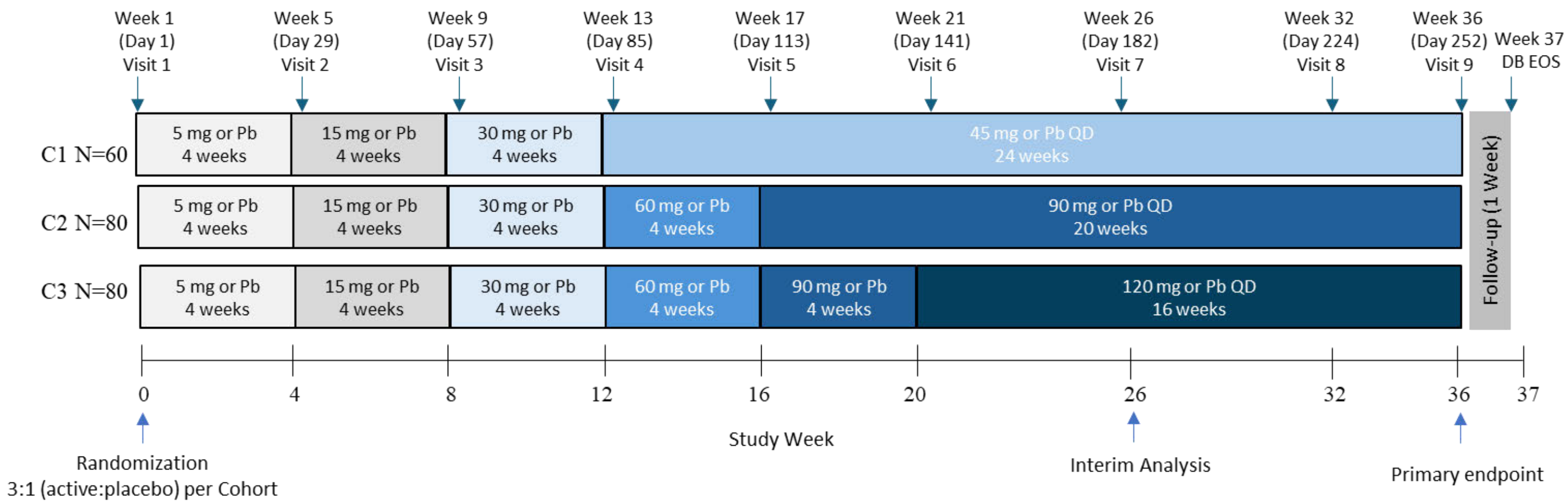

Abbreviations: C=cohort; DB=double-blind; EOS=end of study; N=number of participants; Pb=placebo; QD=once daily.
Note: At 4-week intervals, participants will be up-titrated to the next higher dose during Visit 2 (Week 5), Visit 3 (Week 9), Visit 4 (Week 13), for those in Cohorts 2 and 3 Visit 5 (Week 17), and for those in Cohort 3 Visit 6 (Week 21).

Figure 2: Diagrammatic Representation of Open-Label Extension Study Design

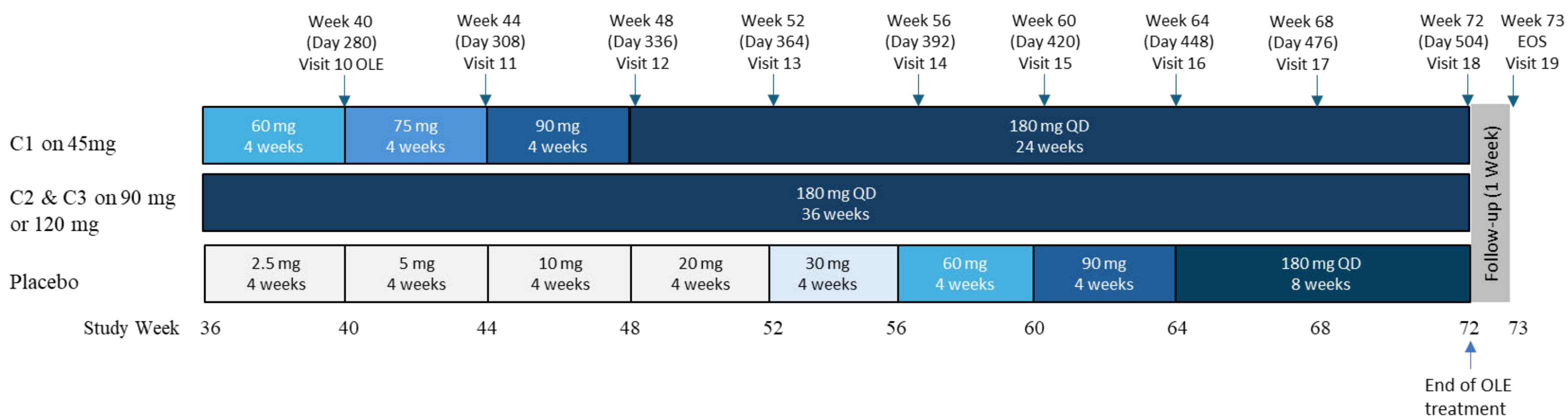

Abbreviations: C=cohort; EOS=end of study; OLE=open-label extension; QD=once daily. As of amendment 6, participants who are on 120 mg in the OLE should transition to 180 mg at their next study visit.

1.3. Schedule of Assessments (SoA)

Table 1: Overall Schedule of Assessments

Study periods	Screen	Treatment period									EOS /ETV[k]
Visit identifier	SCRN	1	2	3	4	5	6	7	8	9[n]	DB EOS
Visit day	-28 to -1	1	29	57	85	113	141	182	224	252	259
Visit week	-4 to -1	1	5	9	13	17	21	26	32	36	37
Visit Interval Window (days)		0	±3	±3	±3	±3	±3	±3	±3	±3	±3
**Assessments**											
Informed consent	X										
Eligibility criteria	X										
Demographics	X										
Medical history	X										
Drug screen	X										
Body height	X										
Body weight (with certified calibrated scales and in hospital gown) in triplicate	X	X	X	X	X	X	X	X	X	X	X
Waist circumference in triplicate		X					X		X	X	X
Concomitant medication	X	X	X	X	X	X	X	X	X	X	X
Physical examination (full)	X										X
Physical examination (symptom-directed)		X	X	X	X	X	X	X	X	X	
Vital signs (BP, HR, temperature) [a]	X	X	X	X	X	X	X	X	X	X	X
12-Lead ECG [b]	X	X	X	X	X	X	X	X	X	X	X
COVID-19 test [c]	X	X									
FSH (postmenopausal women ≤55 years)	X										
Pregnancy test (urine) before dosing [d]	X	X	X	X	X	X	X	X	X	X	X
Pregnancy test (serum) before dosing only if urine test is positive [d]	X										
Clinical laboratory tests [e]	X	X	X	X	X	X	X	X	X	X	X
Viral serology	X										
Adverse events [f]		X	X	X	X	X	X	X	X	X	X
Randomization		X									
Dose administration [g]		X	X	X	X	X	X	X	X	X	
HbA1c	X	X			X					X	

Study periods	Screen	Treatment period									EOS /ETV[k]
Visit identifier	SCRN	1	2	3	4	5	6	7	8	9[n]	DB EOS
Visit day	-28 to -1	1	29	57	85	113	141	182	224	252	259
Visit week	-4 to -1	1	5	9	13	17	21	26	32	36	37
Visit Interval Window (days)		0	±3	±3	±3	±3	±3	±3	±3	±3	±3
**Assessments**											
PK sampling (blood) [h]		X	X	X	X	X	X	X	X	X	
Cardiovascular PD biomarkers (blood)[i]		X			X					X	
Fill out electronic diary daily, including dose administration time		X	X	X	X	X	X	X	X	X	
SF-36 acute version		X					X			X	X
IWQOL-Lite CT		X					X			X	X
Hunger and satiety evaluation[j]		X	X	X	X	X	X	X	X	X	X
PHQ-9 health questionnaires	X	X	X	X	X	X	X	X	X	X	X
C-SSRS screening/baseline[l]	X										
C-SSRS since the last visit		X	X	X	X	X	X	X	X	X	X
Discuss nutrition plan and physical activity[m]	X	X	X	X	X	X	X	X	X	X	

Abbreviations: BP=blood pressure; COVID-19=coronavirus disease 2019; C-SSRS=Columbia Suicide Severity Rating Scale; DB=double-blind; ECG=electrocardiogram; EOS=end of study; ETV=early termination visit; FSH=follicle-stimulating hormone; HbA1c=hemoglobin A1c; HR=heart rate; IWQOL-Lite CT=Impact of Weight on Quality of Life-Lite Clinical Trials Version; OLE=open-label extension; PD=pharmacodynamic; PHQ-9=Patient Health Questionnaire-9; PK=pharmacokinetic; QD=once daily; SF-36=Short Form 36 health survey acute version; TSH=stimulating hormone.

a. Vital signs including blood pressure (in triplicate), heart rate, and temperature (Screening and Day 1 only) will be collected predose. BP will be collected from the left arm in at least 2-minute intervals. See Section 8 for order of procedures. Caffeine-containing products (e.g., coffee, tea, chocolate, and caffeine-containing sodas, colas, etc.) may not be consumed within 2 hours before measuring vital signs and ECGs. Predose vital signs assessments should be performed within 1 hour before dosing.
b. ECGs will be collected and stored centrally. ECGs will be collected in triplicate along with each PK draw and within 15 minutes before the PK draw. See Section 8 for order of procedures.
c. COVID-19 test only if clinically indicated or per site policy.
d. For women of child-bearing potential, urine pregnancy test is performed first, and if positive or inconclusive, confirmed with a serum pregnancy test.
e. Includes fasting glucose after Screening visit (i.e., starting on Day 1). Clinical safety laboratory tests will include hematology, serum chemistry, and coagulation. See Section 10.2. TSH is only collected during the Screening Visit. Norovirus and/or influenza testing may be conducted as standard of care as needed.
f. Adverse events and serious adverse events collected from time of signing the informed consent form through 30 days after date of the last dose.
g. On-study visit days, participants should take all of their study drug at the clinic within 45 minutes due to the PK assessments being performed on those days. At 4-week intervals, participants will be up-titrated to the next higher dose during Visit 2 (Week 5), Visit 3 (Week 9), Visit 4 (Week 13), Visit 5 (Week 17) for those in Cohorts 2 and 3, and for those in Cohort 3 Visit 6 (Week 21). Participants not moving into the OLE will receive the last dose of study drug at Visit 9 (Week 36). Participants who are moving into the OLE will receive their newly assigned dose of study drug at Visit 9 (Week 36) and will continue with QD dosing, with the next study visit occurring at Week 40 according to Table 2. See Pharmacy Manual for return of any unused study drug from the blinded portion of the study.

h. For PK timepoints, refer to Table 3. See Section 8 for order of procedures.

i. PD markers, including, but not limited to, free fatty acid, IL-6, ProBNP, high-sensitivity C-reactive protein, and succinate, will be measured at Day 1, Day 85, and Day 252.

j. The hunger and satiety evaluation will be performed for the first 7 days following each clinic visit, except for Visit 10/EOS/ETV which will be evaluated on the day of the visit only. If the hunger and satiety evaluation is received with triggering results, site staff should conduct an unscheduled phone call visit to evaluate the condition of the participant's adverse event. On Visit 1, Day 1, the hunger and satiety evaluation should be completed prior to study drug administration as a baseline measurement.

k. ETV should be scheduled 7 (±3) days from the last dose. Visit DB EOS will only occur for participants not moving on to the OLE or for those who terminate the study early. Participants moving into the OLE will have a separate Visit 10 (OLE) at Week 40 as described in Table 2.

l. C-SSRS baseline must be completed prior to any study-related activities.

m. There will be a discussion on nutrition approximately 3 days prior to each up-titration visit in addition to a documented discussion on nutrition plan and physical activity at the clinic visit.

n. Participants who will be rolling over to the OLE will re-consent at an appropriate timepoint provided there are no ongoing related AEs. They return all blinded study drug and receive their new study drug at Visit 9 (Week 36).

Note: Participants who discontinue study drug should return to the clinic at the next scheduled visit for the ETV assessments.

Table 2: Overall Schedule of Assessments for Open-Label Extension

Study periods	Treatment period									EOS /ETV[j]
Visit identifier	10 OLE	11	12	13	14	15	16	17	18	19
Visit day	280	308	336	364	392	420	448	476	504	511
Visit week	40	44	48	52	56	60	64	68	72	73
Visit Interval Window (days)	±3	±3	±3	±3	±3	±3	±3	±3	±3	±3
Assessments										
Body weight (with certified calibrated scales and in hospital gown) in triplicate	X	X	X	X	X	X	X	X	X	X
Waist circumference in triplicate						X			X	X
Concomitant medication	X	X	X	X	X	X	X	X	X	X
Physical examination (full)										X
Physical examination (symptom-directed)	X	X	X	X	X	X	X	X	X	
Vital signs (BP, HR, temperature) [a]	X	X	X	X	X	X	X	X	X	X
12-Lead ECG [b]	X	X	X	X	X	X	X		X	X
Pregnancy test (urine) before dosing [c]	X	X	X	X	X	X	X	X	X	X
Clinical laboratory tests [d]	X	X	X	X	X	X	X	X	X	X
Adverse events [e]	X	X	X	X	X	X	X	X	X	X
Dose administration [f]	X	X	X	X	X	X	X	X	X	
HbA1c						X			X	
PK sampling (blood) only for participants who were on placebo [g]	X									
Cardiovascular PD biomarkers (blood)[h]						X			X	
Fill out electronic diary daily, including dose administration time	X	X	X	X	X	X	X	X	X	
SF-36 acute version				X					X	X
IWQOL-Lite CT				X					X	X
Hunger and satiety evaluation[i]	X	X	X	X	X	X	X	X	X	X
PHQ-9 health questionnaires	X	X	X	X	X	X	X	X	X	X
C-SSRS since the last visit	X	X	X	X	X	X	X	X	X	X
Discuss nutrition plan and physical activity[k]	X	X	X	X	X	X	X	X	X	

Abbreviations: BP=blood pressure; C-SSRS=Columbia Suicide Severity Rating Scale; ECG=electrocardiogram; EOS=end of study; ETV=early termination visit; HbA1c=hemoglobin A1c; HR=heart rate; IWQOL-Lite CT=Impact of Weight on Quality of Life-Lite Clinical Trials Version; OLE=open-label extension; PD=pharmacodynamic; PHQ-9=Patient Health Questionnaire-9; PK=pharmacokinetic; SF36=Short Form 36 health survey acute version.

a. Vital signs including blood pressure (in triplicate), heart rate, and temperature (temperature at Screening and Day 1only) will be collected predose. BP will be collected from the left arm in at least 2-minute intervals. See Section 8 for order of procedures. Caffeine-containing products (e.g., coffee, tea, chocolate, and caffeine-containing sodas, colas, etc.) may not be consumed within 2 hours before measuring vital signs and ECGs. Predose vital signs assessments should be performed within 1 hour before dosing.

b. ECGs will be collected predose and stored centrally. At Week 40, ECGs will be collected in triplicate along with each PK draw and within 15 minutes before the PK draw. See Section 8 for order of procedures.

c. For women of child-bearing potential, a urine pregnancy test is performed first, and if positive or inconclusive, confirmed with a serum pregnancy test.

d. Clinical safety laboratory tests will include hematology, serum chemistry, and coagulation. See Section 10.2. Norovirus and/or influenza testing may be conducted as standard of care as needed.

e. Adverse events and serious adverse events collected from time of signing the informed consent form through 30 days after date of the last dose.
f. Up-titrations will occur at Weeks 40, 44, and 48 for participants rolling over to the OLE from Cohort 1, and at Week 40, 44, 48, 52, 56, 60, and 64 for those who were on placebo. Participants already on 120 mg in the OLE should titrate up to 180 mg at their next scheduled visit.
g. For PK timepoints, refer to Table 4. See Section 8 for order of procedures.
h. PD markers, including, but not limited to, free fatty acid, IL-6, ProBNP, high-sensitivity C-reactive protein, and succinate, will be measured.
i. The hunger and satiety evaluation will be performed for the first 7 days following each clinic visit, except for /EOS/ETV which will be evaluated on the day of the visit only. If the hunger and satiety evaluation is received with triggering results, site staff should conduct an unscheduled phone call visit to evaluate the condition of the participant's adverse event.
j. Participants who discontinue study drug early will be allowed to remain in the study to be evaluated for all planned efficacy and safety measures at scheduled visits through EOS (except PK, ECG, daily diary, and Hunger and Satiety assessments), but at a minimum should complete the EOS/ETV as soon as possible. Any participant who withdraws consent from the study should have all scheduled EOS/ETV assessments performed as soon as possible.
k. There will be a discussion on nutrition approximately 3 days prior to each up-titration visit in addition to a documented discussion on nutrition plan and physical activity at the clinic visit.

Table 3: Pharmacokinetic Sampling Schedule

Study Day	Study Week	Planned Dose (Irrespective of Down-Titration)	PK Timepoints (Exact Time of Blood Collection Must be Recorded)	ECG
Day 1	Week 1	All cohorts: start of 5 mg	Within 45 minutes before dosing<br>Between 2 and 4 hours postdose	Yes
Day 29	Week 5	All cohorts: start of 15 mg	Within 45 minutes before dosing<br>Between 2 and 4 hours postdose	Yes
Day 57	Week 9	All cohorts: start of 30 mg	Within 45 minutes before dosing<br>Between 2 and 4 hours postdose	Yes
Day 85	Week 13	Cohort 1 – start of 45 mg<br>Cohort 2 and 3 – start of 60 mg	Within 45 minutes before dosing<br>Between 2 and 4 hours postdose	Yes
Day 113	Week 17	Cohort 1 – after 4 weeks at 45 mg<br>Cohort 2 and 3 – start of 90 mg	Within 45 minutes before dosing<br>Between 2 and 4 hours postdose	Yes
Day 141	Week 21	Cohort 1 – after 8 weeks at 45 mg<br>Cohort 2 – after 4 weeks at 90 mg<br>Cohort 3 – start of 120 mg	Within 45 minutes before dosing<br>Between 2 and 4 hours postdose	Yes
Day 182	Week 26	At steady state for all cohorts	Within 45 minutes before dosing	Yes
Day 224	Week 32	At steady state for all cohorts	Within 45 minutes before dosing	Yes
Day 252	Week 36	Last day of dosing for participants not moving to the open-label extension	Within 45 minutes before dosing<br>Between 2 and 4 hours postdose	Yes

Abbreviations: ECG=electrocardiogram; PK=pharmacokinetic.

Note: On-study days with PK assessments, all study tablets should be consumed at the clinic within 45 minutes.
Note: ECGs will be collected in triplicate, along with each PK draw and within 15 minutes before the PK draw. See the Site Procedural Manual for details.

Note: To verify compliance with the study dosing regimen and adherence to the protocol requirements, random PK blood draws may be conducted on enrolled participants to monitor the concentration of the study drug in the bloodstream.

Table 4: Pharmacokinetic Sampling Schedule for Participants Who Were on Placebo Moving into Open-Label Extension

Study Day	Study Week	Planned Dose (Irrespective of Down-Titration)	PK Timepoints (Exact Time of Blood Collection Must be Recorded)	ECG
Day 280	Week 40	Last dose of 2.5 mg QD (will be up titrated to 5 mg on Day 281)	Within 45 minutes before dosing<br>Between 2 and 4 hours post dose	Yes

Abbreviations: ECG=electrocardiogram; PK=pharmacokinetic; QD=once daily.

Note: On-study days with PK assessments, all study tablets should be consumed at the clinic within 45 minutes.
Note: ECGs will be collected in triplicate, along with each PK draw and within 15 minutes before the PK draw. See the Site Procedural Manual for details.

2. INTRODUCTION

2.1. Background

Aleniglipron is an orally available glucagon-like peptide-1 receptor agonist (GLP-1RA) being developed as an adjunct to diet and exercise, to improve glycemic control in adult participants with type 2 diabetes mellitus (T2DM) (Investigational New Drug [IND] 160966), and for chronic weight management (IND 166912).

2.1.1. People Living with Overweight or Obesity

Obesity is a multifactorial disease with a complex pathogenesis in which biological, social, and environmental factors play a critical role (Powell-Wiley, et al., 2021). In addition, people living with overweight, or obesity can present with multiple other adverse health outcomes associated with a heterogeneity in the pathophysiologic pathways and biological mechanisms (Jastreboff, et al., 2019). Patients with a high body mass index (BMI) present with an increased risk of cardiovascular and all-cause mortality and higher incidence of major comorbid conditions, such as type 2 diabetes, hypertension, dyslipidemia, and cardiovascular diseases (Ghoorah, et al., 2016). The World Health Organization (WHO) defines overweight as a BMI ≥25 and <30 kg/m^2 and obesity as a BMI ≥30 kg/m^2 (WHO, 2024).

People living with overweight and obesity are highly prevalent in the general population. In the United States (US) and according to 2017 to 2018 data from the National Health and Nutrition Examination Survey, nearly 1 in 3 adults (30.7%) are overweight and more than 2 in 5 adults (42.4%) are obese (NIH, 2021). In addition, about 1 in 11 adults (9.2%) have severe obesity (BMI >35 kg/m^2) (NIH, 2021). The WHO estimates that in 2022, 1 in 8 people in the world were living with obesity (WHO, 2024)

2.1.2. Therapy for People Living with Overweight or Obesity

Multiple medical societies have issued treatment recommendations for the treatment of patients living with overweight or obesity, depending on the obesity severity (as approximated by BMI) and associated risk factors (T2DM, previous atherosclerotic cardiovascular disease, and others) (Apovian, et al., 2015; Garvey, et al., 2016). The Endocrine Society recommends that diet, exercise, and behavioral modification be included in all obesity management approaches for patients with BMI ≥25 kg/m^2 and that other tools such as pharmacotherapy be reserved for patients with BMI ≥27 kg/m^2 with comorbidity or BMI >30 kg/m^2. Behavioral modification to reduce food intake, increase physical activity, and reduce sedentariness should be implemented in addition to pharmacotherapies and/or bariatric surgery. As with other chronic disease states, the persistence and maintenance of therapeutic strategy is key to improve long-term outcomes. To achieve this goal, it is recommended to use approved weight loss medications that may also enhance adherence to behavioral changes.

While greater weight loss may be associated with greater health benefits, even a modest 5% to 10% body weight loss, such as that produced by lifestyle modifications and medications, has been shown to produce significant improvements in the control of T2DM (Apovian, et al., 2015; Davies, et al., 2022).

It is recommended that the patient's response to a pharmacologic or other intervention be monitored (both in terms of efficacy and tolerability/safety) on a regular basis (approximately every 3 months). Interventions that yield a weight loss reduction <5% may be ineffective, and on a case-by-case basis, the patient in consultation with the physician should evaluate potential alternatives (Apovian, et al., 2015).

The majority of approved therapies for obesity target central nervous system appetite centers. They work primarily in the arcuate nucleus to promote satiety. There are several classes of drugs in that category (serotoninergic, dopaminergic, or norepinephrine-releasing agents/reuptake inhibitors). Other classes of drugs include lipase inhibitor (orlistat), and most recently, GLP-1RAs (liraglutide and semaglutide), and the GLP-1RA + glucose-dependent insulinotropic peptide (GIP) tirzepatide.

2.1.3. Aleniglipron Clinical Overview

A detailed description of the chemistry, pharmacology, efficacy, and safety of aleniglipron is provided in the IB.

Phase 1

The clinical pharmacology, PK, and PD of aleniglipron were initially evaluated in Phase 1 Study GSBR-1290-01 in healthy participants, Phase 1b/2a Study GSBR-1290-02 in participants with and without T2DM, Phase 1 Study GSBR-1290-03 in Japanese and non-Japanese healthy participants, and Phase 1 study GSBR-1290-05 in participants with overweight or obesity. In addition, Phase 1 Study GBSR-1290-07 was conducted to assess the mass balance, PK, metabolism, and excretion of aleniglipron containing a microtracer dose of [^{14}C]-aleniglipron in healthy adult participants, and Phase 1 Study GSBR-1290-08 compared 4 different aleniglipron formulations in healthy participants living with overweight or obesity.

Results from these studies demonstrated a PK profile appropriate for once daily oral dosing, the totality of available PK data across studies supports dose proportionality overall, and PD results showed clinically relevant reductions in body weight and improvements in HbA1c.

Ongoing Phase 1 studies include: GSBR-1290-10, a Phase 1b randomized, open-label study to assess the safety and tolerability of changing from once weekly subcutaneous semaglutide or tirzepatide to once daily oral aleniglipron; Study GSBR-1290-11 an open-label study to evaluate the drug-drug interactions (DDI) and delayed gastric emptying of aleniglipron in healthy participants or in healthy overweight or obese participants; Study GSBR-1290-18, an open-label study to support the formulation bridging and food-effect on aleniglipron in participants with obesity or overweight,

Phase 2

Two Phase 2 studies have evaluated the safety, tolerability, and efficacy of aleniglipron in participants with obesity or overweight and at least 1 weight-related comorbidity.

GSBR-1290-09 (ACCESS II) was a Phase 2 study to assess the safety, tolerability, and efficacy of 44 weeks of treatment with aleniglipron at target maintenance doses of 120, 180, and 240 mg QD compared to placebo.

In Phase 2 clinical studies, aleniglipron demonstrated clinically meaningful weight reduction and metabolic effects consistent with the GLP-1 receptor agonist class, while maintaining a safety and tolerability profile aligned with known GLP-1–mediated adverse events.

Ongoing Phase 2 studies include GSBR-1290-16, a Phase 2 randomized, double-blind, placebo-controlled study evaluating the effects of aleniglipron on body composition in participants living with obesity or overweight, and GSBR-1290-17, a Phase 2, randomized, double-blind, placebo-controlled study of the safety and tolerability of aleniglipron in adult participants with T2DM living with obesity or overweight.

2.2. Study Rationale

Obesity has reached epidemic proportions in most of the world, with more than 70% of adults in the US living with overweight or obesity (NIH, 2021). The World Health Organization estimates that in 2022, 1 in 8 people in the world were living with obesity, with 2.5 billion adults (18 years and older) overweight, 890 million of those living with obesity (WHO, 2024). Obesity is associated with various cardio-renal and metabolic diseases. Patients living with obesity are 2 to 4 times more likely to develop cardiovascular disease, including heart failure. Nearly 80% of patients with T2DM are living with either overweight or obesity. The number of people with obesity and overweight has been increasing steadily for over 20 years and is paralleled by the rates of people developing T2DM. GLP-1RAs are a class of incretin peptides modeled after naturally occurring glucagon-like peptide-1 which functions to reduce appetite and decrease gastric motility. In the modified form, GLP-1RAs have an extended action and show a profound effect on appetite and induction of weight loss. Although the injectable GLP-1RA class of drugs was initially developed for their ability to increase insulin secretion in response to elevated glucose levels for the treatment of hyperglycemia in patients with T2DM, it has since become the staple for the treatment of overweight/obesity. Despite this advancement in weight management, there remains a concern for the cost, cold-storage requirement, and need for subcutaneous injection of this class of drugs. Therefore, there is a need for GLP-1RAs that can maintain effective concentrations throughout the dosing interval, can be stored easily (no refrigeration), and can be administered easily (orally with few, if any, restrictions regarding food/fluid intake and concomitant medications) to address the current unmet medical need.

Aleniglipron (GSBR-1290) is a potent, orally bioavailable, non-peptide GLP-1RA being developed as an adjunct to diet and exercise, to improve glycemic control in adults with T2DM under IND 160966, and for chronic weight management under IND 166912.

Under IND 160966, studies in non-human primate (NHP) models were performed, which showed that aleniglipron increased glucose-dependent insulin secretion and suppressed food intake with efficacy similar to the injectable peptidic GLP-1RA liraglutide. Based on nonclinical data, aleniglipron has the potential to achieve body weight loss similar to or greater than the effects observed with other GLP-1RAs, approved or in development, via the more convenient oral route of administration.

Aleniglipron was well tolerated in a SAD clinical study at doses that provide exposures associated with glucose-lowering effects in NHPs. A recently completed (proof of principle) Phase 1b/2a study with aleniglipron (GSBR-1290-02) demonstrated relatively good tolerability

and promising efficacy in both weight loss (in participants living with obesity/overweight as well as participants living with T2DM) and hemoglobin A1c (HbA1c) reduction (T2DM participants).

This Phase 2b clinical study will evaluate the efficacy (body weight changes), safety, tolerability, PK, and PD of various aleniglipron dose regimens compared with placebo in participants living with obesity or overweight with ≥1 weight-related comorbidity, in addition to diet and exercise, over a 36-week period. An open-label extension (OLE) will provide an opportunity to collect safety data beyond 36 weeks while providing an opportunity for participants to be on the maximum dose of aleniglipron (180 mg).

2.3. Benefit/Risk Assessment

Aleniglipron has been investigated across both clinical and nonclinical studies, which have demonstrated its potential as an oral GLP-1RA for chronic weight management in patients with obesity or overweight.

Results from the 36-week double blind part of this Phase 2b GSBR-1290-06 study demonstrated that aleniglipron achieved a clinically meaningful and statistically significant placebo-adjusted mean weight loss of 11.3% (27.3 lbs, p<0.0001) at the 120 mg dose at 36 weeks and across all active arms had a 10.4% adverse event (AE)-related treatment discontinuation rate.

Interim results of the Phase 2 study GSBR-1290-09, which included an Independent Data Monitoring Committee review of the data, demonstrated that aleniglipron achieved a placebo-adjusted mean weight loss of 14.4% (34.4 lbs, p<0.0001) at the 180 mg dose and 15.3% (35.5 lbs, p<0.0001) with the 240 mg dose at 36 weeks. Aleniglipron demonstrated a tolerability profile consistent with the GLP-1 receptor agonist class.

The most common AEs with aleniglipron are consistent with other GLP-1RAs (approved or in development), including mild to moderate gastrointestinal (GI) AEs (nausea, vomiting, diarrhea, and constipation). These AEs are considered manageable through the implemented dose escalation scheme, nutritional guidance, and dose modification guidelines.

Overall, results from the above Phase 2 studies demonstrated that aleniglipron has a tolerability profile consistent with the GLP-1 receptor agonist class and did not demonstrate significant off-target safety concerns.

Based on assessment of the overall data to date, the benefit/risk assessment supports the investigation of doses up to 180 mg in the OLE portion of this Phase 2 study to evaluate the efficacy and safety of QD oral aleniglipron for chronic weight management in adults with obesity or overweight and a weight-related comorbidity. More detailed information about the known and expected benefits and risks and expected AEs of aleniglipron may be found in the Investigator's Brochure.

3. OBJECTIVES AND ENDPOINTS

Objectives and Endpoints for Double-Blind Period:

	Objectives	Endpoints
**Primary**	To compare the efficacy of increasing doses of aleniglipron against placebo on body weight change	• Percent change in body weight from Baseline to Week 36
**Secondary**	To compare the efficacy of increasing doses of aleniglipron against placebo on body weight change	• Percentage of participants who achieve ≥5% reduction in body weight at Week 36<br>• Percentage of participants who achieve ≥10% reduction in body weight at Week 36<br>• Percentage of participants who achieve ≥15% reduction in body weight at Week 36<br>• Change in body weight (absolute) from Baseline to Week 36<br>• Change in waist circumference from Baseline to Week 36<br>• Change in body mass index from Baseline to Week 36
	To assess the safety and tolerability profile of a monthly titration scheme	• TEAEs and SAEs<br>• AESIs<br>• Laboratory parameters<br>• Electrocardiogram parameters<br>• Vital signs
**Exploratory**	To assess the plasma pharmacokinetics of aleniglipron	• Change in triglycerides, FFA, and hsCRP at Week 36<br>• Change in patient-reported outcomes (SF-36 acute version, IWQOL-Lite CT, and hunger/satiety evaluation) from Baseline to Week 36<br>• Change in HDL and LDL cholesterol from Baseline to Week 36<br>• Population pharmacokinetic parameters<br>• Change in resting blood pressure from Baseline to Week 36<br>• Change in fasting glucose from Baseline to Week 36<br>• Open-label extension: weight loss up to 72 weeks

Abbreviations: AESI=adverse event of special interest; FFA=free fatty acids; HDL=high-density lipoprotein; hsCRP=high-sensitivity C-reactive protein; IWQOL-Lite CT=Impact of Weight on Quality of Life-Lite Clinical Trials Version; LDL=low-density lipoprotein; PD=pharmacodynamic; SAEs=serious adverse events; SF-36=Short Form 36 health survey acute version; TEAE=treatment-emergent adverse event.

Objectives and Endpoints Open-Label Extension:

	**Objectives**	**Endpoints**
**Primary**	To evaluate long-term safety and tolerability of aleniglipron in adults with obesity or overweight with a comorbidity at Week 72	• TEAEs and SAEs<br>• AESIs<br>• Laboratory parameters<br>• Electrocardiogram parameters<br>• Vital signs
**Secondary**	Durability and maintenance of weight loss from Week 0 – 72, and Week 36 - 72	• Percent change in body weight<br>• Percentage of participants who achieve ≥5% reduction in body weight<br>• Percentage of participants who achieve ≥10% reduction in body weight<br>• Percentage of participants who achieve ≥15% reduction in body weight<br>• Change in body weight (absolute)<br>• Change in waist circumference<br>• Change in body mass index
**Exploratory**	To assess the long term effect of aleniglipron on other parameters	• Change in triglycerides, FFA, and hsCRP<br>• Change in patient-reported outcomes (SF-36 acute version, IWQOL-Lite CT, and hunger/satiety evaluation)<br>• Change in HDL and LDL cholesterol<br>• Change in resting blood pressure<br>• Change in fasting glucose

Abbreviations: AESI=adverse event of special interest; FFA=free fatty acids; HDL=high-density lipoprotein; hsCRP=high-sensitivity C-reactive protein; IWQOL-Lite CT=Impact of Weight on Quality of Life-Lite Clinical Trials Version; LDL=low-density lipoprotein; PD=pharmacodynamic; SAE=serious adverse event; SF-36=Short Form 36 health survey acute version; TEAE=treatment-emergent adverse event.

4. STUDY DESIGN

4.1. Overall Design

This is a randomized, double-blind, placebo-controlled, dose-range finding study of the efficacy, safety, tolerability, PK, and PD of multiple doses of aleniglipron in participants living with obesity or overweight with at least 1 weight-related comorbidity followed by a 36-week open label extension. The starting doses and titration schema are based on results from the SAD study (GSBR-1290-01), the multiple-ascending dose study (GSBR-1290-02), the ethno-bridging PK study (GSBR-1290-03), as well as the formulation PK bridging and titration optimization study (GSBR-1290-05).

The study will enroll male and female participants ≥18 years and <80 years of age at the time of Screening with a BMI ≥30 kg/m^2 (obese) or BMI ≥27 kg/m^2 (overweight) with ≥1 weight-related comorbidity and who have had a stable weight (no more than 5% variation) in the last 3 months prior to Screening. Enrollment of participants with a BMI <30 kg/m^2 will be capped at 10% of the planned sample size, with at least 60% of the participants enrolled with a BMI ≥35 kg/m^2. Enrollment of female participants will also be capped to no more than 65% of the planned sample size. Efforts will be made to ensure that ethnicity and race will be approximately representative of the US population in general to ensure sufficient diversity based on 2023 US Census Data.

The total number of participants is anticipated to be approximately 220 at around 40 sites in the US. Participants not meeting the eligibility criteria at Screening will be considered a screen failure, with the exception of protocol specific and qualifying laboratory results not available on Day -1. Participants who screen fail will be allowed to be rescreened up to 2 times. If abnormal values are obtained for vital signs, ECG, clinical laboratory tests, viral serology, or drugs of abuse assessments in the first instance, that assessment may be repeated once (at the discretion of the Investigator and Sponsor to confirm eligibility).

All on-study assessments to be performed are described in the Schedule of Assessments (SoA) (Section 1.3).

Participants in the double-blind period will be randomized to aleniglipron or placebo in a ratio of 3:1 within each cohort as follows:

- Cohort 1: Dose level 1 aleniglipron (45 mg QD) or placebo N=60
- Cohort 2: Dose level 2 aleniglipron (90 mg QD) or placebo N=80
- Cohort 3: Dose level 3 aleniglipron (120 mg QD) or placebo N=80

Participants will receive multiple-ascending doses of aleniglipron or placebo for 36 weeks in titration steps of 4 weeks per step. Thereafter, in the last titration step dose, participants will receive the target dose of aleniglipron or placebo of their assigned cohort for the remaining duration of treatment (Figure 1).

Participants receiving placebo treatment will be included in each of the 3 cohorts in order to reduce the pill burden, improve logistics and instructions for participants, and to potentially facilitate higher compliance.

The maximum study double-blind duration for participants is 41 weeks (4 weeks for Screening, 36 weeks for treatment, and 1 week for follow-up). Participants agreeing to continue in OLE (with an amended signed consent) will either continue (participants who were on active study drug in Cohorts 1, 2, or 3) or be switched to the study drug (participants who were on placebo in Cohorts 1, 2, or 3) for an additional 36 weeks, for a maximum study duration of 77 weeks (4 weeks for Screening, 36 weeks for blinded study treatment, 36 weeks for open-label treatment, and 1 week for follow-up).

OLE: Participants who were taking 45 mg aleniglipron will receive multiple-ascending QD doses of aleniglipron in titration steps of 4 weeks duration up to 180 mg aleniglipron (the target maintenance dose) and will continue on this dose for the remaining duration of the 36 week OLE treatment period. Participants who were taking 90 mg or 120 mg of aleniglipron during the double-blind period will titrate up to reach the maintenance dose of 180 mg aleniglipron QD for the 36 additional weeks of treatment. Note that some participants may not reach the 180 mg maintenance dose if they have already completed the OLE. Participants who were taking placebo will start at a 2.5 mg QD dose (in the event of delay in availability of the 2.5 mg tablet, participants can start at 5 mg QD until the 2.5 mg tablet is available) and will receive multiple-ascending QD doses of aleniglipron in titration steps of 4 weeks duration up to 180 mg aleniglipron, and will then receive the target maintenance dose (180 mg) for the remaining duration of treatment (for a total of 36 additional weeks of treatment, including the up-titration steps). Participants in the OLE will titrate from 90 mg to 180 mg directly unless they are already at the 120 mg dose, in which case they will up titrate to 180 mg on their next visit. Participants who remain in the double-blind part of the study for 36-weeks on a lower maximum tolerated dose than the maintenance dose may enter the open-label extension at that maximum tolerated dose.

4.2. Scientific Rationale for Study Design

Study GSBR-1290-06 is a Phase 2b double-blind period of this study is designed to compare the ability of aleniglipron to reduce body weight (dose ranging from 45 to 120 mg) compared to placebo during a 36-week treatment period, in participants living with obesity, or overweight with at least 1 weight-related comorbidity and without diabetes mellitus. The double-blind period primary endpoint is percent change in body weight from Baseline to Week 36. Evaluation of the primary endpoint at 36 weeks is considered adequate for evaluation of weight loss efficacy in a Phase 2b trial and will allow evaluation of exposure-response parameters for selection of dose levels and dose escalation schemes for future Phase 3 studies.

In addition, safety, and tolerability over a range of doses will enhance the benefit-risk characterization of aleniglipron.

A placebo group in each cohort will be included to allow evaluation of the efficacy and safety attributable to aleniglipron in this population. A 4-week titration scheme was selected to enhance tolerability before escalation to the next higher dose level.

All participants will receive nutrition plan and physical activity counseling throughout the study.

4.3. Justification for Dose

This study is planned to evaluate a range of maintenance aleniglipron doses (45, 90, and 120 mg daily) compared to placebo to optimize efficacy (% change in body weight) at 36 weeks balanced with the need for a low starting dose and titration schemes to minimize known GI-related events associated with GLP-1RAs.

A starting aleniglipron dose of 5 mg was selected to minimize the GI-related tolerability effects (e.g., nausea and emesis), which are more likely to be observed at starting doses ≥10 mg. Based on the results from the completed and ongoing clinical studies with aleniglipron, the starting dose of 5 mg is expected to be safe and tolerable. For the open-label extension period, a starting aleniglipron dose of 2.5 mg for participant who had been on placebo was selected to see if there are further improvements in tolerability at the start of treatment in relation to the 5 mg starting dose used in the double blind period.

This Phase 2b study plans for slow, 4-week titration steps to potentially further improve tolerability.

Maintenance doses in the range of 45 to 120 mg were selected for the double-blind period of the study aiming to characterize the exposure response. These doses are expected to be safe and well tolerated based on the available clinical data (up to 120 mg) and based on nonclinical safety studies (6-month Good Laboratory Practice toxicity in rats).

As of protocol amendment 6 of this study, the dose of 180 mg of aleniglipron is being added to the OLE period of the study. This dose was selected based on interim analysis data from Study GSBR-1290-09 that demonstrated greater placebo-adjusted observed body weight change of -14.4% (95% CI -19.1% to -9.6%) compared with the 120 mg dose (-14.1%) evaluated in the same study at Week 36. Additionally, there was no increase in the prevalence of GI AEs, no participants who discontinued due to an AE, and no new safety signals at doses above 120mg (180mg and 240mg).

4.4. End of Study Definition

The end of study (EOS) is defined as the date of the last contact of the last participant involved in the study.

A participant is considered to have completed the study if the participant has completed all periods of the study, including the EOS visit.

If a participant meets the criteria of discontinuation of study (Section **7.2**) the study intervention should be permanently discontinued. The participant should return to the clinic as soon as possible for the Early Termination Visit (ETV) assessments which will be the EOS.

Participants who discontinue study drug early will be allowed to remain in the study to be evaluated for all planned efficacy and safety measures at scheduled visits through EOS (except PK, ECG, daily diary, and Hunger and Satiety assessments), but at a minimum should complete the ETV as soon as possible.

5. STUDY POPULATION

Prospective approval of protocol deviations to recruitment and enrollment criteria, also known as protocol waivers or exemptions, is not permitted.

This study will enroll male and female participants ≥18 years and <80 years of age at the time of screening. All participants will have a BMI ≥27.0 kg/m^2. Enrollment of participants with a BMI <30 kg/m^2 will be capped at 10% of the planned sample size, with at least 60% of the participants enrolled with a BMI ≥35 kg/m^2. Enrollment of female participants will also be capped to no more than 65% of the planned sample size. Efforts will be made to ensure that ethnicity and race will be approximately representative of the US population in general to ensure sufficient diversity based on 2023 US Census Data.

5.1. Inclusion Criteria

Participants are eligible to be included in the study only if all the following criteria apply at Screening and Baseline (prior to dosing). If abnormal values are obtained for vital signs, ECG, clinical laboratory tests, viral serology, or drugs of abuse assessments in the first instance, assessment may be repeated once (at the discretion of the Investigator and Sponsor) to confirm eligibility.

1. Signed informed consent before any study-related activities are initiated, must be able to understand the full nature and purpose of the trial (including possible risks and adverse effects), and be willing to comply with all study procedures and adhere to the protocol schedule and restrictions.
2. Men and women, age ≥18 years and <80 years, with:
   - BMI ≥30 kg/m^2 or
   - BMI ≥27.0 kg/m^2 and previous/current diagnosis of ≥1 comorbidity:
     - Hypertension (as defined by a resting systolic blood pressure [BP] of ≥140 mmHg and a resting diastolic BP of ≥90 mmHg or receiving BP lowering medication for the treatment of hypertension)
     - Dyslipidemia as defined by a fasting triglyceride ≥200 mg/dL (2.2 mM), fasting low-density lipoprotein (LDL) cholesterol of ≥100 mg/dL (2.5 mM) or a high-density lipoprotein (HDL) cholesterol <40 mg/dL (1.0 mM), or receiving treatment with an approved lipid lowering medication
     - Documented obstructive sleep apnea or receiving treatment for obstructive sleep apnea
     - Cardiovascular disease, including documented coronary artery atherosclerotic disease, peripheral arterial atherosclerotic disease, stroke, or transient ischemic attacks
     - Documented diagnosis of metabolic dysfunction-associated steatohepatitis (MASH) (confirmed by clinical, laboratory, imaging, or biopsy findings)
3. Screening HbA1c <6.5%

4. Reproductive status:

   a. Female participants of nonchildbearing potential, defined as either surgically sterilized (hysterectomy, bilateral salpingectomy, bilateral tubal ligation, or bilateral oophorectomy ≥6 weeks before screening) or postmenopausal (defined as no menses for ≥12 months without an alternative medical cause and a follicle-stimulating hormone level >40 IU/L at the Screening visit for postmenopausal women ≤55 years).

   – Female participants of childbearing potential must have a negative urine pregnancy test (or confirmatory negative serum pregnancy test) during Screening and Day 1. Additionally, they must not be or plan to be breastfeeding, must agree not to donate ova, not attempt to become pregnant and, if engaging in sexual intercourse with a male partner, must agree to use a highly effective method of contraception (refer to Section 10.4, Appendix 4) from the time of signing the informed consent form (ICF) until ≥30 days after the last dose of the study drug.

   b. Male participants must agree not to donate sperm and, if engaging in sexual intercourse with a female partner who could become pregnant, must agree to use a highly effective method of contraception (refer to Section 10.4, Appendix 4) from signing the ICF until ≥90 days after the last dose of study drug.

5.2. Exclusion Criteria

Participants are excluded from the study if any of the following criteria apply at the Screening visit or before dosing at the timepoints in the SoA (Section 1.3):

Diabetes-related Medical Conditions

1. Previous documented diagnosis of diabetes mellitus (except for gestational diabetes and the participant has an HbA1c <6.5%)
2. Use of metformin for any indication is not permitted

Obesity-related Medical Conditions

3. Self-reported change in body weight >5% within 3 months before Screening
4. Body weight ≤80 kg at Screening
5. Have a prior or planned surgical treatment for obesity (excluding liposuction or abdominoplasty, if performed >1 year prior to screening)
6. Have or plan to have endoscopic and/or device-based therapy for obesity or have had device removal within the last 6 months prior to Screening, including, but not limited to, mucosal ablation, gastric artery embolization, intragastric balloon, and duodenal-jejunal endoluminal liner
7. Have obesity induced by other endocrine disorders (such as Cushing’s syndrome, Prader-Willi syndrome, or melanocortin 4 receptor deficiency)

Other Medical Conditions

8. History or presence of significant cardiovascular, pulmonary, hepatic, renal, hematological, GI, endocrine, immunologic, dermatologic, or neurological disease, including any acute illness or major surgery within the past 3 months, or any clinical laboratory abnormality determined by the Investigator or medically certified delegate to be clinically significant
9. Any chronic disorder or severe disease that, in the opinion of the Investigator, might jeopardize the participant's safety, compliance with the protocol, and/or ability to complete the study
10. History of treatment for cancer in the last 5 years (excludes surgically resected skin squamous cell or basal cell carcinoma, in situ cervical cancer, or in situ prostate cancer)
11. Serum calcitonin level at Screening of ≥20 ng/L if estimated glomerular filtration rate (eGFR) ≥60 mL/min/1.73 m^2 or ≥35 ng/L if eGFR <60 mL/min/1.73 m^2, or a personal or family history of medullary thyroid carcinoma or multiple endocrine neoplasia syndrome type 2.
12. Have a thyroid stimulating hormone level outside the range of 0.4 to 6.0 mIU/L at Screening
13. History of chronic pancreatitis or idiopathic acute pancreatitis, or documented history of biliary stones. A participant with a history of acute pancreatitis caused by gallstones may be included in the study if the participant has a cholecystectomy to resolve the problem
14. Chronic malabsorption, regardless of etiology
15. History of Crohn's disease, ulcerative colitis, or other inflammatory bowel disease
16. Impaired renal function at Screening defined as eGFR <30 mL/min/1.73m^2 per Chronic Kidney Disease Epidemiology Collaboration
17. Fasting serum triglyceride level of > 500 mg/dL at Screening
18. Documented diagnosis of heart failure and at Screening New York Heart Association Class III or IV
19. Within the past year before the Screening visit, any of the following: episode of acute coronary event, transient ischemic attack, stroke, peripheral arterial vascular disease leading to revascularization, or other significant cardiovascular event as determined by the Investigator
20. A seated BP after resting for 5 minutes >160 mm Hg systolic or >100 mm Hg diastolic. The measurement will be performed in triplicate and the average of all 3 measurements used to determine eligibility
21. A pulse rate >100 bpm. If elevated, the measurements may be repeated 2 more times and the average of all 3 measurements used to determine eligibility
22. Evidence of abnormality on the screening visit ECG deemed clinically significant by the Investigator, history of known arrhythmia or prolonged QT interval corrected using Fridericia's formula (QTcF) (males >450 ms; females >470 ms), based on the average of

the triplicate ECGs, or risk factors for torsade de pointes (including a family history of long QT syndrome or sudden cardiac death). Have a personal or family history of long QT syndrome, family history of sudden death in a first-degree relative (parents, siblings, or children) before the age of 40 years, or a personal history of unexplained syncope within the last year. Use of prescription or over-the-counter medications known to significantly prolong the QT or QTc interval at Screening.

23. Have an ECG considered by the Investigator with abnormalities that may interfere with the interpretation of changes in ECG intervals at Screening.
24. Ongoing or history of frequent intermittent or chronic tachyarrhythmia syndromes (such as atrial fibrillation, supraventricular tachycardia, and positional orthostatic tachycardia syndrome). Note that participants with a history of premature atrial contractions or premature ventricular contractions may be included.
25. Liver enzyme results elevated >2.0× above the upper limit of normal (ULN) for aspartate transaminase (AST) or alanine transaminase (ALT), or >1.5× ULN for bilirubin (total, conjugated, or unconjugated) (except for cases of known Gilbert's Syndrome). Testing can be repeated twice.
26. Documented diagnosis of cirrhosis
27. Presence or having sequelae of GI, liver, kidney, or other conditions known to interfere with the absorption, distribution, metabolism, or excretion of drugs
28. Any history of ileus
29. Positive test results for an active viral infection: severe acute respiratory syndrome coronavirus 2, human immunodeficiency virus (HIV)-1 or HIV-2 with positive antibodies, hepatitis B virus with a positive hepatitis B surface antigen (HBsAg), or hepatitis C virus (HCV) with positive antibodies and viral load at the Screening visit
30. Have a history of significant active or unstable major depressive disorder or other severe psychiatric disorder (e.g., schizophrenia, bipolar disorder, or other serious mood or anxiety disorder) within the last 2 years
31. Have a lifetime history of suicide attempt or psychiatric hospitalization
32. Have a Patient Health Questionnaire-9 (PHQ-9) score of 15 or more prior to randomization
33. On the Columbia Suicide Severity Rating Scale (C-SSRS) during Screening (within the last 28 days):
    - A "yes" answer to Question 4 (Active Suicidal Ideation with Some Intent to Act, Without Specific Plan) on the "Suicidal Ideation" portion of the C-SSRS

      or
    - A "yes" answer to Question 5 (Active Suicidal Ideation with Specific Plan and Intent) on the "Suicidal Ideation" portion of the C-SSRS

      or

- A "yes" answer to any of the suicide-related behaviors (actual attempt, interrupted attempt, aborted attempt, preparatory act, or behavior) on the "Suicidal Behavior" portion of the C-SSRS

  and

- the ideation or behavior occurred

Prior/Concomitant Therapy

34. Use of medications intended to promote weight loss, within 6 months prior to Screening, including, but not limited to, the following:
    - Saxenda® (liraglutide 3.0 mg) or other GLP-1RA
    - Wegovy® or Ozempic® (semaglutide)
    - Xenical®/Alli® (orlistat)
    - Meridia® (sibutramine)
    - Acutrim® (phenylpropanolamine)
    - Sanorex® (mazindol)
    - Adipex® or Lomaira™ (phentermine)
    - Belviq® (lorcaserin)
    - Qsymia® (phentermine/topiramate combination)
    - Contrave® (naltrexone when combined with bupropion)
    - Zepbound™ or Mounjaro® (tirzepatide)
    - Other similar body weight loss medication, including over-the-counter medications (e.g., Alli)
35. Have current treatment with or history of treatment with (within 3 months prior to Screening) medications that may cause significant weight gain including, but not limited to, tricyclic antidepressants, atypical antipsychotics, and mood stabilizers. However, participants at a stable dose (greater than 6 months and with no expectation that the dose will change within the next year) and who are weight stable for the last 6 months on these medications may be included in the study.

    Examples of medications causing weight gain that must be at stable doses for at least 6 months and with no expectation of changing within the next year include:
    - imipramine
    - amitriptyline
    - mirtazapine
    - paroxetine
    - phenelzine
    - chlorpromazine

- thioridazine
- clozapine
- olanzapine
- valproic acid and its derivatives
- lithium

Note: Selective serotonin reuptake inhibitors other than paroxetine are permitted. Buproprion, when not combined with naltrexone is permitted.

36. Taking daily doses of amphetamine and amphetamine-like stimulants for attention-deficit/hyperactivity disorder, which include Adderall (amphetamine/mixed amphetamine salts), Ritalin (methylphenadate), and Vyvance (lisdexamfetamine)

    Strattera (atomoxetine) is allowed.

37. Use of strong CYP3A4 inducers or moderate to strong CYP3A4 inhibitors, drugs that are sensitive P-gp or BCRP substrates, or drugs that are P-gp inducers or P-gp inhibitors. See Section 10.5 Appendix 5 for details. Concomitant medications that are sensitive CYP3A4 substrates may require monitoring with or without dose reductions, according to the individual respective product labeling, as summarized in Section 10.5 Appendix 5. Concomitant use of lovastatin and simvastatin is not allowed (however use of other statins is not prohibited)
38. History of substance abuse within 12 weeks before Screening
39. Participants recovering from opiate abuse or taking methadone, buprenorphine/naloxone (Suboxone®), or pentazocine (Talwin™). However, participants prescribed a medically appropriate use for opiates are allowed
40. Positive drugs of abuse screen (excluding marijuana [tetrahydrocannabinol (THC)]) before the first dose of study drug unless the participant is prescribed one of these classes of drugs (narcotics) for a medically appropriate indication.

Prior/Concurrent Clinical Study Experience

41. Are currently enrolled in any other clinical study or have participated in another clinical trial of an investigational drug or device within 60 days before the first study drug administration. At least 6 months before the first study drug administration if the clinical trial included a GLP-1RA, GIP receptor agonist (RA), glucagon RA, amylin RA, peptide YY RA, neuropeptide Y RA, or any combination thereof and/or any investigational product currently being used for participants living with overweight, obesity, or type 2 diabetes.

Other Exclusions

42. Pregnant, breastfeeding, or intends to become pregnant
43. Female of childbearing potential and not using an adequate contraceptive method (adequate contraceptive measures as required by local regulation or practice) for the duration of the trial and for ≥30 days following the last dose of study drug

44. Male not using adequate contraceptive method for the duration of the trial and for ≥90 days following the last dose of study drug
45. Consumption of grapefruit, tangelo, or Seville orange (or products containing grapefruit, tangelo, or Seville orange) within 10 days before the first administration of study drug and throughout the study
46. Demonstrated clinically significant allergic reactions (e.g., food, drug, or atopic reactions, asthmatic episodes) that required intervention, (e.g., emergency room visit, epinephrine administration) and which, in the opinion of the Investigator, would interfere with the participant’s ability to participate in the trial
47. Known hypersensitivity to any of the study drug ingredients

5.3. Lifestyle Considerations

5.3.1. Nutrition and Good Health Instructions

Nausea is a common side effect of GLP-1 RAs, impacting up to 50% of patients. The gastrointestinal issue arises from the medication's effects on slowing down gastric emptying, which can trigger feelings of nausea as the gastro-intestinal tract adjusts to the change in gastric motility and nutrient absorption. As a class of drugs, nausea is often most pronounced when starting GLP-1 RAs or when increasing the dosage; therefore, it is imperative to monitor and adjust dietary plans when participants initiate GLP-1 RA therapy.

Participants will be instructed by a nutritionist/dietician or trained site staff on how to manage nutritional needs, while encouraging diets less likely to worsen nausea and how to increase their physical activity. In general, the nutritional guidance will encourage a low fat, high protein plan that will help reduce loss of muscle mass while decreasing the intake of high calorie foods with low nutritional benefit. High-fat meals can trigger nausea for several reasons, including slowing down stomach/gastric emptying, potentially triggering acid reflux, and affecting gallbladder function required for normal fat-digestion, whereas a low-fat liquid meal have been shown to reduces symptoms of gastroparesis (Homoko, 2015). Importantly, dyspepsia (postprandial distress syndrome [PDS]) symptoms of abdominal discomfort, fullness, early satiety, and nausea occur after ingestion of high calorie meals and are very common affecting ~30% of the population [Talley et al., 1992]. People with dyspepsia/PDS do not respond to proton pump inhibitor therapy and meals with high-fat content delay gastric emptying and prolong the sensation of stomach fullness and induce nausea (Koch, 1996). Therefore, the monitoring of macronutrient composition and calorie load is critical when using GLP1R-agonist as high fat meals can worsen the known tolerability issues with the GLP1R-class.

Exercise habits will also be recommended to increase energy utilization and maintain muscle mass. Detailed guidance for nutrition and exercise guidance will be further detailed in the Site Procedural Manual. Briefly, at all visits, participants will be encouraged to increase their physical activity to at least 150 minutes per week.

5.3.2. Meals and Nutritional Restrictions

Consumption of grapefruit, tangelo, or Seville orange (or products containing grapefruit, tangelo, or Seville orange) is not permitted within 10 days before the first administration of study drug and throughout the study.

At site visits, participants will be instructed to take their study drug dose with a light, low-fat breakfast or a low carbohydrate/fat, high protein meal replacement shake that will be provided by the sponsor. A similar approach to self-administration of drug is recommended throughout the study.

The Sponsor will provide the option of GLP-1 meal replacement service and dietary counseling (dietician/nutritionist, or equivalent qualified delegate) that will focus on advice on healthy food choices with emphasis on calorie restriction using a hypocaloric diet. As a guide to study participants the macronutrient composition of the diet may encompass:

- maximum ~<30% of energy from fat
- approximately ~20-30% of energy from protein
- approximately ~40-50% of energy from carbohydrates
- an energy deficit of approximately 500 kcal/day compared to the participant's estimated total energy expenditure (TEE) is recommended

To promote adherence, it is encouraged that a 3-day diet and exercise diary be completed prior to each counseling visit. During each visit, the participant's diet is reviewed and advice to maximize adherence is provided if needed. The hypocaloric diet should be continued after randomization and throughout the treatment period. In addition to dietary counseling, the participant will also have the option of receiving guidance from a certified exercise physiologist (or equivalent qualified delegate) that will focus on advice on structured or guided exercise programs.

If a BMI ≤22 kg/m^2 is reached, the recommended energy intake should be recalculated with no kcal deficit for the remainder of the study.

Total energy expenditure (TEE) is calculated by multiplying the estimated Basal Metabolic Rate (BMR) (see table below) with a Physical Activity Level value of 1.3 (FAO/WHO/UNU 2004), which reflects an inactive lifestyle. This calculation provides a conservative estimate of caloric requirements: TEE (kcal/day) = BMR × 1.3

Equations for estimating BMR in kcal/day

Sex	Age (years)	BMR kcal/day
Men	18 to 30	15.057 × actual weight in kg + 692.2
	31 to 60	11.472 × actual weight in kg + 873.1
	>60	11.711 × actual weight in kg + 587.7
Woman	18 to 30	14.818 × actual weight in kg + 486.6
	31 to 60	8.126 × actual weight in kg + 845.6
	>60	9.082 × actual weight in kg + 658.5

Abbreviations: BMR = basal metabolic rate; WHO = World Health Organization.
*Revised WHO equations (adapted from: FAO/WHO/UNU 2004).

5.3.3. Caffeine and Tobacco

Caffeine-containing products (e.g., coffee, tea, chocolate, and caffeine-containing sodas, colas, etc.) may not be consumed within 2 hours before measuring vital signs and ECGs. Participants will be allowed to maintain their regular caffeine consumption throughout the study period (excluding the 2 hours before measuring vital signs and ECGs).

Participants must consume no more than 10 cigarettes or equivalent per day.

Participants must have a negative result in a urine drugs of abuse test (excluding marijuana) at Screening and must abstain from recreational drugs throughout the study. Participants must not be in a drug recovery program that prescribes methadone, buprenorphine/naloxone (Suboxone®), or pentazocine (Talwin™).

5.4. Screen Failures

A screen failure occurs when a participant who has consented to participate in the clinical study is not subsequently assigned to study intervention/entered into the study. A minimal set of screen failure information is required to ensure transparent reporting of screen failure participants to meet the CONSORT publishing requirements and to respond to queries from regulatory authorities. Minimal information includes demography, screen failure details, eligibility criteria, and any SAE.

6. STUDY INTERVENTION(S) AND CONCOMITANT THERAPY

6.1. Study Intervention(s) Administered

Study interventions are all prespecified, investigational and non-investigational medicinal products intended to be administered to the study participants during the study conduct. Delegated study site personnel will supervise and instruct participants on the administration of the study intervention (i.e., investigational product [IP]). Participants will self-administer the aleniglipron or placebo tablets (Table 5) orally in the morning with noncarbonated room temperature water (approximately 240 mL), followed by a light breakfast (details of nutritional recommendations will be provided in the Site Procedural Manual). Orally administered study drugs must be swallowed whole and not chewed, divided, dissolved, or crushed. The exact dates and times of study drug administration (when administered in the clinic) will be recorded in the electronic Case Report Form (eCRF). The exact date and time of study drug administration at home will be recorded in the e-diary.

Table 5: Study Intervention(s) Administered

Intervention Name	Aleniglipron	Placebo
Type	Drug	Control
Dose formulation	Tablet	Tablet
Unit dose strength(s)	2.5, 5, 30, and 60 mg	Not applicable
Route of administration	Oral	Oral
Sourcing	Aleniglipron tablets supplied by designated manufacturer/ distributor of Sponsor	Placebo tablets supplied by designated manufacturer/ distributor of Sponsor
Packaging and labeling	Study intervention will be provided in 75 mL oral solid pharmaceutical HDPE bottle containing 1 g desiccant enclosed with a 33 mm PP CRC (fitted with an aluminum foil induction seal). Each bottle will be labeled to conform with country requirements.	

Abbreviations: CRC=child-resistant caps; HDPE=high-density polyethylene; PP=polypropylene.

Participants will receive QD doses of study drug for 252 consecutive days.

6.2. Dose Modification/Titration Adjustments

6.2.1. Dose Reduction

In the event that tolerability cannot be mitigated by any other means of nutritional adjustment, holistic or prescribed anti-emetic treatments, titration adjustments will be permitted upon documented discussion with the Sponsor, as a last resort to manage AEs at the current dose. Every effort should be made to avoid down-titration. Adjustments will include down titrating to a lower dose, and then titrating back up to the target dose or as needed. Education and coaching on tolerance strategies will be provided to enable as many participants as possible to attain their target dose. Recommendations will be detailed in the Site Procedural Manual.

In the event that a participant experiences AEs that preclude continuing at a prescribed dose, the participant may take a lower tolerated dose and should be rechallenged to take the next highest full dose in the morning after 1 or 2 days of introducing further nutritional counseling or anti-emetic/symptomatic therapy. Both the Investigator and SMR must be consulted/informed and communication documented prior to the down-titration and providing the up-titration plan. The participant should receive reinforcement of counseling on steps to improve tolerability. This should be delivered at every visit by the qualified study staff.

Following down-titration, participants should be up-titrated to the next dose level, with the goal of having participants achieve their target dose level, even if delayed, per Investigator discretion.

6.2.2. Dose Interruption/Hold

If the medication needs to be temporarily stopped, it is not desirable to hold dosing beyond 1 or 2 days in order to avoid losing tolerability gained from maintaining daily dosing. Both the Investigator and SMR must be consulted/informed and communication documented providing the up-titration plan.

If unacceptable tolerability symptoms re-occur or persist, the participant should continue in the study at the highest tolerable dose unless the Investigator and the SMR agree on an alternative appropriate approach. All participants must be able to tolerate a minimum dose of 30 mg/day (down titration to 15 mg/day per Investigator guidance only) to remain in the study, otherwise early termination will be initiated.

If the participant has been on a dose hold for >3 days, a new titration schema should be devised upon consultation with the SMR.

Date and reasons for study drug dose interruptions, reductions, and discontinuations will be captured in the Case Report Form.

6.2.3. Management of Participants with Gastrointestinal Symptoms

In the clinical studies conducted with aleniglipron to date, the most commonly reported TEAEs for participants receiving aleniglipron were GI-related events, including nausea, vomiting, constipation, and diarrhea. To mitigate GI symptoms and manage participants with GI AEs, the Investigator should do the following:

- Advise participants to eat smaller meals, e.g., having 4 or more smaller meals throughout the day instead of 3 large daily meals, and to stop eating when they are full. Participants will be able to consult with site or study dietician (centralized) to be advised on type of macronutrient composition of diet and caloric target. The centralized dietician will be available to all study participants.
- Advise participants that lower-fat meals (or a liquid meal, especially 24 hours prior to increasing dose) and a bland, low-calorie meal plan might be better tolerated.
- Adopt a GLP-1 RA meal service provided by the Sponsor coupled to dietary guidance.
- Advise participants to eat slowly to allow time to sense fullness and to stay hydrated.

- Prescribe anti-emetic or antidiarrheal medication according to the Investigator judgment. This should be captured as concomitant medication in the eCRF.
- Advise participants that over time, their body can adapt to the study intervention, and the symptoms may abate.
- Other measures as appropriate which, in the opinion of the Investigator, may help with tolerability.
- A reduction in study drug for unrelenting tolerability issues should rarely be warranted and the process to be followed is described in Section 6.2 and will be outlined in the Site Procedural Manual.

6.3. Identification and Description of the Investigational Product

Aleniglipron oral tablets at the dose strength of 2.5, 5, 30, and 60 mg will be used as the IP in this study. Tablets of matching composition and appearance to the IP will be administered as placebo.

6.4. Supply and Labeling of Investigational Product

Aleniglipron tablets and placebo tablets will be supplied by a designated manufacturer/distributor of the Sponsor to the clinical sites. All IP material will be packaged in 75 mL oral solid pharmaceutical high-density polyethylene bottle containing 1 g desiccant enclosed with a 33 mm polypropylene child-resistant caps (fitted with an aluminum foil induction seal) with an appropriate label.

Based on available stability data, the aleniglipron tablets and placebo tablets are to be stored at controlled room temperature, 15°C to 25°C (59°F to 77°F).

Additional information on aleniglipron is provided in the Investigator's Brochure.

Aleniglipron tablet and placebo tablet doses to be administered to a participant will be labeled according to current Good Manufacturing Practices for medicinal products. The detail on the labels will include, but not be limited to, the following:

- The notation- 'Caution: New Drug—Limited by Federal (or US) Law to Investigational Use.'
- Protocol number
- Name of Sponsor
- Route of administration
- Storage conditions

The final labels will be coded as appropriate to the study design and in compliance with this protocol.

Standard operating procedures will be followed for the receipt, handling, and accountability of the study drugs (aleniglipron tablets and placebo tablets). Study drug supplies will be stored securely under the appropriate conditions at the site pharmacy.

Detailed instructions for the labeling and supply of aleniglipron tablets and placebo tablets are described in the study Pharmacy Manual.

6.5. Preparation, Handling, Storage, and Accountability

The study drugs (aleniglipron tablets and placebo tablets) must be stored in a secure area with access limited to the Investigator and authorized staff at the specified storage temperature. Refer to the directions for use in the study Pharmacy Manual for additional storage requirements.

The Investigator (or delegate) must ensure that deliveries of study drug from the Sponsor (or designated supplier) are correctly received by a responsible person, that all receipts of shipments are recorded on the appropriate drug accountability forms prepared by the site pharmacy, and that the study drugs are stored in a secure area under the recommended storage conditions. It is also the responsibility of the Investigator (or delegate) to ensure that the integrity of packaging not be jeopardized before dispensing.

Only participants enrolled in the study may receive the study drugs in accordance with all applicable regulatory requirements. Only authorized and trained site staff may dispense and administer the study drugs.

Upon receiving the study drug from a trained staff member and being instructed how to take their daily dose, the participant will then take the first dose as instructed and record the dose in the appropriate accountability records. The site staff member will be responsible for dispensing study drug in accordance with the randomization schedule. The dispensing procedures are described in the Pharmacy Manual.

The site pharmacy or a study coordinator will dispense the study drug, and the clinical site staff will give the study drug only to participants included in this study following the procedures stated in this study protocol. Dispensation of study drugs will be recorded in the appropriate drug accountability records and the eCRF. Dispensation of study drug will be verified by a second staff member. Study participants will be instructed on dosing at each dispensation visit.

6.6. Assignment to Study Intervention

All study participants who sign an ICF at Screening will receive a unique sequential number (i.e., a Screening Number).

Participants who meet the study eligibility criteria will be assigned a randomization number on Day 1 that corresponds to a study treatment. The allocation to aleniglipron tablet or placebo tablet will be performed using a randomization algorithm and in accordance with the randomization schedule. To obtain balance across treatment groups, the randomization will be stratified by baseline BMI and sex. The stratification factors and strata within each factor are listed below:

1. Baseline BMI
    a. $\leq$35 kg/m^2
    b. >35 kg/m^2
2. Sex
    a. Male

b. Female

No more than 10% of the participants with BMI <30 kg/m^2 and no more than 65% female participants will be randomized.

All eligible participants will be centrally assigned to a randomized study drug after all eligibility criteria have been verified and confirmed by the Investigator or designee.

6.7. Blinding and Breaking Code

This is a double-blind, placebo-controlled study. Blinding of study drug/placebo assignment is critical to the integrity of this clinical trial. Those blinded to study drug assignment include individuals from the Sponsor (and delegates) involved in day-to-day, study-related activities, the Investigator, clinical study personnel participating in participants' care or clinical evaluations, and the study participants. Blinded personnel must not make any effort to determine which study drug therapy is being administered. Some individuals from the Sponsor who are not involved in the daily operations of the study may be unblinded in order to evaluate clinical, tolerability data for discussions of PK parameters, and SAEs that may need to be reported expeditiously.

Blind-breaking instructions, and the unblinding process will be provided to investigative sites. It is expected that in the majority of cases, AEs can be properly managed without the need for unblinding. However, in the event of a medical emergency in which knowledge of an individual participant's intervention assignment is considered critical to the participant's well-being and management, the Investigator may unblind the intervention assignment for the participant in question. Unless the clinical situation warrants emergency unblinding, the Investigator is strongly advised to discuss options with the SMR or contract research organization (CRO) Medical Monitor (MM) prior to any unblinding. The Investigator should contact the SMR or CRO MM to discuss the participant and circumstances requiring the unblinding. The blind will be broken only for the specific participant under consideration. If the blind is broken for any reason and the Investigator was unable to contact the Sponsor or CRO MM before unblinding, the Investigator must notify the Sponsor and CRO MM as soon as possible, without revealing the participant's intervention assignment (unless important to the safety of participants remaining in the study). The Sponsor (and any Sponsor nominee, e.g., blinded Study Monitor[s]) will remain blinded to the participant's intervention assignment. Every effort will be made to restrict knowledge of the participant assignment to aleniglipron or placebo to the Investigator only. The Study Monitor must be informed promptly that the Investigator has been unblinded to a participant's intervention assignment. The participant may remain in the study under a single blind. The date and reason that the blind was broken must be recorded in the source documentation.

6.8. Study Intervention Compliance

All study drugs are for use only in this clinical study and should not be used for any other purpose.

The Investigator is responsible for study drug accountability, reconciliation, and record maintenance at the clinical site. In accordance with all applicable regulatory requirements, the Investigator or designated trained site staff must maintain study drug accountability records throughout the course of the study. This person will document the quantity of aleniglipron tablets

or placebo tablets received by the site pharmacy and the amount dispensed to, administered to, and returned by participants, as applicable. The shipment/receipt records as well as records of dispensing, administration, and return will be verified and documented by a second member of the site staff. The Investigator (or designated trained site staff) is responsible for assuring the retrieval of all remaining study drug supplies following administration to participants.

The study drug inventory and accountability/dispensing records must be available for inspection by the Study Monitor during the study. Used containers of the study drug will be retained and sequestered per participant and made available to the Study Monitor during reconciliation of study medication. Please see the Pharmacy Manual for further details.

When requested in writing by the Sponsor and following drug accountability and reconciliation, unused study drug supplies may be returned to the Sponsor or destroyed by the Investigator (or delegate) according to investigational site standard operating procedures, provided destruction does not expose humans to risks from the study drugs. Records shall be maintained of the return or destruction of the study drug materials. These records must show the identification and quantity of each unit returned or disposed of the method of destruction (taking into account the requirements of local law), and the person who returned or disposed of the study drug materials. Such records must be submitted to the Sponsor.

Pharmacokinetic Monitoring to Verify Compliance with Dosing Regimen:

To verify compliance with the study dosing regimen and adherence to the protocol requirements, random PK blood draws may be conducted on enrolled participants to monitor the concentration of the study drug in the bloodstream. Based on model projections and existing data, further adherence assessments and follow-up may be conducted.

6.9. Continued Access to Study Intervention After the End of the Study

Participants completing the 36 weeks of treatment during the double-blind period will be offered the opportunity to participate in an OLE to receive up to 180 mg aleniglipron once daily.

6.10. Treatment of Overdose

The maximum tolerated dose for aleniglipron has not yet been established. For this study, any dose of study intervention greater than the planned highest dose in this study within a 24-hour time period will be considered an overdose.

In the event of an overdose, the Investigator/treating physician should do the following:

- Evaluate the participant to determine, in consultation with the MM, if possible, whether study intervention should be interrupted or whether the dose should be reduced.
- Closely monitor the participant for any AE/SAE and laboratory abnormalities, as medically appropriate.
- Document the quantity of the excess dose as well as the duration of the overdose on eCRF.

- There is no specific antidote for overdose with aleniglipron. In the event of a suspected overdose, it is recommended that appropriate supportive clinical care should be instituted, as dictated by the participant's clinical status.

6.11. Prior and Concomitant Therapy

Prior medication use as of the Screening visit will be recorded at Screening and Day 1. Concomitant medication (all medication/treatment/therapy taken between the date of first study drug administration and the end of the follow-up period inclusive) use will be recorded at each clinic visit (Section 1.3).

Use of any prohibited prescription or over-the-counter medications (including herbal products, diet aids, and hormone supplements, with the exception of contraception) must be discontinued within 10 days or 5-times apparent terminal elimination half-life values of the medication (whichever is longer) or 6 months for GLP-1RAs before the first study drug administration and throughout the study, with the exception of medications used to manage AEs at the discretion of the Investigator or medically certified delegate, including, but not limited to, the examples described below.

- For common ailments, occasional use of acetaminophen (doses of 500 mg up to every 6 hours or 2 g per day maximum for ≤3 consecutive days), topical hydrocortisone for skin irritation, and contraceptives for women of childbearing potential (WOCBP).
- Treatment of GI-related symptoms or symptoms due to respiratory viral infections may be allowed (including, but not limited to, antiemetics, analgesics, therapies to treat cough, etc.). The Investigator should consult with the Sponsor on a case-by-case basis.
- While participating in the study, participants will not be allowed the concomitant use of strong CYP3A4 inducers or moderate to strong CYP3A4 inhibitors or P-gp inducers or inhibitors due to their potential to increase/decrease aleniglipron exposure (Section 10.5). If an acute treatment with a moderate CYP3A4 inhibitor is required post-study start and there is no alternative treatment, the Investigator should contact the Sponsor for assessment based on the aleniglipron doses being administered (co-administration at low doses could be feasible). Chronic concomitant treatment with moderate CYP3A4 inducers or inhibitors is not allowed. Co-administration of aleniglipron with sensitive P-gp or BCRP substrates is also not allowed.

7. DISCONTINUATION OF STUDY INTERVENTION AND PARTICIPANT DISCONTINUATION/WITHDRAWAL

Discontinuation of specific sites or of the study as a whole are detailed in Section 10.1.10.3, Appendix 1.

7.1. Discontinuation of Study Intervention (Investigational Product)

In rare instances, it may be necessary for a participant to permanently discontinue study intervention at the discretion of the Investigator or participant following documented discussion with the SMR. If study intervention is permanently discontinued, the participant will be allowed to remain in the study to be evaluated for all planned efficacy and safety measures as shown in the SoA (except for PK, ECG, daily diary, and Hunger and Satiety assessments). If the participant does not wish to attend the scheduled clinic visits, efforts should be made to have the visits converted to phone contacts; however, all efforts should be made to have the participant attend the ETV as soon as possible after study intervention is discontinued. If the ETV cannot be done in person, it can be done as a phone contact (to collect safety-follow up, AEs, and concomitant medications). If the participant refuses to attend the ETV, information about the attempts to follow up with the participant must be documented in the participant's medical record. See the SoA (Section 1.3) for details on information to be collected at the ETV.

Participants who discontinue early from the study, whether due to voluntary withdrawal, Investigator decision, or adverse event, or who complete the study, may be asked to voluntarily participate in a structured qualitative exit interview to capture the participant's experience while on study. All interested participants will provide written informed consent before taking part in the exit interview.

Possible reasons leading to permanent discontinuation of study drug include the following:

- The participant requested to discontinue the study drug.
- The participant has discontinued the study drug on their own and refuses to restart the study drug.
- Any nonstudy medication for weight loss is given for more than 1 week.
- Any TEAE, SAE, or clinically significant laboratory value assessed as possibly or probably related to study drug that the Investigator deems to be medically relevant and may potentially endanger the welfare of the participant.
- Pregnancy.
- Significant noncompliance with the protocol.
- Inability to tolerate at least 30 mg (15 mg per Investigator guidance only)QD dosing.
- The Investigator decides that the participant should be discontinued from the study drug.
- If enrolled in any other clinical study involving an investigational agent

- Initiation of other weight management medications (see Exclusion criteria) or if the participant has bariatric surgery or body weight reduction procedures.
-

7.1.1. Liver Chemistry Stopping Criteria

The study drug should be interrupted or discontinued if 1 or more of the following conditions occur:

- ALT or AST >8× ULN
- ALT or AST >5× ULN for more than 2 weeks
- ALT or AST >3× ULN and serum total bilirubin (TBL) >2× ULN or international normalized ratio (INR) >1.5 × ULN without elevated serum alkaline phosphatase (ALP), and no other disease or condition can be found to explain the abnormalities
- ALT or AST >3× ULN with the appearance of fatigue, nausea, vomiting, right upper quadrant pain or tenderness, fever, rash, and/or eosinophilia (>5%)

See Section 10.6, Appendix 6 for details on suggested actions and follow-up regarding liver safety.

7.1.2. QTc Stopping Criteria

If the mean QTcF of an ECG is >500 ms or there is a >60 ms change from the Baseline predose ECG triplicate, then further dosing with study drug should be withheld until the participant can be assessed. Further assessment will include repeat triplicate ECGs, physical examination, and assessment of symptoms, after which the Investigator or qualified designee in consultation with the SMR will determine if the participant can continue in the study.

7.2. Participant Discontinuation/Withdrawal From the Study

Participants will be advised that they are free to withdraw from the study at any time for any reason or, if necessary, the Investigator (or medically certified delegate) may discontinue a participant from the study to protect the participant's health, following documented discussion with the SMR.

A participant may voluntarily withdraw or be withdrawn from the study for reasons, including, but not limited to, the following:

- The need to take medication chronically that may interfere with study measurements
- Intolerable/unacceptable AEs
- Noncompliance of the participant with the protocol
- Discontinuing study drug and unwilling to be rechallenged
- Withdrawal of consent
- If, in the Investigator's judgment, it is in the participant's best interest.

The Sponsor will be notified as soon as possible of any participant withdrawals. The date and reasons for withdrawal will be recorded in the eCRF and included in the final clinical study report (CSR), along with any AEs and necessary medical treatment.

Any participant who withdraws consent from the study should have the ETV assessments performed as soon as possible, which will be the EOS.In the event that a participant is discontinued from the study due to an SAE, the Investigator or medically certified delegate will evaluate the urgency of the event. If the situation warrants, the Investigatorwill take appropriate diagnostic and therapeutic measures and attempt to notify the SMR. If the situation is not an immediate emergency, the Investigator, or medically certified delegate, at the clinical study site will attempt to contact the SMR for consultation.

7.3. Lost to Follow-up

A participant will be considered lost to follow-up if the participant repeatedly fails to return for scheduled visits and is unable to be contacted by the study site.

The following actions must be taken if a participant fails to return to the clinic for a required study visit:

- The site must attempt to contact the participant and reschedule the missed visit as soon as possible, counsel the participant on the importance of maintaining the assigned visit schedule and ascertain whether the participant wishes to and/or should continue in the study.
- Before a participant is deemed lost to follow-up, the Investigator or medically certified delegate must make every effort to regain contact with the participant (when possible, telephone calls, emails, and if necessary, a certified letter to the participant's last known mailing address or local equivalent methods). These contact attempts should be documented in the participant's medical record.
- Should the participant continue to be unreachable, the participant will be considered to have withdrawn from the study and lost to follow-up.
- Site personnel, or an independent third party, will attempt to collect the vital status of the participant within legal and ethical boundaries for all participants randomized, including those who did not get study intervention. Public sources may be searched for vital status information. If vital status is determined as deceased, this will be documented, and the participant will not be considered lost to follow-up. Sponsor personnel will not be involved in any attempts to collect vital status information.

8. STUDY ASSESSMENTS AND PROCEDURES

All study assessments to be performed and the timing of each assessment are presented in the SoAs (Section 1.3). Clinical staff are required to perform assessments at the prespecified timepoints within the time windows indicated in this protocol. Actual times of procedures for each participant may vary depending on scheduling and will be recorded in the eCRF. For the first set of evaluations on each study visit where applicable (not necessary for 2- to 4-hour return), participants will be instructed to arrive having fasted for at least 8 hours and without having consumed caffeine within 2 hours of arrival. In the event of multiple procedures scheduled at the same time, the order, where possible, will be as described in Figure 3.

Figure 3: Order of Procedures

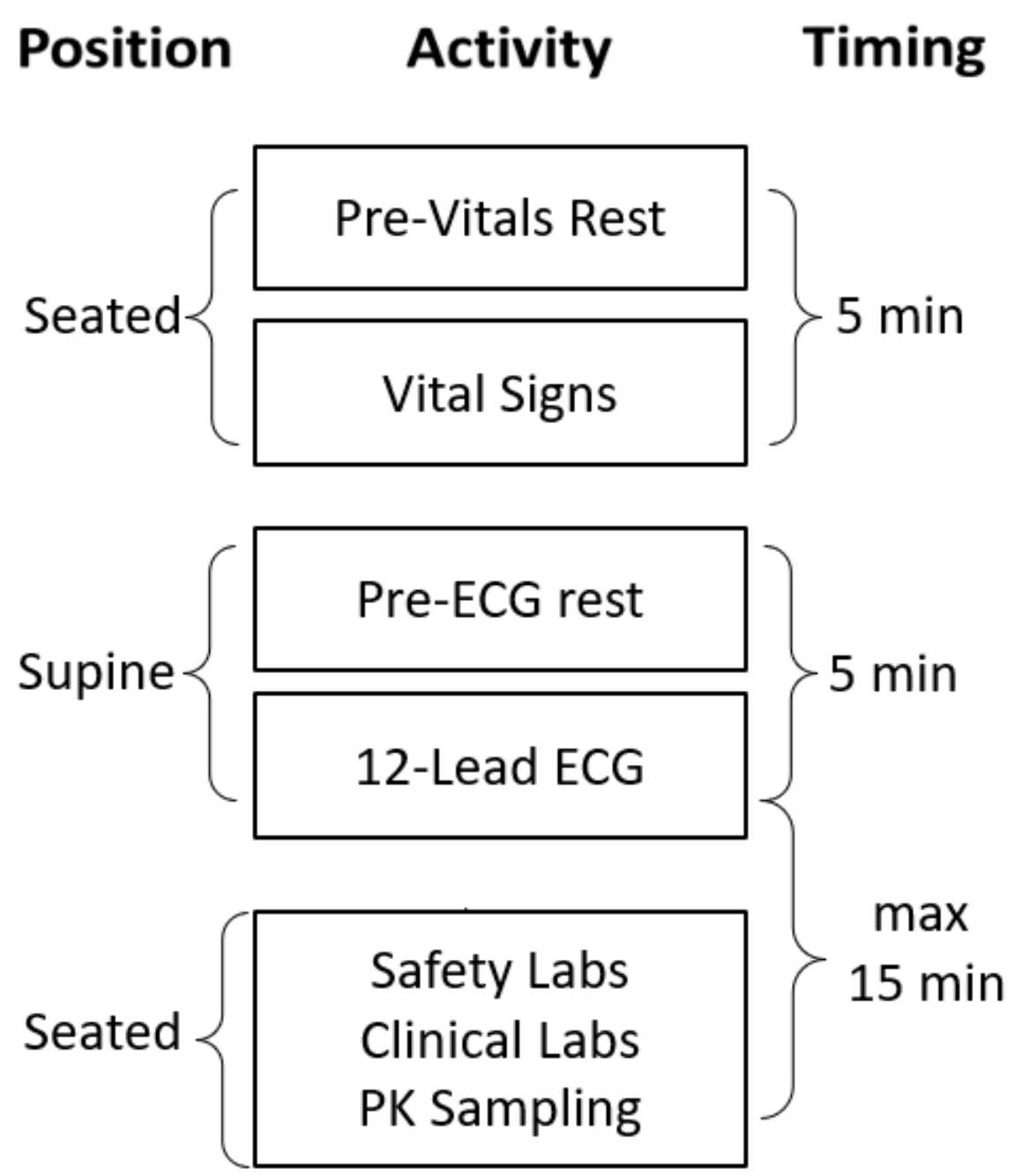

Abbreviations: ECG=electrocardiogram; PK=pharmacokinetic.

8.1. Baseline Procedures

Before performing any study-related procedures, the Investigator (or delegate) will obtain written informed consent from participants.

Clinical staff is required to perform all assessments at the prespecified timepoints within the time windows indicated in the SoAs (Section 1.3). Baseline for body weight, vital signs, blood and urine clinical laboratory tests, and ECG parameter data will be defined as the last assessment done before the first administration of study drug for each participant.

Demographic data, including gender, age, race, and ethnicity, will be recorded at Screening.

Body height (centimeters; at Screening only) using a stadiometer and weight (kilograms) using certified calibrated weight scales in hospital gowns without shoes will be measured at baseline and postbaseline visits as detailed below.

Measuring Height

- Step 1: Ask the participant to remove their footwear and any headgear (light headgear worn for religious reasons can remain, but this should be worn by the participant at every clinic visit when height is measured).
- Step 2: Ask the participant to stand on the height measuring board (stadiometer) or against a wall with their feet together and their knees straight with their heels against the backboard, the stadiometer, or the wall.
- Step 3: Ask the participant to look straight ahead without tilting their head up.
- Step 4: Ask the participant to breathe in and stand tall. Measure and record the participant’s height in centimeters to 1 decimal place.

Measuring Weight (to be measured in triplicate)

- Body weight measurements should be done in a consistent manner using a certified calibrated electronic scale capable of measuring weight in kilograms to 1 decimal place.
- All weights for a given participant should be measured using the same scale, whenever possible, at approximately the same time in the morning after evacuation of bladder contents.
- Body weight must be measured in fasting state (minimum 8 hours). If the participant is not fasting, the participant should be called in for a new visit within the visit window to have the fasting body weight measured.
- Step 1: Ask the participant to remove all outer clothing and headgear (light headgear worn for religious reasons can remain, but this should be worn by the participant at every clinic visit when weight is measured). Participant should be wearing a hospital gown (undergarments are ok to wear).
- Step 2: Make sure the scale is placed on a firm, flat, even surface (not on carpet, on a sloping surface, or a rough, uneven surface).
- Step 3: Ask the participant to step onto the scale with 1 foot on each side of the scale.
- Step 4: Ask the participant to stand still with arms by sides and then record weight in kilograms to the nearest one-tenth kilogram.

Waist circumference (centimeters) will also be measured at the timepoints as indicated in the SoAs (Section 1.3). Proper measurement of waist circumference requires identifying the iliac crests and placing a measuring tape along the top of the iliac crests, parallel to the ground and around the central abdomen. Three measurements should be collected, placing the tape measure each time.

Blood pressure measurements should be performed in triplicate after the participant has been seated for at least 5 minutes. A proper sized cuff should be placed around the left (or right only if left is not available) upper arm. The same arm should be used for each measurement (with preference for the left arm). Three measurements should be taken, at least 2 minutes apart and recorded.

Medical history, including pre-existing conditions, allergies, prior significant illnesses, and surgical history, will be recorded. Medical history will also include smoking history.

Any nonstudy concomitant medication or therapy that is taken by or administered to a participant during the study will be recorded. The medication/therapy name as well as dose, route of administration, indication, and dates of use will be noted, as applicable.

8.2. Participant-reported Outcomes

8.2.1. Electronic Diary

An electronic diary (e-diary) will be used for the purpose of recording and transmitting data on participant-reported outcomes. Symptoms will be reported one week after each dose titration step and information regarding medication taking (including date and time of dosing) will be recorded daily. Upon completion of the e-diary, data will be available for periodic review by the sites, CRO, and Sponsor through the web-based electronic data and control system and summary reports.

A study-specific and secure website will be used by study staff to access and review electronic participant diary data and data summaries at each visit but could be more frequent at Investigator discretion, if needed. The permissions for enabling or preventing such review are set so that only authorized site personnel and e-diary vendor staff can see individually identifying health information, as defined in regulations concerning the protection of privacy for trial participants as well as the individuals who serve as study staff. All users of the website will have undergone identity certification.

Detailed directions for the administration of the e-diary are provided in the electronic patient reported outcome ePRO Manual. Symptoms in the diary should be reviewed by the Investigator for clinical significance and possible AE reporting, but not every symptom in the diary needs to be recorded as an AE. More severe symptoms have greater likelihood of being clinically significant and therefore reported as an AE, but less severe symptoms should still be reviewed for AE reporting at the discretion of the Investigator.

8.2.2. Impact of Weight on Quality of Life-Lite CT, Short Form-36 Acute Version, and Hunger and Satiety Questionnaires

Participants will complete an Impact of Weight on Quality of Life-Lite Clinical Trials Version (IWQOL-Lite CT) and Short Form 36 health survey (SF-36) acute version health survey questionnaires assessing weight-related physical and psychosocial function as indicated in the SoA (Section 1.3). Participants will also complete a hunger and satiety evaluation as indicated in the SoA (Section 1.3). For additional information on the IWQOL-Lite CT assessment, please refer to the following reference (Kolotkin, et al., 2019). For more information on the SF-36 assessment, please refer to the following reference: (Maruish, 2011).

8.3. Safety Assessments

Safety assessments will include clinical assessments, such as medical history, concomitant medication use, evaluation of AEs and TEAEs, weight, physical examination, vital signs (systolic and diastolic BP, HR, and temperature), 12-lead ECGs in triplicate, mental health

questionnaires (C-SSRS and PHQ-9), pregnancy testing if indicated, and laboratory test results. Planned timepoints for all safety assessments are provided in the SoAs (Section 1.3).

8.3.1. Physical Examinations

Vital signs and anthropometrics (including weight) will be collected at each visit.

Full physical examinations will include, at a minimum, assessment of the following: general appearance, head, ears, eyes, nose and throat, neck (including thyroid and lymph nodes), respiratory system, cardiovascular system, GI system, neurological condition, musculoskeletal system, dermatological system, and any other focused assessments suggested by the presence of specific symptoms.

Symptom-directed physical examinations will be performed if deemed required by a medically certified delegate member and will include focused assessments suggested by the presence of specific symptoms.

All physical examinations will be conducted by a licensed Investigator or medically certified delegate or licensed nurse practitioner within the time windows specified in the SoAs (Section 1.3). Physical examinations may be performed at various unscheduled timepoints if deemed necessary by the Investigator or medically certified delegate. All clinically significant findings post dose will be recorded as AEs.

8.3.2. Vital Signs

Vital signs assessments will include systolic and diastolic BP, HR, and temperature. Participants should be resting in a seated position for ≥ 5 minutes before and during vital signs measurements. Predose vital signs assessments should be performed within 1 hour before dosing.

Abnormal vital signs assessments that are NOT clinically significant may be repeated at the Investigator's discretion. Abnormal vital signs assessments that ARE clinically significant will be repeated. All clinically significant findings will be recorded as AEs.

8.3.3. Electrocardiograms

Twelve-lead ECGs in triplicate (including, but not limited to, the measurements of ventricular HR, PR interval, QRS duration, QT interval, and QTcF) will be performed at the timepoints and windows indicated in the SoAs (Section 1.3). Note that during PK sample collections, ECGs will need to be collected in triplicate with each indicated PK sample and should be within 15 minutes before the blood draw. At each protocol scheduled timepoint, ECGs will be performed for safety evaluation. Each ECG will be conducted after the participant has been supine for ≥5 minutes in a quiet setting without distractions (e.g., television, cell phones). ECGs will be interpreted, signed, and dated by the Investigator or medically certified delegate. The ECGs will be classified as normal, abnormal not clinically significant, or abnormal clinically significant. All clinically significant findings will be recorded as AEs and may also be centrally evaluated by the vendor if necessary.

Abnormal ECGs that are NOT clinically significant may be repeated at the Investigator's discretion. Abnormal ECGs that ARE clinically significant will be repeated. In the case of evident bad quality (e.g., muscle tremor) of the tracing, an ECG will be repeated. If the QTcF values from the ECG shows a result above the threshold (i.e., ≥500 msec), then a single 12-lead

ECG in triplicate must be repeated at least hourly until QTcF values from 2 successive ECGs fall below the threshold. If QTcF values remain elevated after 2 successive ECGs (QTcF intervals do not return to < 500 msec or to < 60 msec above baseline), a cardiologist must be consulted and further dosing with study drug should be withheld until the participant can be assessed. Participants under those circumstances should not receive drugs known to prolong QTc interval (e.g., erythromycin, fluconazole, nelfinavir, chloroquine). If the participant has been taking ondansetron (Zofran®), this should also be noted.

All ECG tracings will be kept as part of the participant's permanent trial file at the site. Digital recordings will be analyzed and stored at a central ECG laboratory.

8.3.4. Clinical Laboratory Tests

A full list of clinical laboratory tests to be performed for this study is described in Section 10.2, Appendix 2. The actual date and times of sample collection must be recorded in source documents. Instructions for collection, handling, and shipment are found in the Site Procedural Manual.

In the event of an unexplained, clinically significant abnormal laboratory test result, the test should be repeated and followed up until it has returned to the normal range and/or an adequate explanation of the abnormality is found. Abnormal laboratory test results that are not clinically significant will be repeated at the Investigator's discretion. All clinically significant findings will be recorded as AEs (Section 8.4 and Section 10.3.1).

8.3.4.1. Clinical Laboratory Safety Tests

Blood samples for hematology, serum chemistry, and coagulation will be collected at selected timepoints throughout the study as defined in the SoAs (Section 1.3). All samples should be collected within the timepoints indicated in the SoAs.

All protocol-defined clinical laboratory safety assessments in the SoAs (Section 1.3) will be assessed by a central laboratory using that laboratory's normal ranges. Any additional laboratory tests determined as necessary, for safety reasons, by the Investigator should be performed by the central laboratory whenever possible but can be performed at a local laboratory for urgent participant assessment and/or care. The Investigator must review the laboratory report and document this review. All out of range (abnormal) laboratory values must be evaluated and commented on by the Investigator for clinical significance. Laboratory safety test results will be classified as normal, abnormal not clinically significant, or abnormal clinically significant. All clinically significant findings will be recorded as AEs (Section 8.4 and Section 10.3.1).

8.3.4.2. Other Clinical Laboratory Tests

Other clinical laboratory tests will be assessed by a central laboratory using that laboratory's normal ranges. The Investigator must review the laboratory report and document this review.

<u>Viral serology</u>: HIV-1 antibody, HIV-2 antibody, HbsAg, and HCV antibody and viral load testing (only if positive in HCV antibody test) will be performed at the Screening Visit. Participants will be counseled if any tests return a positive result. Participants who return a positive test result for HbsAg, HCV (positive in both antibody and viral load testing), or HIV at the Screening Visit will not be eligible to participate in the study.

Coronavirus disease 2019 (COVID-19) test: A COVID-19 test is performed during Screening and at check-in on Day 1 only if clinically indicated or per site policy; an antigen test is performed first and then polymerase chain reaction test for confirmation if clinically indicated. Participants with a positive COVID-19 result at Screening or on Day 1 will not be allowed to continue in the study. Participants who test positive for COVID-19 during participation in the study will be considered on a case-by-case basis for the best course of action per Investigator medical judgment.

Norovirus and influenza testing: Testing for norovirus and/or influenza may be performed as indicated by the Investigator.

Drugs of abuse: Urine drugs of abuse screen will be performed as indicated in the SoAs (Section 1.3).

8.3.5. Pregnancy Testing

Women of childbearing potential will have a urine human chorionic gonadotropin (hCG) pregnancy test at Screening to determine their eligibility for participation in this study. A urine hCG pregnancy test also will be conducted on Day 1, at intervals throughout the study, and at the EOS/Early Termination visit to confirm no exposure in utero. If a urine test is positive or inconclusive, pregnancy will be confirmed by a serum test.

During their participation in this study, participants must be instructed to notify their healthcare provider and the study Investigator if they or their partner becomes pregnant.

Serum follicle-stimulating hormone levels will be measured at screening in postmenopausal women ≤55 years of age to confirm postmenopausal status.

8.3.6. PHQ-9 and C-SSRS Questionnaires

Participants will complete a PHQ-9 and C-SSRS health questionnaire as indicated in the SoAs (Section 1.3). The Investigator will refer a participant to a Mental Health Professional or a Qualified Primary Care Professional if a participant has any of the following:

- A PHQ-9 score of ≥ 10
- Any suicidal behavior
- Any suicidal ideation of type 4 or 5 on the C-SSRS

In addition, the IDMC will review the aggregate data throughout the study. If a participant's psychiatric disorder can be adequately treated with psychotherapy and/or pharmacotherapy, then the participant, at the discretion of the Mental Health Professional or Qualified Primary Care Provider, can be continued in the trial on randomized therapy.

For additional information on the PHQ-9 assessment, please refer to the following reference: (Kroenke, et al., 2001). For additional information on the C-SSRS assessment, please refer to the following reference: (Project, 2016).

8.4. Adverse Events, Serious Adverse Events, and Other Safety Reporting

8.4.1. Adverse Events and Serious Adverse Events

An AE is any untoward medical occurrence in a clinical study participant that is administered a pharmaceutical product and does not necessarily have to have a causal relationship with this treatment. An SAE is any AE that results in death, is life-threatening, requires or prolongs an existing hospitalization, results in congenital anomaly/birth defect, results in persistent or significant disability/incapacity, and/or other important medical event. SAEs must be notified to Sponsor within 24 hours. Please refer to Section 10.3 for definition and reporting requirements.

8.4.2. Adverse Events of Special Interest

The following AEs are defined as adverse events of special interest (AESIs): AESIs of major adverse cardiovascular event (MACE), heart failure syndrome, acute pancreatitis, hypoglycemia, thyroid malignancies, supraventricular arrhythmia and cardiac conductive disorders, hepatobiliary disorders, severe GI AEs, acute renal events, depressive disorder and/or suicide ideation, hypersensitivity, and C-cell hyperplasia. All AESIs should be captured on the AE Case Report Form and followed until resolution, stabilization, or the participant is lost to follow-up (also see Section 7.3).

8.4.2.1. Major Adverse Cardiovascular Events

The MACE of special interest includes the following:

- Cardiovascular death
- Acute coronary syndrome
- Nonfatal stroke

Any event of MACE will be sent for adjudication (Section 8.4.4).

8.4.2.2. Heart Failure Syndrome

Criteria for heart failure syndrome include decreased functionality as defined by New York Heart Association, lung congestion and peripheral edema with elevated N-terminal pro-brain natriuretic peptide >400, and echocardiogram demonstrating wall motion findings consistent with heart failure, regardless of ejection fraction.

Any event of (suspected) heart failure syndrome will be sent for adjudication (Section 8.4.4).

8.4.2.3. Acute Pancreatitis

The diagnosis of acute pancreatitis requires 2 of the following 3 criteria:

- Abdominal pain consistent with acute pancreatitis (acute onset, severe, epigastric pain may radiate to the back)
- Serum amylase and/or lipase ≥ 3× ULN

- Characteristic findings of acute pancreatitis on computed tomography and/or magnetic resonance imaging

For any events of suspected acute pancreatitis, appropriate laboratory tests and/or imaging study should be performed. aleniglipron should be discontinued for any confirmed acute pancreatitis. Any suspected or confirmed pancreatitis should be managed at the Investigator's discretion.

Any event of (suspected) acute pancreatitis will be sent for adjudication (Section 8.4.4).

8.4.2.4. Hypoglycemia

Blood glucose is monitored by laboratory tests. Hypoglycemia is defined as blood glucose level <70 mg/dL (3.9 mmol/L).

Occurrence and risk for hypoglycemia should be reviewed at every visit and investigated as indicated. Any episode of hypoglycemia should be recorded in the eCRF. Hypoglycemia events are classified as 3 levels:

- Level 1: Glucose <70 mg/dL (3.9 mmol/L) and ≥54 mg/dL (3.0 mmol/L)
- Level 2: Glucose <54 mg/dL (3.0 mmol/L)
- Level 3: A severe event characterized by altered mental and/or physical status requiring assistance for treatment of hypoglycemia

Any events of level 3 hypoglycemia should be reported as an SAE. The hypoglycemia events should be managed at the Investigator's discretion.

8.4.2.5. Thyroid Malignancies and C-cell Hyperplasia

The C-cell function will be monitored by calcitonin. Participants with serum calcitonin level ≥ 20 ng/L if eGFR ≥60 mL/min/1.73 m^2 or ≥35 ng/L if eGFR <60 mL/min/1.73 m^2 at screening will be excluded, and aleniglipron will be discontinued for any participant who develops serum calcitonin level ≥ 20 ng/L (Section 7.1).

All out of range (abnormal) serum calcitonin level results should be repeated. If the values from the repeat testing are within the normal range, the participant may remain in the study.

Any event of (suspected) thyroid malignancies and C-cell hyperplasia will be sent for adjudication (Section 8.4.4).

8.4.2.6. Supraventricular Arrythmias and Cardiac Conductive Disorders

Defined as ECG findings of supraventricular arrythmias and/or cardiac conductive disorders. For any suspected supraventricular arrythmias and cardiac conductive disorders, 12-lead ECG should be performed and/or additional ECG or diagnostic tests should be performed at the Investigator's discretion.

8.4.2.7. Hepatobiliary Disorders

Liver function (Section 7.1.1) and potential drug-induced liver injury (DILI) (Section 10.6) should be closely monitored and managed.

Any events or suspected events related to hepatobiliary system, including gallbladder diseases, should be evaluated and additional diagnostic tests should be performed at the Investigator's discretion.

Any event of (suspected) cholecystitis demonstrated on ultrasound with or without cholelithiasis will be sent for adjudication (Section 8.4.4).

8.4.2.8. Severe Gastrointestinal Adverse Events

Severe GI AEs are defined as any AEs of GI system with severity of National Cancer Institute Common Terminology Criteria for Adverse Events (NCI-CTCAE) Grade 3 and above.

8.4.2.9. Acute Renal Events

Renal events will be assessed based on laboratory tests and any AEs/SAEs suggestive of new and/or progression of existing renal diseases. Participants and Investigators are advised to report and manage severe nausea, vomiting, and/or dehydration.

8.4.2.10. Depressive Disorder and/or Suicidal Ideation

Any AE of depressive disorder or suicidal ideation is considered as AESI.

8.4.2.11. Hypersensitivity

All allergic or hypersensitivity reactions should be reported as AEs or SAEs if serious criterion is met. Aleniglipron should be temporarily held in the event of suspected severe or serious allergic reactions. The study drug can be restarted if agreed by both Investigator and SMR.

8.4.3. Investigator-reported Outcomes

The definitions of AEs and SAEs can be found in Section 10.3, Appendix 3.

The Investigator and any medically certified delegates are responsible for detecting, documenting, recording, and reporting (as appropriate) events that meet the definition of an AE or SAE and remain responsible for following up of AEs that are serious, considered related to the study intervention, or that caused the participant to discontinue study drug or withdraw from the study (Section 7). This includes events reported by the participant (or, when appropriate, by a caregiver, surrogate, or the participant's legally authorized representative).

The method of recording, evaluating, and assessing causality of AEs and SAEs and the procedures for completing and transmitting SAE reports are provided in Section 10.3, Appendix 3.

8.4.3.1. Time Period and Frequency for Collecting Adverse Event and Serious Adverse Event Information

Detection and recording of study-related AEs and SAEs starts from the signing of the consent form until completion of the last study-related procedure (including follow-up for safety assessments). Detection and recording of TEAEs will start at the time of the first dose of study drug and continue until the last study procedure (Section 1.3).

Any pre-existing conditions or signs and/or symptoms present in a participant before any involvement in the study (i.e., before first dose of study drug) should be recorded as medical history.

In addition, any change in health status, which is reported after informed consent but started before informed consent by the participant, will be documented as medical history. Any worsening of a pre-existing condition that occurs following informed consent will be recorded as an AE.

Any AE reported or observed after the start of dosing with any study treatment until completion of the last study-related procedure (includes follow-up for safety assessments) will be recorded as a TEAE.

A poststudy AE/SAE is defined as any event that occurs outside of the nominal AE/SAE study detection period. Investigators are not obligated to actively seek AEs/SAEs in former study participants. However, if the Investigator learns of any SAE, including a death, at any time after a participant has completed the study and he/she considers the event reasonably related to the study treatment, the Investigator will promptly notify the Sponsor.

Participants with ongoing AEs may be allowed into the open-label extension following documented discussion with the SMR.

8.4.3.2. Method of Detecting Adverse Events and Serious Adverse Events

The Investigator (or medically certified delegate) is responsible for detection, recording, and reporting of events that meet the criteria and definition of AEs. As a consistent method of soliciting AEs, the participant shall be asked a nonleading question such as: “How do you feel?”.

8.4.3.3. Follow-up of Adverse Events and Serious Adverse Events

Following initial observation of an AE/SAE, the Investigator is required to proactively follow the progress of the relevant participant. Any information obtained in relation to the status of the AE/SAE and the condition of the participant must be appropriately documented in the eCRF and in a follow-up SAE Form where required.

All AEs and SAEs documented at a previous visit/contact that are designated as ongoing will be reviewed at subsequent visits/contacts. AEs/SAEs that have not resolved by EOS, or that have not resolved upon discontinuation of the participant’s involvement in the study, must be followed until any of the following occurs:

- The event resolves
- The event stabilizes
- The event returns to baseline, if a baseline value is available
- The event can be attributed to agents other than the study drug or to factors unrelated to study conduct
- It becomes unlikely that any additional information can be obtained (participant or health care practitioner refusal to provide additional information, lost to follow-up after demonstration of due diligence with follow-up efforts)

8.4.3.4. Pregnancy

In instances of pregnancies or suspected pregnancies identified or reported for any female participant (or male participant's female partner), including a positive pregnancy test regardless of age, following administration of study treatment, the pregnant female participant (or the male participant's female partner) will be advised to notify her healthcare provider and the study Investigator. Study drug administration will be discontinued immediately in the event of a reported (or suspected) pregnancy in a female participant.

Pregnancies should be documented on the Pregnancy Form and reported in the same manner as an SAE. The pregnancy will be followed up by the Sponsor (or its representative) through to the expected outcome. At the conclusion of the pregnancy, the outcome should be documented on the exposure in utero report if it is a normal birth (i.e., no SAE for mother or offspring). In the event of an SAE for either mother or offspring, the outcome must be documented on the SAE Form and transmitted within 24 hours according to the Safety Monitoring Plan in place for this study.

Informed consent will be sought from the pregnant female participant (or male participant's female partner) in order to allow the Investigator to access and review relevant medical records to conduct follow-up throughout the gestational period and on the infant following delivery. The Investigator shall follow up on newborn infants that have been exposed to IP in utero for a minimum of 12 months. Upon discovery of any congenital anomalies/birth defects (or neonatal deaths) the Investigator shall submit a follow-up report to the Sponsor or designee Officer using the Pregnancy Form, including information regarding the status of the newborn. A miscarriage or abortion shall also be reported by the Investigator to the Sponsor or designee using the Pregnancy Form.

8.4.4. Adjudication Committee for Selected Adverse Events

An independent external AC will be established to perform ongoing adjudication of selected types of events in randomized participants in a blinded manner. The AC is composed of permanent members covering all required medical specialties. The AC will be totally independent and will have no authorizations to impact trial conduct, trial protocol, or amendments.

The AC will evaluate events sent for adjudication using predefined definitions and guidelines detailed in the AC Charter.

<u>Events for adjudication</u>

MACEs (3-point MACE, including acute coronary syndrome, nonfatal stroke, and cardiovascular death), acute pancreatitis, thyroid cancer, and cholecystitis will be adjudicated, defined as following:

- Death due to cardiovascular cause
- Acute coronary syndrome to include unstable angina, defined as chest pain at rest, MI, which includes ST-elevated myocardial infarction (STEMI) and non-STEMI, and cardiovascular death, which includes sudden death witnessed and unwitnessed

- Stroke, hemorrhagic and ischemic, transient ischemic attacks, which include central neurologic deficits lasting up to 24 hours without sequela, and ministrokes with minor neurologic deficit lasting more than 24 hours with some permanent damage
- Heart failure syndrome, with decreased functionality as defined by New York Heart Association, lung congestion and peripheral edema with elevated N-terminal pro-brain natriuretic peptide >400, echocardiogram demonstrating wall motion findings consistent with heart failure, regardless of ejection fraction.
- Pancreatitis defined by mid abdominal and mid back pain with elevations in lipase and amylase and elevated white blood cell and platelet counts, with or without cholelithiasis
- Thyroid cancer based on biopsy pathology
- Cholecystitis demonstrated on ultrasound with or without cholelithiasis

Sources of events for adjudication include the following:

- Investigator-reported events for adjudication: When reporting AEs, the Investigator must send the appropriate AE category based on predefined criteria.
- AC-identified events: When reviewing documents of any event for adjudication, the AC can identify additional events in scope for adjudication that were not initially reported by the Investigator. In these instances, the Investigator will be notified of the newly identified event and has the option to report the AC-identified event. Regardless of whether the Investigator decides to report the event, it will undergo adjudication. The AC may require the Investigator to provide additional information and/or the Sponsor to perform a database search to identify potential events for adjudication.

If new information becomes available for an event sent for adjudication, it is the responsibility of the Investigator to ensure the new information is uploaded to the system. The adjudication outcome will be reported back to the Investigator.

Further information about this AC is provided in a charter, including a description of the scope of the committee’s responsibilities, the process and definitions to be utilized by the committee for adjudication, and the communication plan, including timelines.

8.4.5. Independent Data Monitoring Committee

In addition to routine trial monitoring, an external IDMC will monitor the safety data from this trial. The IDMC will have access to unblinded data as needed.

Further information about the IDMC is provided in the IDMC charter, including a description of the scope of the committee’s composition, responsibilities and governance, the process to be utilized by the committee, and the communication plan, including timelines.

8.5. Pharmacokinetics

Blood samples will be collected for assessment of plasma concentrations of aleniglipron and derived PK parameters. Clinical staff are required to collect each blood PK sample at the

scheduled timepoints within the windows indicated (Section 1.3). The actual date and time (as hh:mm) of each blood sample collection will be recorded in the eCRF.

All samples will be assayed by a validated bioanalytical method that is specific for the measurement of the analytes relevant to this study. The method validation conducted by the appointed bioanalytical laboratory will address (at a minimum) method accuracy, precision, reproducibility, specificity, recovery and sample stability, and other parameters as appropriate.

Samples of all participants who receive a dose of the active study drug treatments will be analyzed. Repeat analysis may be carried out according to predefined criteria as described in standard operating procedures of the bioanalytical laboratory conducting PK sample analysis for this study. Blood samples (3 mL per timepoint) will be processed with the resultant plasma divided into aliquots and placed into labeled plasma storage tubes. The samples will then be used for the analysis of aleniglipron plasma concentrations and determination of PK parameters.

Identification and quantification of aleniglipron plasma metabolites may be performed.

Instructions for collection, processing, storage, and transfer of blood samples are provided in the Site Procedural Manual.

8.6. Pharmacodynamics

Cardiovascular PD markers, including but not limited to, free fatty acid, IL-6, ProBNP, high-sensitivity C-reactive protein, and succinate, will be measured according to the Schedule of Assessments (Section 1.3).

8.7. Blood Sample Volumes

Blood samples will be collected from each participant throughout the study resulting in the approximate total blood volumes per participant as shown in Table 6. Additional blood may be collected from each participant at any time if required for safety reasons.

Table 6: Approximate Blood Sample Volumes to be Collected per Participant

Sample Type	Blood Volume per Participant not Participating in Open-Label Extension	Blood Volume per Participant Including Open-Label Extension For Participants who Were on Placebo
Hematology	22 mL (11 × 2 mL)	42 mL (21 × 2 mL)
Serum chemistry[a]	55 mL (11 × 5 mL)	105 mL (21 × 5 mL)
Coagulation	29.7 mL (11 × 2.7 mL)	56.7 mL (21 × 2.7 mL)
HbA1c	8 mL (4 × 2 mL)	12 mL (6 × 2 mL)
Viral serology	7 mL (1 × 7mL)	7 mL (1 × 7mL)
PK samples	48 mL (16 × 3 mL)	54 mL (18 × 3 mL)
Cardiovascular PD samples	22.5 mL (3 × 7.5 mL)	37.5 mL (5 × 7.5 mL)
**Total blood volume collected per participant**	**192.2 mL**	**314.2 mL**

Abbreviations: FSH=follicle-stimulating hormone; HbA1c=hemoglobin A1c; PD=pharmacodynamic; PK=pharmacokinetic

a. Also includes serum pregnancy and FSH testing, where required.

9. STATISTICAL CONSIDERATIONS

Detailed methodology for summaries and statistical analyses of the data collected will be documented in a separate Statistical Analysis Plan (SAP) that will be finalized before database lock and unblinding. Any changes to the methods described in the final SAP will be described and justified as needed in the CSR.

This section describes the general framework to be used for the analysis and presentation of data in this study. The information described in this section may be revised during the study to accommodate protocol amendments and to make changes to adapt to unexpected issues in study execution and data that could affect planned analyses. All available data will be included in data listings. Data tabulations will be performed for specific analysis populations.

The study hypothesis for the primary objective is that at least 1 dose level of aleniglipron is superior in percent change from baseline in body weight to placebo at Week 36 in participants without diabetes who have obesity or are overweight with at least 1 weight-related comorbidity.

Treatment comparisons will be tested for the primary objective at 2-sided significance level of 0.05. No multiplicity adjustments will be made for the analysis of secondary and exploratory objectives.

For the open label extension, the primary objective is to evaluate the long-term safety and tolerability of aleniglipron in adults with obesity or overweight with a comorbidity up to Week 72 at doses of aleniglipron up to 120 mg.

Details of the analysis plan for both the Double Blind (Week 0-36) and OLE extension periods (Week 36-72) are provided in separate SAPs for each period.

9.1. Analysis Sets

For the purposes of analysis, the following analysis sets are defined:

Participant Analysis Set	Description
All Randomized Set	All participants who are randomly assigned to a treatment group.<br>Participant disposition and background data, including demographics, baseline characteristics, and participation data will be presented for all participants in the All Randomized Set.
Full Analysis Set	All randomized participants who receive ≥1 dose of study drug (aleniglipron or placebo).<br>Efficacy data will be presented for all participants in the Full Analysis Set.
Safety Analysis Set	All participants who receive ≥1 dose of study drug (aleniglipron or placebo).<br>Safety and tolerability data will be presented for all participants in the Safety Analysis Set.
Pharmacokinetic Analysis Set	All participants in the Safety Analysis Set who have at least 1 quantifiable plasma concentration.<br>Concentration data will be presented for all participants in the PK Analysis Set.
Pharmacodynamic Analysis Set	All participants in the Safety Analysis Set who have sufficient data to derive ≥1 PD parameter.

Abbreviations: PD=pharmacodynamic; PK=pharmacokinetic.

9.1.1. Open-Label Extension Analysis Set

For the open-label extension analysis, the following analysis sets are defined:

Participant Analysis Set	Description
OLE Analysis Set	All participants who receive ≥1 dose of study drug (aleniglipron) in the OLE phase.<br>Participant disposition and background data, including demographics, baseline characteristics, participation data, efficacy data, and safety and tolerability data will be presented for all participants in the OLE Analysis Set.
Safety Analysis Set	All participants who receive ≥1 dose of study drug (aleniglipron or placebo) in the entire study.<br>Safety and tolerability data will be presented for all participants in the Safety Analysis Set.
Pharmacokinetic Analysis Set	All participants in the OLE Analysis Set who have at least 1 quantifiable plasma concentration.<br>Concentration data will be presented for all participants in the PK Analysis Set.
Pharmacodynamic Analysis Set	All participants in the OLE Analysis Set who have sufficient data to derive ≥1 PD parameter.

9.2. Statistical Analyses

Unless stated otherwise, all statistical summaries and analyses will be conducted based on planned randomized treatment group, regardless of the actual treatment received by the participant due to any dose modification. For safety summaries based on the Safety Analysis Set, if a participant randomized to the placebo treatment group accidentally received any aleniglipron, this participant will be included in the aleniglipron group in that cohort. Participants assigned to placebo in Cohorts 1 to 3 will be pulled together as the treatment group of placebo.

All tests of treatment effects will be conducted at a 2-sided alpha level of 0.05, and confidence intervals (CIs) will be calculated at 95%, 2-sided. All tests of interactions between treatment groups and other factors will be conducted at a 2-sided alpha level of 0.10.

9.2.1. Participant Disposition

Summaries of study disposition and study drug disposition for all randomized participants will be provided by treatment group. Corresponding listing will be provided. Counts and percentages of participants who were randomized, discontinued early from the study, study treatment and completed the study, as well as those who experienced dose interruption or dose reduction, will be summarized by treatment group and in total based on the All Randomized Set. Primary reasons for premature study drug or study discontinuation will also be summarized.

9.2.2. Efficacy Data

A restricted maximum likelihood-based mixed models for repeated measures (MMRM) analysis will be used to analyze continuous longitudinal variables. The models will incorporate treatment group, randomization strata, visit, treatment-by-visit interaction, and baseline weight values as covariates. All scheduled timepoints will be included in the MMRM models. Participants will be considered as repeated measure units over visits. Linear contrast from the model will be used to test the treatment difference between each dose group and placebo at each timepoint. Test p-values, point estimates, and corresponding 95% CIs of treatment difference will be provided at each timepoint using the MMRM analysis based on least squares means and Type III tests. In the MMRM model, missing data will not be imputed. This analysis is unbiased under the missing at random assumption. An unstructured covariance will be used to model the within-subject variance-covariance structure for the model errors. If convergence fails, a Toeplitz covariance matrix will be used. If Toeplitz covariance matrix does not converge, a first order autoregressive, AR(1), covariance matrix will be used. If convergence is still not met, then compound symmetry will be used.

9.2.2.1. Primary Endpoint

The primary efficacy assessment on percent change from Baseline in body weight at Week 36 will be guided by the primary and secondary estimands and conducted using the Full Analysis Set.

For the primary estimand, the hypothetical strategy is used to handle the ICEs, so only data collected before the occurrence of the ICEs will be used in the MMRM analysis (Section 1.1).

For the secondary estimand, the treatment policy strategy is used to handle the ICEs; that is, all observed values including those collected after ICEs will be used in the analysis regardless of the occurrence of the ICEs.

Primary efficacy comparison will be based on the contrast between each treatment group of aleniglipron (45, 90, or 120 mg QD) and placebo at Week 36 from the MMRM analysis of percent change from baseline in body weight. More detail will be provided in the SAP.

9.2.2.2. Secondary Endpoints

The following secondary endpoints will be analyzed on Full Analysis Set:

- Percentage of participants who achieve ≥5% reduction in body weight at Week 36
- Percentage of participants who achieve ≥10% reduction in body weight at Week 36
- Percentage of participants who achieve ≥15% reduction in body weight at Week 36
- Change in body weight (absolute) from Baseline to Week 36
- Change in BMI from Baseline to Week 36
- Change in waist circumference from Baseline to Week 36

Change from Baseline in continuous variables will be analyzed by the MMRM model for primary and secondary estimands as described Section 9.2.2.1.

Treatment comparisons for the percentage of participants with ≥5%, ≥10%, and ≥15% reduction in body weight will be analyzed using a logistic regression model with treatment group, randomization strata, and baseline weight value as covariates. Missing values for such binary endpoints will be imputed by categorizing the imputed values of the missing body weight at Week 36.

9.2.2.3. Exploratory End Points

A descriptive characterization will be produced for the following exploratory end points including, but not limited to:

- Change in triglycerides, free fatty acids (FFA), and high-sensitivity C-reactive protein (hsCRP) to Week 36
- Change in patient-reported outcomes (SF-36 acute version, IWQOL-Lite CT, and hunger/satiety evaluation) from Baseline to Week 36
- Change in HDL and LDL cholesterol from Baseline to Week 36
- Change in resting blood pressure from Baseline to Week 36
- Change in fasting glucose from Baseline to Week 36

Exploratory endpoints also include evaluation of drug exposure via population pharmacokinetics. Results may be reported in a separate document.

9.2.2.4. Open-Label Extension Endpoints

A descriptive characterization will be produced for the following endpoints to assess the safety and effectiveness of aleniglipron including, but not limited to:

- Safety and tolerability including TEAEs, SAEs, AESI, laboratory parameters, ECGs, and vital signs
- Percent change in body weight
- Percentage of participants who achieve ≥5%, ≥10% or ≥15% reduction in body weight
- Change in body weight (absolute)
- Change in waist circumference
- Change in body mass index
- Exploratory endpoints including change in triglycerides, FFA, hsCRP, IWQOL-Lite CT, HDL, LDL, resting blood pressure, and fasting glucose

More detail will be provided in the SAP.

9.2.3. Demographic and Baseline Characteristics

Demographic data (including sex, age, race, and ethnicity) will be summarized using descriptive statistics (n, mean, standard deviation [SD], median, minimum, and maximum) and tabulated by dose group, and overall on the All Randomized Set.

Height, weight, and BMI will be summarized using descriptive statistics (n, mean, standard SD, median, minimum, and maximum) and tabulated by dose group and overall.

Medical history will be coded using the latest version of Medical Dictionary for Regulatory Activities (MedDRA). The number of participants reported with conditions/procedures and the number of conditions/procedures reported will be summarized by treatment and overall, grouped according to system organ class (SOC) and preferred term, using descriptive statistics.

9.2.4. Safety/Tolerability

Unless specified otherwise, safety and tolerability assessments will be guided by an estimand comparing safety of aleniglipron doses with placebo irrespective of adherence to study drug including data collected during the treatment period plus safety follow-up from participants who are randomized and are exposed to at least 1 dose of study drug. Thus, safety analyses will be conducted using the Safety Analysis Set.

Safety evaluations will be based on the incidence, severity, and relationship of AEs, vital signs, physical examination findings, clinical laboratory results, and ECGs.

Abnormalities in clinical laboratory parameters and the ECG parameter QTcF will be based on predefined normal ranges and will be tabulated by dose group showing participant counts and percentages.

9.2.4.1. Extent of Exposure

Study drug administration will be listed by participant. Duration of treatment and drug compliance (% of dosage and % of tablet) will be presented by treatment group and overall with descriptive statistics, as well as counts and percentage of participants with exposure and compliance in certain categories.

Days of exposure to study drug will be calculated as date of last dose of study drug – date of first dose of study drug + 1. Percent of compliance to the study drug regimen will be calculated as 100 × number of actual tablets taken / number of expected tablets taken.

9.2.4.2. Adverse Event

All AEs will be coded using the latest version of MedDRA by SOC and preferred term, classified from verbatim terms. The count and percentage of participants with TEAEs will be summarized by SOC and preferred term and tabulated for each dose group. Summaries of TEAEs by severity and relationship will also be presented. Summaries will also be presented for AESI (Section 8.4.2), AEs leading to death, SAEs, AEs leading to dose interruption, AEs leading to dose reduction, and for AEs leading to study withdrawal. The duration, treatment, and outcome of AEs will be determined and included in the listings.

9.2.4.3. Prior and Concomitant Medication

Prior and concomitant medications will be coded using the latest version of the World Health Organization Drug Dictionary (WHO-DD). The number and percentage of participants taking prior medication and concomitant medication will be summarized using frequency tables according to WHO Drug Anatomical Therapeutic Chemical Level 1 Code Description and the WHO-DD Preferred Name. If a participant has >1 prior or concomitant medication coded to the same category, the participant will be counted only once.

9.2.4.4. Vital Signs

Observed values and changes from baseline for vital signs (systolic and diastolic BP, HR, body temperature) will be summarized at each scheduled timepoint using descriptive statistics (n, mean, SD, median, minimum, and maximum) and tabulated for each dose group.

9.2.4.5. Physical Examination Data

Physical examination data will be listed only. It is noted that any postdose clinically significant findings will be reported as AEs. Body weight and BMI changes from baseline compared with placebo will be assessed, details of which will be addressed in the SAP.

9.2.4.6. Clinical Laboratory Data

Clinical laboratory safety data will be summarized by the laboratory parameter. Normal reference ranges and abnormal results will be used in the summary of laboratory data. Observed values and changes from baseline for continuous clinical laboratory parameters will be summarized at each scheduled timepoint using descriptive statistics (n, mean, SD, median, minimum, and maximum) and tabulated for each dose group. Categorical clinical laboratory data will be summarized at each scheduled timepoint using participant counts and percentages and tabulated by dose group. Shift tables presenting the number and percentage of participants with a normal value at baseline and a postbaseline abnormal value will also be summarized by treatment group. Individual participant profiles will be presented for any laboratory parameters with ≥1 postdose value outside the laboratory’s reference ranges and deemed clinically significant.

9.2.4.7. Electrocardiogram Data

The following ECG parameters will be analyzed: PR interval, QT interval, QRS duration, QTcF, and ventricular HR. Observed values and changes from baseline for ECG parameters will be summarized at each scheduled timepoint using descriptive statistics (n, mean, SD, median, minimum, and maximum) and tabulated for each dose group.

For QTcF, the number of participants with values >450, >480, and >500 msec or an increase from baseline >30 and >60 msec will also be presented, in accordance with International Council for Harmonization of Technical Requirements for Pharmaceuticals for Human Use (ICH) E14 (note for guidance on the clinical evaluation of QT/corrected QT interval prolongation and proarrhythmic potential for non-antiarrhythmic drugs, Committee for Medicinal Products for Human Use/ICH/2/04).

9.2.5. Pharmacokinetic Data

9.2.5.1. Plasma Pharmacokinetic Parameters

Plasma concentrations of aleniglipron at specified timepoints predose and postdose will be used to calculate PK parameters, via population PK analysis.

Individual participant aleniglipron plasma concentrations at each timepoint will be listed and summarized by treatment using descriptive statistics (n, mean, SD, coefficient of variation, minimum, maximum, median, geometric mean and coefficient of variation of the geometric mean) for each dose group. Individual and mean (by treatment) plasma concentration-time data may be presented graphically.

Plasma concentration-time data of aleniglipron may be used for the calculation of the following PK parameters using population PK:

- C_{max}
- T_{max}
- AUC_{0-tau}
- Accumulation Ratio

PK parameters and analysis might be described in a separate report.

Plasma samples may be used for identification of aleniglipron metabolites. Metabolite identification data will be used for internal exploratory purposes and will not be included in the CSR.

9.2.6. Pharmacodynamic Data

The PD effect of aleniglipron will be assessed based on several variables, including, but not limited to, triglycerides, FFA, cholesterol, hsCRP, and body weight.

9.2.7. Subgroup Analyses

Subgroup analyses of the primary endpoint will be made to assess consistency of the treatment effect across the following subgroups using the primary estimand:

- Age group: <45, ≥45 to <55, ≥55 to <65, ≥65
- Sex: female versus male
- Baseline BMI: <30, ≥30 to <35, ≥35 kg/m^2
- Pre-diabetes status (HbA1c <5.7% versus ≥5.7%)
- Race
- Ethnicity

If the number of participants is too small (<10%) within a subgroup, then the subgroup categories may be redefined prior to unblinding the study. Further details on the statistical analysis will be provided in the SAP.

For the subgroup analysis, the same fixed effects given for the primary analysis model plus factors of subgroup, 2-way interaction of subgroup and treatment, 2-way interaction of subgroup and visit, and 3-way interaction of treatment, visit, and subgroup. The interaction of subgroup and treatment at the primary efficacy endpoint will be evaluated to assess the treatment by subgroup interaction. Additional subgroup analyses may also be performed.

9.3. Interim Analysis

An interim (efficacy and safety) analysis may be conducted to inform dose escalation schemes to accelerate the development of future studies after all participants complete the Week 26 visit (when participants have completed titration and safety and tolerability has been observed for at least 4 weeks at each dose level). There will be no action taken with respect to the study conduct and analysis, including stopping the study based on the superiority of aleniglipron versus placebo of the interim analysis. There will be no inflation of the Type 1 error rate and no need to employ an alpha spending function or multiplicity adjustment. An independent data center will carry out the analysis and the Sponsor will review the interim analysis for safety and efficacy. Details of the interim analyses, operational support, and unblinding will be specified in the SAP.

9.4. Sample Size Determination

Approximately 220 participants will be randomly assigned to active treatment and placebo in a randomization ratio of 3:1 in all 3 cohorts in the double-blind period of the study. All participants assigned to placebo in each cohort will be pulled together as the placebo group, which will be compared to each aleniglipron treatment group in Cohort 1 to 3 to establish efficacy. The sample size calculation is based on the primary estimand and its endpoint (percent body weight change from Baseline to Week 36). Assuming an SD of 6%, a 2-sided alpha level of 0.05, 35 completers for one aleniglipron treatment group and 35 completers for the placebo group can provide approximately >90% power to detect a treatment difference of -5% between the aleniglipron treatment group and placebo in percent body weight change from Baseline to Week 36. Assuming a 20% dropout rate in aleniglipron 45 mg group, 60 participants will be randomized for Cohort 1, with 45 participants on aleniglipron 45 mg and 15 participants on placebo. Assuming a 35% dropout rate in all other treatment groups, 80 participants will be randomized for each of Cohort 2 and 3, with 60 participants on aleniglipron and 20 participants on placebo.

10. SUPPORTING DOCUMENTATION AND OPERATIONAL CONSIDERATIONS

10.1. Appendix 1: Regulatory, Ethical, and Study Oversight Considerations

10.1.1. Regulatory and Ethical Considerations

10.1.1.1. Investigator Responsibilities

The Investigator must conduct the clinical study in accordance with the Institutional Review Board / Ethics Committee (IRB / EC)-approved study protocol and the Good Clinical Practice (GCP) regulations, applicable in the jurisdiction(s) in which the study is conducted and, in which, the Sponsor intends to submit the study for regulatory consideration and/or marketing authorization.

10.1.1.2. Ethical Considerations

The Investigator(s) and all parties involved in this study will conduct the study in the spirit of the ethical principles set forth in the Declaration of Helsinki, and the current ICH GCP guidelines.

Conduct of the study must be approved by an appropriately constituted IRB / EC before participants are enrolled. Prior to initiation of the study, written IRB / EC approval of the protocol and ICFs, based on the principles of current ICH GCP will be received. This approval must be issued on the IRB / EC letterhead and refer to the ICFs and to the study protocol by title and protocol number and include date of each document.

A copy of the signed and dated letter of approval will be emailed to the Sponsor or designee before commencement of the study. Any written information and/or advertisements to be used for participant recruitment must be approved by the IRB / EC before use. A list of the IRB / EC voting members, their titles or occupations, and their institutional affiliations will also be provided to the Sponsor's representative before study initiation. If approval is suspended or terminated by the IRB / EC, the Investigator will notify the Sponsor immediately.

Protocol amendments that may impact participant safety or the validity of the study will be approved by the IRB / EC, following written agreement for the amendment from the Sponsor. The amended protocol will not be implemented at sites until such approvals are received other than in the case of an urgent safety measure.

The Investigator must maintain an accurate and complete record of all submissions made to the IRB / EC. The records should be filed in the Investigator Site File and copies must be sent to the Sponsor and maintained in the study Trial Master File.

The trial will be submitted to required clinical trial registries (www.clinicaltrials.gov).

The Sponsor will ensure that participants will be informed, through the ICF, of the availability of medical care or compensation for injury (if any) for more than minimal risk research.

10.1.2. Informed Consent Process

The Investigator is responsible for obtaining informed consent from each participant participating in the trial in compliance with US Title 21 Code of Federal Regulations (21 CFR) Part 50, and ICH and IRB / EC guidelines.

Prior to initiation of the trial at the site, the ICF must be reviewed and accepted by the Sponsor and approved by the governing IRB / EC. The Investigator or medically certified delegate will discuss the purpose and pertinent details of the trial with each potential participant, provide sufficient time to consider the study, and if the potential participant understands and agrees to participate, sign, and date the appropriate IRB / EC-approved ICF before undergoing any trial-specific procedures. The ICF must be personally signed and dated by the participant and by the person who conducted the informed consent discussion. Additional signature requirements may exist. The original signed and dated ICF will be retained with the participant's trial records, and a copy of the signed ICF will be given to the participant. The Investigator or medically certified delegate will maintain a log of all participants who sign the ICF. At a minimum, the log will include a participant identifier, the dates of informed consent and screening procedures, the outcome of the screening, and the reason the participant did not enroll in the trial, if applicable.

Should a protocol amendment become necessary, the ICF may need to be revised to reflect the changes to the protocol. It is the responsibility of the Sponsor or designee to ensure that an amended ICF is reviewed and has received favorable opinion from the IRB / EC, and the Investigator has to ensure that the amended ICF is signed by all participants subsequently entered in the trial and those currently in the trial, if affected by the amendment.

10.1.3. Data Protection

The Investigator is responsible for maintaining the privacy and confidentiality of the participant's medical or health information collected during the trial. The Investigator is also responsible for ensuring that all use, review, and disclosure of participant's medical or health information is in accordance with the Health Insurance Portability and Accountability Act regulations and the ICF approved by the IRB / EC and signed by the participant. Specifically, all data collected about a participant during the trial will be identified only by a number.

10.1.4. Study Protocol Guidelines

10.1.4.1. Emergency Contact With Investigator

Suitable arrangements must be made for participants to contact the Investigator or medically certified delegate in the event of an emergency.

10.1.4.2. COVID-19 Preparedness

The clinical site should have in place procedures and strategies to accommodate the current COVID-19 preparedness. Such procedures should include requirements in relation to criteria, such as the following:

- Attendance (e.g., who is permitted to be on-site, limitations, records of attendance, plans for suppliers and deliveries)
- Physical layout (e.g., physical distancing requirements and signage)

- Flexibility (e.g., procedures for scaling, ability to respond to outbreak)
- Support for remote interactions with Sponsors and study teams (e.g., communication, including infrastructure)
- The local environment (e.g., contact with local health authorities, ability to access up-to-date information)
- Any other relevant criteria

10.1.4.3. Protocol Deviations and Violations

Investigators and study site personnel must not deviate from the protocol unless it is absolutely necessary to avoid an immediate hazard to the study participants. The Investigator is responsible for complying with all protocol requirements and applicable regulations regarding protocol deviations. The Sponsor must be notified of all deviations to the protocol (e.g., inclusion/exclusion criteria, dosing, missed study visits) to determine the impact of the deviation on the participant and/or the study.

The Investigator should notify the IRB / EC of significant deviations from the protocol in accordance with local procedures.

10.1.4.4. Protocol Waivers

Protocol waivers will not be granted by the Sponsor in this study.

10.1.4.5. Protocol Amendments

Administrative amendments to the protocol will be classed as amendments of typographical errors, clarifications of confusing wording, and other minor modifications, including, but not limited to, name, address, and contact information changes that have no impact on the safety of the participant or the science of the study. Administrative amendments will be submitted to the IRB / EC for information only. The Sponsor (or designee) will ensure that acknowledgement is received and filed. Otherwise, an amendment will be classed as a substantial amendment and will be submitted to the IRB / EC for approval and the appropriate regulatory authorities, as applicable.

10.1.5. Quality Control and Assurance

10.1.5.1. Study Monitoring

The Sponsor is responsible for assuring the proper conduct of the study with regard to protocol adherence and validity of the data recorded in the eCRF.

During the course of the trial, the Sponsor and/or designee will visit the trial sites and/or perform remote monitoring at regular intervals. The Investigators must make themselves available to the Study Monitor during an on-site (or remote) monitoring visit to enable discussion on any arising issues relating to the study. The purpose of the monitoring (on-site or remote) is to ensure that the eCRF is completed correctly and the protocol is being adhered to, to perform source data verification, and to monitor drug accountability.

The Investigator agrees to allow the Study Monitor direct access to relevant source documents (original documents, data, and records). Direct access includes permission to examine, analyze, verify, and reproduce any record(s) and report(s) that are important to evaluation of the clinical trial. The Investigator also agrees to allocate his/her time and the time of his/her staff to the Study Monitor to discuss findings and any relevant issues.

Participant confidentiality must be maintained during any monitoring activities conducted by the Sponsor or designee.

In accordance with applicable regulations and ICH GCP, a Study Monitor will contact the site to organize an on-site (or remote) visit before participant enrolment to review the protocol and data collection procedures with site staff. In addition, the assigned Study Monitor will periodically contact the site, including conducting on-site (or remote) visits. The extent, nature, and frequency of on-site visits will be based on such considerations as the study objectives and/or endpoints, the purpose of the study, study design complexity, and enrolment rate.

During these site visits, the Study Monitor will do the following:

- Check the progress of the study.
- Review study data collected.
- Conduct source document verification.
- Identify any issues and address their resolution.
- Check study treatment accountability records.
- Review biological samples collected for the study and ensure that they are labeled and stored correctly.
- Verify the following:
  - The data are authentic, accurate and complete
  - Safety and rights of participants are being protected
  - Study is conducted in accordance with the currently approved protocol (and any amendments), ICH GCP, and all applicable regulatory requirements

At study closure, a Study Monitor will conduct the following activities in conjunction with the Investigator or site staff as appropriate:

- Ensure that all data queries have been resolved
- Conduct final accountability and reconciliation for study treatment supplies, including inventory and final disposition (e.g., destruction, shipping to repository, etc.)
- Review of site study records for completeness

10.1.5.2. Quality Assurance

To ensure compliance with all applicable GCP regulations, the Sponsor (or a designated third party), may conduct a quality assurance audit of the study site. Regulatory agencies may also conduct a regulatory inspection of this study. Such audits/inspections can occur at any time during or after completion of the study. If an audit or inspection occurs, the Investigator and

Institution agree to notify the Sponsor as soon as possible following awareness of an impending regulatory inspection. The Investigator and Institution agree to allow the auditor/inspector direct access to all relevant documents and allocate his/her time and the time of his/her staff to the auditor/inspector to discuss findings and any relevant issues.

The Sponsor will perform or oversee the performance of the quality assurance and quality control activities for this study according to GCP and standard operating procedures; however, responsibility for the accuracy, completeness, and reliability of the study data presented to the Sponsor lies with the Investigator (and delegate[s]) generating the data.

Prior to the study initiation, the Sponsor or Sponsor's representative will explain the protocol, Investigator's Brochure, and eCRF to the Investigator and the site staff involved in this study. In addition, during and after the trial, periodic site visits will be made to monitor for compliance, including verification of the accuracy and completeness of data recorded on the eCRFs, source documents, and trial treatments accountability records.

10.1.6. Dissemination of Clinical Study Data

After the EOS, a CSR will be written and submitted to relevant IRB / EC and regulatory authorities in accordance with local requirements.

10.1.7. Source Documents

Source documentation must be prepared and available for Sponsor review prior to commencement of enrollment activities and must capture, at a minimum, the following aspects and parameters:

- Adherence to protocol procedures
- Participant identification, eligibility, and participation
- Informed consent procedures
- Protocol specified assessments
- Dates of visits
- Safety parameters, including reporting and follow-up of AEs and use of concomitant medication
- PK and PD parameters
- Study drug receipt/dispensing/return records
- Study drug administration information
- Date of participant completion
- Discontinuation from treatment or withdrawal from the study and the reason, if appropriate

The author of an entry in the source documents must be identifiable. Worksheets may be used for the capture of data to facilitate completion of the eCRF. Any such worksheets will become part of the participants' source documentation.

Following the ICH GCP guidelines, direct access to source documentation and medical records must be allowed for the purpose of verifying that the data recorded in the eCRF are consistent with the original source data.

10.1.8. Electronic Case Report Form

The Sponsor will designate a CRO to perform data management. eCRFs will be used at the clinical trial site to collect trial data for enrolled participants. When data are available, authorized clinical trial site personnel will carefully and accurately record the data on the eCRF. Sites must ensure that all source documents are maintained according to ICH/GCP guidance and support the data that are entered onto the eCRFs. The eCRF data will be captured in a system validated according to procedures that comply with Food and Drug Administration 21 CFR Part 11 and the ICH E6(R2) Guidelines for GCP. The eCRFs will be reviewed and signed by the Investigator or someone clinically qualified and identified on the delegation log as someone who can sign-off on the eCRF.

The CRO will write a data management plan outlining the data management systems, procedures, and agreements between the CRO and Sponsor. The plan will be reviewed and signed by a representative of the Sponsor's data management department. Outside the electronic data capture system, when appropriate, the CRO will receive Food and Drug Administration (FDA) 21 CFR Part 11 compliant external laboratory data transfers from a validated laboratory information management system. After database lock, the Investigator will receive a copy of the participant data for archiving at the trial site.

10.1.9. Recordkeeping and Retention of Records

In compliance with the ICH GCP guidelines, the Investigator/institution will maintain all source documents that support the data collected from each participant, as well as all study documents as specified in ICH E6(R2) GCP, Essential Documents for the Conduct of a Clinical Trial. The Investigator/institution will take measures to prevent accidental or premature destruction of these documents.

The Investigator must retain all records relating to the conduct of this trial for ≥2 years after a marketing application for the drug is approved; or if an application is not filed or not approved for the drug, for ≥2 years after clinical development for the drug has been formally discontinued, and the appropriate regulatory or health authorities have been notified. However, in certain instances, documents may need to be retained for a longer period if required by regulatory requirements or by an agreement with the Sponsor. The Investigator must allow representatives of the FDA, the governing IRB / EC, or other regulatory agencies to inspect all trial records. If informed of such an inspection, the Investigator will notify the Sponsor immediately. The Investigator must obtain written approval from the Sponsor prior to the destruction of any records relating to the conduct of this trial. If the responsible Investigator retires, relocates, or for other reasons withdraws from the responsibility of keeping the study records, custody must be transferred to a person who will accept the responsibility. The Sponsor must be notified in writing of the name and address of the new custodian.

Under no circumstance shall the Investigator relocate or dispose of any study documents before having obtained written approval from the Sponsor.

10.1.10. Study and Site Start and Closure

10.1.10.1. Notification of the General Practitioner

It is the responsibility of the Investigator, or medically certified delegate, to notify, where applicable, the participant's general practitioner of the participant's involvement in the trial. This notification should include the nature of the trial, treatments, expected benefits, AEs, and concomitant drugs to be avoided. The consent of the participant should be sought before contacting their general practitioner.

10.1.10.2. Clinical Laboratory Certification and Reference Ranges

Before the initiation of this study, the Investigator, or medically certified delegate, will obtain a copy of the certification form, with certification number and expiration date for all clinical laboratories used in the study. Reference ranges for each clinical laboratory test used in this study will be obtained from the laboratory that will perform the test during the study.

10.1.10.3. Study/Site Termination

The Sponsor reserves the right to do the following:

- Suspend or terminate execution of this clinical study at any time
- Suspend or terminate participation of an investigational site of this clinical study at any time

Reasons will be provided in the event of any of the above occurrences taking place. Reasons for termination may include but are not limited to the following:

- Unacceptable safety and tolerability
- Serious or persistent noncompliance by the Investigator with the protocol, clinical research agreement, principles of the ICH GCP, or applicable regulatory guidelines in conducting the study
- IRB / EC decision to terminate or suspend approval for the Investigator
- Regulatory authority request for study suspension or termination
- Enrollment is unsatisfactory

The Investigator reserves the right to withdraw from the study for safety reasons at any time following consultation with the Sponsor.

10.1.10.4. Study Completion/Site Closure

The study is considered completed at the last contact of the last participant involved in the study. The final data from the investigational site will be sent to the Sponsor (or designee) in the time frame specified in the Clinical Trial Agreement. Investigational sites will be closed upon study completion. An investigational site is considered closed when all required documents and study supplies have been collected and a site closure visit has been performed by the Sponsor (or designee).

10.1.10.5. EOS and Regulatory Notification

At EOS, the IRB / EC and relevant regulatory authorities will be notified by the Sponsor (or designee) according to applicable IRB / EC and regulatory requirements.

10.1.11. Financing and Insurance

Financial aspects of the study are addressed in a separate clinical study agreement.

The Investigator is required to have adequate current insurance to cover claims for negligence and/or malpractice.

The Sponsor will provide insurance coverage for the clinical study as required by national regulations. The Sponsor will be subscribed to an insurance policy covering, in its terms and provisions, its legal liability for injuries caused to participating persons and arising out of this research performed strictly in accordance with the study protocol as well as with applicable law and professional standards prior to commencement of the study.

10.1.12. Intellectual Property, Confidentiality, and Publications

10.1.12.1. Ownership

All information provided by the Sponsor and all data and information generated by the clinical site staff as part of the study (other than a participant's medical records) are the sole property of the Sponsor.

All rights, title, and interests in any inventions, know-how, or other intellectual or industrial property rights that are conceived or reduced to practice by clinical site staff during the course of, or as a result of, the study are the sole property of the Sponsor and are hereby assigned to Gasherbrum Bio, Inc.

If a written contract, which includes ownership provisions inconsistent with this statement for the conduct of the study, is executed between the Sponsor and the clinical site, that contract's ownership provisions shall apply rather than this statement.

10.1.12.2. Confidentiality

All information provided by the Sponsor and all data and information generated by the clinical site as part of the study will be kept confidential by the Investigator and other site staff. The Investigator or other site personnel will not use this information and data for any purpose other than conducting the study. These restrictions do not apply to the following:

- Information that becomes publicly available through no fault of the Investigator or site staff
- Information that is necessary to disclose in confidence to an IRB / EC solely for the evaluation of the study
- Information that is necessary to disclose in order to provide appropriate medical care to a study participant
- Study results that may be published as described in the Publication Policy (Section 10.1.12.3)

If a written contract, which includes confidentiality provisions inconsistent with this statement for the conduct of the study, is executed, that contract's confidentiality provisions shall apply rather than this statement.

10.1.12.3. Publication Policy

The Sponsor may publish the results of this study at an appropriate time. No publication of the results shall take place without the express consent of the Sponsor. Prior to submitting for any publication, presentation, use for instructional purposes, or otherwise disclosing the study results generated by the sites (collectively, a "Publication"), the Investigator shall provide the Sponsor with a copy of the proposed publication and allow the Sponsor a period of ≥30 calendar days (or for abstracts, ≥15 working days) to review the proposed publication. Proposed publications shall not include Sponsor confidential information.

At the request of the Sponsor the submission or other disclosure of a proposed publication will be delayed for a sufficient length of time to allow the Sponsor to seek patent or similar protection of any inventions, know-how or, other intellectual or industrial property rights disclosed in the proposed publication.

If a written contract, which includes publication provisions inconsistent with the statement for the conduct of the study, is executed, that contract's publication provisions shall apply rather than this statement.

10.2. Appendix 2: Clinical Laboratory Tests

Table 7: Full Laboratory Tests

**Hematology**	**Serum Chemistry, Including Liver Function**		
Hemoglobin<br>Hematocrit<br>Red blood cell indices<br>Thrombocyte count (platelets)<br>WBC count (absolute and %) with differential	Sodium<br>Potassium<br>Chloride<br>Bicarbonate<br>Phosphate<br>Calcium<br>Glucose<br>Amylase<br>Lipase<br>Uric acid<br>Magnesium	Creatine kinase<br>Creatinine<br>eGFR calculated using CKD-EPI formula<br>Blood urea nitrogen<br>Alanine transaminase<br>Alkaline phosphatase<br>Aspartate transaminase<br>Gamma-glutamyl transferase<br>Total bilirubin<br>Conjugated bilirubin<br>Unconjugated bilirubin	Total cholesterol<br>High-density lipoprotein<br>Low-density lipoprotein<br>Triglycerides (serum)<br>Free fatty acid<br>Calcitonin<br>Albumin<br>Globulins<br>Total protein<br>Lactate dehydrogenase<br>Thyroid stimulating hormone (at Screening Visit only)<br>Hemoglobin A1c<br>High-sensitivity C-reactive protein
**Coagulation**			
Prothrombin time<br>INR<br>aPTT<br>Fibrinogen			
**Virology**	**Drug Screen (Urine)**		**Pregnancy/Postmenopausal Status**
HIV-1/-2 antibody<br>HbsAg<br>Hepatitis C virus antibody and viral load<br>COVID-19 (SARS-CoV-2 antigen and viral load)[a]<br>Norovirus, influenza[c]	Opiates<br>Cocaine<br>Benzodiazepines	Barbiturates<br>Methadone<br>Amphetamines	FSH for postmenopausal women<br>Serum hCG [b]<br>Urine hCG [b]

Abbreviations: aPTT=activated partial prothrombin time; CKD-EPI=Chronic Kidney Disease Epidemiology Collaboration; COVID-19=coronavirus disease 2019; eGFR=estimated glomerular filtration rate; FSH=follicle-stimulating hormone; HbsAg=hepatitis B surface antigen; hCG=human chorionic gonadotropin; HIV=human immunodeficiency virus; INR=international normalized ratio; PCR=polymerase chain reaction; SARS-CoV-2=severe acute respiratory syndrome coronavirus 2; WBC=white blood cell.

a. COVID-19 test performed only if clinically indicated as determined by the Investigator; SARS-CoV-2 antigen test is performed first, and then PCR test for confirmation if clinically indicated.
b. If the urine hCG is positive or inconclusive, pregnancy will be confirmed by a serum hCG test.
c. Norovirus and/or influenza testing may be performed if indicated by the Investigator.

If an abnormal laboratory test result is received from a local laboratory during an emergency, the local laboratory result should also be collected and recorded in the EDC system.

10.3. Appendix 3: Adverse Events and Serious Adverse Events: Definitions and Procedures for Recording, Evaluating, Follow-up, and Reporting

10.3.1. Definition of Adverse Event

An AE is any untoward medical occurrence in a clinical study participant that is administered a pharmaceutical product and does not necessarily have to have a causal relationship with this treatment. An AE can therefore be any unfavorable and unintended sign (including an abnormal laboratory finding, for example), symptom, or disease temporally associated with the use of a

medicinal (investigational) product, whether or not considered related to the medicinal (investigational) product (definition per ICH).

Any AE for which a causal relationship with the IP is at least a reasonable possibility (possibly or probably related) is referred to as an adverse drug reaction.

The following are considered normal for the class of GLP-1RA drugs and thus should not be considered AEs:

- Loss of appetite
- Early satiety
- Weight loss

10.3.2. Definition of Serious Adverse Event

An SAE is any AE that, at any dose, meets at least 1 of the following:

- Results in death
- Is life-threatening
- Note: The term 'life-threatening' in the definition of 'serious' refers to an event in which the participant was at risk of death at the time of the event; it does not refer to an event that hypothetically might have caused death, if it were more severe.
- Requires inpatient hospitalization or prolongation of an existing hospitalization,
- Note: Only hospitalizations that are longer than expected based on Investigator judgment will be considered prolonged hospitalizations.
- Results in persistent or significant disability/incapacity
- Note: Results in a substantial disruption of a person's ability to conduct normal life functions.
- Is a congenital anomaly/birth defect (that occurs in the offspring of a participant exposed to the IP)
- Other important medical event

Medical and scientific judgment should be exercised in deciding whether other situations should be considered serious, such as important medical events that may not be immediately life--threatening or result in death or hospitalization but may jeopardize the participant or may require medical or surgical intervention to prevent one of the other outcomes listed in the above definition. These should also be considered serious.

Examples of such events are intensive treatment in an emergency room or at home for allergic bronchospasm, blood dyscrasias or convulsions that do not result in hospitalization, or development of drug dependency or drug abuse.

10.3.3. Definition of Suspected Unexpected Serious Adverse Reaction

A suspected unexpected serious adverse reaction (SUSAR) is an AE that meets all the following criteria:

- Is serious
- There is at least a reasonable possibility of a causal relationship between the event and the IP
- Is considered unexpected (i.e., the event is not listed in the Reference Safety Information section of the Investigator's Brochure or is not listed at the specificity or severity that has been observed)
- Note: "Unexpected" as used in this definition also refers to AEs that are mentioned in the Investigator's Brochure as occurring with a class of drugs or are anticipated from the pharmacological properties of the drug but are not specifically mentioned as occurring with the particular drug under investigation.

10.3.3.1. Assessment of Intensity

The Investigator will assess the severity of each AE and SAE reported during the study. The assessment will be based on the Investigator's clinical judgment. The severity of each AE and SAE will be graded using the most current version of the NCI-CTCAE (Version 5) 5-point scale (Table 8).

Table 8: NCI-CTCAE 5-Point Scale for Grading the Intensity of Each AE and SAE

Grade		Definition
I	Mild	Asymptomatic or mild symptoms: Clinical or diagnostic observations only; intervention not indicated
II	Moderate	Minimal, local or noninvasive intervention indicated; limiting age-appropriate- instrumental activities of daily living
III	Severe	Severe or medically significant but not immediately life-threatening: Hospitalization or prolongation of hospitalization indicated; disabling; limiting self-care activities of daily living
IV	Life-threatening[a]	Life-threatening consequences, urgent intervention indicated
V	Death[a]	Death related to AE

Abbreviations: AE=adverse event; NCI-CTCAE=National Cancer Institute Common Terminology Criteria for Adverse Events; SAE=serious adverse event.
a. Applies only to SAEs.

If an AE has multiple aspects (symptoms), the aspect with the highest severity will be graded.

The term severe is a measure of severity. Thus, a severe AE is not necessarily serious. For example, itching for several days may be rated as severe but may not be clinically serious.

10.3.3.2. Assessment of Causality

The Investigator will make an assessment as to the relationship between the study drug and the occurrence of each AE/SAE. The Investigator will use clinical judgment to determine whether or not the AE/SAE is causally related to the study treatment(s). Alternative causes, such as natural history of an underlying medical condition, concomitant therapy, other risk factors, and the temporal relationship of the event to the study drug, will be considered and investigated. The Investigator will also consult the Investigator's Brochure (or the product information sheet in instances where the study drug is an approved agent) in the determination of his/her assessment.

The causal relationship of the study drug to an AE will be rated according to the scale in Table 9.

Table 9: Relationship of the Study Treatment to an AE (Definitions)

Causal Relationship	Definition
Not related	Another cause of the event is most plausible; or clinically plausible temporal sequence is inconsistent with the onset of the event and the trial treatment administration; or a causal relationship is considered biologically implausible.
Unlikely related	An event or laboratory test abnormality, with a time to drug intake that makes a relationship improbable (but not impossible); disease or other drugs provide plausible explanations.
Possibly	An event that follows a reasonable temporal sequence from administration of the trial treatment or a known or expected response pattern to the suspected drug, but that could readily have been produced by a number of other factors.
Probably	An event that follows a reasonable temporal sequence from administration of the trial treatment, and there is a biologically plausible mechanism for trial treatment causing or contributing to the AE, and the event could not be reasonably explained by the known characteristics of the participant’s clinical state. In addition, the relationship may be confirmed by improvement on stopping the trial treatment and reappearance of the event on repeated exposure.
Related	An event cannot be reasonably explained by an alternative explanation, e.g., concomitant drug(s), concomitant disease(s). The relationship in time to study drug administration is very suggestive.

Abbreviations: AE=adverse event.

The Investigator will follow all related AEs observed during the trial until the AEs are considered resolved, until 30 days after the last dose of aleniglipron, or until completion of the follow-up visit, whichever is later. The Investigator will follow all SAEs, whether assessed as possibly related, probably related, or not related to study treatment, until the SAEs are considered stable, resolved, or until EOS (whichever is earlier). The Sponsor may request additional follow-up on specific unresolved AEs.

10.3.4. Action Taken and Outcome

For all AEs reported, the actions taken and outcomes must be specified and documented in the eCRF. Actions taken with the study drug may include any of the following:

- Dose not changed
- Dose reduced
- Drug withdrawn
- Not applicable
- Unknown
- Drug interrupted

Other action(s) taken:

- No action

- Medication required
- Termination of a concomitant medication
- Change of the dose of a concomitant medication
- Hospitalization or prolongation of hospitalization (please complete an SAE Form)
- Initiation/termination of a nondrug therapy
- Other (please specify)

The following terms and definitions are used in assessing the final outcome of an AE (Table 10).

Table 10: Terms and Definitions Used in Assessing the Final Outcome of an AE

Term	Definition
Recovered	The participant has fully recovered, or by medical or surgical treatment, the condition has returned to the level observed at the first trial-related activity after the participant signed the informed consent.
Recovering	The condition is improving and the participant is expected to recover from the event.
Recovered with sequelae	The participant has recovered from the condition but with lasting effect due to a disease, injury, treatment, or procedure. If a sequela meets an SAE criterion, the AE must be reported as an SAE.
Not recovered	The condition of the participant has not improved, and the symptoms are unchanged, or the outcome is not known at the time of reporting.
Fatal	This term is only applicable if the participant died from a condition related to the reported AE. Outcomes of other reported AEs in a participant before he/she died should be assessed as "recovered," "recovering," "recovered with sequelae," or "not recovered." An AE with a fatal outcome must be reported as an SAE.
Unknown	This term is only applicable if the participant is lost to follow-up.

Abbreviations: AE=adverse event; SAE=serious adverse event.

10.3.5. Recording Adverse Events and/or Serious Adverse Events

All AEs, regardless of seriousness, severity, or presumed relationship to study treatment, must be collected from time of signing the ICF and through 30 days after date of the last dose and recorded using medical terminology in the eCRF and/or other sources. Whenever possible, diagnoses should be given when signs and symptoms are due to a common etiology (e.g., cough, runny nose, sneezing, sore throat, and head congestion may be reported as "upper respiratory infection"). Investigators must record in the eCRF the date of onset of the event, their opinion concerning the relationship of the AE to the study drug, severity of the event, whether the event is serious or nonserious, actions taken to manage the event, the outcome of the event, and date of resolution where applicable.

This study will utilize electronic patient-reported outcome/e-diary technology. Any specific symptoms should be captured in the e-diary by the participant and assessed by the site staff for reporting as AEs.

Any increase to the severity of an AE must be recorded as a separate AE, ensuring that the end date and time of the preceding AE matches the start date and time of the subsequent AE so that the overall duration of the AE is continuous.

10.3.5.1. Follow-up of Adverse Events and Serious Adverse Events

The Investigator will ensure that AE/SAE follow-up includes any supplemental investigations as may be indicated to elucidate the nature and/or causality of the event. This may include additional laboratory tests or investigations, histopathological examinations, or consultation with other health care professionals. The Sponsor may also request that the Investigator perform or arrange for the conduct of supplemental measurements and/or evaluations.

If a participant dies during participation in the study or during a recognized follow-up period, the Sponsor will be provided with a copy of any postmortem findings, including histopathology.

New or updated information will be reported as required by Section 10.3.6. Due to COVID-19, Investigators must also ensure compliance with local governing legislation and reporting requirements associated with COVID-19 infections.

10.3.6. Reporting of Serious Adverse Events, Pregnancy, and Suspected Unexpected Serious Adverse Reactions

The Investigator must notify the study Sponsor and/or its designee within 24 hours of becoming aware of the occurrence of an SAE or pregnancy. If any new clinical information relating to an existing SAE or pregnancy becomes available, this information must also be reported within 24 hours.

The notification must be directed to the following:<br>Email: [redacted]

Information on SAE or pregnancy will be recorded on the SAE or Pregnancy Form, respectively. All SAE information should be entered into the eCRF. If the eCRF is not available, and for pregnancies, a paper SAE or Pregnancy Form should be completed and sent to Parexel Safety Services via email. Blank copies are included in the study Investigator's file. It is not acceptable for the Investigator to send photocopies of the participant's medical records to the Sponsor company or its representative in lieu of completion of the appropriate AE eCRF page or SAE Form. However, there may be instances when copies of medical records for certain cases are requested by the Sponsor or its representative. In this instance, all participant identifiers will be blinded on the copies of the medical records before submission to the Sponsor or its representative.

The completed SAE/Pregnancy Form and cover sheet should be sent via email immediately upon completion to structuretx_safety@parexel.com. If the Investigator does not have all information regarding an SAE/pregnancy, they will not wait to receive additional information before completing and sending the form.

Additional relevant information or clinical follow-up should be sent via email to structuretx_safety@parexel.com as soon as it becomes available. The Investigator should follow the participant with the event until resolution or stabilization of the condition. Follow-up reports (as many as required) should be completed and emailed following the same procedure above.

A final report is required once the condition is resolved or stabilized and no more information about the event is expected. The final report should be completed and emailed following the same procedure above.

The Investigator must keep a copy of all documentation related to the event in the clinical site files.

If the Investigator learns of any SAE, including death, at any other time after a participant completes the study, and he/she considers the event reasonably related to the study drug, the Investigator will promptly notify the Sponsor/MM.

It is the responsibility of the Sponsor to assess the expectedness of an SAE in order to determine whether a reported SAE fits the classification of a SUSAR. The Investigator's opinion regarding causality will be considered in the Sponsor's determination of the SAE as a SUSAR. The causality assessment given by the Investigator cannot be downgraded by the Sponsor. Both the Sponsor's and Investigator's causality assessment will be considered to determine expedited reportability of an SAE as a SUSAR.

The Investigator must also report SAEs to the IRB / EC that approved the protocol in accordance with the IRB / EC's safety reporting procedures.

The Sponsor assumes responsibility for appropriate reporting of SAEs (including SUSARs) to the regulatory authorities, in accordance with applicable regulatory requirements.

10.4. Appendix 4: Contraceptive and Barrier Guidance

WOCBP must agree not to donate ova, not to attempt to become pregnant, and, if engaging in sexual intercourse with a male partner, must agree to use a highly effective method of contraception from signing the consent form until ≥ 30 days after the last dose of study drug.

Male participants must agree not to donate sperm and, if engaging in sexual intercourse with a female partner who could become pregnant, must agree to use a highly effective method of contraception from signing the consent form until ≥ 90 days after the last dose of study drug.

A highly effective method of contraception for WOCBP is 1 of the following:

- Have a biologically sterile or surgically sterile partner (i.e., a female participant's male partner has undergone effective surgical sterilization such as vasectomy) before the female participant entered the clinical trial and has obtained documentation of absence of sperm in his ejaculate, and he is the sole sexual partner of the female participant during the clinical trial
- Use of a combination of a condom by the female participant's partner and one of the following effective methods of contraception:
  - Oral or intravaginal combined (estrogen- and progestogen-containing) hormonal contraceptives
  - Oral, injectable, or implantable progestogen-only hormonal contraceptives associated with inhibition of ovulation
  - Intrauterine device or intrauterine hormone-releasing system

A highly effective method of contraception for **male participants** is 1 of the following:

- Biologically sterile or surgically sterile (i.e., the male participant has undergone effective surgical sterilization such as vasectomy) before entering the clinical trial and has obtained documentation of absence of sperm in his ejaculate
- Use of a combination of a condom in addition to having a female partner of childbearing potential and use 1 the following effective methods of contraception:
  - Oral or intravaginal combined (estrogen- and progestogen-containing) hormonal contraceptives
  - Oral, injectable, or implantable progestogen-only hormonal contraceptives associated with inhibition of ovulation
  - Intrauterine device or intrauterine hormone-releasing system
  - Bilateral tubal ligation/occlusion

People with same sex partners are not required to use highly effective methods of contraception. However, abstinence is not an acceptable form of contraception for heterosexual partners.

10.5. Appendix 5: Clinical Inhibitors and Inducers

Aleniglipron is a P-gp and CYP3A4 substrate. Because aleniglipron is a Biopharmaceutics Classification System (BCS) Class 4 compound (poor solubility, low permeability) and a Biopharmaceutical Drug Disposition Classification System (BDDCS) Class 4 compound (where absorptive and efflux transporters could be important), changes in transporters (i.e., P-gp) will have the largest effect on variability in aleniglipron exposure. Therefore, it is much more likely that P-gp inhibition could increase the exposure to aleniglipron and that P-gp inducers could decrease the exposure to aleniglipron. It is also likely that aleniglipron is not a sensitive CYP3A4 substrate.

This study will exclude participants who are taking concomitant medications that are sensitive P-gp substrates (e.g., dabigatran etexilate, digoxin, fexofenadine, everolimus, sirolimus, or venetoclax) or sensitive BCRP substrates (e.g., ozanimod, sulfasalazine).

Because aleniglipron is only a weak CYP3A4 inhibitor, concomitant medications that are CYP3A4 substrates do not need to be excluded from this study; however, concomitant medications that are sensitive CYP3A4 substrates may require monitoring with or without dose reductions, according to the individual respective product labeling as summarized in (Table 12). As lovastatin and simvastatin are sensitive CYP3A4 substrates, concomitant use of these medications is not allowed. Participants taking lovastatin or simvastatin should be switched to rosuvastatin at least 2 days prior to randomization. Participants on other statin drugs, including atorvastatin or rosuvastatin, are allowed to be enrolled.

Therefore, the following exclusion criteria (Table 11) will be applied to the Phase 2b study:

Table 11: Excluded Medications Based on Concomitant Use of P-gp and CYP3A4 Substrates, Inhibitors, and Inducers

Class and Potential Effect	List of Excluded Medications			
Sensitive P-gp substrates	dabigatran etexilate<br>digoxin	everolimus	fexofenadine<br>sirolimus	venetoclax
Sensitive BCRP substrates	ozanimod	sulfasalazine		
Sensitive CYP3A4 substrates[a]	lovastatin	simvastatin		
P-gp inhibitors<br>(potential to increase exposure to aleniglipron)	amiodarone<br>azithromycin<br>capmatinib<br>carvedilol<br>clarithromycin<br>curcumin<br>cyclosporine<br>digoxin<br>diosmin<br>dronedarone<br>erythromycin	fexofenadine<br>flibanserin<br>fluvoxamine<br>glecaprevir<br>indinavir<br>itraconazole<br>ketoconazole<br>lapatinib<br>lopinavir<br>nirmatrelvir<br>osimertinib	pibrentasvir<br>piperine<br>quercetin<br>quinidine<br>ranolazine<br>rifampin<br>ritonavir<br>saquinavir<br>Schisandra chinensis<br>sofosbuvir	telaprevir<br>tepotinib<br>tipranavir<br>valspodar<br>velpatasvir<br>vemurafenib<br>verapamil<br>voxilaprevir
P-gp inducers<br>(potential to decrease exposure to aleniglipron)	apalutamide<br>avasimibe<br>carbamazepine	curcumin<br>dan-shen [Salvia miltiorrhiza]	genistein<br>green tea<br>lorlatinib	rifampin<br>St. John's wort<br>tivantinib
Strong CYP3A4 inhibitors<br>(potential to increase exposure to aleniglipron)	adagrasib<br>boceprevir<br>ceritinib<br>clarithromycin<br>cobicistat<br>danoprevir<br>dasabuvir<br>elvitegravir<br>ensartinib	ensitrelvir<br>idelalisib<br>indinavir<br>itraconazole<br>josamycin<br>ketoconazole<br>lonafarnib<br>lopinavir<br>mibefradil	mifepristone<br>nefazodone<br>nelfinavir<br>nirmatrelvir<br>ombitasvir<br>paritaprevir<br>posaconazole<br>relacorilant<br>ribociclib	ritonavir<br>saquinavir<br>telaprevir<br>telithromycin<br>tipranavir<br>troleandomycin<br>tucatinib<br>voriconazole
Moderate CYP3A4 inhibitors at study start[b]<br>(have the potential to increase exposure to aleniglipron)	amprenavir<br>aprepitant<br>atazanavir<br>berotralstat<br>casopitant<br>ciprofloxacin	diltiazem<br>dronedarone<br>duvelisib<br>erythromycin<br>faldaprevir<br>fedratinib	ipatasertib<br>isavuconazole<br>lefamulin<br>lenacapavir<br>letermovir<br>netupitant	ritlecitinib<br>Schisandra sphenanthera<br>tofisopam<br>treosulfan<br>verapamil

Class and Potential Effect	List of Excluded Medications			
	clofazimine<br>conivaptan<br>crizotinib	fluconazole<br>grapefruit juice<br>imatinib	nilotinib<br>nivasorexant<br>ravuconazole	voxelotor<br>ziritaxestat
Strong CYP3A4 inducers<br>(potential to decrease exposure to aleniglipron)	apalutamide<br>avasimibe<br>carbamazepine	enzalutamide<br>ivacaftor<br>ivosidenib	lumacaftor<br>mitotane<br>phenytoin	rifampin<br>rifapentine<br>St. John's wort

Abbreviations: BCRP=breast cancer resistance protein; CYP=cytochrome P450 enzyme; P-gp=P-glycoprotein.
[a] As lovastatin and simvastatin are sensitive CYP3A4 substrates, concomitant use of these medications is not allowed. Participants taking lovastatin or simvastatin should be switched to rosuvastatin at least 2 days prior to randomization. Participants on other statin drugs, including atorvastatin or rosuvastatin, are allowed to be enrolled.
[b] If acute treatment with a moderate inhibitor is required post-study start and there is no alternative treatment, the Investigator will contact the Sponsor for assessment based on the aleniglipron doses being administered (coadministration at low doses could be feasible). Chronic concomitant treatment with moderate CYP3A4 inhibitors would not be allowed post-study start.

Because aleniglipron is only a weak CYP3A4 inhibitor, concomitant medications that are sensitive CYP3A4 substrates may require monitoring with or without dose reductions, according to the individual respective product labeling, as summarized below.

Table 12: CYP3A4 Inhibition: Recommendation from the Product Label of Concomitant Medications that are Sensitive CYP3A4 Substrates

Concomitant Medication	Instructions for Use with Strong or Moderate CYP3A4 Inhibitors	Instructions for Use with Weak CYP3A4 Inhibitors
Drugs that have specific recommendations when concomitantly used with a weak CYP3A inhibitor		
Darifenacin	The systemic exposure of darifenacin from Enablex extended-release tablets is increased in the presence of CYP3A4 inhibitors. The daily dose of Enablex should not exceed 7.5 mg when coadministered with strong CYP3A4 inhibitors (for example, ketoconazole, itraconazole, ritonavir, nelfinavir, clarithromycin and nefazadone).	No dosing adjustments are recommended in the presence of mild or moderate CYP3A4 inhibitors.
Lemborexant	Strong or moderate CYP3A inhibitors: Avoid concomitant use.	The maximum recommended dose is 5 mg.
Lomitapide	Strong and moderate CYP3A4 inhibitors are contraindicated with JUXTAPID. Participants must avoid grapefruit juice.	Do not exceed 30 mg daily of JUXTAPID when used concomitantly with weak CYP3A4 inhibitors.
Naloxegol	Concomitant use with strong CYP3A4 inhibitors is contraindicated.<br>Moderate CYP3A4 inhibitors: Increased naloxegol concentrations; avoid concomitant use; if unavoidable, reduce dosage to 12.5 mg QD and monitor for adverse reactions.	Clinically significant increases in naloxegol concentrations are not expected. No dosage adjustments are necessary.
Drugs that do not distinguish between strong, moderate, or weak CYP3A4 inhibitors and recommend monitoring with or without dose reductions		

Concomitant Medication	Instructions for Use with Strong or Moderate CYP3A4 Inhibitors	Instructions for Use with Weak CYP3A4 Inhibitors
Alfentanil	Monitor participants closely at frequent intervals and consider dosage reduction of alfentanil HCl injection.	
Budesonide	If treatment with inhibitors of CYP3A4 activity (such as ketoconazole, itraconazole, ritonavir, indinavir, saquinavir, erythromycin, etc.) is indicated, reduction of the budesonide dose should be considered.	
Buspirone	Subsequent dose adjustment may be necessary and should be based on clinical assessment.	
Darunavir	Administration of darunavir and ritonavir and other drugs that inhibit CYP3A may decrease the clearance of darunavir and ritonavir and may result in increased plasma concentrations of darunavir and ritonavir.	
Felodipine	Caution should be used when CYP3A4 inhibitors are coadministered with felodipine. A conservative approach to dosing felodipine should be taken.	
Indinavir	Coadministration of CRIXIVAN and other drugs that inhibit CYP3A4 may decrease the clearance of indinavir and may result in increased plasma concentrations of indinavir. Dose reductions should be considered.	
Maraviroc	Reduce dose to 150 mg twice daily in participants receiving CYP3A4 inhibitors.	
Midazolam	Caution is advised when midazolam injection is administered concomitantly with drugs that are known to inhibit the P450-3A4 enzyme. These drug interactions may result in prolonged sedation caused by a decrease in plasma clearance of midazolam.	
Nisoldipine	Known inducer or inhibitor of CYP3A4 should be avoided in general.	
Tacrolimus	Since tacrolimus is metabolized mainly by CYP3A enzymes, drugs or substances known to inhibit these enzymes may increase tacrolimus whole blood concentrations. Monitoring of whole blood concentrations and appropriate dosage adjustments of tacrolimus are recommended.	
Drugs that have no recommendations for concomitant use of a weak CYP3A4 inhibitor (exercise caution, monitor and reduce dose as needed)		
Avanafil	Do not use STENDRA in participants taking strong CYP3A4 inhibitors.	None
Conivaptan	The coadministration of VAPRISOL with strong CYP3A inhibitors, such as ketoconazole, itraconazole, clarithromycin, ritonavir, and indinavir, is contraindicated.	None
Dasatinib	The coadministration with strong CYP3A inhibitors may increase dasatinib concentrations. Increased dasatinib concentrations may increase the risk of toxicity. Avoid concomitant use of strong CYP3A4 inhibitors. If concomitant administration of a strong CYP3A4 inhibitor cannot be avoided, consider a SPRYCEL dose reduction.	None
Dronedarone	Concomitant use of a strong CYP3A inhibitor are contraindicated.	None
Eletriptan	Strong CYP3A4 inhibitors significantly increase the exposure of RELPAX. Relpax should not be	None

Concomitant Medication	Instructions for Use with Strong or Moderate CYP3A4 Inhibitors	Instructions for Use with Weak CYP3A4 Inhibitors
	used within at least 72 hours of treatment with strong CYP3A4 inhibitors.	
Eplerenone	Because eplerenone metabolism is predominantly mediated via CYP3A4, do not use INSPRA with drugs that are strong inhibitors of CYP3A4. Reduce the starting dose for hypertension to 25 mg QD when used with moderate CYP3A4 inhibitors (e.g., verapamil, erythromycin, saquinavir, fluconazole).	None
Ibrutinib	Avoid concomitant use of other strong CYP3A inhibitors.<br>Dose modifications of IMBRUVICA are recommended when used concomitantly with moderate CYP3A inhibitors.	None
Isavuconazonium sulfate (isavuconazole)	Contraindicate coadministration of all strong CYP3A4 inhibitors.	None
Ivabradine	The concomitant use of strong CYP3A4 inhibitors is contraindicated. Avoid concomitant use of moderate CYP3A4 inhibitors when using Corlanor.	None
Lurasidone	Strong CYP3A4 inhibitors: Recommended dosage is 10.5 mg QD.<br>Moderate CYP3A4 inhibitors: Recommended dosage is 21 mg QD.	None
Mobocertinib	Avoid concomitant use of EXKIVITY with strong or moderate CYP3A inhibitors. If concomitant use of a moderate CYP3A inhibitor is unavoidable, reduce the dose of EXKIVITY.	None
Quetiapine	The dose of SEROQUEL should be reduced to one-sixth of the original dose if coadministered with a strong CYP3A4 inhibitor.	None
Sildenafil	A starting dose of 25 mg of VIAGRA should be considered in participants taking erythromycin or strong CYP3A4 inhibitors.	None
Ticagrelor	Strong CYP3A inhibitors substantially increase ticagrelor exposure and thus increase the risk of dyspnea, bleeding, and other adverse events. Avoid use of strong inhibitors of CYP3A.	None
Tolvaptan	Concomitant use of tolvaptan with strong CYP3A inhibitors is contraindicated. Avoid coadministration of SAMSCA with moderate CYP3A inhibitors.	None
Triazolam	Strong inhibitors of CYP 3A that should not be used concomitantly with triazolam.	None

Concomitant Medication	Instructions for Use with Strong or Moderate CYP3A4 Inhibitors	Instructions for Use with Weak CYP3A4 Inhibitors
Vardenafil	A single 2.5 mg dose of Levitra should not be exceeded in a 24-hour period when used with strong CYP3A4 inhibitors. A single 5 mg dose should not be exceeded in a 24 hour period when used with moderate CYP3A4 inhibitors	None

Abbreviations: CYP = cytochrome P450 enzyme; HCl = hydrochloride; QD=once daily.

10.6. Appendix 6: Evaluation of Potential Drug-Induced Liver Injury

Definition

- Hepatocellular injury (usually detected by serum ALT/AST elevations) can be caused by drugs that rarely, if ever, cause severe DILI.
- Hy's Law is an ominous indicator of severe DILI, which is defined as follows:
  - ALT/ AST > 3× ULN
  - TBL >2× ULN
  - Without initial findings of cholestasis (elevated serum ALP)
  - No other reason can be found to explain the combination of increased ALT/AST and TBL

Detection

Any laboratory testing (specific in protocol or ad hoc/additional testing). If any ad hoc testing is carried out, the information is to be added to the case report forms and database. If any other information (clinical signs/symptoms and/or laboratory data) indicates potential liver injury, liver enzyme (ALT/AST) and TBL should be obtained.

Confirmation

Any ALT/AST elevation to >3× ULN should be followed by repeat testing as soon as possible but not later than 72 hours for all usual serum measures (ALT, AST, ALP, and TBL) to confirm the abnormalities and to monitor the changes.

Reporting

Any potential DILI case (i.e., even before all other possible causes of liver injury have been excluded) should be reported to the Sponsor as an SAE within 24 hours of awareness. Reporting should include all available information, especially that needed for evaluating the severity and likelihood that the drug caused the reaction, and should initiate a close follow-up until complete resolution of the problem and completion of all attempts to obtain supplementary data.

Close observation

All potential DILI events should be closely observed, which include the following:

- Repeating liver enzyme and serum bilirubin tests 2 or 3 times weekly. The frequency of retesting can decrease to once a week or less if abnormalities stabilize or the trial drug has been discontinued and the participant is asymptomatic.
- Obtaining a more detailed history of symptoms and prior or concurrent diseases.
- Obtaining a history of concomitant drug use (including nonprescription medications and herbal and dietary supplement preparations), recreational drug use, and special diets.
- Ruling out acute viral hepatitis types A, B, C, D, and E; autoimmune or alcoholic hepatitis; MASH; hypoxic/ischemic hepatopathy; and biliary tract disease.
- Obtaining a history of exposure to environmental chemical agents.
- Obtaining additional tests to evaluate liver function, as appropriate (e.g., INR, direct bilirubin).
- Considering gastroenterology or hepatology consultations.

<u>Actions</u>

The study drug should be interrupted or discontinued if 1 or more of these conditions occur:

- ALT or AST >8× ULN
- ALT or AST >5× ULN for more than 2 weeks
- ALT or AST >3× ULN and serum TBL > 2× ULN or INR > 1.5 × ULN without elevated serum ALP, and no other disease or condition can be found to explain the abnormalities
- ALT or AST >3× ULN with the appearance of fatigue, nausea, vomiting, right upper quadrant pain or tenderness, fever, rash, and/or eosinophilia (>5%)

<u>Evaluation for alternative causes</u>

The SMR and Investigator should work closely together to evaluate potential alternative causes, including, but not limited to, the following:

- Acute viral hepatitis
- Alcoholic and autoimmune hepatitis
- Hepatobiliary disorders
- MASH
- Cardiovascular causes
- Concomitant treatments

<u>Follow-up to resolution</u>

All participants with potential DILI should be followed until all abnormalities return to normal or to the baseline state.

Rechallenge

For any study drug interruption or discontinuation due to potential DILI, rechallenge decision should be made jointly by Investigator and Sponsor MM and based on the following:

- For confirmed Hy's Law case, rechallenge should not be attempted.
- For potential Hy's Law case with manageable alternative causes and moderate liver enzyme elevations (ALT/AST = 5× ULN), rechallenge should not be attempted unless the alternative causes are well controlled, liver function tests are back to normal or baseline, and agreed upon by both SMR and Investigator.
- For moderate ALT/AST (between 3× and 5× ULN) elevation without TBL elevation, the study drug might be restarted if liver function tests are back to normal or baseline and agreed upon by both SMR and Investigator.

Reference

Guidance for Industry DILI: Premarketing Clinical Evaluation by FDA

11. REFERENCES

APA, 2013. *Diagnostic and Statistical Manual fof Mental Disorders (DSM-5).* Washington DC: American Psychiatric Asssociation.

Apovian, C. et al., 2015. Pharmacological management of obesity: an endocrine society clinical practice guideline. *J Clin Endocrinol Metab*, pp. 100(2):342-362.

Benet, L., 2013. The role of BCS (Biopharmaceutics Classification System) and BDDCS (Biopharmaceutics Drug Disposition Classification System) in drug development. *J Pharm Sci,* pp. 102(1):34-42.

Davies, M. et al., 2022. Managment of hyperglycemia in type 2 diabetes, 2022. A consensus report by the American Diabetes Association (ADA) and the European Association for the Study of Diabetes (EASD). *Diabetes Care*, pp. 45(11):2753-2786.

Garvey, W. et al., 2016. American association of clinical endocrinologists and American college of endocrinology comprehensive clinical practice guidelines for medical care of patients with obesity. *Endoc Pract*, pp. 22 Suppl 3:1-203.

Ghoorah, K. et al., 2016. Obesity and cardiovascular outcomes: a review. *Eur Heart J Acute Cardiovasc Care,* pp. 5(1):77-85.

Gorgojo-Martinez, J. et al., 2023. Clinical recommendations to manage gastrointestinal adverse events in patients treated with Glp-1 receptor agonists: a multidisciplinary expert consensus. *Journal of Clinical Medicine*, pp. 12, 145.

Jastreboff, A. et al., 2019. Obesity as a disease: the obesity society 2018 position statement. *Obesity*, pp. 27:7-9.

Kolotkin, R., Williams, V., Ervin, C. & al, e., 2019. Validation of a new measure of quality of life in obesity trials: impact of weight on quality of life-lite clinical trials version. *Clin Obes,* 9(3), p. e12310.

Kroenke, K., Spitzer, R. & Williams, J., 2001. The PHQ-9: validity of a brief depression severity measure. *Journal of GEneral INternal Medicine,* 16(9), pp. 606-613.

Lin, P. et al., 2003. DASH Collaborative Research Group. Estimation of energy requirements in a controlled feeding trial.. *Am J Clin Nutr,* pp. 77(3):639-45.

Maruish, M., 2011. *User's manual for the SF-36v2 Health Survey.* 3rd ed. Lincoln, RI: Quality Metric Incorporated.

NIH, N. I. o. A. A. a. A., 2024. *Rethinking drinking. Alcohol and your health..* [Online]
Available at: https://www.rethinkingdrinking.niaaa.nih.gov/

NIH, N. I. o. D. a. D. a. K. D., 2021. *Overweight & obesity statistics.* [Online]
Available at: https://www.niddk.nih.gov/health-information/health-statistics/overweight-obesity

Powell-Wiley, T., Poirier, P., Burke, L. & al, e., 2021. Obesity and cardiovascular disease a scientific statement from the American Heart Association. *Circulation,* pp. 143:e984-e1010.

Project, T. C. L., 2016. *The Columbia Protocol for Research.* [Online]
Available at: https://cssrs.columbia.edu/the-columbia-scale-c-ssrs/cssrs-for-research/
[Accessed June 2024].

WHO, W. H. O., 2024. *Obesity and overweight.* [Online]
Available at: https://www.who.int/news-room/fact-sheets/detail/obesity-and-overweight

Signature Page for VV-CLIN-000055 v2.0

Approval Task (retain roles) Task<br>Verdict: Approved	[redacted]<br><br>02-Mar-2026 15:56:32 GMT+0000

Signature Page for VV-CLIN-000055 v2.0

Signature Page for VV-CLIN-000055 v2.0

Approval Task (retain roles) Task<br>Verdict: Approved	[redacted]<br>02-Mar-2026 15:56:32 GMT+0000

Signature Page for VV-CLIN-000055 v2.0

PROTOCOL
SUMMARY OF CHANGES

Protocol Version No: Amendment 6, Version 7	Date: 27 Feb 2026
**Supersedes: Amendment 5, Version 6**	**Date: 10 Nov 2025**

1. SUMMARY OF CHANGES

1.1. Substantial Changes

This protocol amendment contains the following changes which are considered substantial.

Table 1: Summary List of Substantial Changes

Protocol Section	Description of Change	Rationale
1.1 Synopsis, 1.2 Schema, 2.2 Study Rationale, 4.1 Overall Design, 4.3 Justification for Dose, 6.9 Continued Access to Study Intervention	Added 180 mg dose as the highest dose in the open label extension period of the study	180 mg dose of aleniglipron [redacted] and has been determined to be safe and efficacious. Adding to the open label extension to allow participants to receive the most benefit

1.2. Nonsubstantial Changes

This protocol amendment contains the following changes that are considered nonsubstantial (do not significantly impact the safety of participants, the scientific validity of the study, or the overall conduct and management of the study). Note that grammatical edits may occur throughout the document but are not included in this summary of changes.

Table 2: Summary List of Nonsubstantial Changes

Protocol Section	Description of Change	Rationale
2.1.3 Aleniglipron Clinical Overview	Revised the section to include current information on completed and ongoing clinical studies	To provide the most current information
2.3 Benefit/Risk Assessment	Revised the section to include current information	To provide the most current information
3 Objectives and Endpoints	Added “findings” to electrocardiogram	For clarity
4.1 Overall Design, 4.2 Scientific Rationale for Study Design	Added minor clarifications to identify the double-blind versus open label extension parts of the study	For clarity

Protocol Section	Description of Change	Rationale
4.4 End of Study Definition	Added clarification for participants who discontinue study early versus participants who discontinue study drug but remain in the study	For clarity
7.1 Discontinuation of Study Intervention	Revised the language for participants who discontinue study intervention but remain in the study	For clarity
7.1 Discontinuation of Study Intervention	Added to the possible reasons leading to permanent discontinuation of study drug	For clarity
7.2 Participant Discontinuation/Withdrawal From the Study	Revised the language	For clarity
10.6 Appendix 6	Added a section on Reporting any potential drug induced liver injury	For clarity

STATISTICAL ANALYSIS PLAN

DATE OF PLAN:

Version 1.0/ 05 November 2025

STUDY DRUG:

Aleniglipron (GSBR-1290)

PROTOCOL NUMBER:

GSBR-1290-06

STUDY TITLE:

A Phase 2b, Randomized, Double-blind, Placebo-controlled, Dose-range Finding Study of the Efficacy and Safety of Multiple Doses of Aleniglipron (GSBR-1290) in Participants Living with Obesity (Body Mass Index ≥ 30 kg/m^2) or Overweight (Body Mass Index ≥ 27 kg/m^2) with at Least One Weight-related Comorbidity

SPONSOR:

Gasherbrum Bio, Inc.

601 Gateway Blvd. Suite 900
South San Francisco, CA 94080

This study is being conducted in compliance with good clinical practice, including the archiving of essential documents.

SIGNATURE PAGE

[redacted]
Reason: I approve this document
Date: Nov 11, 2025 15:08:37 EST

[redacted] Date

[redacted]
Reason: I approve this document
Date: Nov 11, 2025 15:10:50 EST

[redacted] Date

[redacted] Electronically signed by: [redacted]
Reason: I approve this document
Date: Nov 11, 2025 13:43:08 PST

Date

[redacted]

[redacted] Electronically signed by: [redacted]
Reason: I approve this document
Date: Nov 11, 2025 14:29:14 PST

Date

[redacted]

[redacted] Electronically signed by: [redacted]
Reason: I approve this document
Date: Nov 11, 2025 17:43:12 PST

Date

[redacted]

[redacted] Electronically signed by: [redacted]
Reason: I approve this document
Date: Nov 11, 2025 17:57:50 PST

Date

[redacted]

VERSION HISTORY

Version	Version Date	Description
0.1	31 March 2025	Draft version
1.0	05 November 2025	Final version

TABLE OF CONTENTS

TITLE PAGE 1
VERSION HISTORY 4
LIST OF TABLES 7
LIST OF FIGURES 7
LIST OF ABBREVIATIONS 8
1. INTRODUCTION 10
2. STUDY OBJECTIVE(S) AND ENDPOINT(S) 11
2.1. Study Objective(s) 11
2.2. Estimands 12
2.2.1. Primary Estimand 12
2.2.2. Secondary Estimand 12
2.3. Study Design 13
2.3.1. Overview 13
2.3.2. Randomization and Blinding 15
2.3.3. Study Drug and Duration 15
2.3.4. Sample Size Considerations 16
3. STATISTICAL METHODOLOGY 17
3.1. General Considerations 17
3.1.1. Analysis Day 17
3.1.2. Definition of Baseline 17
3.1.3. Analysis Sets 17
3.1.4. Summary Statistics 18
3.1.5. Assessment Window 19
3.1.6. Hypothesis Testing 20
3.1.7. Handling of Dropouts and Missing Data 20
3.2. Participant Data and Study Conduct 21
3.2.1. Participant Disposition 21
3.2.2. Protocol Deviations 21
3.2.3. Analysis Populations 21
3.2.4. Demographic and Baseline Characteristics 22
3.2.5. Medical History 22

3.2.6. Prior and Concomitant Medications 23
3.2.7. Study Drug Exposure and Compliance 23
3.2.8. Dose Modification 24
3.3. Efficacy Assessment 25
3.3.1. Primary Efficacy Endpoint Analysis 25
3.3.1.1. Definition of Endpoint(s) 25
3.3.1.2. Analysis for the Primary Estimand 25
3.3.1.3. Analysis for the Secondary Estimand 26
3.3.2. Secondary Efficacy Endpoint(s) Analysis 26
3.3.2.1. Secondary Endpoints 26
3.3.2.2. Main Analytical Approach 26
3.3.2.3. Analyses for the Secondary Estimand 27
3.3.3. Exploratory Endpoints 27
3.3.3.1. Exploratory Efficacy Analysis 27
3.3.3.2. Pharmacokinetic and Pharmacokinetic/Pharmacodynamic Methods 27
3.3.3.3. Patient-Reported Outcomes 28
3.4. Safety Assessment 29
3.4.1. Adverse Events (AEs) 30
3.4.2. Vital Signs 32
3.4.3. Clinical Laboratory Data 33
3.4.4. Electrocardiograms 33
3.4.5. Physical Examination Data 34
3.5. Pharmacokinetic Assessment 34
3.5.1. Sample Collections for Pharmacokinetic Analysis 34
3.5.2. Handling Missing or Below the Lower Limit of Quantification Data 34
3.5.3. Pharmacokinetic Concentration 35
3.5.4. Pharmacokinetic Parameters 35
3.6. Pharmacodynamic Assessment 35
3.7. Other Analyses 35
3.7.1. Subgroup Analyses 35
3.7.2. Other Exploratory Analyses 36
3.7.3. Dose-Response Analysis 36
4. ANALYSIS TIMING 38

4.1. Interim Analysis 38
4.2. Final Analysis 38
5. CHANGES FROM PROTOCOL-SPECIFIED STATISTICAL ANALYSES 38
6. APPENDIX....................39
6.1. Appendix A: Schedule of Assessments 39
6.2. Appendix B: Searching Criteria for AESIs 42
6.3. Appendix C: Clinical Laboratory Tests 44
6.4. Appendix D: Pharmacokinetic Sampling Schedule....................45
6.5. Appendix E: Imputation Rules for Missing AE and Medication Dates 47
6.5.1. Imputation Rules for AE End Date and Medication End Date....................47
6.5.2. Imputation Rules for AE Start Date and Medication Start Date 47
7. REFERENCES 48

LIST OF TABLES

Table 1 Study Objectives and Endpoints....................11
Table 2 Study Intervention(s) Administered 15
Table 3 Definition of Analysis Sets....................17
Table 4 Assessment Window....................19
Table 5 Categorical Criteria for Abnormal Treatment-Emergent Blood Pressure and Pulse Measurements....................32

LIST OF FIGURES

Figure 1 Diagrammatic Representation of Study Design 14

LIST OF ABBREVIATIONS

Abbreviation	Definition
ADaM	Analysis Data Model
AE	Adverse event
ANCOVA	Analysis of covariate
ATC	Anatomical therapeutic chemical
BMI	Body mass index
BP	Blood pressure
CDISC	Clinical Data Interchange Standards Consortium
CIR	Copy Increments in Reference
eCRF	Electronic case report form
CSR	Clinical Study Report
CV	Coefficient of variation
DSMB	Data Safety Monitoring Board
ECG	Electrocardiogram
EOS	End of study
EOT	End of treatment
FDA	Food and Drug Administration
GM	Geometric mean
HbA1c	Hemoglobin A1c
HOV	Healthy overweight/obese volunteers
HR	Heart rate
ICH	International Council for Harmonisation of Technical Requirements for Pharmaceuticals for Human Use
MAR	Missing at random
MedDRA	Medical Dictionary for Regulatory Activities
MI	Multiple imputation
MMRM	Mixed model for repeated measurement
MMTT	Mixed meal tolerance test
MTD	Maximum tolerated dose
NCI-CTCAE	National Cancer Institute Common Terminology Criteria for Adverse Events
OLE	Open label extension
PCL	Protocol clarification letter
PD	Pharmacodynamics
PK	Pharmacokinetics
QD	Once daily
REML	Restricted maximum likelihood
SAE	Serious adverse event
SAP	Statistical Analysis Plan
SD	Standard deviation
SE	Standard error
SDTM	Study Data Tabulation Model
SOC	System organ class
T2DM	Type 2 diabetes mellitus
TEAE	Treatment-emergent adverse event
TESAE	Treatment-emergent serious adverse event
WHO	World Health Organization

WHO-DD	World Health Organization Drug Dictionary

1. INTRODUCTION

The purpose of this Statistical Analysis Plan (SAP) is to provide a description of the statistical methods to be implemented for the primary analysis of data from the Phase 2b clinical study GSBR-1290-06 (ACCESS) of aleniglipron (GSBR-1290) for the treatment of participants living with obesity or overweight and at least one weight-related comorbidity. If circumstances arise during the study such that more appropriate analytic procedures become available, the SAP may be revised. The statistical definitions and analytical methods described in this SAP supersede those in the protocol. The SAP will be finalized prior to unblinding and database lock/snapshot for final/interim analysis. Any revisions to the endpoint analyses will be made prior to the database freeze for the final analysis. Reasons for such revisions will be described in the final Clinical Study Report (CSR). The scope of analyses for this SAP does not include the open label extension.

2. STUDY OBJECTIVE(S) AND ENDPOINT(S)

2.1. Study Objective(s)

Table 1 Study Objectives and Endpoints

	Objectives	Endpoints
**Primary**	To compare the efficacy of increasing doses of aleniglipron against placebo on body weight change	• Percent change in body weight from Baseline to Week 36
**Secondary**	To compare the efficacy of increasing doses of aleniglipron against placebo on body weight change	• Percentage of participants who achieve ≥5% reduction in body weight at Week 36<br>• Percentage of participants who achieve ≥10% reduction in body weight at Week 36<br>• Percentage of participants who achieve ≥15% reduction in body weight at Week 36<br>• Change in body weight (absolute) from Baseline to Week 36<br>• Change in waist circumference from Baseline to Week 36<br>• Change in body mass index from Baseline to Week 36
	To assess the safety and tolerability profile of a monthly titration scheme	• TEAEs and SAEs<br>• AESI<br>• Laboratory parameters<br>• Electrocardiogram<br>• Vital signs
**Exploratory**	To assess the plasma pharmacokinetics of aleniglipron and other parameters	• Change in triglycerides, FFA, and hsCRP at Week 36<br>• Change in patient-reported outcomes (SF-36 acute version, IWQOL-Lite CT, and hunger/satiety evaluation) from Baseline to Week 36<br>• Change in HDL and LDL cholesterol from Baseline to Week 36<br>• Population pharmacokinetic parameters<br>• Change in resting blood pressure from Baseline to Week 36

	Objectives	Endpoints
		• Change in fasting glucose from Baseline to Week 36

Abbreviations: AESI=adverse event of special interest; FFA=free fatty acids; HDL=high-density lipoprotein; hsCRP=high-sensitivity C-reactive protein; IWQOL-Lite CT= Impact of Weight on Quality of Life-Lite Clinical Trials Version; LDL=low-density lipoprotein; PD=pharmacodynamic; SAEs=serious adverse events; SF-36=Short Form 36 health survey acute version; TEAEs=treatment-emergent adverse events.

2.2. Estimands

2.2.1. Primary Estimand

ICH E9 (R1) Addendum provides a general framework for defining estimands for clinical trials. The primary clinical interest of this study is to estimate the treatment effect of aleniglipron relative to placebo at Week 36 of treatment in participants who meet the inclusion criteria and would have completed the treatment period.

The "efficacy" estimand is described by the following attributes:

- Population: participants with obesity (body mass index [BMI] ≥30 kg/m^2) or overweight (BMI ≥27 kg/m^2) with at least one weight-related comorbidity who meet the protocol inclusion criteria (age 18-80, screening HbA1c <6.5%).
- Treatment: randomized treatment with titration to 1 of 3 target doses, with allowance of down- titration based on tolerability, or placebo.
- Endpoint: percent change in body weight from baseline to 36 weeks.
- Intercurrent event (ICE): permanent discontinuation of study drug, initiation of other anti-obesity medication, or bariatric surgery, whichever is earlier. The ICE is handled by the hypothetical strategy. When an ICE occurs, subsequent data will be regarded as invalid and set to missing. There are no other defined ICEs. Down-titration will not be considered as ICEs for the definition of this estimand in this study.
- Population-level summary: treatment difference in primary and secondary efficacy endpoints between aleniglipron (45, 90, and 120 mg once daily [QD]) and placebo.

Rationale for "efficacy" estimand: This Phase 2b study aims to study the efficacy of aleniglipron under the ideal condition that all participants adhere to the randomized treatment.

2.2.2. Secondary Estimand

A secondary clinical interest of this study is to estimate the treatment effect of aleniglipron relative to placebo at Week 36 in participants who meet the inclusion criteria regardless of adherence to treatment.

The secondary estimand is described by the following attributes:

- Population: age 18 – 80 participants with obesity (body mass index [BMI] ≥30 kg/m2) or overweight (BMI ≥27 kg/m^2) with at least one weight-related comorbidity who meet the protocol inclusion criteria (age 18-80, screening HbA1c <6.5%).
- Treatment: randomized treatment with titration to 1 of 3 target doses, with allowance of down- titration based on tolerability, or placebo.
- Endpoint: percent change in body weight from baseline to 36 weeks.
- Intercurrent event (ICE): permanent discontinuation of study drug, initiation of other anti-obesity medication, or bariatric surgery, whichever is earlier. The ICE is handled by the treatment strategy. All observed values, including those collected after ICEs, will be used in the analysis. There are no other defined ICEs. Down-titration will not be considered as ICEs for the definition of this estimand in this study.
- Population-level summary: treatment difference in primary and secondary efficacy endpoints between aleniglipron (45, 90, and 120 mg once daily [QD]) and placebo.

Rationale for secondary estimand: This Phase 2b study aims to study the efficacy of aleniglipron regardless of adherence to the randomized treatment.

Unless specified otherwise, safety and tolerability assessments will be conducted by comparing the safety of aleniglipron doses with placebo for the entire study period (the treatment period plus safety follow-up period where applicable), irrespective of adherence to study drug for all participants who are randomized and are exposed to at least 1 dose of study drug.

2.3. Study Design

2.3.1. Overview

This is a randomized, double-blind, placebo-controlled, dose-range finding study of the efficacy, safety, tolerability, pharmacokinetics (PK), and pharmacodynamics (PD) of multiple doses of aleniglipron in participants living with obesity or overweight with at least one weight-related comorbidity.

The study enrolled male and female participants ≥18 years and <80 years of age at the time of Screening with a BMI ≥30 kg/m^2 (obese) or BMI ≥27 kg/m^2 (overweight) with ≥1 weight-related comorbidity and who have had a stable weight (no more than 5% variation) in the last 3 months prior to screening. Enrollment of participants with a BMI <30 kg/m^2 will be capped at 10% of the planned sample size, with at least 60% of the participants enrolled with a BMI ≥35 kg/m^2.

Enrollment of female participants will also be capped to no more than 65% of the planned sample size. Efforts will be made to ensure that ethnicity and race will be approximately representative of the US population in general (e.g., including approximately 18% Hispanic, 12% Black or African American, etc.) to ensure sufficient diversity based on 2023 US Census Data.

All on-study assessments to be performed are described in the protocol's Schedule of Assessments (Appendix A). Blood samples will be collected at various times to assess biomarkers for safety monitoring as well as for PK assessments.

Participants will be randomized to 3 cohorts in a ratio of 3:4:4 and aleniglipron or placebo in a ratio of 3:1 within each Cohort as follows:

- Cohort 1: Dose level 1 aleniglipron (maintenance dose: 45 mg QD) or placebo N=60
- Cohort 2: Dose level 2 aleniglipron (maintenance dose: 90 mg QD) or placebo N=80
- Cohort 3: Dose level 3 aleniglipron (maintenance dose: 120 mg QD) or placebo N=80

Participants will receive multiple-ascending, QD doses of aleniglipron or placebo in titration steps of 4 weeks duration (refer to Figure 1 below). Thereafter, in the last titration step dose, participants will receive the target maintenance dose (target dose) of study drug for the remaining duration of treatment (for a total of 36 weeks of treatment).

Figure 1 Diagrammatic Representation of Study Design

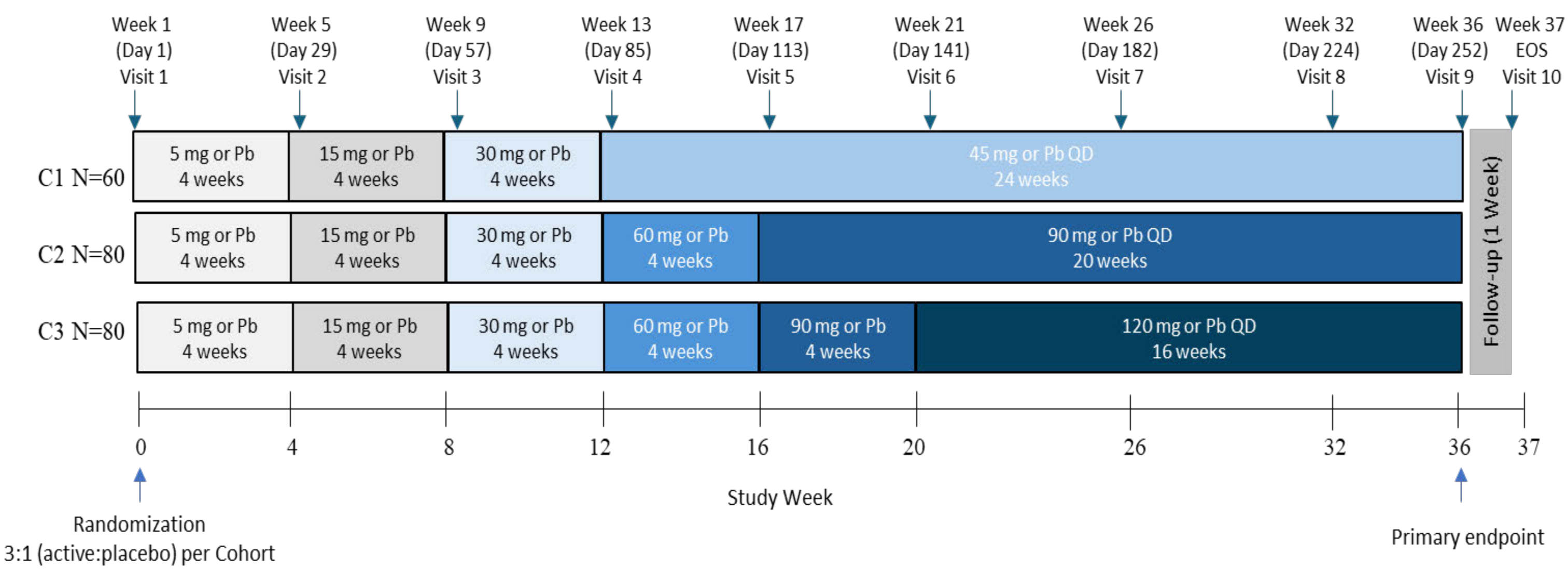

Abbreviations: C=cohort; DB=double-blind; EOS=end of study; N=number of participants; Pb=placebo; QD=once daily.

Note: At 4-week intervals, participants will be up-titrated to the next higher dose during Visit 2 (Week 5), Visit 3 (Week 9), Visit 4 (Week 13), for those in Cohorts 2 and 3 Visit 5 (Week 17), and for those in Cohort 3 Visit 6 (Week 21).

Titration Adjustments

Titration adjustments are permitted, per protocol, upon documented discussion with the Sponsor to manage participants experiencing adverse events at the current dose. Adjustments

will include down titrating to the next lower dose, or as needed, and then re-titrating up to the target dose, in consultation between the site Investigator and the Sponsor Medical Representative (SMR).

Education and coaching on tolerance strategies will be provided by qualified study personnel to enable as many participants as possible to attain their target dose.

2.3.2. Randomization and Blinding

Participants who meet the study eligibility criteria will be randomized to 3 dosing cohorts and aleniglipron/ placebo within each cohort. The allocation to aleniglipron tablet or placebo tablet will be performed using a randomization algorithm and in accordance with the randomization schedule. To obtain balance across treatment groups, the randomization will be stratified by baseline BMI and sex. The stratification factors and strata within each factor are listed below:

1. Baseline BMI
   a. ≤35 kg/m^2
   b. >35 kg/m^2
2. Sex
   a. Male
   b. Female

No more than 10% of the participants with BMI <30 kg/m^2 and no more than 65% female participants will be randomized. The study will remain blinded until data base lock.

2.3.3. Study Drug and Duration

Study interventions are all prespecified, investigational and non-investigational medicinal products intended to be administered to the study participants during the study conduct.

Delegated study site personnel will supervise and instruct participants on the administration of the study intervention (i.e., investigational product [IP]). The exact dates and times of study drug administration (when administered in the clinic) will be recorded in the electronic Case Report Form (eCRF). The exact date and time of study drug administration at home will be recorded in the e-diary.

Table 2 Study Intervention(s) Administered

Intervention Name	Aleniglipron	Placebo
Type	Drug	Control
Dose formulation	Tablet	Tablet
Unit dose strength(s)	2.5, 5, 30, and 60 mg	Not applicable

Intervention Name	Aleniglipron	Placebo
Route of administration	Oral	Oral
Sourcing	Aleniglipron tablets supplied by designated manufacturer/ distributor of Sponsor	Placebo tablets supplied by designated manufacturer/ distributor of Sponsor
Packaging and labeling	Study intervention will be provided in 75 mL oral solid pharmaceutical HDPE bottle containing 1 g desiccant enclosed with a 33 mm PP CRC (fitted with an aluminum foil induction seal). Each bottle will be labeled to conform with country requirements.	

Abbreviations: CRC=child-resistant caps; HDPE=high-density polyethylene; PP=polypropylene; QD=once daily.

Participants will receive QD doses of study drug for 252 consecutive days.

2.3.4. Sample Size Considerations

Approximately 220 participants will be randomly assigned to active treatment and placebo in a randomization ratio of 3:1 in all 3 cohorts. All participants assigned to placebo in each cohort will be pulled together as the placebo group, which will be compared to each aleniglipron treatment group in Cohort 1 to 3 to establish efficacy. The sample size calculation is based on the primary estimand and its endpoint (percent body weight change from Baseline to Week 36). Assuming an SD of 6%, a 2-sided alpha level of 0.05, 35 completers for one aleniglipron treatment group and 35 completers for the placebo group can provide approximately >90% power to detect a treatment difference of -5% between the aleniglipron treatment group and placebo in percent body weight change from Baseline to Week 36. Based on observations from prior aleniglipron studies and Week 36 results from the orforglipron Phase 2 study (discontinuation of orforglipron due to AE ranging from 10 to 17% across dose cohorts, Reference 8), assuming a 20% dropout rate in aleniglipron 45 mg group, 60 participants will be randomized for Cohort 1, with 45 participants on aleniglipron 45 mg and 15 participants on placebo. Assuming a 35% dropout rate in all other treatment groups, 80 participants will be randomized for each of Cohort 2 and 3, with 60 participants on aleniglipron and 20 participants on placebo.

3. STATISTICAL METHODOLOGY

3.1. General Considerations

All data processing, summarization and analyses will be performed using SAS® Enterprise Guide Version 8.2 or higher statistical software package.

The following principles will be applied to all TFLs unless otherwise stated in later parts of this SAP.

3.1.1. Analysis Day

Analysis day will be calculated from the date of first dose of study drug. The day of the first dose of study drug will be study Day 1, and the day immediately before study Day 1 will be study Day -1. There will be no study Day 0.

3.1.2. Definition of Baseline

Baseline for body weight, vital signs, blood clinical laboratory parameters, and ECG parameter data will be defined as the last non-missing measurement (including both scheduled and unscheduled visits) at or before randomization visit (prior to first dosing of study drug) unless otherwise specified. For numerical measurements that have duplicates or triplicates (for the same visit), the arithmetic mean should be used.

The change from baseline value will be calculated as post-baseline value minus baseline value.

3.1.3. Analysis Sets

Table 3 Definition of Analysis Sets

Participant Analysis Set	Description
All Randomized Set	All participants who are randomly assigned to a treatment group.<br>Participant disposition and background data, including demographics, baseline characteristics, and participation data, will be presented for all participants in the All Randomized Set.
Full Analysis Set	All randomized participants who receive ≥1 dose of study drug (alenigliptron or placebo).<br>Efficacy data will be presented for all participants in the Full Analysis Set.

Participant Analysis Set	Description
Safety Analysis Set	All participants who receive ≥1 dose of study drug (aleniglipron or placebo).<br>Safety and tolerability data will be presented for all participants in the Safety Analysis Set.
Pharmacokinetic Analysis Set	All participants in the Safety Analysis Set who have at least 1 quantifiable plasma concentration.<br>Concentration data will be presented for all participants in the PK Analysis Set.
Pharmacodynamic Analysis Set	All participants in the Safety Analysis Set who have sufficient data to derive ≥1 PD parameter.

3.1.4. Summary Statistics

Unless otherwise noted, all tests of treatment effects will be conducted at a 2-sided alpha level of 0.05, and confidence intervals (CIs) will be calculated at 95%, 2-sided. All tests of interactions between treatment groups and other factors will be conducted at a 2-sided alpha level of 0.10. Participants assigned to placebo in Cohorts 1 to 3 will be pooled as the treatment group of placebo. Treatment comparisons will be displayed showing the treatment difference LS means and the 95% CIs for the treatment differences, along with the p-values for the treatment comparisons.

Unless stated otherwise, statistical summaries and analyses will be conducted based on planned randomized treatment group of aleniglipron (45, 90, or 120 mg QD) and placebo, regardless of the actual treatment(s) received by the participant due to any dose modification. Continuous and categorical outcomes will be summarized by treatment group and overall unless otherwise specified.

Safety summaries will be based on the Safety Analysis Set. If a participant randomized to the placebo treatment group accidentally received any aleniglipron, this participant will be included in the aleniglipron group in that cohort.

For categorical measures, summary statistics will include sample size, frequency, and percentages. A logistic regression model will be used to examine the treatment difference in binary efficacy outcomes with missing endpoints imputed. Fisher's exact test (or Chi-square test where Fisher's exact test fails) may be used for treatment comparisons in other categorical outcomes.

Continuous data will generally be summarized with descriptive statistics including n, mean, standard deviation (SD), median, minimum, and maximum for both actual and change from baseline measurement by randomized dose and overall if applicable. The precision of the

measurement for each continuous variable will be used to determine the number of decimal places to present in tables, figures, and derived listings. Min and max values will be reported with the same precision as the units of measure. The mean and median will be reported to 1 greater decimal place, and the SD will be reported to 2 additional decimal places. Any values that require transformation to standard units (metric or Standard Unit (SI)) will be converted with the appropriate corresponding precision.

All data collected on the CRF, and laboratory data will be provided in listings. Listings and summary tables will include observed data: any imputed values for missing data are not included unless specified otherwise.

3.1.5. Assessment Window

The start day of the analysis window will follow the scheme in the table below. Where multiple measurements for a particular parameter appear within an analysis window, the result closest to the target day will be used. If equidistant, results from the scheduled visit will be used. If both are unscheduled and/or early termination visits, the later result will be used for the summary measure. For Week 36 visit (Visit 9), if a participant misses the nominal visit, data will be selected for analysis following the scheme in the table and the rules aforementioned. Otherwise, data from nominal Visit 9 will be used for analysis. Windows for assessments planned while on treatment, for Pharmacodynamic variables should exclude assessments taken after the participant's end of treatment (EOT) date.

Table 4 Assessment Window

Visit (Week)	Target Day	Visit Number	Analysis Window (days)
0	1	1	1
4	29	2	2-43
8	57	3	44-71
12	85	4	72-99
16	113	5	100-127
20	141	6	128-161
26	182	7	162-203
32	224	8	204-238
36	252	9	≥239 to OLE enrollment date or EOS date

Visits with incomplete date will not be subject to windowing for analysis but will be included in listings. Though all measures may not be used in data summaries (e.g., ≥2 lab measures within the same analysis visit window), all measurements appear in the datasets and listings. For subjects where the event date is missing, the study day and analysis window will also be missing.

3.1.6. Hypothesis Testing

The study hypothesis for the primary objective is that at least 1 dose level of aleniglipron is superior in percent reduction from baseline in body weight relative to placebo at Week 36 in participants who have obesity or are overweight with at least 1 weight-related comorbidity. Thus, the null hypothesis corresponding to the primary estimand is as follows:

- Null hypothesis: No dose of aleniglipron is different from placebo in percent reduction from baseline in body weight at Week 36 in participants who have obesity or are overweight with at least 1 weight-related comorbidity.

3.1.7. Handling of Dropouts and Missing Data

In general, for participants who are withdrawn from the study before planned study completion for any reason, all data up to the point of discontinuation will be included for analysis.

For efficacy analysis relative to the primary estimand, data greater than 2 days subsequent to an ICE will be regarded as invalid and set to missing. For the analysis of continuous data (body weight, waist circumference, BMI, and other quantitative variables) using the MMRM model, missing data will be imputed implicitly. This analysis is deemed unbiased under the missing at random assumption. For the body weight categorical analyses using logistic regression, missing values at Week 36 will be imputed by the predicted value from MMRM model using non-missing data (before ICE + 2 days) in the same treatment group, then dichotomized into status (Yes/No) of achieving various percentage of body weight reduction.

In efficacy analysis relative to the secondary estimand, the analysis of continuous data (body weight, waist circumference and BMI) at Week 36 using the ANCOVA model, missing data will be imputed based on observed data using a Copy-Increments-to-Reference (CIR) imputation approach, assuming that the benefit accrued up to ICE is retained but there is no additional residual benefit after the ICE. For the body weight categorical analyses using logistic regression, missing values at Week 36 will be imputed with the CIR method before dichotomized into weight loss status data.

Adverse events (AEs) with missing start dates will be considered as treatment-emergent unless the partial date excludes that possibility. For AEs, treatment emergent adverse events (TEAEs) and prior and concomitant medications, the first day of the month will be used to impute the missing start day and January will be used to impute missing start month. Please refer to Appendix E for imputation of missing dates.

The purpose of imputing day and month values is to determine adverse events, treatment emergence or whether a medication is a prior or a concomitant medication.

3.2. Participant Data and Study Conduct

3.2.1. Participant Disposition

Participant disposition will be summarized with counts and percentages by treatment group and overall using all participant who were randomized, including the following:

- Number of randomized participants
- Number and percent of participants who completed treatment
- Number and percent of participants who completed study
- Number and percent of participants who did not complete the treatment and reasons for early discontinuation of treatment
- Number and percent of participants who did not complete the study and reasons for early discontinuation of study
- Number and percent of participants who experienced dose interruption
- Number and percent of participants who experienced dose reduction

The percentages will be based on the All-Randomized Set.

Cumulative probability, calculated based on Kaplan-Meier method, of time from first dose to first study dose interruption, first dose reduction, premature study drug discontinuation, premature study drug discontinuation due to AE, study discontinuation and study discontinuation due to AE will be provided based on the Safety Analysis Set. Time to event in weeks will be calculated as (date of event or censor – date of first dose) / 7. EOT date will be used as censoring time for time to dose interruption/reduction, study drug discontinuation (due to AE). EOS date will be used as censoring time for time to study discontinuation (due to AE).

A listing of study disposition for all randomized participants will be provided.

A summary of "reason of screen failures" will be provided for all screened participants.

3.2.2. Protocol Deviations

Protocol deviations will be identified based on the clinical protocol to align with International Conference on Harmonization guidelines. Protocol deviations will be identified prior to unblinding.

Participants with CSR-reportable major protocol deviations will be summarized by treatment group and category based on the All-Randomized Set.

All protocol deviations will be listed by treatment group, participant and category.

3.2.3. Analysis Populations

Number and percentage of participants belonging to the respective population will be

summarized based on the All-Randomized Set.

3.2.4. Demographic and Baseline Characteristics

The following demographic and baseline characteristics on All-Randomized Set will be included but not limited to:

- Age (years)
- Age groups (<45, ≥45 to <55, ≥55 to <65, ≥65)
- Sex
- Race
- Ethnicity
- Height (cm)
- Body Weight (kg)
- Body mass index (BMI) (kg/m2)
- BMI groups (<30, ≥30 to <35, ≥35 to <40, and ≥40 kg/m^2)
- Waist circumference (cm)
- Glycated hemoglobin level (%)
- Fasting glucose level (mg/dl)
- Pre-diabetes status (HbA1c <5.7% vs ≥5.7%)
- eGFR (mL/min/1.73 m^2)
- eGFR groups (≥ 60 mL/min/1.73 m^2 vs < 60 mL/min/1.73 m^2)
- MASH (Yes vs No)
- SBP (mm Hg)
- DBP (mm Hg)
- Pulse (beat/min)
- Total cholesterol (mg/dl)
- HDL (mg/dl)
- LDL (mg/dl)
- VLDL (mg/dl)
- Triglycerides (mg/dl)

3.2.5. Medical History

Medical history will be coded to system organ class and preferred term using the Medical

Dictionary for Regulatory Activities (MedDRA) version 28.0. The number and percentage of participants reported with conditions/procedures will be summarized by treatment group and overall, grouped according to System Organ Class (SOC) and preferred term based on the All-Randomized Set.

3.2.6. Prior and Concomitant Medications

Prior and concomitant medications will be coded to anatomical therapeutic chemical (ATC) class and preferred term using the World Health Organization Drug Dictionary (WHO-DD) version WHO Drug B3 Global, March 2025.

For summary purposes, medications will be considered prior medications if they are stopped prior to the first dose of study drug and considered concomitant medications if they were taken at any time after the first dose of study drug (i.e., started prior to the first dose of study drug and were ongoing or started after the first dose of study drug).

The number and percentage of participants taking prior medication and concomitant medication will be summarized by treatment group and overall, according to WHO Drug Anatomical Therapeutic Chemical Level 3 Code Description and the WHO-DD Preferred Name. If a participant has >1 prior or concomitant medication coded to the same category, the participant will be counted only once.

Additionally, concomitant medications of interest as defined below will be summarized by treatment group:

- baseline antihypertensive therapy, by type/class
- baseline lipid lowering therapy, by type/class
- changes to baseline medication in post randomization (in term of type/class and dose):
    - antihypertensive therapy, and
    - lipid lowering therapy
- utilization after randomization of:
    - antidiarrheal medication,
    - constipation medication, and
    - antiemetic medication
    - any symptomatic treatment for headache
    - any other therapies used to treat TEAEs

Summary of prior and concomitant medications will be based on the Safety Analysis Set.

3.2.7. Study Drug Exposure and Compliance

Study drug exposure and compliance will be summarized with descriptive statistics by cohort and titration steps/weeks based on the Safety Analysis Set. Days of exposure to study drug

will be calculated as date of last dose of study drug – date of first dose of study drug + 1. Note that the exposure (days) calculation is intended to describe the length of time a participant was exposed to study drug and therefore does not take study drug interruptions into account. Total intake (mg) will be calculated as unit dose strength x number of tablets taken. Dose compliance (%) will be calculated as 100 x total dose received (mg) /total expected dose received (mg). Compliance will be based on tablet counts consumed as reported by the participants at each study visit. Cumulative dose (mg) will be calculated as total drug intake since baseline. Cumulative dose compliance (%) will be calculated as 100 x cumulative dose/total expected dose since baseline.

Dose compliance (%) category will be summarized with counts and percentages of participants with compliance in the following categories:

<80%

80-100%

>100%

Individual study drug administration data will be listed by participant.

3.2.8. Dose Modification

Study drug dose interruption and dose reduction will be assessed for each participant summarized with descriptive statistics by treatment group based on the Safety Analysis Set. A dose interruption event starts when study drug is withheld and ends when study drug is taken again. A dose reduction event starts when current dose is lower than the target dose (after 7 days of the target day for up-titration) or previous dose during up-titration, or lower than the previous dose during the maintenance dose. Total duration of dose interruption for participant is the sum of days that study drug was withheld during all dose interruptions (last date of study drug withheld – first date of study drug withheld +1). Total duration of dose reduction is calculated as the total number of days that dose is lower than scheduled dose per protocol. Count and percentage of participants with any dose modification (dose interruption or dose reduction) will be presented. Participant's total count and total duration of dose interruption or dose reduction will be summarized with descriptive statistics, by treatment group.

A table will be provided to summarize the actual maintenance dose level by treatment group. Actual maintenance dose is defined as the highest dose X such that the participant was dosed at dose X or higher for at least 80% of the dosing period from Week 20 to Week 36.

3.3. Efficacy Assessment

3.3.1. Primary Efficacy Endpoint Analysis

3.3.1.1. Definition of Endpoint(s)

The primary efficacy measure will be the percent change in body weight from baseline at Week 36.

The percent change in body weight at each nominal visit is defined as:

*(postbaseline body weight [kg] – baseline body weight [kg]) / baseline body weight [kg] * 100%*

3.3.1.2. Analysis for the Primary Estimand

The primary efficacy assessment is based on the "efficacy estimand" and will be conducted using the FAS for the primary endpoint (percent change from baseline in body weight at Week 36).

For the "efficacy estimand", the hypothetical strategy is used to handle the ICEs (permanent discontinuation of study drug), so only data collected before the occurrence of any ICEs (+2 days) will be used in the MMRM analysis as described below. Through the MMRM, the potential efficacy measures (after the ICEs) had participants not had ICEs will be implicitly imputed.

The primary efficacy comparison will be based on the MMRM model fit of percent change from baseline in body weight and the contrast between each treatment group of aleniglipron and placebo at Week 36.

The model for the analysis of the primary efficacy endpoint of percent change from baseline in body weight will be based on a restricted maximum likelihood (REML) MMRM model which includes the fixed class effects of treatment group of aleniglipron (45, 90, or 120 mg QD) and placebo, sex (male, female), baseline BMI stratum (≤35 kg/m^2 and >35 kg/m^2) at randomization, visit, and treatment-by-visit interaction, as well as the fixed covariate of baseline body weight value. An unstructured (UN) covariance structure will be used to model the within-subject variance-covariance structure for the model errors. Significance tests will be based on least squares (LS) means and Type III tests. If this analysis fails to converge, the following covariance structures will be tested in order:

- Toeplitz (TOEP)
- Autoregressive (AR(1))
- Compound symmetry (CS)

The first covariance structure that converges will be used. The Kenward-Roger approximation will be used to estimate denominator degrees of freedom.

Summary descriptives (n, mean, median, std, min and max) for change and percent change in body weight will be presented by visit. Least squares mean percent change in body weight from baseline to Week 36 will be derived along with 2-sided 95% CIs for treatment groups.

Treatment group comparisons at Week 36 will be performed at the full significance level of 0.05.

3.3.1.3. Analysis for the Secondary Estimand

The analysis of the secondary estimand will be conducted using an ANCOVA model and FAS. Missing data due to ICEs (permanent study drug discontinuation) will be imputed using copy increments in reference (CIR) multiple imputation method with reference to the placebo group according to the treatment strategy.

If study intervention is permanently discontinued, the participant should return to the clinic within 7(±3) days from the last dose for the early termination visit assessments. Missing values of body weight at Week 36 will be imputed using a CIR imputation approach.

After 100 imputations, percent change from baseline in body weight for each of the 100 complete data sets will then be analyzed using an ANCOVA model with terms for treatment group, sex (male, female), baseline BMI stratum (≤35 kg/m^2, >35 kg/m^2), and baseline body weight as covariates. The ANCOVA LSM estimates and standard deviations for the 100 data sets are pooled into one estimate and associated standard deviation using Rubin's formula (1987):

$$m_{MI} = \frac{1}{100}\sum_{i=1}^{100} m_{i,} \quad SD_{MI} = \sqrt{\frac{1}{100}\sum_{i=1}^{100} SD_i^{\,2} + \left(1+\frac{1}{100}\right)\left(\frac{1}{100-1}\right)\sum_{i=1}^{100}(m_i - m_{MI})^2},$$

where m_i and SDi are the estimated means and standard deviations for the 100 copies of the dataset, and m_{MI}, SD_{MI} are the pooled estimates.

From m_{MI} and SD_{MI}, the 95% confidence interval for the treatment differences and the associated p-value are calculated

3.3.2. Secondary Efficacy Endpoint(s) Analysis

3.3.2.1. Secondary Endpoints

The following secondary study endpoints will be analyzed with both the "efficacy estimand" and secondary estimand using the data in the FAS:

- Percentage of participants who achieve ≥5% reduction in body weight at Week 36
- Percentage of participants who achieve ≥10% reduction in body weight at Week 36
- Percentage of participants who achieve ≥15% reduction in body weight at Week 36
- Change in body weight (absolute) from Baseline to Week 36
- Change in waist circumference from Baseline to Week 36
- Change in body mass index from Baseline to Week 36

3.3.2.2. Main Analytical Approach

Change from baseline in body weight, BMI, and waist circumference will be analyzed

using the MMRM model and the "efficacy estimand" as described in Section 3.3.1.2.

Percentage of participants with ≥5%, ≥10%, and ≥15% reduction in body weight will be analyzed using a logistic regression model with treatment group, sex, baseline BMI stratum, and baseline weight value as covariates. Missing values for binary endpoints (Yes/No) will be imputed by dichotomizing the imputed body weight at Week 36 using predicted value from MMRM model aforementioned.

The odds ratio, 95% confidence interval and p-value will be presented for treatment groups versus placebo.

3.3.2.3. Analyses for the Secondary Estimand

The secondary efficacy endpoints will also be analyzed with the "secondary estimand" using the data in the FAS. Continuous endpoints (body weight, BMI, waist circumference) with missing values will be imputed and then analyzed with the same ANCOVA model as in Section 3.3.1.3. Binary endpoints will be analyzed using the same method as described in Section 3.3.2.2 with missing body weight at Week 36 imputed with the CIR multiple imputation method as specified in Section 3.3.1.3.

3.3.3. Exploratory Endpoints

3.3.3.1. Exploratory Efficacy Analysis

The following exploratory study endpoints will be summarized using descriptive statistics by treatment group and visit on FAS data.

The MMRM model for the "primary estimand" will also be used for analyzing the following endpoints (not exhaustive list):

- Change in triglycerides from baseline to Week 36
- Change in free fatty acids (FFA) from baseline to Week 36
- Change in high-sensitivity C-reactive protein (hsCRP) from baseline to Week 36
- Change in HDL cholesterol from baseline to Week 36
- Change in LDL cholesterol from baseline to Week 36
- Change in total cholesterol from baseline to Week 36
- Change in resting blood pressure from baseline to Week 36
- Change in fasting glucose from baseline to Week 36
- Change in HbA1c from baseline to Week 36
- Percentage of participants who achieve ≥20% reduction in body weight at Week 36

3.3.3.2. Pharmacokinetic and Pharmacokinetic/Pharmacodynamic Methods

Aleniglipron concentration data will be summarized and analyzed using a population PK approach via nonlinear mixed-effects modeling. The relationships between aleniglipron dose and/or concentration and selected efficacy, tolerability, and safety endpoints may be characterized. Additionally, the impact of intrinsic and extrinsic factors, such as age, weight, gender, and renal function on PK and/or PD parameters, may be examined as needed. Additional

analyses may be conducted if they are deemed appropriate. Population PK and/or PD parameters and analysis will be described in a separate report.

3.3.3.3. Patient-Reported Outcomes

Short-Form-36 Health Survey Version 2, Acute Form

The following domain and component scores will be derived from the Short-Form-36 Health Survey Version 2, Acute Form (SF-36 v2):

- Mental Component Score
- Physical Component Score
- Physical Functioning domain
- Role-Physical domain
- Bodily Pain domain
- General Health domain
- Vitality domain
- Social Functioning domain
- Role-Emotional domain, and
- Mental Health domain

For each of the above parameters, the raw scores will be transformed into the domain scores (t- scores) and the following analyses for the actual value and change from baseline value conducted:

- Descriptive summaries by treatment group and visit, and
- MMRM analysis as described in Section 3.3.1.2

Impact of Weight on Quality of Life-Lite Clinical Trial

The following parameters will be included from the Impact of Weight on Quality of Life-Lite Clinical Trial (IWQOL-Lite-CT):

- IWQOL-Lite-CT total score (all items: Items 1 through 20)
- Physical Function composite score (5 items: Items 1 through 3, 16, and 17)
- Physical composite score (7 items: Items 1 through 5, 16, and 17), and
- Psychosocial composite score (13 items: Items 6 through 15, 18, 19, and 20)

The IWQOL-Lite-CT total and composite scores range from 0 to 100, with higher scores reflecting better levels of functioning.

The IWQOL-Lite-CT scores are computed according to the IWQOL-Lite scoring rules (Kolotkin and Crosby 2002), as following:

- Each composite raw score will be calculated if a minimum of 50% of the items for that composite has a non-missing value; the total score will be calculated if a minimum of 75% of all 20 items has a non-missing value.
  - physical composite score: 4 of 7 items

    - physical function composite score: 3 of 5 items
    - psychosocial composite score: 7 of 13 items
    - IWQOL-Lite-CT total score: 15 of 20 items
- If the minimum required number of items are answered for a composite then:
    - Calculate the average of the valid non-missing responses corresponding to the items in the total or each composite (1 = "never" or "not at all true" and 5 = "always" or "completely true")
    - The composite score is then calculated by transforming the raw composite score to the 0 (worst)-to-100 (best) metric using the following formula for every participant at each time point:

$$100\ (S_{max} - C_{avg})/(S_{max} - S_{min})$$

        - C_{avg} is the raw average score of all nonmissing item responses in the composite; this average must be a number between 1 and 5, inclusive
        - S_{max} is the maximum possible raw score value (i.e., 5)
        - S_{min} is the minimum possible raw score value (i.e., 1)
        - Inserting the maximum and minimum possible score values, the formula is reduced to $100\ (5 - C_{avg})/4$.

For the total and each composite score, the following analyses for the actual value and change from baseline value will be conducted:

- Descriptive summaries by treatment group and visit, and
- MMRM analysis as described in Section 3.3.1.2

Hunger and Satiety Evaluation

The count and percentage of participants hunger and satiety evaluation response categories at each time point will be summarized by nominal visit, evaluation and treatment group.

Score of each of bothersome symptoms listed below for participants who experienced bothersome symptoms since starting of medication or last visit will be summarized with descriptive statistics by visit and treatment group.

Bothersome symptom: nausea, vomiting, diarrhea, constipation, bloating, abdominal pain, headache, other.

3.4. Safety Assessment

Unless specified otherwise, safety and tolerability assessments will use an estimand comparing safety of aleniglipron doses with placebo irrespective of adherence to study drug including data collected during the treatment period using the Safety Analysis Set.

Safety evaluations will be based on the incidence, severity, seriousness, and relationship of

AEs, vital signs, physical examination findings, clinical laboratory results, and ECGs.

Abnormalities in clinical laboratory parameters and the ECG parameter QTcF will be based on predefined normal ranges and will be tabulated by dose group showing participant counts and percentages.

3.4.1. Adverse Events (AEs)

All AEs will be coded to system organ class (SOC) and preferred term (PT) using MedDRA version 27.1. Treatment-emergent adverse events (TEAEs) are defined as AEs that start or worsen in severity after the first dose of study drug, and onset date is within 7 days after the last dose of study drug. The severity of each AE will be graded using the most current version of the National Cancer Institute Common Terminology Criteria for Adverse Events (NCI-CTCAE) (Version 5). Related events are defined as events that are related, possibly, or probably related to study drug. AEs with a missing relationship are considered related. At each level of subject summarization, a subject is classified according to the closest relationship if the subject reported one or more events. For events with a missing severity during the baseline period, it will be treated as "mild" in severity for determining treatment-emergence. Events with a missing severity during the postbaseline period will be treated as "severe" and treatment-emergence will be determined by comparing to baseline severity. A participant with multiple occurrences of an AE will be counted only once in the respective AE category. A patient with multiple CTCAE grades for the same PT will be summarized under the maximum CTCAE grade recorded for the event.

In AE summaries, the primary system organ class will be presented by descending order of frequency (alphabetically for ties), then PTs will be sorted by descending order of frequency within each system organ class (alphabetically for ties). The sort order for the PT will be based on their frequency. Adverse events of special interest (AESI) will include: major adverse cardiovascular event (MACE), heart failure syndrome, acute pancreatitis, hypoglycemia, thyroid malignancies, supraventricular arrhythmia and cardiac conductive disorders, hepatobiliary disorders, severe gastrointestinal (GI) AEs, acute renal events, depressive disorder and/or suicide ideation, hypersensitivity, and C-cell hyperplasia. AESI will be identified as described in Appendix B: Searching Criteria for AESIs.

An overview summary of AEs will be provided including counts and percentages of participants (and event counts) with the following:

- Any TEAEs
- Any study drug related TEAEs
- Any TEAEs of special interest (AESIs)
- Any serious AEs (SAEs)
- Any study drug related SAEs
- Any TEAEs leading to dose reduction
- Any TEAEs leading to dose interruption

- Any TEAEs leading to permanent discontinuation of study drug
- Any TEAEs leading to discontinuation from study
- Any AEs leading to death

The following TEAE summaries of number and percentage of participants as well as event counts will also be produced:

- TEAEs by SOC and PT
- TEAEs related to study drug by SOC and PT
- TEAEs by SOC, PT, and worst reported severity
- Treatment-emergent SAEs by SOC and PT
- Common TEAEs (frequency > 5% of all participants) by PT
- Treatment-emergent AESI by category, SOC and PT
- Serious AESI by category, SOC and PT
- TEAEs leading to study drug discontinuation by SOC and PT
- TEAEs leading to study discontinuation by SOC and PT
- Prevalence of GI-related TEAEs by week/titration step, including intermittent AE*
- Prevalence of GI-related TEAEs by week/titration step, excluding intermittent AE
- Prevalence of GI-related TEAEs by week/titration step, target dose level and maximum severity including intermittent AE
- Prevalence of GI-related TEAEs by week/titration step, target dose level and maximum severity excluding intermittent AE
- Incidence of GI-related TEAEs by week/titration step and severity, including intermittent AE
- Incidence of GI-related TEAEs by week/titration step and severity, excluding intermittent AE
- Prevalence of treatment-emergent nausea/vomiting/diarrhea/constipation by week/titration step and maximum severity, including intermittent AE
- Prevalence of treatment-emergent nausea/vomiting/diarrhea/constipation by week/titration step and maximum severity, excluding intermittent AE
- Prevalence of treatment-emergent nausea/vomiting/diarrhea/constipation by week/titration step, target dose level and maximum severity, including intermittent AE
- Prevalence of treatment-emergent nausea/vomiting/diarrhea/constipation by week/titration step, target dose level and maximum severity, excluding intermittent AE
- Incidence of treatment-emergent nausea/vomiting/diarrhea/constipation by week/titration step and severity
- Incidence of treatment-emergent nausea/vomiting/diarrhea/constipation by week/titration step, target dose level and severity

*Intermittent AEs are AEs reported with “intermittent” or “occasionally” in the term.

Listings of all AEs, SAEs, AESIs, AEs leading to discontinuation of study drug, AEs leading to withdrawal from the study, and AEs leading to death will be presented by site, treatment group subject/sex/age(enrollment), SOC, PT, AE onset date/day, AE resolution date/day, severity, seriousness, AESI category, action taken, outcome, and drug relatedness.

3.4.2. Vital Signs

Observed values and changes from baseline for vital signs (systolic and diastolic BP, HR) will be summarized by treatment group and visit using descriptive statistics (n, mean, SD, median, minimum, and maximum).

Counts and percentages of participants with treatment-emergent abnormal sitting systolic blood pressure (SBP), sitting diastolic blood pressure (DBP), and pulse will be presented by treatment for participants who have both baseline and at least 1 postbaseline result. A treatment-emergent high result is defined as a change from a value less than or equal to the high limit at baseline to a value greater than the high limit at any time that meets the specified change criteria during the postbaseline period. A treatment-emergent low result is defined as a change from a value greater than or equal to the low limit at baseline to a value less than the low limit at any time that meets the specified change criteria during the postbaseline period. To assess decreases, change from the minimum value during the baseline period to the minimum value during the postbaseline period will be used. To assess increases, changes from the maximum value during the baseline period to the maximum value during the postbaseline period will be used. Both planned and unplanned measurements will be included in the analysis. The criteria for identifying participants with treatment-emergent vital sign abnormalities are stated in Table 5.

Table 5 Categorical Criteria for Abnormal Treatment-Emergent Blood Pressure and Heart Rate Measurements

Parameter	Low	High
Systolic BP (mm Hg) (Supine or sitting – forearm at heart level)	≤90 and decrease from baseline ≥20	≥139 and increase from baseline ≥20
Diastolic BP (mm Hg) (Supine or sitting – forearm at heart level)	≤50 and decrease from baseline ≥10	≥90 and increase from baseline ≥10
Heart rate (bpm) (Supine or sitting)	<50 and decrease from baseline ≥15	>100 and increase from baseline ≥15

Abbreviations: BP = blood pressure; bpm = beats per minute.

In addition, the following analyses will be conducted by treatment:

- counts and percentages of participants who had resting heart rate (HR) changes from baseline at 2 consecutive visits of more than two predefined thresholds:
  - 10 beats per minute (bpm)
  - 20 bpm
- counts and percentages of participants who had at least 1 resting HR exceeding 100 bpm,
  and

- counts and percentages of participants who had at least 1 resting HR exceeding 100 bpm occurring at 2 consecutive study visits.

Listings of vital signs will be presented.

3.4.3. Clinical Laboratory Data

Clinical laboratory safety data (hematology, serum chemistry, and coagulation as listed in Appendix C) will be reported with units based on the reported values. If there are multiple reported units collected for the same parameter, the results will be reported in the International System of Unit. Normal reference ranges and abnormal results will be used in the summary of laboratory data.

Observed values and changes from baseline for continuous clinical laboratory parameters will be summarized at each visit using descriptive statistics (n, mean, SD, median, minimum, and maximum) and tabulated for each treatment group.

Categorical clinical laboratory data will be summarized at each visit using participant counts and percentages and tabulated by treatment group. For categorical clinical laboratory data, the number and percentage of participants with normal and abnormal values will be summarized by treatment group.

Shift tables will present the number and percentage of participants within each baseline CTCAE 5.0 grade versus each postbaseline CTCAE 5.0 grade for lipase, amylase, calcitonin, aspartate aminotransferase, alanine transaminase, alkaline phosphatase, gamma-glutamyl transferase, total bilirubin, direct bilirubin, plasma glucose, serum creatinine and estimated glomerular filtration rate lab parameters by each treatment group. Values will be summarized by their worst post baseline grade.

Potential drug-induced liver injuries will be evaluated with liver test parameters ALT, AST and total bilirubin. Number and percentage of participants will be tabulated with the following categories for pre-baseline and post-baseline period separately by treatment group:

ALT: >= 3 ULN; >= 5 ULN; >= 10 ULN

AST: >= 3 ULN; >= 5 ULN; >= 10 ULN

Total bilirubin: >= 2 ULN

ALT or AST >= 3 ULN and total bilirubin >= 2 ULN

Individual participant profiles will be listed for any laboratory parameters with ≥1 postdose value outside the laboratory's reference ranges and deemed clinically significant. The listing will include participant identification, treatment group, laboratory collection date, study day, analyte name, and analyte finding.

The MMRM model or ANCOVA (if MMRM model is not applicable) may be used for the analysis during the treatment period for the continuous measurements for selected lab tests.

3.4.4. Electrocardiograms

The mean value for the triplicate ECG will be utilized for the ECG analysis.

The following ECG parameters will be analyzed: PR interval, QT interval, QRS duration, QTcF, and ventricular HR. Observed values and changes from baseline for ECG parameters will be summarized at each timepoint using descriptive statistics (n, mean, SD, median, minimum, and maximum) and tabulated for each dose group.

For QTcF, the count and percentage of participants with values >450, >480, and >500 msec or an increase from baseline >30 and >60 msec will also be presented, in accordance with International Council for Harmonization of Technical Requirements for Pharmaceuticals for Human Use (ICH) E14 (note for guidance on the clinical evaluation of QT/corrected QT interval prolongation and proarrhythmic potential for non-antiarrhythmic drugs, Committee for Medicinal Products for Human Use/ICH/2/04).

For HR, the number of participants with values ≥100 bpm, or an increase from baseline ≥ 20 bpm will also be presented by treatment group.

An MMRM analysis will be conducted to analyze changes from baseline in HR and QTc interval.

Individual ECG data will be listed.

3.4.5. Physical Examination Data

Physical examination data will be listed only. Any postdose clinically significant findings will be reported as AEs.

3.5. Pharmacokinetic Assessment

3.5.1. Sample Collections for Pharmacokinetic Analysis

Blood samples will be collected for assessment of plasma concentrations of aleniglipron. Clinical staff is required to collect each blood PK sample at the scheduled timepoints within the windows indicated (Appendix D: Pharmacokinetic Sampling Schedule). The actual date and time (as hh:mm) of each blood sample collection will be recorded in the eCRF.

3.5.2. Handling Missing or Below the Lower Limit of Quantification Data

If an aleniglipron concentration for a PK sample is missing, the record will be excluded from this analysis. If the date or time for a PK sample or for the dose immediately prior to a PK sample is missing, the relevant concentration will either be deleted from the analysis dataset, or, if feasible, the date or time will be imputed according to protocol specifications or based on a logical interpretation of available information from data proximate to the missing data.

The timing and frequency of concentration measurements reported as below the lower limit of quantitation (BLQ) will be summarized. If BLQ samples (post administration of the drug) represent less than or equal to 5% of the total samples in the dataset, and if the occurrence of BLQ samples does not appear to be treatment-group dependent, all BLQ values will be removed from the analysis dataset prior to analysis. This method corresponds to the M1 method recommended by Beal as appropriate for many datasets. If BLQ samples represent greater than 5% of the data or if the occurrence of BLQ samples is substantially treatment-

group dependent, an alternative procedure may be considered for implementation based upon a strategy discussed by Gasherbrum Bio and the vendor for population PKPD analysis.

3.5.3. Pharmacokinetic Concentration

Individual aleniglipron concentration data will be listed. Individual plasma concentrations of aleniglipron may be summarized by cohort for each dose level for the Pharmacokinetic Analysis Set descriptively (n, mean, SD, coefficient of variation, minimum, maximum, median, geometric mean [GM], and geometric coefficient of variation). A box plot of C_{trough} plasma concentration of GSBR-1290 by cohort and visit will be generated. Individual plasma concentration against actual sampling time points relative to dosing time will be plotted for Day 1 post dose (5mg) hours (up to 6 hrs).

3.5.4. Pharmacokinetic Parameters

This study only collects sparse samples for PK analysis. Non compartmental analysis (NCA) will not be performed. A population PKPD analysis will be performed and report separately.

3.6. Pharmacodynamic Assessment

The PD effect of aleniglipron will be assessed on the following variables (including and not limited to):

Cardiovascular PD biomarkers measured at Week 0, Week 12, and Week 36:

- Free fatty acid (FFA)
- High-sensitivity C- reactive protein (hsCRP)

Other clinically relevant biomarkers measured on Week 12 and Week 36:

- Triglycerides
- Total cholesterol
- Low-density lipoprotein (LDL) cholesterol
- High-density lipoprotein (LDL) cholesterol

Descriptive analysis (n, mean, SD, median, minimum, and maximum) will be generated on absolute value and change from baseline (FFA and hsCRP) of the above variables by treatment group and timepoint on the PD analysis sets.

3.7. Other Analyses

3.7.1. Subgroup Analyses

Subgroup analyses of the primary endpoint will be conducted to assess consistency of the treatment effect across the following subgroups based on MMRM models and using the

primary estimand:

- Age group: <45, ≥45 to <55, ≥55 to <65, ≥65
- Sex: female versus male
- Baseline BMI: <30, ≥30 to <35, ≥35 kg/m^2
- Pre-diabetes status: HbA1c <5.7% versus ≥5.7%
- Race
- Ethnicity

If the number of participants is too small (<10%) within a subgroup, then the subgroup categories may be redefined prior to unblinding the study.

In the models for the subgroup analysis, the same fixed effects used in the primary analysis MMRM model plus factors of subgroup, 2-way interaction of subgroup and treatment, 2-way interaction of subgroup and visit, and 3-way interaction of treatment, visit, and subgroup. The interaction of subgroup and treatment estimated from the model will be evaluated to assess the treatment by subgroup interaction. If the model does not converge, other structures for the covariance matrix, as noted earlier, will be applied in the model. Additionally, highest order interaction terms of the subgroup may be dropped from models that fail convergence.

Treatment difference estimates and 95% CI will be provided at each category of each subgroup.

3.7.2. Other Exploratory Analyses

The primary endpoint will be analyzed using the primary estimand with assigned treatment group replaced by the actual dose used during the maintenance period as defined below.

This analysis will include all participants that are treated ≥ 80% of the dosing period of Week 20 to Week 36, which is equivalent to dosing days ≥ 90 within this period of time.

Actual maintenance dose is defined as the highest dose X such that the patient was dosed at dose X or higher for at least 80% of the dosing period from Week 20 to Week 36. If number of participants at dose level Y is too small that the MMRM model doesn't converge at Y, these participants should be combined with the next lower dose level.

3.7.3. Dose-Response Analysis

Due to the potentially delayed body weight response to dose titration, body weight taken on Visit 1 is considered to correspond to 0 mg dose level, body weight on Visit 2 corresponds to dose level started on Visit 1, and so on until Visit 9 (Week 36).

The impact of dose titration on body weight over time is analyzed with a random coefficient model using SAS® Proc Mixed. The model will include the fixed effects of treatment group of aleniglipron (45, 90, or 120 mg QD) and placebo, strata (sex by

baseline BMI stratum [≤35 kg/m^2, >35 kg/m^2]) and (continuous) dose level, treatment by dose level interaction and baseline body weight value. An unstructured (UN) covariance structure is assumed for the random intercept and slope effects.

Slopes for each treatment group will be estimated along with 95% CI. Mean predicted body weight will be plotted with dose level.

Additionally, a sensitivity analysis will be conducted on the dose response data with a non-linear Emax model defined below:

$$E = E_0 - \frac{(E_{\max} + \eta)d}{d + d_{50}}$$

Where

E = expected treatment effect relative to the baseline body weight

E_0 = baseline body weight at the zero-exposure level

E_{max} = maximum treatment effect relative to the baseline body weight

d = titration dose

d_{50} = dose level at half-maximal effect (median effective dose)

η = random subject term

SAS® Proc NLIN will be used to fit the model and parameter estimates will be presented. Predicted responses constructed with parameter estimates will be plotted.

4. ANALYSIS TIMING

4.1. Interim Analysis

An interim (efficacy and safety) analysis may be conducted to inform dose escalation schemes. No formal interim analysis for purpose of early termination for efficacy or futility is planned. Unblinded sponsor personnel will be limited and documented within a Data Access/Unblinding Plan. All Investigators, site staff, and participants will remain blinded up to participant entry to the open label extension.

4.2. Final Analysis

After the database is locked, the randomized treatment assignments will be unblinded and the pre-final TFLs will be generated. After all comments on the pre-final analysis have been resolved and the study database is declared final, the final TFLs for the final analysis will be generated. If there were no changes to the pre-final analysis or the study database, the pre-final TFLs may be considered final. Results presented in graphical and tabular presentations will be structured in alignment with CDISC standards.

5. CHANGES FROM PROTOCOL-SPECIFIED STATISTICAL ANALYSES

This SAP supersedes the statistical analysis described in the protocol amendment 4 signed on May 07, 2025.

6. APPENDIX

6.1. Appendix A: Schedule of Assessments

**Study periods**	**Screen**	**Treatment period**									**EOS /ETV[k]**
**Visit identifier**	**SCRN**	**1**	**2**	**3**	**4**	**5**	**6**	**7**	**8**	**9[n]**	**DB EOS**
**Visit day**	**-28 to -1**	**1**	**29**	**57**	**85**	**113**	**141**	**182**	**224**	**252**	**259**
**Visit week**	**-4 to -1**	**1**	**5**	**9**	**13**	**17**	**21**	**26**	**32**	**36**	**37**
**Visit Interval Window (days)**		**0**	**±3**	**±3**	**±3**	**±3**	**±3**	**±3**	**±3**	**±3**	**±3**
**Assessments**											
Informed consent	X										
Eligibility criteria	X										
Demographics	X										
Medical history	X										
Drug screen	X										
Body height	X										
Body weight (with certified calibrated scales and in hospital gown) in triplicate	X	X	X	X	X	X	X	X	X	X	X
Waist circumference in triplicate		X					X		X	X	X
Concomitant medication	X	X	X	X	X	X	X	X	X	X	X
Physical examination (full)	X										X
Physical examination (symptom-directed)		X	X	X	X	X	X	X	X	X	
Vital signs (BP, HR, temperature) [a]	X	X	X	X	X	X	X	X	X	X	X
12-Lead ECG [b]	X	X	X	X	X	X	X	X	X	X	X
COVID-19 test [c]	X	X									
FSH (postmenopausal women ≤55 years)	X										
Pregnancy test (urine) before dosing [d]	X	X	X	X	X	X	X	X	X	X	X
Pregnancy test (serum) before dosing only if urine test is positive [d]	X										
Clinical laboratory tests [e]	X	X	X	X	X	X	X	X	X	X	X
Viral serology	X										
Adverse events [f]		X	X	X	X	X	X	X	X	X	X
Randomization		X									
Dose administration [g]		X	X	X	X	X	X	X	X	X	
HbA1c	X	X			X					X	

Study periods	Screen	Treatment period									EOS /ETV[k]
Visit identifier	SCRN	1	2	3	4	5	6	7	8	9[n]	DB EOS
Visit day	-28 to -1	1	29	57	85	113	141	182	224	252	259
Visit week	-4 to -1	1	5	9	13	17	21	26	32	36	37
Visit Interval Window (days)		0	±3	±3	±3	±3	±3	±3	±3	±3	±3
**Assessments**											
PK sampling (blood) [h]		X	X	X	X	X	X	X	X	X	
Cardiovascular PD biomarkers (blood)[i]		X			X					X	
Fill out electronic diary daily, including dose administration time		X	X	X	X	X	X	X	X	X	
SF-36 acute version		X					X			X	X
IWQOL-Lite CT		X					X			X	X
Hunger and satiety evaluation[j]		X	X	X	X	X	X	X	X	X	X
PHQ-9 health questionnaires	X	X	X	X	X	X	X	X	X	X	X
C-SSRS screening/baseline[l]	X										
C-SSRS since the last visit		X	X	X	X	X	X	X	X	X	X
Discuss nutrition plan and physical activity[m]	X	X	X	X	X	X	X	X	X	X	

AbbreviationsBP=blood pressure; COVID-19=coronavirus disease 2019; C-SSRS=Columbia Suicide Severity Rating Scale; DB=double-blind; ECG=electrocardiogram; EOS=end of study; ETV=early termination visit; FSH=follicle-stimulating hormone; HbA1c=hemoglobin A1c; HR=heart rate; IWQOL-Lite CT=Impact of Weight on Quality of Life-Lite Clinical Trials Version; OLE=open-label extension; PD=pharmacodynamic; PHQ-9=Patient Health Questionnaire-9; PK=pharmacokinetic; QD=once daily; SF-36=Short Form 36 health survey acute version; TSH=stimulating hormone.

a. a. Vital signs including blood pressure (in triplicate), heart rate, and temperature (Screening and Day 1 only) will be collected predose. BP will be collected from the left arm in at least 2-minute intervals. See Section 8 for order of procedures. Caffeine-containing products (e.g., coffee, tea, chocolate, and caffeine-containing sodas, colas, etc.) may not be consumed within 2 hours before measuring vital signs and ECGs. Predose vital signs assessments should be performed within 1 hour before dosing.

b. b. ECGs will be collected and stored centrally. ECGs will be collected in triplicate along with each PK draw and within 15 minutes before the PK draw. See Section 8 for order of procedures.

c. c. COVID-19 test only if clinically indicated or per site policy.

d. d. For women of child-bearing potential, urine pregnancy test is performed first, and if positive or inconclusive, confirmed with a serum pregnancy test.

e. e. Includes fasting glucose after Screening visit (i.e., starting on Day 1). Clinical safety laboratory tests will include hematology, serum chemistry, and coagulation. See Section 10.2. TSH is only collected during the Screening Visit. Norovirus and/or influenza testing may be conducted as standard of care as needed.

f. f. Adverse events and serious adverse events collected from time of signing the informed consent form through 30 days after date of the last dose.

g. g. On-study visit days, participants should take all of their study drug at the clinic within 15 minutes due to the PK assessments being performed on those days. At 4-week intervals, participants will be up-titrated to the next higher dose during Visit 2 (Week 5), Visit 3 (Week 9), Visit 4 (Week 13), for those in Cohorts 2 and 3 Visit 5 (Week 17), and for those in Cohort 3 Visit 6 (Week 21). Participants not moving into the OLE will receive the last dose of study drug at Visit 9 (Week 36). Participants who are moving into the OLE will receive their newly assigned dose of study drug at Visit 9 (Week 36) and will continue with QD dosing, with the next study visit occurring at

Week 38 according to Table 2. See Pharmacy Manual for return of any unused study drug from the blinded portion of the study.

h. For PK timepoints, refer to Appendix D. See Section 8 for order of procedures.
i. i. PD markers, including, but not limited to, free fatty acid, IL-6, ProBNP, high-sensitivity C-reactive protein, and succinate, will be measured at Day 1, Day 85, and Day 252.
j. j. The hunger and satiety evaluation will be performed for the first 7 days following each clinic visit, except for Visit 10/EOS/ETV which will be evaluated on the day of the visit only. If the hunger and satiety evaluation is received with triggering results, site staff should conduct an unscheduled phone call visit to evaluate the condition of the participant's adverse event. On Visit 1, Day 1, the hunger and satiety evaluation should be completed prior to study drug administration as a baseline measurement.
k. k. ETV should be scheduled 7 (±3) days from the last dose. Visit DB EOS will only occur for participants not moving on to the OLE or for those who terminate the study early. Participants moving into the OLE will have a separate Visit 10 (OLE) at Week 38 as described in Table 2.
l. l. C-SSRS baseline must be completed prior to any study-related activities.
m. m. There will be a discussion on nutrition approximately 3 days prior to each up-titration visit in addition to a documented discussion on nutrition plan and physical activity at the clinic visit.
n. n. Participants who will be rolling over to the OLE will re-consent at an appropriate timepoint provided there are no ongoing related AEs. They return all blinded study drug and receive their new study drug at Visit 9 (Week 36).

Note: Participants who discontinue study drug should return to the clinic at the next scheduled visit for the ETV assessments.

6.2. Appendix B: Searching Criteria for AESIs

Event	Criteria/Search Term
**MACE**	Adjudication
**Heart failure syndrome**	Adjudication
**Acute pancreatitis**	Adjudication
**Thyroid malignancies**	Adjudication
**Cholecystitis**	Adjudication
**Hypoglycemia**	MedDRA PT of hypoglycemia
**Supraventricular arrhythmia and cardiac conductive disorders**	Arrhythmia related investigations, signs, and symptoms SMQ (20000051), narrow and broad terms<br>Supraventricular tachyarrhythmias SMQ (20000057), broad and narrow terms<br>Tachyarrhythmia terms, nonspecific SMQ (20000164), narrow terms only<br>Ventricular tachyarrhythmias SMQ (20000058), narrow terms only<br>Conduction defects SMQ (20000056), narrow terms only<br>Cardiac conduction disorders HLT (10000032), all PTs
**Acute Renal Event**	Narrow terms in Acute renal failure SMQ (20000003)
**Hypersensitivity**	Anaphylactic reaction SMQ (20000021)<br>Hypersensitivity SMQ (20000214)<br>Angioedema SMQ (20000024)
**Hepatobiliary Disorder**	Broad and narrow terms in the Liver related investigations, signs and symptoms SMQ (20000008)<br>Broad and narrow terms in the Cholestasis and jaundice of hepatic origin SMQ (20000009)<br>Broad and narrow terms in the Hepatitis non-infections SMQ (20000010)<br>Broad and narrow terms in the Hepatic failure, fibrosis and cirrhosis and other liver damage SMQ (20000013)<br>Narrow terms in the Liver-related coagulation and bleeding disturbances SMQ (20000015)<br>Narrow PTs in Gallbladder related disorders SMQ (20000124)<br>Narrow PTs in Biliary tract disorders SMQ (20000125)<br>Narrow PTs in Gallstone related disorders SMQ (20000127)
**Severe GI AEs**	SOC of GI of grade 3 or higher
**C-cell hyperplasia**	MedDRA PT of thyroid C-cell hyperplasia

Event	Criteria/Search Term
**Depressive disorder and/or suicide ideationep**	Narrow terms from the Depression and suicide/self-injury SMQ as defined in MedDRA SMQs 20000037 [Suicide/self-injury] and 200000167 [Depression (excluding suicide and self-injury)]

6.3. Appendix C: Clinical Laboratory Tests

<table>
<tr><th>Hematology</th><th colspan="3">Serum Chemistry, Including Liver Function</th></tr>
<tr><td>Hemoglobin
Hematocrit
Red blood cell indices
Thrombocyte count (platelets)
WBC count (absolute and %) with differential</td><td rowspan="3">Sodium
Potassium
Chloride
Bicarbonate
Phosphate
Calcium
Glucose
Amylase
Lipase
Uric acid
Magnesium</td><td rowspan="3">Creatine kinase
Creatinine
eGFR calculated using CKD-EPI formula
Blood urea nitrogen
Alanine transaminase
Alkaline phosphatase
Aspartate transaminase
Gamma-glutamyl transferase
Total bilirubin
Conjugated bilirubin
Unconjugated bilirubin</td><td rowspan="3">Total cholesterol
High-density lipoprotein
Low-density lipoprotein
Triglycerides (serum)
Free fatty acid
Calcitonin
Albumin
Globulins
Total protein
Lactate dehydrogenase
Thyroid stimulating hormone (at Screening Visit only)
Hemoglobin A1c
High-sensitivity C-reactive protein</td></tr>
<tr><th>Coagulation</th></tr>
<tr><td>Prothrombin time
INR
aPTT
Fibrinogen</td></tr>
<tr><th>Virology</th><th colspan="2">Drug Screen (Urine)</th><th>Confirmation of Postmenopausal Status</th></tr>
<tr><td>HIV-1/-2 antibody
HbsAg
Hepatitis C virus antibody and viral load
COVID-19 (SARS-CoV-2 antigen and viral load)[a]
Norovirus, influenza[c]</td><td colspan="2">Opiates
Cocaine
Benzodiazepines
Barbiturates
Methadone
Amphetamines</td><td>FSH for postmenopausal women
Serum hCG [b]
Urine hCG [b]</td></tr>
</table>

Abbreviations: aPTT = activated partial prothrombin time; CKD-EPI = Chronic Kidney Disease Epidemiology Collaboration; COVID-19 = coronavirus disease 2019; eGFR = estimated glomerular filtration rate; FSH = follicle-stimulating hormone; HbsAg = hepatitis B surface antigen; hCG = human chorionic gonadotropin; HIV = human immunodeficiency virus; INR = international normalized ratio; PCR = polymerase chain reaction; SARS-COV-2 = severe acute respiratory syndrome coronavirus 2; WBC = white blood cell.

a. COVID-19 test performed only if clinically indicated as determined by the Investigator; SARS-CoV-2 antigen test is performed first, and then PCR test for confirmation if clinically indicated.
b. If the urine hCG is positive or inconclusive, pregnancy will be confirmed by a serum hCG test.
c. Norovirus and/or influenza testing may be performed if indicated by the Investigator.

6.4. Appendix D: Pharmacokinetic Sampling Schedule

Study Day	Study Week	Planned Dose (Irrespective of Down-Titration)	PK Timepoints (Exact Time of Blood Collection Must be Recorded)	ECG
Day 1	Week 1	All cohorts: start of 5 mg	Within 45 minutes before dosing<br>Between 2 and 4 hours postdose	Yes
Day 29	Week 5	All cohorts: start of 15 mg	Within 45 minutes before dosing<br>Between 2 and 4 hours postdose	Yes
Day 57	Week 9	All cohorts: start of 30 mg	Within 45 minutes before dosing<br>Between 2 and 4 hours postdose	Yes
Day 85	Week 13	Cohort 1 – start of 45 mg<br>Cohort 2 and 3 – start of 60 mg	Within 45 minutes before dosing<br>Between 2 and 4 hours postdose	Yes
Day 113	Week 17	Cohort 1 – after 4 weeks at 45 mg<br>Cohort 2 and 3 – start of 90 mg	Within 45 minutes before dosing<br>Between 2 and 4 hours postdose	Yes
Day 141	Week 21	Cohort 1 – after 8 weeks at 45 mg<br>Cohort 2 – after 4 weeks at 90 mg<br>Cohort 3 – start of 120 mg	Within 45 minutes before dosing<br>Between 2 and 4 hours postdose	Yes
Day 182	Week 26	At steady state for all cohorts	Within 45 minutes before dosing	Yes
Day 224	Week 32	At steady state for all cohorts	Within 45 minutes before dosing	Yes
Day 252	Week 36	Last day of dosing for participants not moving to the open-label extension	Within 45 minutes before dosing<br>Between 2 and 4 hours postdose	Yes

Abbreviations: ECG=electrocardiogram; PK=pharmacokinetic.
Note: On-study days with PK assessments, all study tablets should be consumed at the clinic within 15 minutes.

Note: ECGs will be collected in triplicate along with each PK draw and within 15 minutes before the PK draw. See the Site Procedural Manual for details.
Note: To verify compliance with the study dosing regimen and adherence to the protocol requirements, random PK blood draws may be conducted on enrolled participants to monitor the concentration of the study drug in the bloodstream.

6.5. Appendix E: Imputation Rules for Missing AE and Medication Dates

An imputation of end date should be performed before an imputation of start date.

6.5.1. Imputation Rules for AE End Date and Medication End Date

If the end date (AE end date, medication end date) is partially missing or completely missing, the following rules will be applied:

- If the end date is completely missing and not ongoing, then the end date will be imputed as the end of study (EOS) date.
- Partial end date where only the year is known:
  - The end date will be imputed as the EOS date if the year of end date is the same as the year of the EOS date; otherwise, day and month of end date will be imputed as the last day of the year (i.e., 31 December).
- Partial end date where only the year and month are known:
  - The end date will be imputed as the EOS date if the year and month of end date are the same as year and month of the EOS date; otherwise, day of end date will be imputed as the last day of the month.

6.5.2. Imputation Rules for AE Start Date and Medication Start Date

If the start date (AE start date, medication start date) is partially missing or completely missing, the following rules will be applied:

- If the start date is completely missing, then the start date will be imputed as the first dose date.
- Partial start date where only the year is known:
  - The start date will be imputed as the first dose date if the year of start date is the same as the year of the first dose date; otherwise, day and month of start date will be imputed as the first day of the year (i.e., 01 January).
- Partial start date where only the year and month are known:
  - The start date will be imputed as the first dose date if the year and month of start date are the same as year and month of the first dose date; otherwise, day of start date will be imputed as the first day of the month.
- If the imputed start date is after the end date, the start date will be imputed as the end date.
- If the imputed AE start date is before the informed consent date, the AE start date will be imputed as the informed consent date.

7. REFERENCES

1. Pharmaceutical Statistics Using SAS: A Practical Guide (SAS Press) 1st Edition by Alex Dmitrienko (Author), Christy Chuang-Stein
2. Anwar YA, White WB. Ambulatory monitoring of the blood pressure devices, analysis, and clinical utility. In: William White, editor. Blood Pressure Monitoring in Cardiovascular Medicine and Therapeutics. Humana Press, Inc; 2001: p 64
3. Darken P, Nyberg J, Ballal S, Wright D. The attributable estimand: a new approach to account for intercurrent events. *Pharm Stat*. 2020;19(5):626-635. https://doi.org/10.1002/pst.2019
4. [FDA] E9(R1) Statistical principles for clinical trials: Addendum: Estimands and sensitivity analysis in clinical trials. https://www.fda.gov/media/148473/download
5. [EMA] European Medicines Agency. ICH E9 (R1) addendum on estimands and sensitivity analysis in clinical trials to the guideline on statistical principles for clinical trials. Step 5. EMA/CHMP/ICH/436221/2017. Published February 17, 2020. Accessed June 30, 2022. https://www.ema.europa.eu/en/documents/scientific-guideline/ich-e9-r1-addendum-estimands- sensitivity-analysis-clinical-trials-guideline-statistical-principles_en.pdf
6. Ernst ME, Bergus GR. Noninvasive 24-hour ambulatory blood pressure monitoring: overview of technology and clinical applications. *Pharmacotherapy*. 2002;22(5):597-612. https://doi.org/10.1592/phco.22.8.597.33212
7. Kolotkin RL, Crosby RD. Psychometric evaluation of the impact of weight on quality of life-lite questionnaire (IWQOL-lite) in a community sample. *Qual Life Res*. 2002;11(2):157-71. https://doi.org/10.1023/a:1015081805439
8. Wharton S, Blevins T, Connery L, et al; GZGI Investigators. Daily oral GLP-1 receptor agonist orforglipron for adults with obesity. *N Engl J Med*. 2023; 389:877-888. [PMID: 37351564] doi: 10.1056/NEJMoa2302392
9. Lipkovich, I, Ratitch, B, Mallinckrodt, CH. Causal Inference and Estimands in Clinical Trials. *Stat. in Biopharm Research*. 2020; 12:54-67. https://doi.org/10.1080/19466315.2019.1697739
10. Pickering TG, Hall JE, Appel LJ, et al. Recommendations for blood pressure measurement in humans and experimental animals. Part 1: blood pressure measurement in humans: a statement for professionals from the Subcommittee of Professional and Public Education of the American Heart Association Council on High Blood Pressure Research. *Hypertension*. 2005;45(1):142-161. https://doi.org/10.1161/01.HYP.0000150859.47929.8e
11. Carpenter JR, Roger JH, Kenward MG. Analysis of longitudinal trials with protocol deviation: a framework for relevant, accessible assumptions, and inference via multiple imputation. J Biopharm Stat. 2013;23(6):1352–71. https://doi.org/10.1080/10543406.2013.834911.

STATISTICAL ANALYSIS PLAN

DATE OF PLAN:

Version 1.0/ 10 November 2025

STUDY DRUG:

Aleniglipron (GSBR-1290)

PROTOCOL NUMBER:

GSBR-1290-06

STUDY TITLE:

A Phase 2b, Randomized, Double-blind, Placebo-controlled, Dose-range Finding Study of the Efficacy and Safety of Multiple Doses of Aleniglipron (GSBR-1290) in Participants Living with Obesity (Body Mass Index ≥ 30 kg/m^2) or Overweight (Body Mass Index ≥ 27 kg/m^2) with at Least One Weight-related Comorbidity

SPONSOR:

Gasherbrum Bio, Inc.

601 Gateway Blvd. Suite 900
South San Francisco, CA 94080

This study is being conducted in compliance with good clinical practice, including the archiving of essential documents.

SIGNATURE PAGE

[redacted]

Electronically signed by: [redacted]
Reason: I approve this document
Date: Nov 11, 2025 15:13:25 EST

[redacted]

Date

[redacted]

Electronically signed by: [redacted]
Reason: I approve this document
Date: Nov 11, 2025 15:15:26 EST

[redacted]

Date

[redacted] Electronically signed by: [redacted]
Reason: I approve this document
Date: Nov 11, 2025 13:48:08 PST

Date

[redacted]
Sponsor Medical Representative
Gasherbrum Bio, Inc.

[redacted] Electronically signed by: [redacted]
Reason: I approve this document
Date: Nov 11, 2025 14:31:23 PST

Date

[redacted]

Gasherbrum Bio, Inc.

[redacted] Electronically signed by: [redacted]
Reason: I approve this document
Date: Nov 11, 2025 14:35:03 PST

Date

[redacted]
Sponsor Biostatistics Representative
Gasherbrum Bio, Inc

[redacted]
Reason: I approve this document
Date: Nov 11, 2025 15:05:28 PST

Date

[redacted]

Gasherbrum Bio, Inc.

VERSION HISTORY

Version	Version Date	Description
1.0	10 November 2025	Approved version

TABLE OF CONTENTS

TITLE PAGE 1
VERSION HISTORY 4
LIST OF ABBREVIATIONS 8
1. INTRODUCTION 10
2. STUDY OBJECTIVE(S) AND ENDPOINT(S) 11
2.1. Study Objective(s) 11
2.2. Study Design 12
2.2.1. Overview 12
2.2.2. Randomization and Blinding 14
2.2.3. Study Drug and Duration 14
2.2.4. Sample Size Considerations 15
3. STATISTICAL METHODOLOGY 16
3.1. General Considerations 16
3.1.1. Analysis Day 16
3.1.2. Definition of Baseline 16
3.1.3. Analysis Sets 16
3.1.4. Summary Statistics 17
3.1.5. Assessment Window 17
3.1.6. Hypothesis Testing 18
3.1.7. Handling of Dropouts and Missing Data 19
3.2. Participant Data and Study Conduct 19
3.2.1. Participant Disposition 19
3.2.2. Protocol Deviations 20
3.2.3. Analysis Populations 20
3.2.4. Demographic and Baseline Characteristics 20
3.2.5. Prior and Concomitant Medications 21
3.2.6. Study Drug Exposure and Compliance 22
3.2.7. Dose Modification 22
3.3. Efficacy Assessment 23
3.3.1. Primary Efficacy Endpoint Analysis 23
3.3.1.1. Definition of Endpoint(s) 23

3.3.1.2. Analytical Approach 23
3.3.2. Secondary Efficacy Endpoint(s) Analysis....23
3.3.2.1. Secondary Endpoints 23
3.3.2.2. Analytical Approach 23
3.3.2.3. Exploratory Efficacy Analysis 24
3.3.2.4. Pharmacokinetic and Pharmacokinetic/Pharmacodynamic Methods 24
3.3.2.5. Patient-Reported Outcomes 24
3.4. Safety Assessment 26
3.4.1. Adverse Events (AEs) 26
3.4.2. Vital Signs....28
3.4.3. Clinical Laboratory Data 29
3.4.4. Electrocardiograms 30
3.4.5. Physical Examination Data 30
3.5. Pharmacokinetic Assessment 30
3.5.1. Sample Collections for Pharmacokinetic Analysis 30
3.5.2. Handling Missing or Below the Lower Limit of Quantification Data 31
3.5.3. Pharmacokinetic Concentration 31
3.5.4. Pharmacokinetic Parameters 31
3.6. Pharmacodynamic Assessment....31
4. ANALYSIS TIMING 33
4.1. Interim Analysis....33
4.2. Final Analysis 33
5. CHANGES FROM PROTOCOL-SPECIFIED STATISTICAL ANALYSES 33
6. APPENDIX....34
6.1. Appendix A: Overall Schedule of Assessments for Open-Label Extension 34
6.2. Appendix B: Searching Criteria for AESIs 36
6.3. Appendix C: Clinical Laboratory Tests....38
6.4. Appendix D: Pharmacokinetic Sampling Schedule for Participants Who Were on Placebo Moving into Open-Label Extension 40
6.5. Appendix E: Imputation Rules for Missing AE and Medication Dates 41
6.5.1. Imputation Rules for AE End Date and Medication End Date....41
6.5.2. Imputation Rules for AE Start Date and Medication Start Date 41

6.6. Appendix F: List of TLFs for OLE Interim Analysis 42
7. REFERENCES 43

List of Tables

Table 1 Study Objectives and Endpoints 11
Table 2 Study Intervention(s) Administered 14
Table 3 Definition of Analysis Sets 16
Table 4 Assessment Window 18
Table 5 Categorical Criteria for Abnormal Treatment-Emergent Blood Pressure and Heart Rate Measurements 28

List of Figures

Figure 1 Diagrammatic Representation of Study Design 13
Figure 2 Diagrammatic Representation of Open-Label Extension Study Design 13

LIST OF ABBREVIATIONS

Abbreviation	Definition
ADaM	Analysis Data Model
AE	Adverse event
ANCOVA	Analysis of covariate
ATC	Anatomical therapeutic chemical
BMI	Body mass index
BP	Blood pressure
CDISC	Clinical Data Interchange Standards Consortium
eCRF	Electronic case report form
CSR	Clinical Study Report
CV	Coefficient of variation
DSMB	Data Safety Monitoring Board
ECG	Electrocardiogram
EOS	End of study
EOT	End of treatment
FDA	Food and Drug Administration
GM	Geometric mean
HbA1c	Hemoglobin A1c
HOV	Healthy overweight/obese volunteers
HR	Heart rate
ICH	International Council for Harmonisation of Technical Requirements for Pharmaceuticals for Human Use
MAR	Missing at random
MedDRA	Medical Dictionary for Regulatory Activities
MI	Multiple imputation
MMRM	Mixed model for repeated measurement
MMTT	Mixed meal tolerance test
MTD	Maximum tolerated dose
NCI-CTCAE	National Cancer Institute Common Terminology Criteria for Adverse Events
OLE	Open label extension
PCL	Protocol clarification letter
PD	Pharmacodynamics
PK	Pharmacokinetics
QD	Once daily
REML	Restricted maximum likelihood
SAE	Serious adverse event
SAP	Statistical Analysis Plan
SD	Standard deviation
SE	Standard error
SDTM	Study Data Tabulation Model
SOC	System organ class
T2DM	Type 2 diabetes mellitus
TEAE	Treatment-emergent adverse event
TESAE	Treatment-emergent serious adverse event
WHO	World Health Organization

WHO-DD	World Health Organization Drug Dictionary

1. INTRODUCTION

The purpose of this Statistical Analysis Plan (SAP) is to provide a description of the statistical methods to be implemented for the primary analysis of open-label extension (OLE) data from the Phase 2b clinical study GSBR- 1290-06 (ACCESS) of aleniglipron (GSBR-1290) for the treatment of participants living with obesity or overweight and at least one weight-related comorbidity. If circumstances arise during the study such that more appropriate analytic procedures become available, the SAP may be revised. The statistical definitions and analytical methods described in this SAP supersede those in the protocol. The SAP will be finalized prior to final analysis. Any revisions to the endpoint analyses will be made prior to the database freeze for the final analysis. Reasons for such revisions will be described in the final Clinical Study Report (CSR). The scope of analyses for this SAP pertains to the OLE phase.

2. STUDY OBJECTIVE(S) AND ENDPOINT(S)

2.1. Study Objective(s)

Table 1 Study Objectives and Endpoints

	Objectives	Endpoints
**Primary**	To evaluate long-term safety and tolerability of aleniglipron in adults with obesity or overweight with a comorbidity at Week 72	• TEAEs and SAEs<br>• AESI<br>• Laboratory parameters<br>• Electrocardiogram<br>• Vital signs
**Secondary**	Durability and maintenance of weight loss from Week 0 – 72, and Week 36 - 72	• Percent change in body weight<br>• Percentage of participants who achieve ≥5% reduction in body weight<br>• Percentage of participants who achieve ≥10% reduction in body weight<br>• Percentage of participants who achieve ≥15% reduction in body weight<br>• Change in body weight (absolute)<br>• Change in waist circumference<br>• Change in body mass index
**Exploratory**	To assess the long term effect of aleniglipron on other parameters	• Change in triglycerides, FFA, and hsCRP<br>• Change in patient-reported outcomes (SF-36 acute version, IWQOL-Lite CT, and hunger/satiety evaluation)<br>• Change in HDL and LDL cholesterol<br>• Change in resting blood pressure<br>• Change in fasting glucose

2.2. Study Design

2.2.1. Overview

This is a randomized, double-blind, placebo-controlled, dose-range finding study of the efficacy, safety, tolerability, pharmacokinetics (PK), and pharmacodynamics (PD) of multiple doses of aleniglipron in participants living with obesity or overweight with at least one weight-related comorbidity.

The study enrolled male and female participants ≥18 years and <80 years of age at the time of Screening with a BMI ≥30 kg/m^2 (obese) or BMI ≥27 kg/m^2 (overweight) with ≥1 weight-related comorbidity and who have had a stable weight (no more than 5% variation) in the last 3 months prior to screening. Enrollment of participants with a BMI <30 kg/m^2 will be capped at 10% of the planned sample size, with at least 60% of the participants enrolled with a BMI ≥35 kg/m^2.

Enrollment of female participants will also be capped to no more than 65% of the planned sample size. Efforts will be made to ensure that ethnicity and race will be approximately representative of the US population in general (e.g., including approximately 18% Hispanic, 12% Black or African American, etc.) to ensure sufficient diversity based on 2023 US Census Data.

All on-study assessments to be performed are described in the protocol's Schedule of Assessments (Appendix A). Blood samples will be collected at various times to assess biomarkers for safety monitoring as well as for PK assessments.

Participants will be randomized to 3 cohorts in a ratio of 3:4:4 and aleniglipron or placebo in a ratio of 3:1 within each Cohort as follows:

- Cohort 1: Dose level 1 aleniglipron (maintenance dose: 45 mg QD) or placebo N=60
- Cohort 2: Dose level 2 aleniglipron (maintenance dose: 90 mg QD) or placebo N=80
- Cohort 3: Dose level 3 aleniglipron (maintenance dose: 120 mg QD) or placebo N=80

Participants will receive multiple-ascending, QD doses of aleniglipron or placebo in titration steps of 4 weeks duration (refer to Figure 1 below). Thereafter, in the last titration step dose, participants will receive the target maintenance dose (target dose) of study drug for the remaining duration of treatment (for a total of 36 weeks of treatment).

Participants may consent for an additional 36 weeks of treatment in an open label extension (OLE). Participants who continue on OLE will be treated as follows:

- Participants on Cohort 1 45 mg active drug will titrate (12-weeks, 60 mg, 75 mg, 90 mg in 4 week titration steps) to 120 mg.
- Participants on Cohort 2 90 mg active drug will continue at an increased dose of 120 mg.

- Participants on Cohort 3 120 mg active drug will continue at 120 mg.
- Participants on placebo will be starting on 2.5 mg/QD of aleniglipron and follow the titration scheme shown in Figure 2, to achieve 120 mg/qd in 24 weeks.

The maximum study duration is 77 weeks (4 weeks for Screening, 36 weeks for blinded study treatment, 36 weeks for open-label treatment, and 1 week for follow-up).

Figure 1 Diagrammatic Representation of Study Design

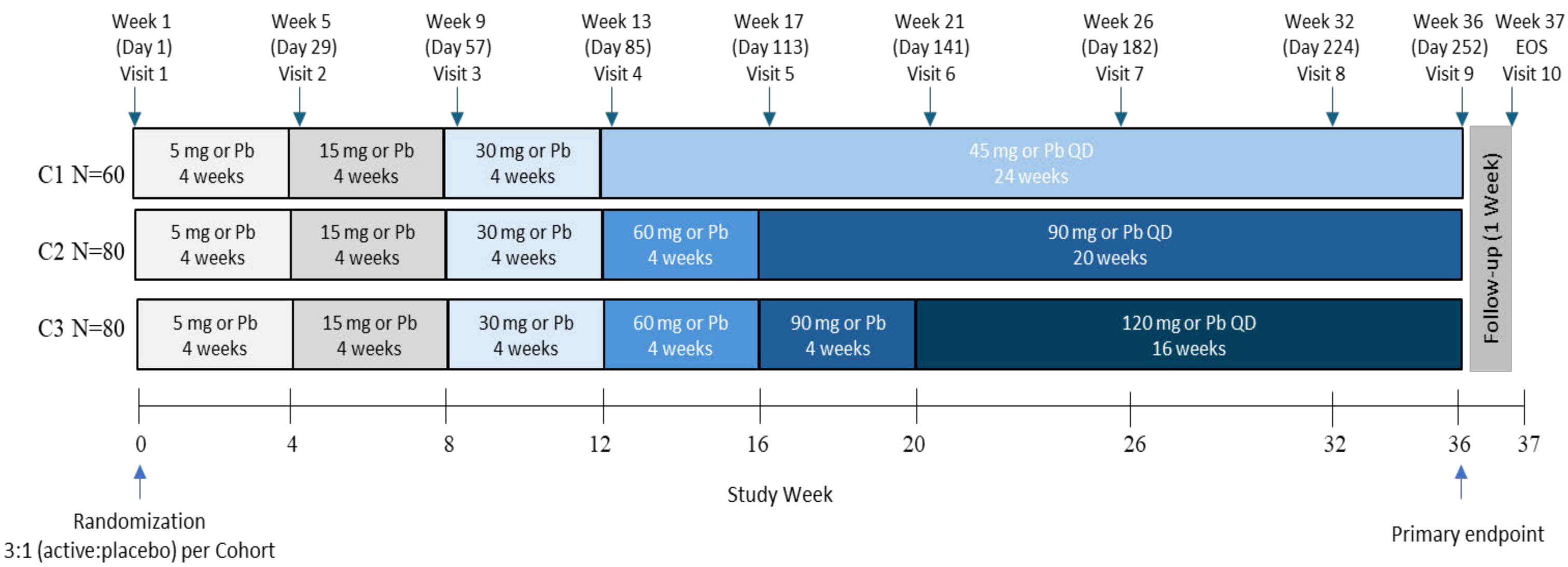

Abbreviations: C=cohort; DB=double-blind; EOS=end of study; N=number of participants; Pb=placebo; QD=once daily.

Note: At 4-week intervals, participants will be up-titrated to the next higher dose during Visit 2 (Week 5), Visit 3 (Week 9), Visit 4 (Week 13), for those in Cohorts 2 and 3 Visit 5 (Week 17), and for those in Cohort 3 Visit 6 (Week 21).

Figure 2 Diagrammatic Representation of Open-Label Extension Study Design

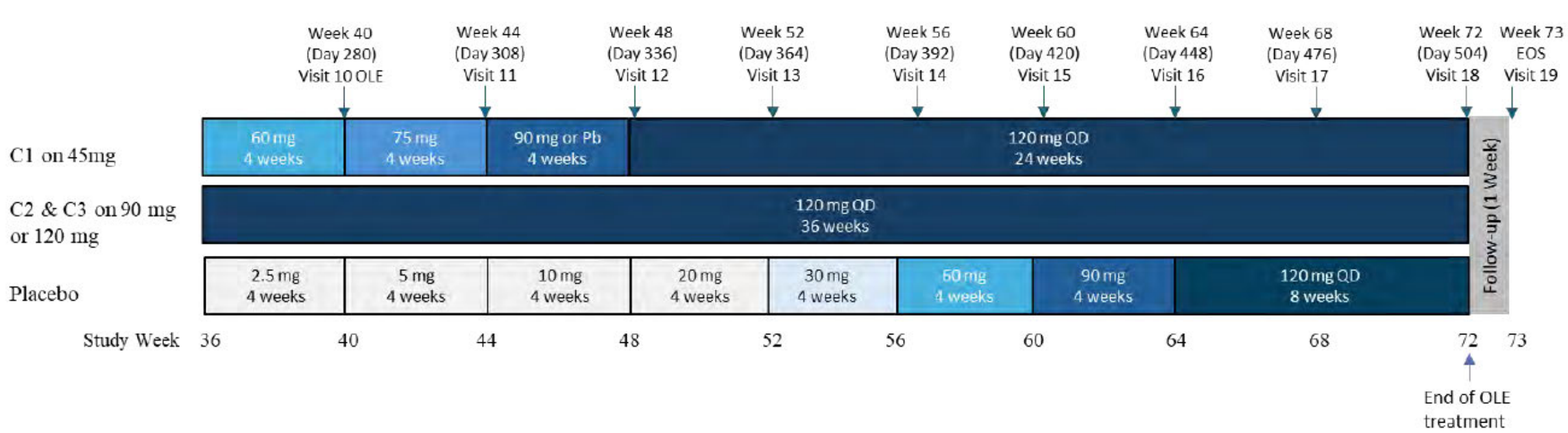

Abbreviations: C=cohort; EOS=end of study; OLE=open-label extension; QD=once daily.

Note: Note: Up-titrations will occur at Weeks 40, 44 and 48 for participants rolling over to the OLE from Cohort 1, and at Weeks 40, 44, 48, 52, 56, 60, and 64 for those who were on placebo.

Titration Adjustments

Titration adjustments are permitted, per protocol, upon documented discussion with the Sponsor to manage participants experiencing adverse events at the current dose. Adjustments will include down titrating to the next lower dose, or as needed, and then re-titrating up to the target dose, in consultation between the site Investigator and the Sponsor Medical Representative (SMR).

Education and coaching on tolerance strategies will be provided by qualified study personnel to enable as many participants as possible to attain their target dose.

2.2.2. Randomization and Blinding

This is the OLE phase of the study. Randomization and blinding are not applicable.

2.2.3. Study Drug and Duration

Study interventions are all prespecified, investigational medicinal products intended to be administered to the study participants during the study conduct.

Delegated study site personnel will supervise and instruct participants on the administration of the study intervention (i.e., investigational product [IP]). The exact dates and times of study drug administration (when administered in the clinic) will be recorded in the electronic Case Report Form (eCRF). The exact date and time of study drug administration at home will be recorded in the e-diary.

Table 2 Study Intervention(s) Administered

**Intervention Name**	**Aleniglipron**
Type	Drug
Dose formulation	Tablet
Unit dose strength(s)	2.5, 5, 30, and 60 mg
Route of administration	Oral
Sourcing	Aleniglipron tablets supplied by designated manufacturer/ distributor of Sponsor

Packaging and labeling	Study intervention will be provided in 75 mL oral solid pharmaceutical HDPE bottle containing 1 g desiccant enclosed with a 33 mm PP CRC (fitted with an aluminum foil induction seal). Each bottle will be labeled to conform with country requirements.

Abbreviations: CRC=child-resistant caps; HDPE=high-density polyethylene; PP=polypropylene; QD=once daily.

Participants will receive QD doses of study drug for 252 consecutive days and the potential of approximately 504 days for participants completing the OLE portion of the study.

2.2.4. Sample Size Considerations

OLE participants are those who completed the double-blind phase of the study and consent to continue with the OLE phase.

3. STATISTICAL METHODOLOGY

3.1. General Considerations

All data processing, summarization and analyses will be performed using SAS® Enterprise Guide Version 8.2 or higher statistical software package.

The following principles will be applied to all TFLs unless otherwise stated in later parts of this SAP.

3.1.1. Analysis Day

Analysis day will be calculated from the date of first dose of study drug in the double-blind phase. The day of the first dose of study drug will be study Day 1.

3.1.2. Definition of Baseline

Baseline for body weight, vital signs, blood clinical laboratory parameters, and ECG parameter data will be defined as the last non-missing measurement (including both scheduled and unscheduled visits) at or prior to first dosing of study drug in OLE phase unless otherwise specified. For numerical measurements that have duplicates or triplicates (for the same visit), the arithmetic mean should be used.

The change from baseline value will be calculated as post-baseline value minus baseline value.

3.1.3. Analysis Sets

Table 3 Definition of Analysis Sets

Participant Analysis Set	Description
OLE Analysis Set	All participants who receive ≥1 dose of study drug (aleniglipron) in the OLE phase.<br>Participant disposition and background data, including demographics, baseline characteristics, participation data, efficacy data, and safety and tolerability data will be presented for all participants in the OLE Analysis Set.
Safety Analysis Set	All participants who receive ≥1 dose of study drug (aleniglipron or placebo) in the entire study.<br>Safety and tolerability data will be presented for all participants in the Safety Analysis Set.

Participant Analysis Set	Description
Pharmacokinetic Analysis Set	All participants in the OLE Analysis Set who have at least 1 quantifiable plasma concentration.<br>Concentration data will be presented for all participants in the PK Analysis Set.
Pharmacodynamic Analysis Set	All participants in the OLE Analysis Set who have sufficient data to derive ≥1 PD parameter.

3.1.4. Summary Statistics

Unless stated otherwise, statistical summaries and analyses will be conducted based on treatment group of aleniglipron (DB Aleniglipron 45 mg, DB Aleniglipron 90 mg, and DB Aleniglipron 120 mg) and placebo, regardless of the actual treatment(s) received by the participant due to any dose modification. Continuous and categorical outcomes will be summarized by treatment group and overall unless otherwise specified.

Safety summaries will be based on both of the OLE Analysis Set and the Safety Analysis Set.

For categorical measures, summary statistics will include sample size, frequency, and percentages.

Continuous data will generally be summarized with descriptive statistics including n=, mean, standard deviation (SD), median, minimum, and maximum for both actual and change from baseline measurement by dose and overall if applicable. The precision of the measurement for each continuous variable will be used to determine the number of decimal places to present in tables, figures, and derived listings. Min and max values will be reported with the same precision as the units of measure. The mean and median will be reported to 1 greater decimal place, and the SD will be reported to 2 additional decimal places. Any values that require transformation to standard units (metric or Standard Unit (SI)) will be converted with the appropriate corresponding precision.

All data collected on the CRF, and laboratory data will be provided in listings. Listings and summary tables will include observed data: any imputed values for missing data are not included unless specified otherwise.

3.1.5. Assessment Window

The start day of the analysis window will follow the scheme in the table below. Where multiple measurements for a particular parameter appear within an analysis window, the result closest to the target day will be used. If equidistant, results from the scheduled visit will be used. If both are unscheduled and/or early termination visits, the later result will be

used for the summary measure. For Week 36 visit (Visit 9), if a participant misses the nominal visit, data will be selected for analysis following the scheme in the table and the rules aforementioned. Otherwise, data from nominal Visit 9 will be used for analysis. Windows for assessments planned while on treatment, for Pharmacodynamic variables should exclude assessments taken after the participant's end of treatment (EOT) date.

Table 4 Assessment Window

Study Phase	Visit (Week)	Target Day	Visit Number	Analysis Window (days)
Double Blind	0	1	1	1
	4	29	2	2-43
	8	57	3	44-71
	12	85	4	72-99
	16	113	5	100-127
	20	141	6	128-161
	26	182	7	162-203
	32	224	8	204-238
	36	252	9	239-enroll to OLE
Open-label Extension	40	280	10	Enroll to OLE -294
	44	308	11	295-322
	48	336	12	323-350
	52	364	13	351-378
	56	392	14	379-406
	60	420	15	407-434
	64	448	16	435-462
	68	476	17	463-490
	72	504	18	>=491

Visits with incomplete date will not be subject to windowing for analysis but will be included in listings. Though all measures may not be used in data summaries (e.g., ≥2 lab measures within the same analysis visit window), all measurements appear in the datasets and listings. For subjects where the event date is missing, the study day and analysis window will also be missing.

3.1.6. Hypothesis Testing

No hypothesis test is planned.

3.1.7. Handling of Dropouts and Missing Data

In general, for participants who are withdrawn from the study before planned study completion for any reason, all data up to the point of discontinuation will be included for analysis.

For efficacy analysis, all data will be summarized as observed. Missing data will not be imputed.

Adverse events (AEs) with missing start dates will be considered as treatment-emergent unless the partial date excludes that possibility. For AEs, treatment emergent adverse events (TEAEs) and prior and concomitant medications, the first day of the month will be used to impute the missing start day and January will be used to impute missing start month. Please refer to Appendix E for imputation of missing dates.

The purpose of imputing day and month values is to determine adverse events, treatment emergence or whether a medication is a prior or a concomitant medication.

3.2. Participant Data and Study Conduct

3.2.1. Participant Disposition

For the open-label phase, participant disposition at the OLE baseline will be summarized with counts and percentages by treatment group and overall using all participant who enrolled in the open-label phase, including the following:

- Number of participants treated in OLE
- Number and percent of participants who completed treatment
- Number and percent of participants who completed study
- Number and percent of participants who did not complete the treatment and reasons for early discontinuation of treatment
- Number and percent of participants who did not complete the study and reasons for early discontinuation of study
- Number and percent of participants who experienced dose interruption
- Number and percent of participants who experienced dose reduction
- Duration of follow-up in weeks

The percentages will be based on the OLE analysis set.

Cumulative probability, calculated based on Kaplan-Meier method , of time from first dose to first study dose interruption, first dose reduction, premature study drug discontinuation,

premature study drug discontinuation due to AE, study discontinuation and study discontinuation due to AE will be provided based on OLE Analysis Set. EOT date will be used as censoring time for time to dose interruption/reduction. EOS date will be used as censoring time for time to study discontinuation (due to AE).

A listing of study disposition for all OLE participants will be provided.

3.2.2. Protocol Deviations

Protocol deviations will be identified based on the clinical protocol to align with International Conference on Harmonization guidelines. Protocol deviations will be identified prior to unblinding.

Participants with CSR-reportable major protocol deviations will be summarized by treatment group and category based on the OLE analysis set.

All protocol deviations will be listed by treatment group, participant and category.

3.2.3. Analysis Populations

Number and percentage of participants belonging to the respective population will be summarized based on the OLE analysis set.

3.2.4. Demographic and Baseline Characteristics

The following demographic and characteristics at OLE baseline on the OLE analysis set will be included but not limited to:

- Age (years)
- Age groups (<45, ≥45 to <55, ≥55 to <65, ≥65)
- Sex
- Race
- Ethnicity
- Height (cm)
- Body Weight (kg)
- Body mass index (BMI) (kg/m2)
- BMI groups (<30, ≥30 to <35, ≥35 to <40, and ≥40 kg/m^2)
- Waist circumference (cm)
- Glycated hemoglobin level (%)
- Fasting glucose level (mg/dl)

- Pre-diabetes status (HbA1c <5.7% vs ≥5.7%)
- eGFR (mL/min/1.73 m^2)
- eGFR groups (≥ 60 mL/min/1.73 m^2 vs < 60 mL/min/1.73 m^2)
- SBP (mm Hg)
- DBP (mm Hg)
- Pulse (beat/min)
- Total cholesterol (mg/dl)
- HDL (mg/dl)
- LDL (mg/dl)
- VLDL (mg/dl)
- Triglycerides (mg/dl)

3.2.5. Prior and Concomitant Medications

Prior and concomitant medications will be coded to anatomical therapeutic chemical (ATC) class and preferred term using the World Health Organization Drug Dictionary (WHO-DD) version WHO Drug B3 Global, March 2025.

For summary purposes, medications will be considered prior medications if they are stopped prior to the first dose of study drug and considered concomitant medications if they were taken at any time after the first dose of study drug (i.e., started prior to the first dose of study drug and were ongoing or started after the first dose of study drug).

The number and percentage of participants taking prior medication and concomitant medication will be summarized by treatment group and overall, according to WHO Drug Anatomical Therapeutic Chemical Level 3 Code Description and the WHO-DD Preferred Name. If a participant has >1 prior or concomitant medication coded to the same category, the participant will be counted only once.

Additionally, concomitant medications of interest as defined below will be summarized by treatment group:

- baseline antihypertensive therapy, by type/class
- baseline lipid lowering therapy, by type/class
- changes to baseline medication in post randomization (in term of type/class and dose):
  - antihypertensive therapy, and
  - lipid lowering therapy
- utilization after randomization of:
  - antidiarrheal medication,

- constipation medication, and
- antiemetic medication
- any symptomatic treatment for headache
- any other therapies used to treat TEAEs

Summary of prior and concomitant medications will be based on the Safety Analysis Set.

3.2.6. Study Drug Exposure and Compliance

Study drug exposure and compliance in OLE phase will be summarized with descriptive statistics by cohort and titration steps/weeks based on the OLE Analysis Set. Days of exposure to study drug will be calculated as date of last dose of study drug – date of first dose of study drug + 1. Note that the exposure (days) calculation is intended to describe the length of time a participant was exposed to study drug and therefore does not take study drug interruptions into account. Total intake (mg) will be calculated as unit dose strength x number of tablets taken. Dose compliance (%) will be calculated as 100 x total dose received (mg) /total expected dose received (mg). Compliance for the placebo group will be based on tablet counts consumed as reported by the participants at each study visit. Cumulative dose (mg) will be calculated as total drug intake since baseline. Cumulative dose compliance (%) will be calculated as 100 x cumulative dose/total expected dose since baseline.

Dose compliance (%) category will be summarized with counts and percentages of participants with compliance in the following categories:

<80%

80-100%

>100%

Individual study drug administration data will be listed by participant.

3.2.7. Dose Modification

Study drug dose interruption and dose reduction will be assessed for each participant summarized with descriptive statistics by treatment group based on the Safety Analysis Set.

- A dose interruption event starts when study drug is withheld and ends when study drug is taken again.
- A dose reduction event starts when current dose is lower than the target dose (after 7 days of the target day for up-titration) or previous dose during up-titration, or lower than the previous dose during the maintenance dose.

Total duration of dose interruption for participant is the sum of days that study drug was withheld during all dose interruptions (last date of study drug withheld – first date of study drug withheld +1). Total duration of dose reduction is calculated as the total number of days

that dose is lower than scheduled dose per protocol. Count and percentage of participants with any dose modification (dose interruption or dose reduction) will be presented. Participant's total count and total duration of dose interruption or dose reduction will be summarized with descriptive statistics, by treatment group.

A table reflecting the consequences of dose modifications will be provided to summarize the actual maintenance dose level against treatment groups. Actual maintenance dose is defined as the highest dose X such that the patient was dosed at dose X or higher for at least 80% of the dosing period from Week 64 to Week 72.

3.3. Efficacy Assessment

3.3.1. Primary Efficacy Endpoint Analysis

3.3.1.1. Definition of Endpoint(s)

The primary efficacy measure will be the percent change in body weight from baseline at Week 72.

The percent change in body weight at each nominal visit is defined as:

*(postbaseline body weight [kg] – baseline body weight [kg]) / baseline body weight [kg] * 100%*

3.3.1.2. Analytical Approach

Summary descriptives (n, mean, median, std, min and max) for percent change in body weight from double-blind baseline and OLE baseline will be presented by visit and treatment group separately. Non-missing data collected no later than end of treatment + 2 days will be included in the analysis.

3.3.2. Secondary Efficacy Endpoint(s) Analysis

3.3.2.1. Secondary Endpoints

The following secondary study endpoints will be analyzed using the data in the OLE Analysis Set:

- Percentage of participants who achieve ≥5% reduction in body weight at Week 72
- Percentage of participants who achieve ≥10% reduction in body weight at Week 72
- Percentage of participants who achieve ≥15% reduction in body weight at Week 72
- Change in body weight (absolute) from Baseline to Week 72

3.3.2.2. Analytical Approach

Change from double-blind baseline and OLE baseline in body weight will be summarized using descriptive statistics by visit and treatment group.

Summary statistics of percentage of participants with ≥5%, ≥10%, and ≥15% reduction will be based on observed data collected no later than end of treatment + 2 days at Week 72.

3.3.2.3. Exploratory Efficacy Analysis

The following exploratory study endpoints will be summarized using descriptive statistics by treatment group and visit on OLE Analysis Set.

- Change in triglycerides from baseline to Week 72
- Change in free fatty acids (FFA) from baseline to Week 72
- Change in high-sensitivity C-reactive protein (hsCRP) from baseline to Week 72
- Change in HDL cholesterol from baseline to Week 72
- Change in LDL cholesterol from baseline to Week 72
- Change in resting blood pressure from baseline to Week 72
- Change in fasting glucose from baseline to Week 72
- Change in HbA1c from baseline to Week 72

3.3.2.4. Pharmacokinetic and Pharmacokinetic/Pharmacodynamic Methods

Aleniglipron concentration data will be summarized and analyzed using a population PK approach via nonlinear mixed-effects modeling. The relationships between aleniglipron dose and/or concentration and selected efficacy, tolerability, and safety endpoints may be characterized. Additionally, the impact of intrinsic and extrinsic factors, such as age, weight, gender, and renal function on PK and/or PD parameters, may be examined as needed. Additional analyses may be conducted if they are deemed appropriate. Population PK and/or PD parameters and analysis will be described in a separate report.

3.3.2.5. Patient-Reported Outcomes

Short-Form-36 Health Survey Version 2, Acute Form

The following domain and component scores will be derived from the Short-Form-36 Health Survey Version 2, Acute Form (SF-36 v2):

- Mental Component Score
- Physical Component Score
- Physical Functioning domain
- Role-Physical domain
- Bodily Pain domain
- General Health domain
- Vitality domain
- Social Functioning domain
- Role-Emotional domain, and
- Mental Health domain

For each of the above parameters, the raw scores will be transformed into the domain scores (t- scores). Descriptive summaries by treatment group and visit for the actual value and change from baseline value will be provided.

Impact of Weight on Quality of Life-Lite Clinical Trial

The following parameters will be included from the Impact of Weight on Quality of Life-Lite Clinical Trial (IWQOL-Lite-CT):

- IWQOL-Lite-CT total score (all items: Items 1 through 20)
- Physical Function composite score (5 items: Items 1 through 3, 16, and 17)
- Physical composite score (7 items: Items 1 through 5, 16, and 17), and
- Psychosocial composite score (13 items: Items 6 through 15, 18, 19, and 20)

The IWQOL-Lite-CT total and composite scores range from 0 to 100, with higher scores reflecting better levels of functioning.

The IWQOL-Lite-CT scores are computed according to the IWQOL-Lite scoring rules (Kolotkin and Crosby 2002), as following:

- Each composite raw score will be calculated if a minimum of 50% of the items for that composite has a non-missing value; the total score will be calculated if a minimum of 75% of all 20 items has a non-missing value.
    - physical composite score: 4 of 7 items
    - physical function composite score: 3 of 5 items
    - psychosocial composite score: 7 of 13 items
    - IWQOL-Lite-CT total score: 15 of 20 items
- If the minimum required number of items are answered for a composite then:
    - Calculate the average of the valid non-missing responses corresponding to the items in the total or each composite (1 = “never” or “not at all true” and 5 = “always” or “completely true”)
    - The composite score is then calculated by transforming the raw composite score to the 0 (worst)-to-100 (best) metric using the following formula for every participant at each time point:

$$100\ (S_{max} - C_{avg})/(S_{max} - S_{min})$$

        - C_{avg} is the raw average score of all nonmissing item responses in the composite; this average must be a number between 1 and 5, inclusive
        - S_{max} is the maximum possible raw score value (i.e., 5)
        - S_{min} is the minimum possible raw score value (i.e., 1)
        - Inserting the maximum and minimum possible score values, the formula is reduced to $100\ (5 - C_{avg})/4$.

For the total and each composite score, descriptive summaries by treatment group and visit for the actual value and change from baseline value will be provided.

Hunger and Satiety Evaluation

The count and percentage of participants hunger and satiety evaluation response categories at each time point will be summarized by nominal visit, evaluation and

treatment group.

Score of each of bothersome symptoms listed below for participants who experienced bothersome symptoms since starting of medication or last visit will be summarized with descriptive statistics by visit and treatment group.

Bothersome symptom: nausea, vomiting, diarrhea, constipation, bloating, abdominal pain, headache, other.

3.4. Safety Assessment

Unless specified otherwise, safety and tolerability in the OLE phase will be assessed using the OLE Analysis Set by treatment group irrespective of adherence to study drug.

Safety evaluations will be based on the incidence, severity, seriousness, and relationship of AEs, vital signs, physical examination findings, clinical laboratory results, and ECGs.

Abnormalities in clinical laboratory parameters and the ECG parameter QTcF will be based on predefined normal ranges and will be tabulated by dose group showing participant counts and percentages.

3.4.1. Adverse Events (AEs)

All AEs will be coded to system organ class (SOC) and preferred term (PT) using MedDRA version 28.0. Treatment-emergent adverse events (TEAEs) are defined as AEs that start or worsen in severity after the first dose of study drug, and onset date is within 7 days after the last dose of study drug. The severity of each AE will be graded using the most current version of the National Cancer Institute Common Terminology Criteria for Adverse Events (NCI-CTCAE) (Version 5). Related events are defined as events that are related, possibly, or probably related to study drug. AEs with a missing relationship are considered related. At each level of subject summarization, a subject is classified according to the closest relationship if the subject reported one or more events. For events with a missing severity during the baseline period, it will be treated as “mild” in severity for determining treatment-emergence. Events with a missing severity during the postbaseline period will be treated as “severe” and treatment-emergence will be determined by comparing to baseline severity. A participant with multiple occurrences of an AE will be counted only once in the respective AE category. A patient with multiple CTCAE grades for the same PT will be summarized under the maximum CTCAE grade recorded for the event.

In AE summaries, the primary system organ class will be presented by descending order of frequency (alphabetically for ties), then PTs will be sorted by descending order of frequency within each system organ class (alphabetically for ties). The sort order for the PT will be based on their frequency. Adverse events of special interest (AESI) will include: major adverse cardiovascular event (MACE), heart failure syndrome, acute pancreatitis,

hypoglycemia, thyroid malignancies, supraventricular arrhythmia and cardiac conductive disorders, hepatobiliary disorders, severe gastrointestinal (GI) AEs, acute renal events, depressive disorder and/or suicide ideation, hypersensitivity, and C-cell hyperplasia. AESI will be identified as described in Appendix B.

An overview summary of AEs will be provided including counts and percentages of participants (and event counts) with the following:

- Any TEAEs
- Any study drug related TEAEs
- Any TEAEs of special interest (AESIs)
- Any serious AEs (SAEs)
- Any study drug related SAEs
- Any TEAEs leading to dose reduction
- Any TEAEs leading to dose interruption
- Any TEAEs leading to permanent discontinuation of study drug
- Any TEAEs leading to discontinuation from study
- Any AEs leading to death

The following TEAE summaries of number and percentage of participants as well as event counts will also be produced:

- TEAEs by SOC and PT
- TEAEs related to study drug by SOC and PT
- TEAEs by SOC, PT, and worst reported severity
- Treatment-emergent SAEs by SOC and PT
- Common TEAEs (frequency > 5% of all participants) by PT
- Treatment-emergent AESI by category, SOC and PT
- Serious AESI by category, SOC and PT
- TEAEs leading to study drug discontinuation by SOC and PT
- TEAEs leading to study discontinuation by SOC and PT
- Prevalence of GI-related TEAEs by week/titration step, including intermittent AE*
- Prevalence of GI-related TEAEs by week/titration step, excluding intermittent AE
- Prevalence of GI-related TEAEs by week/titration step, target dose level and maximum severity including intermittent AE
- Prevalence of GI-related TEAEs by week/titration step, target dose level and maximum severity excluding intermittent AE
- Incidence of GI-related TEAEs by week/titration step and severity, including intermittent AE
- Incidence of GI-related TEAEs by week/titration step and severity, excluding intermittent AE
- Prevalence of treatment-emergent nausea/vomiting/diarrhea/constipation by week/titration step and maximum severity, including intermittent AE
- Prevalence of treatment-emergent nausea/vomiting/diarrhea/constipation by

week/titration step and maximum severity, excluding intermittent AE

- Prevalence of treatment-emergent nausea/vomiting/diarrhea/constipation by week/titration step, target dose level and maximum severity, including intermittent AE
- Prevalence of treatment-emergent nausea/vomiting/diarrhea/constipation by week/titration step, target dose level and maximum severity, excluding intermittent AE
- Incidence of treatment-emergent nausea/vomiting/diarrhea/constipation by week/titration step and severity
- Incidence of treatment-emergent nausea/vomiting/diarrhea/constipation by week/titration step, target dose level and severity

*Intermittent AEs are AEs reported with “intermittent” or “occasionally” in the term.

Listings of all AEs, SAEs, AESIs, AEs leading to discontinuation of study drug, AEs leading to withdrawal from the study, and AEs leading to death will be presented by site, treatment group subject/sex/age(enrollment), SOC, PT, AE onset date/day, AE resolution date/day, severity, seriousness, AESI category, action taken, outcome, and drug relatedness.

3.4.2. Vital Signs

Observed values and changes from OLE baseline for vital signs (systolic and diastolic BP, and HR) will be summarized by treatment group and visit using descriptive statistics (n, mean, SD, median, minimum, and maximum).

Counts and percentages of participants with treatment-emergent abnormal sitting systolic blood pressure (SBP), sitting diastolic blood pressure (DBP), and pulse will be presented by treatment for participants who have both baseline and at least 1 postbaseline result. A treatment-emergent high result is defined as a change from a value less than or equal to the high limit at baseline to a value greater than the high limit at any time that meets the specified change criteria during the postbaseline period. A treatment-emergent low result is defined as a change from a value greater than or equal to the low limit at baseline to a value less than the low limit at any time that meets the specified change criteria during the postbaseline period. To assess decreases, change from the minimum value during the baseline period to the minimum value during the postbaseline period will be used. To assess increases, changes from the maximum value during the baseline period to the maximum value during the postbaseline period will be used. Both planned and unplanned measurements will be included in the analysis. The criteria for identifying participants with treatment-emergent vital sign abnormalities are stated in Table 5.

Table 5 Categorical Criteria for Abnormal Treatment-Emergent Blood Pressure and Heart Rate Measurements

Parameter	Low	High
Systolic BP (mm Hg) (Supine or sitting – forearm at heart level)	≤90 and decrease from baseline ≥20	≥139 and increase from baseline ≥20

Parameter	Low	High
Diastolic BP (mm Hg) (Supine or sitting – forearm at heart level)	≤50 and decrease from baseline ≥10	≥90 and increase from baseline ≥10
Hear rate (bpm) (Supine or sitting)	<50 and decrease from baseline ≥15	>100 and increase from baseline ≥15

Abbreviations: BP = blood pressure; bpm = beats per minute.

In addition, the following analyses will be conducted by treatment:

- counts and percentages of participants who had resting heart rate (HR) changes from baseline at 2 consecutive visits of more than two predefined thresholds:
    - 10 beats per minute (bpm)
    - 20 bpm
- counts and percentages of participants who had at least 1 resting HR exceeding 100 bpm,
  and
- counts and percentages of participants who had at least 1 resting HR exceeding 100 bpm occurring at 2 consecutive study visits.

Listings of vital signs will be presented.

3.4.3. Clinical Laboratory Data

Clinical laboratory safety data (hematology, serum chemistry, and coagulation) will be reported with units based on the reported values. If there are multiple reported units collected for the same parameter, the results will be reported in the International System of Unit. Normal reference ranges and abnormal results will be used in the summary of laboratory data.

Observed values and changes from OLE baseline for continuous clinical laboratory parameters will be summarized at each visit using descriptive statistics (n, mean, SD, median, minimum, and maximum) and tabulated for each treatment group.

Categorical clinical laboratory data will be summarized at each visit using participant counts and percentages and tabulated by treatment group. For categorical clinical laboratory data, the number and percentage of participants with normal and abnormal values will be summarized by treatment group.

Shift tables will present the number and percentage of participants within each baseline CTCAE 5.0 grade versus each postbaseline CTCAE 5.0 grade for lipase, amylase, calcitonin, aspartate aminotransferase, alanine transaminase, alkaline phosphatase, gamma-glutamyl transferase, total bilirubin, direct bilirubin, plasma glucose, serum creatinine and estimated glomerular filtration rate lab parameters by each treatment group. Values will be summarized by their worst post baseline grade.

Potential drug-induced liver injuries will be evaluated with liver test parameters ALT, AST

and total bilirubin. Number and percentage of participants will be tabulated with the following categories for pre-baseline and post-baseline period separately by treatment group:

ALT: >= 3 ULN; >= 5 ULN; >= 10 ULN

AST: >= 3 ULN; >= 5 ULN; >= 10 ULN

Total bilirubin >= 2 ULN

ALT or AST >= 3 ULN and total bilirubin >= 2 ULN

(Assumption: no elevated serum ALP, and no other disease or condition can be found to explain the abnormalities).

Individual participant profiles will be listed for any laboratory parameters with ≥1 postdose value outside the laboratory's reference ranges and deemed clinically significant. The listing will include participant identification, treatment group, laboratory collection date, study day, analyte name, and analyte finding.

3.4.4. Electrocardiograms

The mean value for the triplicate ECG will be utilized for the ECG analysis.

The following ECG parameters will be analyzed: PR interval, QT interval, QRS duration, QTcF, and ventricular HR. Observed values and changes from baseline for ECG parameters will be summarized at each timepoint using descriptive statistics (n, mean, SD, median, minimum, and maximum) and tabulated for each dose group.

For QTcF, the count and percentage of participants with values >450, >480, and >500 msec or an increase from baseline >30 and >60 msec will also be presented, in accordance with International Council for Harmonization of Technical Requirements for Pharmaceuticals for Human Use (ICH) E14 (note for guidance on the clinical evaluation of QT/corrected QT interval prolongation and proarrhythmic potential for non-antiarrhythmic drugs, Committee for Medicinal Products for Human Use/ICH/2/04).

For HR, the number of participants with values ≥100 bpm, or an increase from baseline ≥ 20 bpm will also be presented by treatment group.

Individual ECG data will be listed.

3.4.5. Physical Examination Data

Physical examination data will be listed only. Any postdose clinically significant findings will be reported as AEs.

3.5. Pharmacokinetic Assessment

3.5.1. Sample Collections for Pharmacokinetic Analysis

Blood samples will be collected for assessment of plasma concentrations of aleniglipron. Clinical staff is required to collect each blood PK sample at the scheduled timepoints within the windows indicated (Appendix D). The actual date and time (as hh:mm) of each blood

sample collection will be recorded in the eCRF.

3.5.2. Handling Missing or Below the Lower Limit of Quantification Data

If an aleniglipron concentration for a PK sample is missing, the record will be excluded from this analysis. If the date or time for a PK sample or for the dose immediately prior to a PK sample is missing, the relevant concentration will either be deleted from the analysis dataset, or, if feasible, the date or time will be imputed according to protocol specifications or based on a logical interpretation of available information from data proximate to the missing data.

The timing and frequency of concentration measurements reported as below the lower limit of quantitation (BLQ) will be summarized. If BLQ samples (post administration of the drug) represent less than or equal to 5% of the total samples in the dataset, and if the occurrence of BLQ samples does not appear to be treatment-group dependent, all BLQ values will be removed from the analysis dataset prior to analysis. This method corresponds to the M1 method recommended by Beal as appropriate for many datasets. If BLQ samples represent greater than 5% of the data or if the occurrence of BLQ samples is substantially treatment-group dependent, an alternative procedure may be considered for implementation based upon a strategy discussed by Gasherbrum Bio and the vendor for population PKPD analysis.

3.5.3. Pharmacokinetic Concentration

Individual aleniglipron concentration data will be listed. Individual plasma concentrations of aleniglipron may be summarized by treatment group for each dose level for the Pharmacokinetic Analysis Set descriptively (n, mean, SD, coefficient of variation, minimum, maximum, median, geometric mean [GM], and geometric coefficient of variation).

3.5.4. Pharmacokinetic Parameters

This study only collects sparse samples for PK analysis. Non compartmental analysis (NCA) will not be performed. A population PKPD analysis will be performed and report separately.

3.6. Pharmacodynamic Assessment

The PD effect of aleniglipron will be assessed on the following variables (including and not limited to):

Cardiovascular PD markers measured at Week 60 and Week 72:

- Free fatty acid (FFA)
- High-sensitivity C- reactive protein (hsCRP)

Other clinically relevant markers measured on Week 60 and Week 72:

- Triglycerides

- Total cholesterol
- Low-density lipoprotein (LDL) cholesterol
- High-density lipoprotein (LDL) cholesterol

Descriptive analysis (n, mean, SD, median, minimum, and maximum) will be generated on absolute value and change from OLE baseline (FFA and hsCRP) of the above variables by treatment group and timepoint on the PD analysis sets.

4. ANALYSIS TIMING

4.1. Interim Analysis

Interim analysis (efficacy and safety) will be conducted for the OLE phase after all participants complete double-blind phase. The interim analysis will include all data up to October 30, 2025. There will be no action taken with respect to the study conduct and analysis. There will be no inflation of the Type 1 error rate and no need to employ an alpha spending function or multiplicity adjustment. A select group of Sponsor personnel will review the interim analysis for safety and efficacy.

A list of outputs for the OLE interim analysis is attached in Appendix F.

4.2. Final Analysis

After the database is locked, the randomized treatment assignments will be unblinded and the pre-final TFLs will be generated. After all comments on the pre-final analysis have been resolved and the study database is declared final, the final TFLs for the final analysis will be generated. If there were no changes to the pre-final analysis or the study database, the pre-final TFLs may be considered final. Results presented in graphical and tabular presentations will be structured in alignment with CDISC standards.

5. CHANGES FROM PROTOCOL-SPECIFIED STATISTICAL ANALYSES

This SAP supersedes the statistical analysis described in the protocol amendment 4 signed on May 07, 2025.

6. APPENDIX

6.1. Appendix A: Overall Schedule of Assessments for Open-Label Extension

Study periods	Treatment period									EOS /ETV[j]
Visit identifier	10 OLE	11	12	13	14	15	16	17	18	19
Visit day	280	308	336	364	392	420	448	476	504	511
Visit week	40	44	48	52	56	60	64	68	72	73
Visit Interval Window (days)	±3	±3	±3	±3	±3	±3	±3	±3	±3	±3
Assessments										
Body weight (with certified calibrated scales and in hospital gown) in triplicate	X	X	X	X	X	X	X	X	X	X
Waist circumference in triplicate						X			X	X
Concomitant medication	X	X	X	X	X	X	X	X	X	X
Physical examination (full)										X
Physical examination (symptom-directed)	X	X	X	X	X	X	X	X	X	
Vital signs (BP, HR, temperature) [a]	X	X	X	X	X	X	X	X	X	X
12-Lead ECG [b]	X	X	X	X	X	X	X		X	X
Pregnancy test (urine) before dosing [c]	X	X	X	X	X	X	X	X	X	X
Clinical laboratory tests [d]	X	X	X	X	X	X	X	X	X	X
Adverse events [e]	X	X	X	X	X	X	X	X	X	X
Dose administration [f]	X	X	X	X	X	X	X	X	X	
HbA1c						X			X	
PK sampling (blood) only for participants who were on placebo [g]	X									
Cardiovascular PD biomarkers (blood)[h]						X			X	
Fill out electronic diary daily, including dose administration time	X	X	X	X	X	X	X	X	X	
SF-36 acute version				X					X	X
IWQOL-Lite CT				X					X	X
Hunger and satiety evaluation[i]	X	X	X	X	X	X	X	X	X	X
PHQ-9 health questionnaires	X	X	X	X	X	X	X	X	X	X
C-SSRS since the last visit	X	X	X	X	X	X	X	X	X	X
Discuss nutrition plan and physical activity[k]	X	X	X	X	X	X	X	X	X	

Abbreviations: BP=blood pressure; C-SSRS=Columbia Suicide Severity Rating Scale; ECG=electrocardiogram; EOS=end of study; ETV=early termination visit; HbA1c=hemoglobin A1c; HR=heart rate; IWQOL-Lite CT=Impact of Weight on Quality of Life-Lite Clinical Trials Version; OLE=open-label extension; PD=pharmacodynamic; PHQ-9=Patient Health Questionnaire-9; PK=pharmacokinetic; SF36=Short Form 36 health survey acute version.

a. Vital signs including blood pressure (in triplicate), heart rate, and temperature (temperature at Screening and Day 1only) will be collected predose. BP will be collected from the left arm in at least 2-minute intervals. See Section 8 for order of procedures. Caffeine-containing products (e.g., coffee, tea, chocolate, and

caffeine-containing sodas, colas, etc.) may not be consumed within 2 hours before measuring vital signs and ECGs. Predose vital signs assessments should be performed within 1 hour before dosing.
b. ECGs will be collected predose and stored centrally. At Weeks 38 and 40, ECGs will be collected in triplicate along with each PK draw and within 15 minutes before the PK draw. See Section 8 for order of procedures.
c. For women of child-bearing potential, a urine pregnancy test is performed first, and if positive or inconclusive, confirmed with a serum pregnancy test.
d. Clinical safety laboratory tests will include hematology, serum chemistry, and coagulation. See Section 10.2. Norovirus and/or influenza testing may be conducted as standard of care as needed.

e. Adverse events and serious adverse events collected from time of signing the informed consent form through 30 days after date of the last dose.
f. Up-titrations will occur at Weeks 40, 44, and 48 for participants rolling over to the OLE from Cohort 1, and at Week 40, 44, 48, 52, 56, 60, and 64 for those who were on placebo.
g. For PK timepoints, refer to Table 4. See Section 8 for order of procedures.
h. PD markers, including, but not limited to, free fatty acid, IL-6, ProBNP, high-sensitivity C-reactive protein, and succinate, will be measured.
i. The hunger and satiety evaluation will be performed for the first 7 days following each clinic visit, except for /EOS/ETV which will be evaluated on the day of the visit only. If the hunger and satiety evaluation is received with triggering results, site staff should conduct an unscheduled phone call visit to evaluate the condition of the participant's adverse event.
j. ETV should be scheduled 7 (±3) days from the last dose.
k. There will be a discussion on nutrition approximately 3 days prior to each up-titration visit in addition to a documented discussion on nutrition plan and physical activity at the clinic visit.

Note: Participants who discontinue study drug should return to the clinic at the next scheduled visit for the ETV assessments.

6.2. Appendix B: Searching Criteria for AESIs

Event	Criteria/Search Term
**MACE**	Adjudication
**Heart failure syndrome**	Adjudication
**Acute pancreatitis**	Adjudication
**Thyroid malignancies**	Adjudication
**Cholecystitis**	Adjudication
**Hypoglycemia**	MedDRA PT of hypoglycemia
**Supraventricular arrhythmia and cardiac conductive disorders**	Arrhythmia related investigations, signs, and symptoms SMQ (20000051), narrow and broad terms<br>Supraventricular tachyarrhythmias SMQ (20000057), broad and narrow terms<br>Tachyarrhythmia terms, nonspecific SMQ (20000164), narrow terms only<br>Ventricular tachyarrhythmias SMQ (20000058), narrow terms only<br>Conduction defects SMQ (20000056), narrow terms only<br>Cardiac conduction disorders HLT (10000032), all PTs
**Acute Renal Event**	Narrow terms in Acute renal failure SMQ (20000003)
**Hypersensitivity**	Anaphylactic reaction SMQ (20000021)<br>Hypersensitivity SMQ (20000214)<br>Angioedema SMQ (20000024)
**Hepatobiliary Disorder**	Broad and narrow terms in the Liver related investigations, signs and symptoms SMQ (20000008)<br>Broad and narrow terms in the Cholestasis and jaundice of hepatic origin SMQ (20000009)<br>Broad and narrow terms in the Hepatitis non-infections SMQ (20000010)<br>Broad and narrow terms in the Hepatic failure, fibrosis and cirrhosis and other liver damage SMQ (20000013)<br>Narrow terms in the Liver-related coagulation and bleeding disturbances SMQ (20000015)<br>Narrow PTs in Gallbladder related disorders SMQ (20000124)<br>Narrow PTs in Biliary tract disorders SMQ (20000125)<br>Narrow PTs in Gallstone related disorders SMQ (20000127)
**Severe GI AEs**	SOC of GI of grade 3 or higher
**C-cell hyperplasia**	MedDRA PT of thyroid C-cell hyperplasia

Event	Criteria/Search Term
**Depressive disorder and/or suicide ideationep**	Narrow terms from the Depression and suicide/self-injury SMQ as defined in MedDRA SMQs 20000037 [Suicide/self-injury] and 200000167 [Depression (excluding suicide and self-injury)]

6.3. Appendix C: Clinical Laboratory Tests

**Hematology**	**Serum Chemistry, Including Liver Function and Urinalysis**		
Hemoglobin<br>Hematocrit<br>Red blood cell indices<br>Thrombocyte count (platelets)<br>WBC count (absolute and %) with differential<br>**Coagulation**<br>Prothrombin time INR<br>aPTT<br>Fibrinogen	Sodium<br>Potassium<br>Chloride<br>Bicarbonate<br>Phosphate<br>Calcium<br>Glucose<br>Amylase<br>Lipase<br>Uric acid<br>Magnesium	Creatine kinase<br>Creatinine<br>eGFR calculated using CKD-EPI formula Blood urea nitrogen Alanine transaminase<br>Alkaline phosphatase<br>Aspartate transaminase<br>Gamma-glutamyl transferase<br>Total bilirubin<br>Conjugated bilirubin<br>Unconjugated bilirubin	Total cholesterol<br>High-density lipoprotein Low-density lipoprotein<br>Triglycerides<br>Calcitonin Albumin Globulins Total protein<br>Lactate dehydrogenase Thyroid stimulating hormone<br>hemoglobin A1c<br>high-sensitivity C-reactive protein
**Virology**	**Drug Screen (Serum or Urine)** [b]		**Confirmation of Postmenopausal Status**
HIV-1/-2 antibody HbsAg<br>Hepatitis C virus antibody and viral load<br>COVID-19, SARS-COV-2 antibody[a]	Methamphetamines Opiates<br>Cocaine<br>Benzodiazepines	Barbiturates<br>Methadone<br>Tricyclic antidepressants<br>Amphetamines	FSH for postmenopausal women<br>Pregnancy test Serum hCG [c] Urine hCG [c]

Abbreviations: aPTT = activated partial prothrombin time; CKD-EPI = Chronic Kidney Disease Epidemiology Collaboration; COVID-19 = coronavirus disease 2019; eGFR = estimated glomerular filtration rate; FSH = follicle-stimulating hormone; HbsAg = hepatitis B surface antigen; hCG = human chorionic gonadotropin; HIV = human immunodeficiency virus; INR = international normalized ratio; PCR = polymerase chain reaction; SARS-COV-2 = severe acute respiratory syndrome coronavirus 2; WBC = white blood cell.

a. COVID-19 test performed only if clinically indicated as determined by the Investigator; COVID-19 antigen test is performed first, and then PCR test for confirmation if clinically indicated.

b. Either serum or urine drug screening is acceptable according to site routine procedure but should be kept consistent throughout the study.

c. If the urine hCG is positive or inconclusive, pregnancy will be confirmed by a serum hCG test.

6.4. Appendix D: Pharmacokinetic Sampling Schedule for Participants Who Were on Placebo Moving into Open-Label Extension

Study Day	Study Week	Planned Dose (Irrespective of Down-Titration)	PK Timepoints (Exact Time of Blood Collection Must be Recorded)	ECG
Day 280	Week 40	Last dose of 2.5 mg QD (will be up titrated to 5 mg on Day 281)	Within 45 minutes before dosing<br>Between 2 and 4 hours post dose	Yes

Abbreviations: ECG=electrocardiogram; PK=pharmacokinetic; QD=once daily.
Note: On-study days with PK assessments, all study tablets should be consumed at the clinic within 15 minutes. Note: ECGs will be collected in triplicate, along with each PK draw and within 15 minutes before the PK draw. See the Site Procedural Manual for details.

6.5. Appendix E: Imputation Rules for Missing AE and Medication Dates

An imputation of end date should be performed before an imputation of start date.

6.5.1. Imputation Rules for AE End Date and Medication End Date

If the end date (AE end date, medication end date) is partially missing or completely missing, the following rules will be applied:

- If the end date is completely missing and not ongoing, then the end date will be imputed as the end of study (EOS) date.
- Partial end date where only the year is known:
  - The end date will be imputed as the EOS date if the year of end date is the same as the year of the EOS date; otherwise, day and month of end date will be imputed as the last day of the year (i.e., 31 December).
- Partial end date where only the year and month are known:
  - The end date will be imputed as the EOS date if the year and month of end date are the same as year and month of the EOS date; otherwise, day of end date will be imputed as the last day of the month.

6.5.2. Imputation Rules for AE Start Date and Medication Start Date

If the start date (AE start date, medication start date) is partially missing or completely missing, the following rules will be applied:

- If the start date is completely missing, then the start date will be imputed as the first dose date.
- Partial start date where only the year is known:
  - The start date will be imputed as the first dose date if the year of start date is the same as the year of the first dose date; otherwise, day and month of start date will be imputed as the first day of the year (i.e., 01 January).
- Partial start date where only the year and month are known:
  - The start date will be imputed as the first dose date if the year and month of start date are the same as year and month of the first dose date; otherwise, day of start date will be imputed as the first day of the month.
- If the imputed start date is after the end date, the start date will be imputed as the end date.
- If the imputed AE start date is before the informed consent date, the AE start date will be imputed as the informed consent date.

6.6. Appendix F: List of TLFs for OLE Interim Analysis

Table Number	Title
Table 14.1.1	Disposition
Table 14.1.2	Demographic and Baseline Characteristics
Table 14.1.5.3	Summary of Dose Modification
Table 14.2.1.1.1	Summary of Body Weight - DB Baseline
Table 14.2.1.1.2	Summary of Body Weight - OLE Baseline
Table 14.3.1.1	Overview of Treatment-Emergent Adverse Events
Table 14.3.1.2	Treatment-Emergent Adverse Events by System Organ Class and Preferred Term
Table 14.3.1.4	Treatment-Emergent Adverse Events by System Organ Class, Preferred Term, and Worst Reported Severity
Table 14.3.2.1	Treatment-Emergent Serious Adverse Events by System Organ Class and Preferred Term
Table 14.3.2.5.1	Treatment-Emergent Adverse Events Leading to Discontinuation of Study Drug by System Organ Class and Preferred Term
Table 14.3.2.5.2	Treatment-Emergent Adverse Events Leading to Dose Reduction of Study Drug by System Organ Class and Preferred Term
Table 14.3.2.5.3	Treatment-Emergent Adverse Events Leading to Dose Interruption of Study Drug by System Organ Class and Preferred Term
Table 14.3.2.5.4	Treatment-Emergent Adverse Events Leading to Discontinuation of Study by System Organ Class and Preferred Term
Table 14.3.3.1.3.1	Prevalence of Treatment-emergent Nausea/Vomiting/Diarrhea/Constipation, by Week/Titration Step, Target Dose Level, and Maximum Severity, including Intermittent AE
Table 14.3.3.1.3.2	Prevalence of Treatment-emergent Nausea/Vomiting/Diarrhea/Constipation, by Week/Titration Step, Target Dose Level, and Maximum Severity, excluding Intermittent AE
Table 14.3.3.1.5	Incidence of Treatment-emergent Nausea/Vomiting/Diarrhea/Constipation, by Week/Titration Step, Target Dose Level, and Severity
Table 14.3.4.2.2	Clinical Laboratory – Serum Chemistry
Table 14.3.4.2.7	Evaluation of Potential Drug-induced Liver Injury
Table 14.3.4.3.2	Specified Abnormal Electrocardiogram Findings

7. REFERENCES

1. Pharmaceutical Statistics Using SAS: A Practical Guide (SAS Press) 1st Edition by Alex Dmitrienko (Author), Christy Chuang-Stein
2. Anwar YA, White WB. Ambulatory monitoring of the blood pressure devices, analysis, and clinical utility. In: William White, editor. Blood Pressure Monitoring in Cardiovascular Medicine and Therapeutics. Humana Press, Inc; 2001: p 64
3. Darken P, Nyberg J, Ballal S, Wright D. The attributable estimand: a new approach to account for intercurrent events. *Pharm Stat*. 2020;19(5):626-635. https://doi.org/10.1002/pst.2019
4. [FDA] E9(R1) Statistical principles for clinical trials: Addendum: Estimands and sensitivity analysis in clinical trials. https://www.fda.gov/media/148473/download
5. [EMA] European Medicines Agency. ICH E9 (R1) addendum on estimands and sensitivity analysis in clinical trials to the guideline on statistical principles for clinical trials. Step 5. EMA/CHMP/ICH/436221/2017. Published February 17, 2020. Accessed June 30, 2022. https://www.ema.europa.eu/en/documents/scientific-guideline/ich-e9-r1-addendum-estimands- sensitivity-analysis-clinical-trials-guideline-statistical-principles_en.pdf
6. Ernst ME, Bergus GR. Noninvasive 24-hour ambulatory blood pressure monitoring: overview of technology and clinical applications. *Pharmacotherapy*. 2002;22(5):597-612. https://doi.org/10.1592/phco.22.8.597.33212
7. Kolotkin RL, Crosby RD. Psychometric evaluation of the impact of weight on quality of life-lite questionnaire (IWQOL-lite) in a community sample. *Qual Life Res*. 2002;11(2):157-71. https://doi.org/10.1023/a:1015081805439
8. Wharton S, Blevins T, Connery L, et al; GZGI Investigators. Daily oral GLP-1 receptor agonist orforglipron for adults with obesity. *N Engl J Med*. 2023; 389:877-888. [PMID: 37351564] doi: 10.1056/NEJMoa2302392
9. Lipkovich, I, Ratitch, B, Mallinckrodt, CH. Causal Inference and Estimands in Clinical Trials. *Stat. in Biopharm Research*. 2020; 12:54-67. https://doi.org/10.1080/19466315.2019.1697739
10. Pickering TG, Hall JE, Appel LJ, et al. Recommendations for blood pressure measurement in humans and experimental animals. Part 1: blood pressure measurement in humans: a statement for professionals from the Subcommittee of Professional and Public Education of the American Heart Association Council on High Blood Pressure Research. *Hypertension*. 2005;45(1):142-161. https://doi.org/10.1161/01.HYP.0000150859.47929.8e
