# Supplementary material for: Oral small molecule GLP-1 receptor agonist aleniglipron in people with overweight or obesity: a randomized, double-blind, placebo-controlled phase 2b trial
Source: Nat Med. 2026 Jun 5;32(8):2941–7. doi: 10.1038/s41591-026-04476-6 (PMC13472849; doi:10.1038/s41591-026-04476-6)
Supplement: Supplementary file 2 [file 41591_2026_4476_MOESM2_ESM.pdf]

nature portfolio

Corresponding author(s): Julio Rosenstock

Last updated by author(s): May 14, 2026

Reporting Summary

Nature Portfolio wishes to improve the reproducibility of the work that we publish. This form provides structure for consistency and transparency in reporting. For further information on Nature Portfolio policies, see our Editorial Policies and the Editorial Policy Checklist.

Statistics

For all statistical analyses, confirm that the following items are present in the figure legend, table legend, main text, or Methods section.

n/a	Confirmed	
☐	☒	The exact sample size (*n*) for each experimental group/condition, given as a discrete number and unit of measurement
☐	☒	A statement on whether measurements were taken from distinct samples or whether the same sample was measured repeatedly
☐	☒	The statistical test(s) used AND whether they are one- or two-sided<br>*Only common tests should be described solely by name; describe more complex techniques in the Methods section.*
☐	☒	A description of all covariates tested
☐	☒	A description of any assumptions or corrections, such as tests of normality and adjustment for multiple comparisons
☐	☒	A full description of the statistical parameters including central tendency (e.g. means) or other basic estimates (e.g. regression coefficient) AND variation (e.g. standard deviation) or associated estimates of uncertainty (e.g. confidence intervals)
☐	☒	For null hypothesis testing, the test statistic (e.g. *F*, *t*, *r*) with confidence intervals, effect sizes, degrees of freedom and *P* value noted<br>*Give P values as exact values whenever suitable.*
☐	☒	For Bayesian analysis, information on the choice of priors and Markov chain Monte Carlo settings
☐	☒	For hierarchical and complex designs, identification of the appropriate level for tests and full reporting of outcomes
☒	☐	Estimates of effect sizes (e.g. Cohen's *d*, Pearson's *r*), indicating how they were calculated

Our web collection on statistics for biologists contains articles on many of the points above.

Software and code

Policy information about availability of computer code

Data collection	MediData Rave was used for electronic data collection (no version numbers).
Data analysis	All data processing, summarization and analyses were performed using SAS® Enterprise Guide Version 8.2 or higher statistical software package.

For manuscripts utilizing custom algorithms or software that are central to the research but not yet described in published literature, software must be made available to editors and reviewers. We strongly encourage code deposition in a community repository (e.g. GitHub). See the Nature Portfolio guidelines for submitting code & software for further information.

Data

Policy information about availability of data

All manuscripts must include a data availability statement. This statement should provide the following information, where applicable:

- Accession codes, unique identifiers, or web links for publicly available datasets
- A description of any restrictions on data availability
- For clinical datasets or third party data, please ensure that the statement adheres to our policy

De-identified individual patient data for variables necessary to address a specific research question may be shared with an approved data sharing request. Researchers will submit a request containing the research objectives, endpoints/outcomes of interest, statistical analysis plan, data requirements, publication plan, and qualifications of the researcher(s). Gasherbrum Bio, Inc. does not grant external requests for individual de-identified patient data for the following purposes:

• re-evaluating safety and efficacy end points already addressed in the product labelling,
• assessing safety or efficacy for an indication in current development
Requests are reviewed by a committee of internal advisors, and if not approved, may be further arbitrated by a panel of external advisors. Upon approval, information necessary to address the research question will be provided under the terms of a data sharing agreement.
Data sharing requests will be considered beginning 36 months after the study publication (manuscript accepted for publication) and either 1) the product has been granted marketing authorization in at least two regulatory jurisdictions, or 2) clinical development for the product and/or indication discontinues and the data will not be submitted to regulatory authorities. There is no end date for eligibility to submit a data sharing request for this study. Access can be requested from info@structuretx.com. Requests will be acknowledged and reviewed within 60 days of receipt of request.

Research involving human participants, their data, or biological material

Policy information about studies with human participants or human data. See also policy information about sex, gender (identity/presentation), and sexual orientation and race, ethnicity and racism.

Reporting on sex and gender	Sex was determined by self-reporting; we did not collect alternative data to reconcile sex and gender. The Informed Consent Form signed by all participants described that data collected during the study, including sex of participants, may be shared as part of study reporting, but with no personal identifying information attached.
Reporting on race, ethnicity, or other socially relevant groupings	Race and ethnicity were self reported, and included:<br>White<br>Black or African American<br>Asian<br>Multiple<br>Not Hispanic or Latino<br>Hispanic or Latino
Population characteristics	Key covariates in the therapeutic area of chronic weight management were collected:<br>Baseline BMI<br>• ≤35 kg/m2<br>• >35 kg/m2<br>Sex<br>• Male<br>• Female
Recruitment	The study was conducted at 38 clinical trial sites across the US that use conventional recruiting strategies including previously existing potential participants database, media outreach.<br>Self selection may create bias in that those who volunteer for clinical trials may have different motivations compared with patients who may use a drug, if approved, in the real world. A double-blind, placebo-controlled trial helps to alleviate the impact of self-selection bias.
Ethics oversight	A central IRB, Advarra, was used for ethics oversight. The conduct of this clinical study met all local legal and regulatory requirements. The study was conducted in accordance with the ethical principles that have their origin in the Declaration of Helsinki and the International Council for Harmonisation of Technical Requirements for Pharmaceuticals for Human Use (ICH) guideline E6: Good Clinical Practice (GCP).Participants were reimbursed for travel, meals, and other study-related expenses in accordance with IRB approval.

Note that full information on the approval of the study protocol must also be provided in the manuscript.

Field-specific reporting

Please select the one below that is the best fit for your research. If you are not sure, read the appropriate sections before making your selection.

☒ Life sciences ☐ Behavioural & social sciences ☐ Ecological, evolutionary & environmental sciences

For a reference copy of the document with all sections, see nature.com/documents/nr-reporting-summary-flat.pdf

Life sciences study design

All studies must disclose on these points even when the disclosure is negative.

Sample size	We estimated that 230 participants would provide >90% power to detect a treatment difference of -5% body weight change between the aleniglipron treatment group and placebo from baseline to Week 36, assuming an SD of 6%, a 2-sided alpha level of 0.05, 35 completers for one aleniglipron treatment group and 35 completers for the placebo group.
Data exclusions	The full analysis set included all randomized participants who received ≥1 dose of aleniglipron or placebo and had ≥1 post dose assessment. The safety analysis set included all participants who received ≥1 dose of aleniglipron or placebo. Safety and tolerability data are presented for all participants in the safety analysis set. One patient randomized to aleniglipron 120 mg treatment group did not receive any study treatment and was excluded from the efficacy and safety analysis population

Replication	The reproducibility of findings was supported by the use of a predefined protocol and statistical analysis plan, standardized study procedures, and systematic data monitoring to ensure consistency and data integrity. Full methodological and analytic details are provided in the protocol and SAP. No experimental replication was required.
Randomization	Participants were randomized to three dose level cohorts (45, 90, or 120 mg) at a ratio of 3:4:4, then to aleniglipron or placebo at a ratio of 3:1 within each cohort. Randomization was stratified by baseline BMI (≤35 kg/m2/>35 kg/m2) and sex.
Blinding	All participants and study personnel were blinded to study drug assignment.

Reporting for specific materials, systems and methods

We require information from authors about some types of materials, experimental systems and methods used in many studies. Here, indicate whether each material, system or method listed is relevant to your study. If you are not sure if a list item applies to your research, read the appropriate section before selecting a response.

Materials & experimental systems

n/a	Involved in the study	
☒	☐	Antibodies
☒	☐	Eukaryotic cell lines
☒	☐	Palaeontology and archaeology
☒	☐	Animals and other organisms
☐	☒	Clinical data
☒	☐	Dual use research of concern
☒	☐	Plants

Methods

n/a	Involved in the study	
☒	☐	ChIP-seq
☒	☐	Flow cytometry
☒	☐	MRI-based neuroimaging

Clinical data

Policy information about clinical studies

All manuscripts should comply with the ICMJE guidelines for publication of clinical research and a completed CONSORT checklist must be included with all submissions.

Clinical trial registration	ClinicalTrials.gov identifier NCT06693843
Study protocol	The full protocol will be made available as a supplemental file submitted with this manuscript.
Data collection	Between October 28, 2024, and February 7, 2025, 406 adults were screened, and 230 participants underwent randomization across 38 sites in the US. Site locations are listed in Supplementary Table 1 with the manuscript.
Outcomes	Endpoints were pre-defined and guided by the FDA draft guidance "Obesity and Overweight: Developing Drugs and Biological Products for Weight Reduction."<br>The primary endpoint (efficacy estimand) was the percent change in body weight from baseline to Week 36. The secondary endpoints were percentages of participants who achieved ≥5%, ≥10%, and ≥15% reductions in body weight at Week 36, and changes in body weight (absolute), waist circumference, and BMI from baseline to Week 36. Additional secondary safety endpoints were TEAEs, SAEs, adverse events of special interest (AESI), laboratory parameters, electrocardiograms, and vital signs.<br><br>The primary efficacy estimand, pre-defined in the statistical analysis plan, estimates the treatment effect of aleniglipron relative to placebo at Week 36 in participants who met the inclusion criteria and would have completed the treatment period. Data collected before the occurrence of any intercurrent events (ICE) (+2 days) were used for the analysis. ICE were defined as permanent discontinuation of study drug, initiation of other anti-obesity medication, or bariatric surgery; data subsequent to ICE were handled by the hypothetical strategy, regarded as invalid and set to missing. Missing data were imputed implicitly. The primary endpoint based on the efficacy estimand was analyzed using a mixed model for repeated measures (MMRM) with treatment group, randomization strata (baseline BMI and sex), visit, treatment-by-visit interaction, and baseline measurement as covariates. An unstructured covariance model was used.The continuous endpoints based on the 2 estimands were analyzed using an MMRM with treatment group, randomization strata (baseline BMI and sex), visit, treatment-by-visit interaction, and baseline measurement as covariates. An unstructured covariance model was used. Using the MMRM model, missing data was imputed implicitly. This analysis is deemed unbiased under the missing at random assumption. For the body weight categorical endpoints, logistic regression was used to analyze the data. Missing values at Week 36 were imputed according to the multiple-imputation approach, and values were combined using Rubin's rule.<br>All tests of treatment effects were conducted at a 2-sided alpha level of 0.05, and 2-sided 95% CIs were calculated. Lipid parameters, fasting glucose, and hsCRP were log-transformed before fitting the MMRM as described in the supplementary material.<br>Because of the exploratory nature of the OLE study, no estimand or model-based approach was planned. Change from double-blind baseline in body weight was summarized based on observed data. Summary descriptives (n, mean, median, std, min and max) were presented by visit and treatment group separately.

Plants

Seed stocks

Report on the source of all seed stocks or other plant material used. If applicable, state the seed stock centre and catalogue number. If plant specimens were collected from the field, describe the collection location, date and sampling procedures.

Novel plant genotypes

Describe the methods by which all novel plant genotypes were produced. This includes those generated by transgenic approaches, gene editing, chemical/radiation-based mutagenesis and hybridization. For transgenic lines, describe the transformation method, the number of independent lines analyzed and the generation upon which experiments were performed. For gene-edited lines, describe the editor used, the endogenous sequence targeted for editing, the targeting guide RNA sequence (if applicable) and how the editor was applied.

Authentication

Describe any authentication procedures for each seed stock used or novel genotype generated. Describe any experiments used to assess the effect of a mutation and, where applicable, how potential secondary effects (e.g. second site T-DNA insertions, mosiacism, off-target gene editing) were examined.
